# Aryl Aldehyde-Anchored Small Molecules Recruit FBXO22 for Targeted Degradation of NSD2

**DOI:** 10.1021/acs.jmedchem.6c00020

**Published:** 2026-06-29

**Authors:** Hua Tang, Yaxian Liao, Tsung-Yu Yeh, Kazuya Nishibayashi, Milad Rouhimoghadam, Ka Yang, Chunrong Li, Regina Stasser de Gonzalez, Yuan Zhao, Nina J. Hawkins, Justin M. Reitsma, Steven P. Gygi, Weiping Tang

**Affiliations:** † Lachman Institute for Pharmaceutical Development, School of Pharmacy, 5228 University of Wisconsin-Madison, Madison, Wisconsin 53705, United States; ‡ Department of Chemistry, University of Wisconsin–Madison, Madison, Wisconsin 53706, United States; § Technology & Therapeutic Platforms, AbbVie Incorporated, North Chicago, Illinois 60064, United States; ∥ Department of Cell Biology, Harvard Medical School, Boston, Massachusetts 02115, United States

**Keywords:** protein, degradation, E3, ligase, PROTAC, NSD2, aldehyde, FBXO22, covalent, ligand

## Abstract

Targeted protein degradation (TPD) has emerged as a transformative strategy in drug discovery, yet the repertoire of E3 ligase recruiters remains limited. Here, we report the discovery of an aldehyde-anchored PROTAC that covalently engages the E3 ligase FBXO22 to induce degradation of the histone methyltransferase NSD2 and CDK12. Competitive electrophile screening identified a phenyl aldehyde warhead as optimal, with SAR studies revealing that degradation is highly sensitive to the steric and electronic environment of the aldehyde moiety. The lead degrader, T9, effectively and selectively induces NSD2 degradation across multiple cancer cell lines. Mechanistic investigations confirmed that degradation is dependent on FBXO22, the ubiquitin–proteasome system, and the neddylation pathway, with mutagenesis identifying Cys326 as the critical residue for covalent engagement. This work establishes a stable covalent ligand for FBXO22, expanding chemical space of PROTAC design by introducing an accessible aldehyde-based E3 ligase ligand with broad potential for protein degradation.

## Graphical abstract

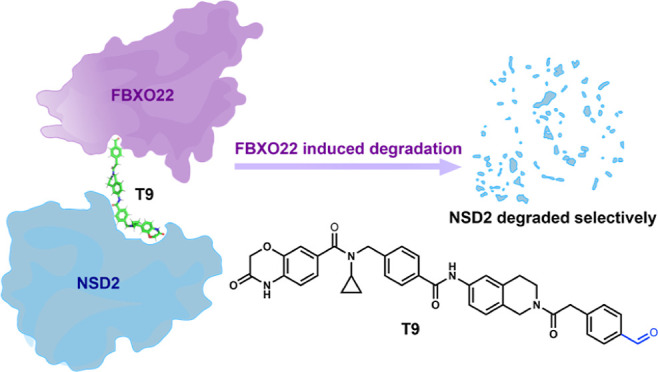

## Introduction

Targeted Protein Degradation (TPD) has emerged as a groundbreaking strategy in drug discovery, particularly for overcoming the limitations of traditional inhibitors and enabling access to a broad class of disease-relevant proteins that were once considered “undruggable” due to their lack of enzymatic activity or suitable functional binders.^1^ Unlike conventional small-molecule inhibitors that transiently block protein function, TPD exploits the cell’s endogenous degradation machinery to irreversibly eliminate disease-driving proteins.^2^ Two major degradation systems operate in mammalian cells: the autophagy–lysosomal pathway and the ubiquitin–proteasome system (UPS),^3^ with the latter serving as the primary route for protein degradation. By levering these natural pathways, TPD holds tremendous potential for transforming treatment options and improving patient outcomes.

Among UPS-dependent modalities, proteolysis-targeting chimeras (PROTACs) have garnered substantial interest. These heterobifunctional molecules consist of a ligand for the protein of interest (POI), a ligand for an E3 ubiquitin ligase, and a linker that connects the two. PROTACs induce proximity between POI and E3 ligase, facilitating ubiquitin transfer from E2 to POI and triggering proteasomal degradation.^3,4^ Dozens of PROTACs have advanced clinical trials, underscoring their significant therapeutic potential.^5^

Despite significant progress, the clinical translation of PROTACs still faces key challenges. Of the ∼600 E3 ubiquitin ligases encoded in the human genome, only a handful have been co-opted for PROTAC design.^6^ Among these, cereblon (CRBN)^2,7^ and the von Hippel-Lindau protein (VHL)^8^ are the most extensively utilized. Ligands for a few other ligases have been discovered, but remain largely unutilized in applications widely, including MDM2,^9^ IAPs,^10^ DCAF family,^11–15^ and others.^16,17^ However, CRBN and VHL are not universally expressed or functional across all tissues and disease contexts.^14,18^ CRBN ligands often trigger neo-substrate degradation (e.g., IKZF1, IKZF3, GSPT1, and SALL4), leading to potential off-target effects.^19,20^ The VHL may not be advantageous in certain contexts; for instance, it is observed that VHL is downregulated in pancreatic cancer.^21^ Moreover, both CRBN and VHL ligands typically feature large scaffolds, complicating optimization for improved pharmacokinetic and pharmacodynamic profiles.

To address these limitations, there is growing interest in expanding the E3 ligase toolbox to include novel, structurally tractable recruiters to treat malignant tumors.^22^ In 2023, James, Arrowsmith, and their co-workers reported an unusual class of PROTACs^23^ that utilize a simple primary amine with an aliphatic alkyl chain to promote the degradation of the nuclear receptor-binding SET domain-containing 2 (NSD2) protein. NSD2 functions as a methyltransferase responsible for the dimethylation of lysine 36 on histone H3 and is implicated in several malignancies, including pediatric acute lymphoblastic leukemia (ALL),^24^ pancreatic cancer, lung cancers, and multiple myeloma.^25^ Subsequently, the researchers advanced a second generation of NSD2 PROTACs by incorporating a slightly more rigid linker between the alkyl primary amine and NSD2 ligand.^26^ They discovered that the alkyl primary amine serves as a precursor to an aliphatic aldehyde, which covalently reacts with Cys326 of the F-box only protein 22 (FBXO22) E3 ligase. Multiple lines of evidence support the conversion of the amine into an unstable aliphatic aldehyde via amine oxidase activity in the media. Furthermore, they proposed that the previously reported primary amine-tethered PROTAC for degrading XIAP protein is also dependent on FBXO22.^27^ FBXO22 is a component of the SCF (Skp1–Cullin–F-box) E3 ligase complex and has been implicated in the degradation of multiple regulatory proteins, including p21,^28^ KLF4,^29^ p53,^30^ GAK,^31^ and VHL.^32^ In parallel, Winter^33^ and colleagues demonstrated that an alkylamine-tethered molecule, designated SPN3, exhibited a similar capacity to degrade FKBP12 through covalent modification of Cys326 within the C-terminal domain of FBXO22. Independently, Zhang and co-workers^34^ identified a class of PROTACs that utilizes a chloroacetamide electrophile to react with C227 and/or C228 in FBXO22, thereby enabling protein degradation. Subsequently, four studies,^35–38^ including our preprint,^38^ identified FBXO22-related degraders. The mechanisms of all these degraders are associated with the Cys 326 residue of FBXO22. Notably, Yauch and co-workers^35^ discovered compound G-6599, which contains an internal aryl alkyne and functions as a monovalent degrader for SMARCA2/4. Gray and co-workers^36^ reported newly identified degraders bearing a 2-pyridinecarboxyaldehyde moiety, that induce FBXO22-dependent degradation of BRD4 and CDK12. Brown and co-workers^37^ identified a degrader similar to ours that induces the degradation of NSD2 without requiring further biotransformation.

Here, we report directly active and stable aryl aldehyde-based ligands for FBXO22 that circumvents the need for in situ oxidation of a primary amine into an unstable aliphatic aldehyde, thereby enabling efficient degradation of NSD2 and CDK12. The aryl aldehyde moiety covalently engages Cys326 and demonstrates superior degradation efficiency compared to UNC8153, which employs a flexible hexylamine recruiter. Mechanistic studies confirm that degradation proceeds via the UPS pathways in a neddylation-dependent manner and does not require prior biotransformation of the ligand. In contrast to flexible alkyl linkers, our stable and more rigid aryl aldehyde-bearing scaffold likely enhances pharmacological properties, offering a robust new strategy for covalent E3 ligase recruitment in PROTAC design.

## Results and Discussion

### Design of Directly Active and Stable Electrophilic FBXO22-Recruiting NSD2 PROTACs

We were intrigued by the simple alkylamine-based PROTAC UNC8153 and its structure–activity relationship, which revealed an optimal spacing between a primary amine and an aryl group.^26^ We hypothesized that the amine undergoes oxidation to form an electrophilic aldehyde, enabling covalent reaction with cysteine residues on a novel E3 ligase and promoting the degradation of NSD2 through a mechanism involving metabolic activation. Inspired by the simplicity of the alkylamine-derived E3 ligase ligand, we sought to identify a stable and directly reactive electrophile capable of covalently engaging the same E3 ligase, thereby bypassing the need for enzymatic oxidation. To this end, we initiated a screening campaign to discover suitable electrophilic warheads (Figure 1). Our investigation began with a competition assay in HEK293T HiBiT-NSD2 reporter cell line developed in our lab, using UNC8153 as a reference degrader alongside a focused panel of electrophilic fragments. Loss of NSD2 degradation across competitor dose ranges served as a readout of binding competition by the electrophiles. Our goal was to identify a stable electrophilic warhead capable of effective engagement with this E3 ligase, paving the way for the development of novel, directly active E3 ligase ligands.

**Figure 1:**
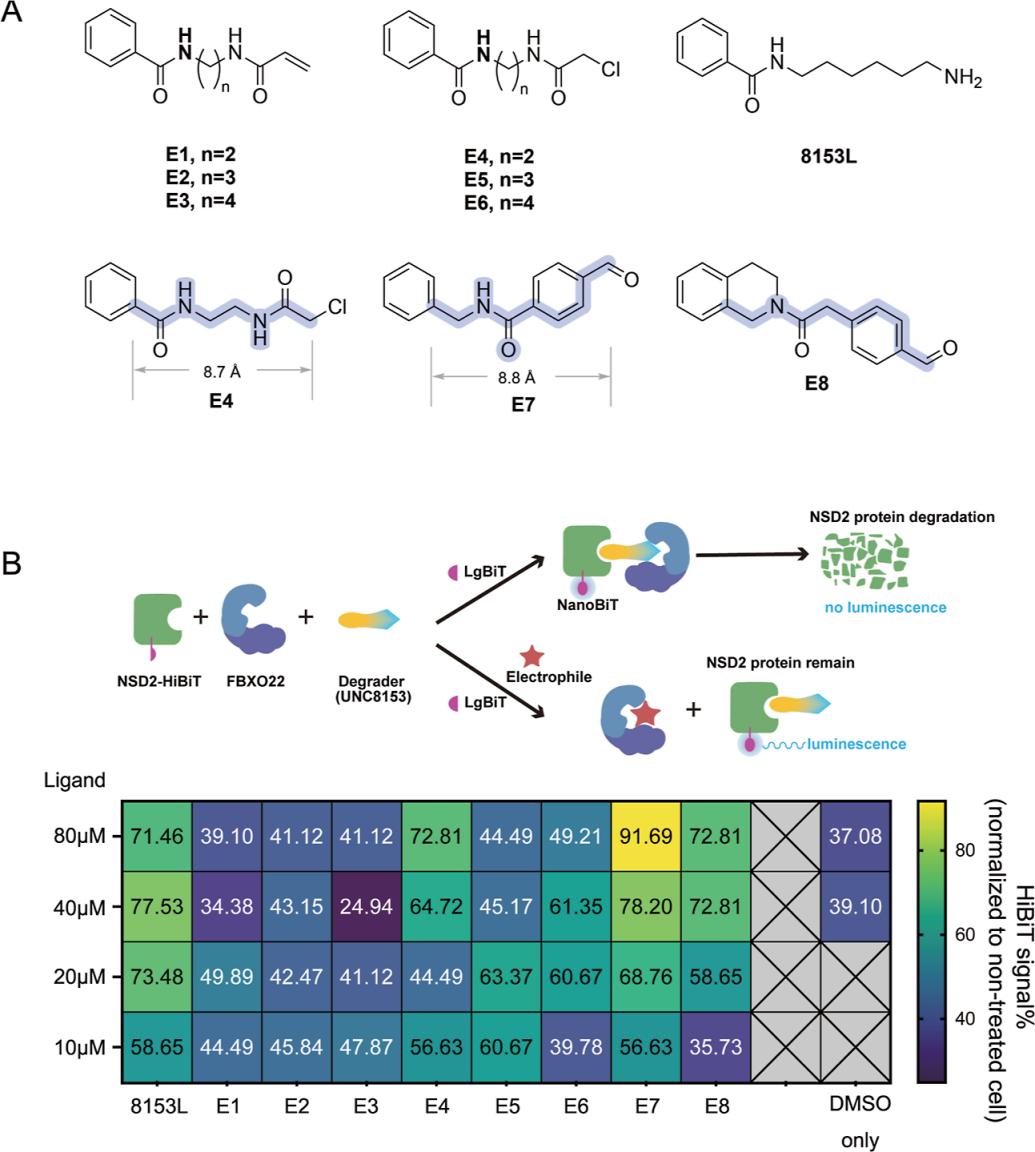
Competition assay using UNC8153 as an NSD2 degrader in the 293T-NSD2-HiBiT cell line. (A) Structures of electrophiles E1–E8 and 8153L, the fragment of UNC8153. (B) Schematic presentation of NSD2-HiBiT screening assay. Relative NSD2-HiBiT signal ratio normalized to nontreated DMSO control after 6 h degrader treatment.

We first evaluated two widely used covalent warhead classes — acrylamides and chloroacetamides — both commonly featured in covalent kinase inhibitors^39^ and PROTACs.^40^ In our competition assay, acrylamide-based fragments showed no significant activity against UNC8153. In contrast, the chloroacetamide fragment E4 displayed robust competition. Structurally, E4 comprises eight atoms with a molecular span of ∼8.7 Å between the phenyl carbon and the α-carbon of the carbonyl group, a geometry that supports effective competition. Recognizing the advantages of aryl aldehydes as reversible electrophiles with tunable reactivity, we next investigated them as alternative warheads. To mimic the spatial arrangement of E4, we designed compound E7, an aryl aldehyde with an ∼8.8 Å distance between the phenyl carbon and the aldehyde group, as determined by energy-minimized modeling. Competition experiments revealed that E7 displayed similar or better occupancy against UNC8153 compared with 8153L, the fragment derived from UNC8153, thereby validating aryl aldehyde as a promising alternative to chloroacetamide. During the course of our work, second generation of amine-based NSD2 PROTACs incorporating a slightly more rigid linker, featuring a tetrahydroisoquinoline ring between the alkyl primary amine and the NSD2 ligand, were reported, and FBXO22 was identified as the cognate E3 ligase.^26^ In this mechanism, cellular oxidases convert the alkylamine into an unstable aliphatic aldehyde, which covalently engages FBXO22 to drive targeted degradation. To further optimize the reactivity and mitigate potential off-target effects, we designed E8, a less reactive analog of E7. In E8, the electron-withdrawing para carbonyl group of E7 was replaced with a methylene group, and a tetrahydroisoquinoline ring was introduced to reduce conformational flexibility. E8 exhibited moderately reduced electrophilic reactivity compared to E7, yet retained binding characteristics comparable to E4 and 8153L. These results support E8 as a viable and potentially more selective FBXO22 ligand.

Based on the results of the competition assays, we next investigated whether PROTACs incorporating various aryl aldehyde-based electrophiles could effectively degrade NSD2. Using a HiBiT-based degradation assay in HEK293T-NSD2-HiBiT cells, the E7-derived PROTAC T1 achieved a notable *D*_max_ of 70% at 1 μM, confirming its strong potential as an E3 ligase recruiter. To further refine this scaffold, we explored a series of linker modifications (Table 1). Extending the linker by one or two methylene units (compounds T2, T3, and T4) reduced activity, with degradation observed only at higher concentrations (10 μM) and absent at 1 μM.

**Table 1:**
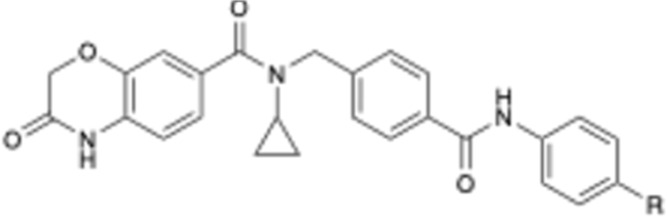

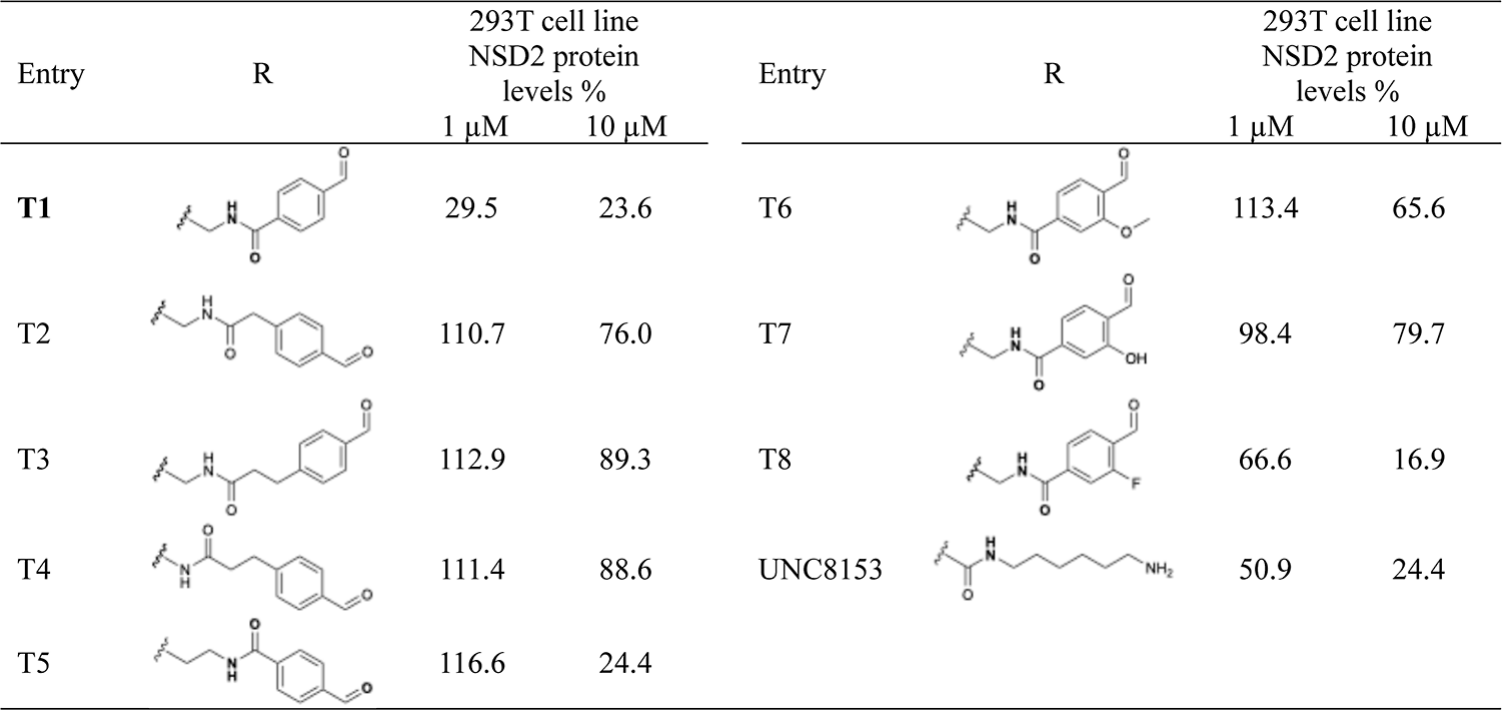
NSD2 Degradation Result of E7-Derived PROTAC T1 and Its Analogs Using an NSD2 HIBIT Assay in 293T Cell Line

We next evaluated the effect of aldehyde orientation. Relocating the amide linkage of T4 to the para-position of the phenyl aldehyde yielded compound T5. Although T5 retained degradation activity comparable to T1 at 10 μM, it lost activity at 1 μM, indicating that increased molecular flexibility compromises target engagement. We also attempted to tune the electron density of the aldehyde moiety to reduce potential off-target reactivity. Introduction of a methoxy group at the ortho-position (compound T6) abolished activity. Similarly, the *ortho*-hydroxy (T7) and electro-deficient *ortho*-fluoro analog (T8) failed to induce degradation, suggesting that modifications at this position interfere with covalent engagement, possibly through steric hindrance effects.

As soon as the second generation amine-based NSD2 PROTACs incorporating the tetrahydroisoquinoline were reported as improved ligands for FBXO22 E3 ubiquitin ligase,^26^ we turned our attention to E8-derived PROTACs, which feature the same tetrahydroisoquinoline linker and a phenyl aldehyde lacking a para-carbonyl group (Table 2). Compound T9 achieved a *D*_max_ of ∼65% at 1 μM, only slightly weaker than T1. Shifting the aldehyde from the para-to the meta-position (compound T10) reduced the degradation by about 50%. Complete removal of the carbonyl linkage (compounds T11 and T12) abolished degradation, highlighting the importance of both aldehyde orientation and structural rigidity. Linker elongation experiments further confirmed this trend. Insertion of a methylene group (compound T13) decreased degradation to ∼54% of *D*_max_ at 10 μM, while oxygen insertion (compound T14) eliminated activity entirely, even after positional adjustments (compounds T15 and T16). Further elongation (compound T17) was similarly ineffective. These results suggest that excessive linker flexibility disrupts proper alignment of the aldehyde with FBXO22, thereby reducing covalent capture efficiency.

**Table 2:**
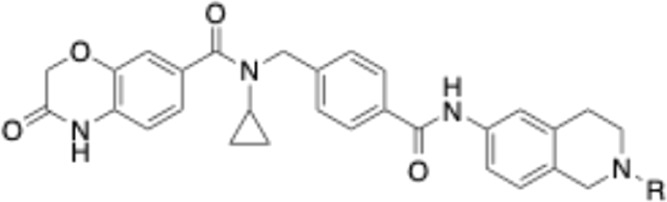

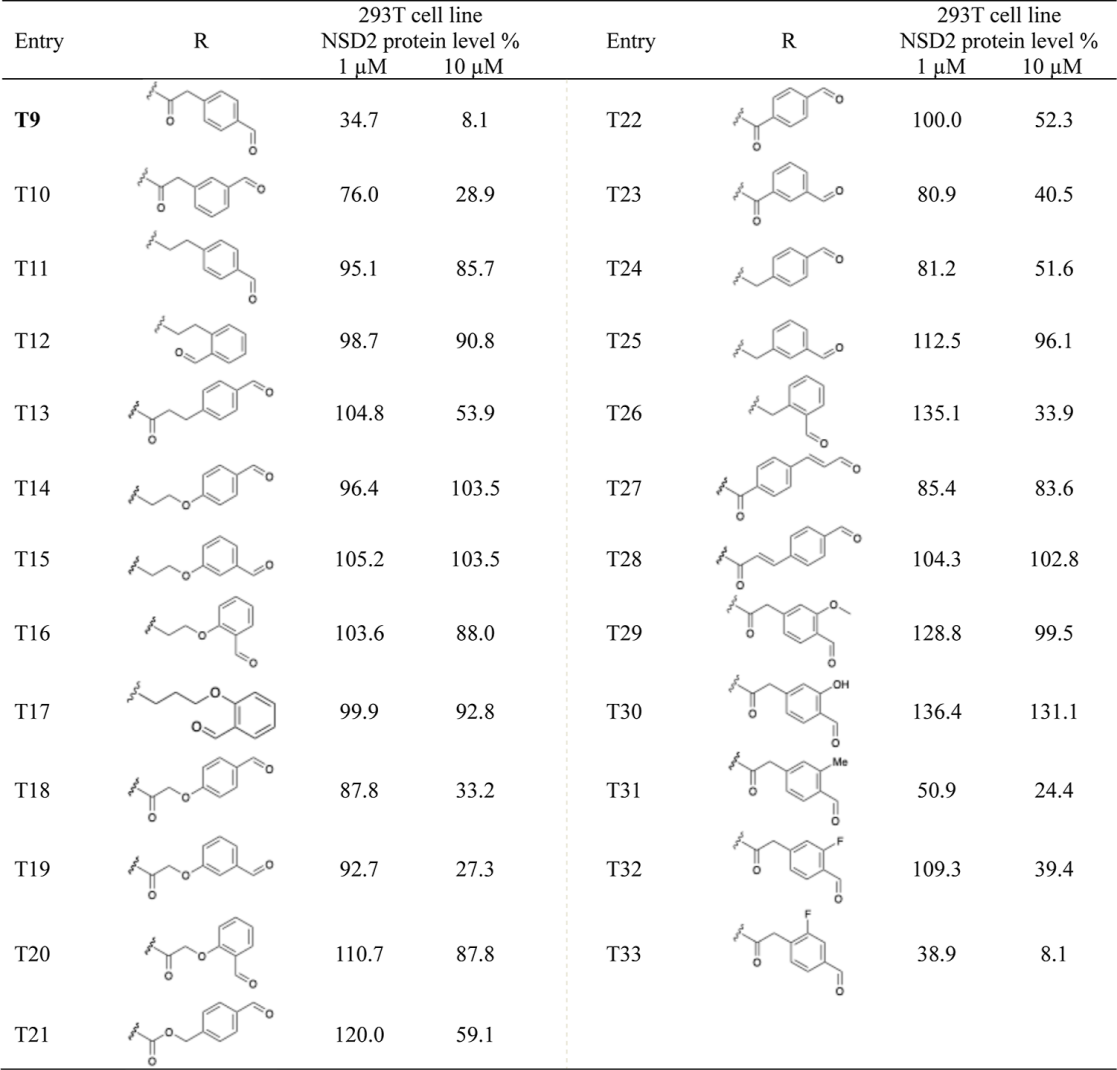
Degradation Result of E8-Derived PROTAC T9 and Its Analogs Using an NSD2 HIBIT Assay in 293T Cell Line

To address this limitation, we reduced linker flexibility by replacing methylene groups with carbonyls, generating compounds T18, T19, and T20. These analogs displayed significantly improved activity compared to T14, supporting the hypothesis that rigidifying the linker stabilizes a productive ternary complex formation. We also evaluated carbamate substitutions (compounds T21), motivated by their favorable chemical and proteolytic stability in drug design.^41^ However, these analogs showed no appreciable improvement relative to their amide counterparts. Together, these results demonstrate that elongation or enhanced flexibility consistently diminishes activity, while rigidifying elements promote efficient degradation.

Conversely, shortening the linker by one methylene unit also reduced activity: both T22 (para) and T23 (meta) showed weaker degradation than T9. Substitution of the para-acetyl group of the phenylaldehyde with methylene (compounds T24, T25, and T26) or replacement with a Michael acceptor electrophile in T27 led to only marginal degradation. Interestingly, the bielectrophilic design (T28) also failed to outperform T22. Collectively, these observations indicate that the aldehyde’s distance from the NSD2 ligand is finely tuned, and that both shortening and elongation compromise activity.

Finally, we explored substitution at the aldehyde’s ortho and meta-positions to modulate electronic effects. Ortho-substituents (−OMe, −OH, −Me, −F) consistently abolished activity, likely due to steric interference at the protein–ligand interface. In contrast, a *meta*-fluoro derivative (T33) retained activity comparable to T9, suggesting that electron-withdrawing substituents at the meta-position may preserve reactivity without introducing steric penalties. These findings highlight the stringent structural constraints governing FBXO22 recruitment and suggest that steric hindrance adjacent to the aldehyde must be avoided, while meta-substitution with electron-withdrawing groups offers a viable path for further optimization. Additionally, T9 incorporates a phenyl aldehyde, which is bulkier than linear electrophiles such as alkyl aldehydes or amines; this increased steric bulk may help reduce off-target engagement of other cysteine-containing protein pockets.

### T1 and T9 Display the Degradation Ability in Multiple Cell Lines

Based on preliminary screening results from the NSD2-HiBiT assay, T1 and T9 (Figure 2A) emerged as our most potent NSD2 degraders, exhibiting DC_50_ values of 1.3 μM and 0.37 μM, respectively (Figure 2B). As previous studies reported, high NSD2 expression has been correlated to poor clinical outcomes for patients with nonsmall cell lung cancer (NSCLC), multiple myeloma (MM), and prostate cancer (PC).^42–44^ We further characterized our compounds in three related cell lines. In the H358 and RPMI8226 cells, treatment with T1 and T9 across a range of concentrations (0.1–10 μM) for 4 h resulted in significant, dose-dependent degradation of both the long and short isoforms of NSD2, as confirmed by Western blotting analysis (Figures 2C and S1A). Notably, both compounds achieved over 50% degradation of total NSD2 at 1 μM, consistent with their performance in the NSD2-HiBiT assay. However, in H358 and RPMI8226 cells treated at 2 μM, T9 showed slightly reduced potency relative to T1.

**Figure 2:**
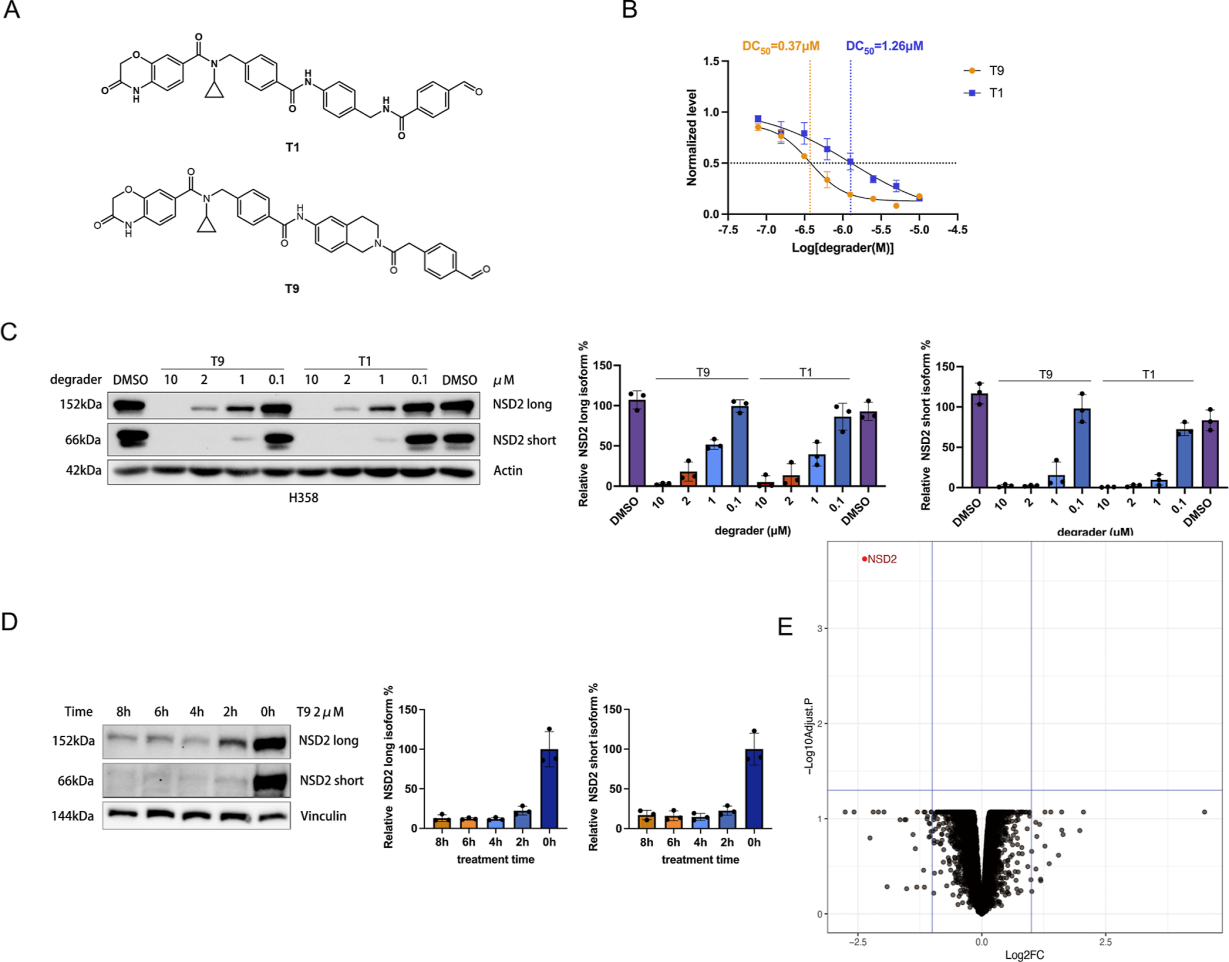
T1 and T9 display the NSD2 degradation activity in multiple cell lines in a dose- and time-dependent manner. (A) The structure of NSD2 degraders T1 and T9. (B) Degradation of NSD2 in HEK293T-NSD2-HiBiT cells after 4 h T1/T9 treatment displayed as normalized relative change between the treated groups vs the control group. DC_50_ = 0.37 μM for T9 and DC_50_ = 1.26 μM for T1. (C) Immunoblotting analysis of NSD2 in H358 treated with various concentrations of T1 and T9 for 4 h. The graph represents quantification of the long and short NSD2 isoforms/Actin protein content (*n* = 3 biological independent samples). (D) Immunoblotting analysis of NSD2 in H358 cells treated with 2 μM of T9 degrader for 0, 2, 4, 6, 8 h time points. The graph represents quantification of the long and short NSD2 isoforms/Vinculin protein content (*n* = 3 biological independent samples). (E) Global proteomics analysis of T9 in H358 cells (*N* = 3) after 4 h treatment of DMSO or 4 μM T9. *P*-value was adjusted by Benjamini-Hochberg method to restrain the false discovery rate (FDR).

In contrast, results in the PC3 prostate cancer cell line revealed opposite trend: T9 induced significant NSD2 degradation at 10 μM, whereas T1 showed no apparent effect at the same concentration (Figure S1B). These findings suggest cell line–dependent degradation efficacy. To further characterize the kinetics of degradation, we performed a time-course analysis using T9 in H358 cells (Figure 2D). Cells treated with 2 μM T9 showed substantial NSD2 degradation within 2 h, with around 70% of protein eliminated, and maximal reduction achieved by 4 h. T1 displayed a similar time-dependent degradation profile (Figure S1C), indicating that both degraders act rapidly to induce FBXO22-mediated NSD2 elimination. Based on the activity of T9 across various cell lines, it was observed that degradation is more prominent in the H358 cell line. We hypothesize that the expression of ligase (FBXO22) serves as the primary driving factor. Comparative analysis of FBXO22 protein levels across different cell lines indicates that FBXO22 levels are significantly higher in the H358 cell line compared to the PC3 and RPMI8226 cell lines (Figure S2B), which correlates with the observed efficacy at 2 μM. Consequently, the cellular level of FBXO22 appears to be a critical limiting factor for T9-induced turnover.

### PWWP1 Domain Engagement Is Required for T9-Mediated NSD2 Degradation

To confirm that the degradation of NSD2 is dependent on PWWP1 domain engagement, we evaluated the activity of T9 across various NSD2 isoforms with distinct domain architectures. Treatment with T9 resulted in the robust degradation of both NSD2-Long and NSD2-Short isoforms, both of which contain the PWWP1 domain—across multiple cell lines, including H358, RPMI8226, and PC3 (Figure S2A). Critically, we observed that the RE-IIBP isoform, which lacks the PWWP1-targeted recruitment site, was completely resistant to T9-induced degradation. These findings demonstrate that PWWP1 engagement is required for PROTAC-mediated recruitment and subsequent proteasomal degradation.

### T9 Induces the Degradation Selectively

To evaluate proteome-wide selectivity, we performed global proteomic profiling in the H358 cell line treated with 4 μM degrader T9 for 4 h. Despite the inherent electrophilicity of the aldehyde moiety—which might be expected to promote off-target reactivity with cellular nucleophiles—the degrader exhibited an exquisite selectivity profile, with NSD2 identified as the sole protein significantly depleted across the global proteome (Figure 2E). These data confirm that T9 is a highly specific chemical tool for the selective degradation of endogenous NSD2.

### T9-Induced NSD2 Protein Degradation Is Proteosome and Neddylation-Dependent

Since T9 exhibited activity across a broader range of cell lines, we selected it for mechanistic studies. To elucidate the mechanism of T9-induced NSD2 degradation, H358 cells were cotreated with the proteasome inhibitor MG132, which completely abrogated NSD2 degradation (Figure 3A), confirming that T9 acts through the ubiquitin–proteasome system (UPS). To further define the involvement of specific E3 ligase machinery, we treated cells with MLN4924, a selective inhibitor of the NEDD8-activating enzyme, which is required for activation of Cullin–RING ligases (CRLs). At 1 μM, MLN4924 also fully blocked T9-induced NSD2 degradation, indicating that the degradation process is dependent on a Cullin–RING E3 ligase complex. Together, these results strongly support that T9 mediates NSD2 degradation through a canonical CRL-dependent UPS pathway.

**Figure 3:**
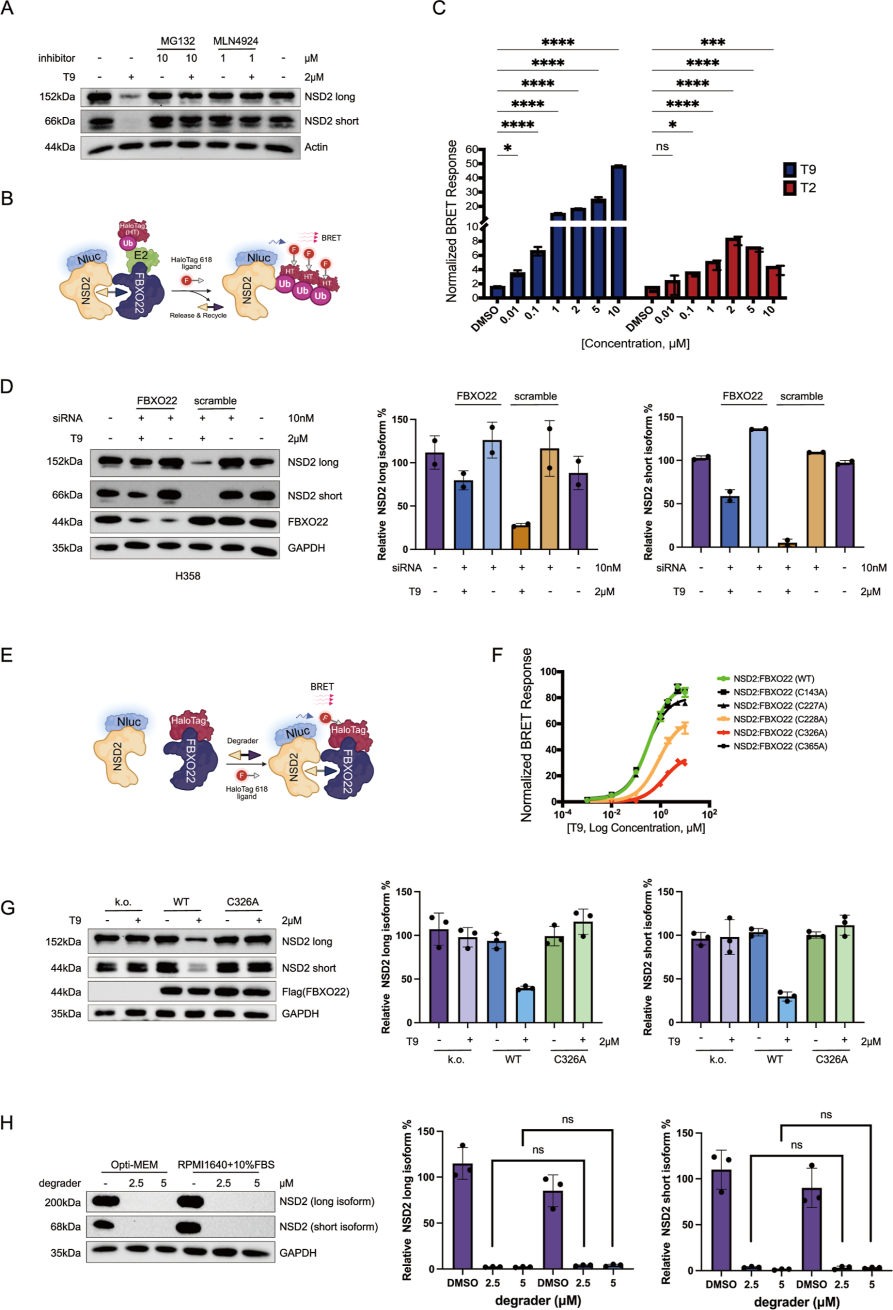
Mechanistic study of T9-induced NSD2 degradation. (A) Immunoblot of NSD2-long and NSD2-short upon T9 treatment for 4 h in the presence or absence of the MG132 proteasome inhibitor and NEDD8 inhibitor–MLN4924 in H358 cells. (B) Schematic of NanoBRET mechanism measuring NSD2-ubiquitin protein interaction.^45^ (C) NanoBRET analysis of NSD2 ubiquitination in 293T cells treated with varying concentrations of T2 and T9 (*n* = 3 biological independent samples, statistical significance was determined by two-way ANOVA; ****p < 0.0001; ns = not significant). (D) Knockdown of FBXO22 by siRNA in H358 cells. T9 was added 48 h postknockdown. The graph represents quantification of the long and short NSD2 isoforms/GAPDH protein content (*n* = 2 biological independent samples). (E) Schematic of NanoBRET mechanism measuring NSD2-FBXO22 protein–protein interaction.^46^ (F) NanoBRET analysis of the interaction between NSD2 and FBXO22 cysteine mutants in 293T cells treated with varying concentrations of T9. (*n* = 3 biological independent samples, statistical significance was determined by two-way ANOVA; ****p < 0.0001; ns = not significant). (G) Immunoblot of transfected 293T cells upon different FBXO22 mutation type. FBXO22 k.o. 293T cells were transiently transfected with empty vector (k.o.), wild-type HA-FBXO22 (WT), or mutant C326A HA-FBXO22 (C326A). The graph represents quantification of the long and short NSD2 isoforms/GAPDH protein content­(*n* = 3 biological independent samples). (H) Immunoblot of NSD2 long and short isoform degradation in solution Opti-MEM and RPMI1640 supplemented with 10%FBS. The graph represents quantification of the long and short NSD2 isoforms/GAPDH protein content (*n* = 3 biological independent samples; ns = nonsignificant).

### Degradation Induced by T9 Involves FBXO22 and Is Independent of Oxidase

To confirm that NSD2 degradation induced by compound T9 is mediated by the E3 ligase FBXO22, we performed RNA interference to knock-down FBXO22 expression (Figure 3D). Using a scrambled siRNA as a negative control, we observed a partial rescue of NSD2 protein levels upon FBXO22 knockdown, consistent with reduced availability of the E3 ligase. This result supports the role of FBXO22 in mediating T9-induced degradation. A parallel experiment in PC3 cells yielded similar results, further validating FBXO22 as the responsible ligase across multiple cell types (Figure S1D).

Beyond this FBXO22-dependent mechanism, previously reported FBXO22 PROTACs bearing amine-based moieties require amine oxidase activity in the medium.^27,33^ In contrast, our aldehyde degrader T9 maintains comparable degradation efficiency in both complete media containing amine oxidase and oxidase-free media (Figure 3H). These results demonstrated that T9 engages FBXO22 directly without the need for metabolic activation. Our data highlight a key advantage of aldehyde-based recruiters like T9: they enable immediate, enzyme-independent covalent binding to FBXO22, improving both mechanistic clarity and translational tractability.

### T9 Facilitates Ternary Complex Formation with NSD2 and FBXO22

To further elucidate the mechanism of action of compound T9, we performed an in vitro coimmunoprecipitation (Co-IP) assay to assess the formation of a ternary complex involving T9, NSD2, and FBXO22. In this experiment, engineered 293T cells, which stably express WT HA-FBXO22, were treated with T9 in the presence of MLN4924, a neddylation inhibitor that stabilizes E3 ligase–substrate interactions by preventing downstream ubiquitination and degradation. As shown in Figure S3B, HA-FBXO22 coimmunoprecipitated with endogenous NSD2 only in the presence of T9 and MLN4924, confirming the formation of a stable ternary complex. These findings support the proposed PROTAC mechanism of T9, whereby covalent engagement of FBXO22 facilitates proximity-induced degradation of NSD2.

We then utilized a more quantitative NanoBRET assay to elucidate the mechanistic factors underlying FBXO22-mediated NSD2 degradation. We quantified compound-induced NSD2-FBXO22 ternary complex formation (TCF, Figures S3A and `[Fig fig3]E), substrate ubiquitination (Figure 3B,C), and proteasome recruitment (Figure S2C,S2D) within live cells. Both T9 and T2 promoted dose-dependent NSD2-FBXO22 TCF, with T9 exhibiting substantially greater potency and maximal BRET amplitude relative to T2 (Figure S3A), indicating superior stabilization of the productive complex. The results suggest that rigidified linkers likely stabilize productive ternary complex formation. Additionally, in the presence of WT FBXO22, both T9 and T2 induced dose-dependent NSD2 polyubiquitination and proteasome recruitment, with T9 displaying greater potency than T2 in promoting these downstream events. Together, these data confirm the mechanism of action of T9 and T2, demonstrating that NSD2 degradation proceeds through compound-induced NSD2-FBXO22 ternary complex formation, ubiquitin transfer, and proteasome engagement, rather than by indirect or off-target effect.

### Cysteine 326 of FBXO22 Mediates the T9-Induced NSD2 Degradation

Previous studies have implicated several cysteine residues within the FIST_C-terminal region of FBXO22 as potential covalent engagement sites for electrophilic degraders.^26,33,34^ To determine the specific cysteine residue involved in T9-mediated degradation, we first focused on C227 and C228, which have been proposed as reactive sites in other FBXO22-directed PROTACs^34^ (Figure S3D). Western blot analysis revealed that mutation of C227/228 did not impair T9-induced NSD2 degradation, indicating that these residues are not essential for covalent engagement in our system, despite the presence of an aldehyde warhead. We then turned our attention to C326, a residue previously shown to be critical for FBXO22 reactivity in the context of alkylamine-derived degraders.^26,33^ Using a similar approach, we generated plasmid expressing HA-FBXO22 with a C326A mutation and treated transfected cells with 2 μM of T9. Compared to wild-type and FBXO22-knockout cells, the C326A mutant fully abrogated NSD2 degradation, while wild-type cells retained full activity (Figure 3G). These results unambiguously support that Cys326 is essential for T9-induced degradation, whereas Cys227/Cys228 are nonessential.

Additionally, NanoBRET assays were performed to evaluate the impact of specific cysteine mutations on the formation of the NSD2–FBXO22 ternary complex (Figures 3F and S3C). Mutational analysis revealed that FBXO22 cysteine residues contribute differently to downstream degradation efficiency. Consistent with our degradation data, the C228A substitution modestly reduced ternary complex formation (TCF) amplitude but did not significantly impair NSD2 degradation, suggesting that partial attenuation of TCF stability remains compatible with effective substrate turnover. In contrast, the C326 mutation markedly impaired NSD2 degradation and resulted in a substantially weaker TCF signal, indicating that this residue is critical for complex stability.

Thus, in our system, covalent engagement of FBXO22 by T9 occurs specifically through C326, validating this residue as the critical nucleophilic site for aryl aldehyde-based FBXO22 recruiters.

### Functional Phenotypic Consequences of NSD2 Degradation

To evaluate whether the targeted degradation of NSD2 translates into meaningful biological outcomes, we assessed the impact of T9 on cancer cell fitness and motility. Given the established role of NSD2 in driving oncogenic signaling and proliferation in various malignancies,^47^ we first examined the antiproliferative effects of T9 in H358 and PC3 cells. As shown in Figure 4A, treatment with T9 induced a significant, dose-dependent inhibition of cell growth over a 4 day period. The observed GI_50_ values correlate with the concentrations required for near-complete NSD2 turnover, providing a direct link between the chemical event of degradation and a global reduction in cellular fitness. To further demonstrate the advantages of the benzaldehyde warhead in T9, we compared its antiproliferative activity with that of its primary amine-based progenitor, UNC8732, in PC3 and H358 cancer cell lines. As illustrated in Figure S3F, T9 demonstrated markedly enhanced antiproliferative efficacy compared with UNC8732 in both cell lines. At the highest dose, T9 robustly suppressed PC3 cell growth to roughly 45%, whereas UNC8732 exhibited a much weaker effect. Notably, in the H358 cell line, UNC8732 showed a complete lack of antiproliferative activity across all concentrations, while T9 maintained dose-dependent growth inhibitory effects. These findings collectively demonstrate that bypassing the requirement for intracellular metabolic activation via direct preinstallation of the benzaldehyde motif provides a significant functional advantage, translating into more potent cellular efficacy. Beyond proliferation, NSD2 is a known driver of the Epithelial–Mesenchymal Transition (EMT) and metastatic potential.^48^ To determine whether our degrader disrupts these functional outputs, we performed a wound healing assay in PC3 cells. Treatment with T9 dramatically suppressed the migratory capacity of the cells compared to the DMSO control (Figure 4B). At concentrations of 5–10 μM, wound closure was effectively halted over 24 h. These results demonstrate that the removal of the NSD2 protein via FBXO22-mediated degradation results in a robust suppression of the NSD2-driven migratory program, reinforcing the therapeutic relevance of this strategy.

**Figure 4:**
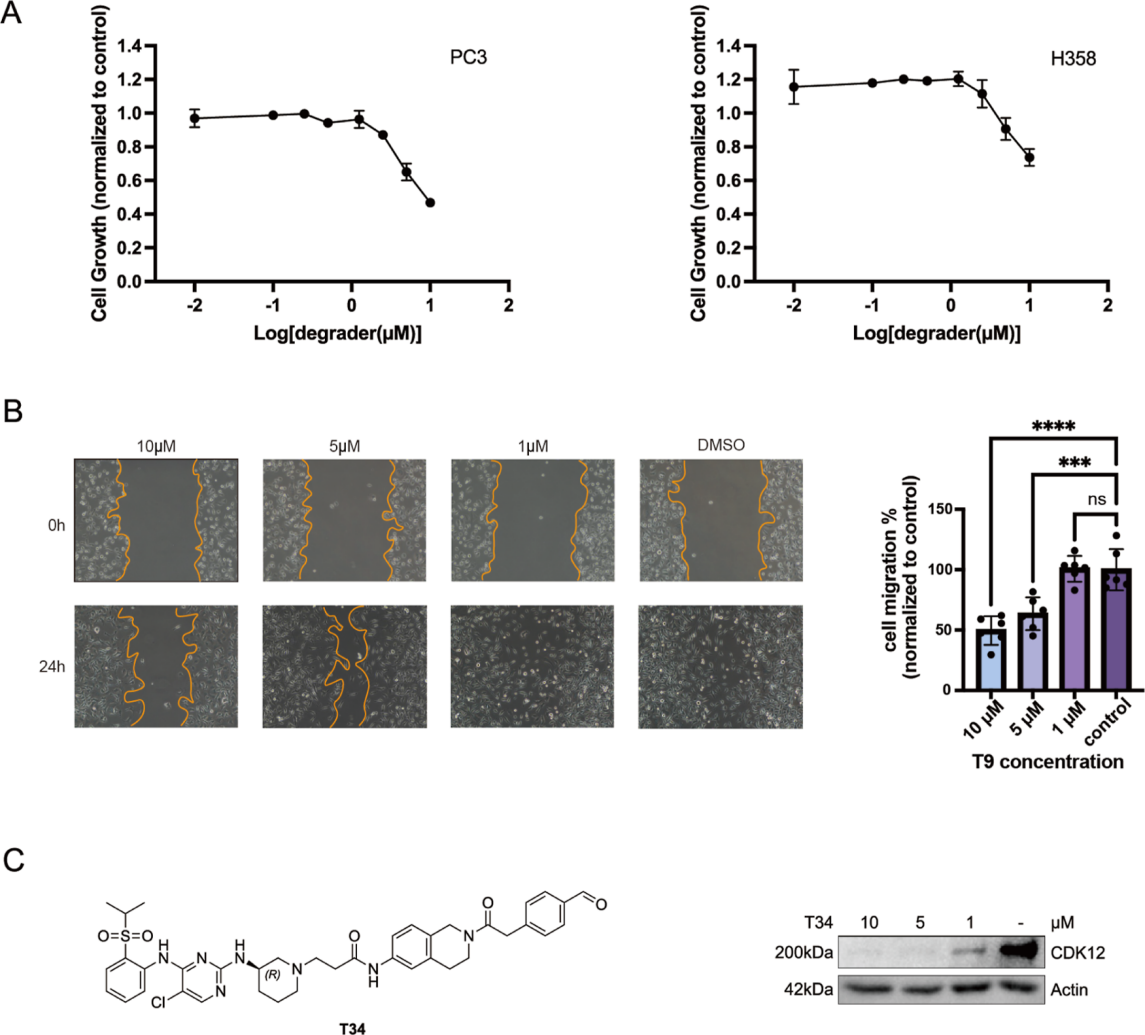
Effect of T9 in cancer cell proliferation and migration, and the development of a CDK12-targeting degrader by recruiting FBXO22. (A) 4 day antiproliferation effect in PC3 (left) and H358 (right) upon 10 μM T9 treatment. (*n* = 3 biological independent samples) (B) Antimigration effect of T9 on PC3 cells at 24 h. Representative images from three independent experiments are shown. Orange lines delineate the boundaries; the absence of lines at 24 h for the 1 μM and DMSO groups indicates complete wound closure. The graph represents the quantification of migration based on the ratio of cell coverage in the treated group versus the control group (*n* = 6, 2 replicates from 3 biologically independent samples; ns = nonsignificant; ***p < 0.001; and ****p < 0.0001). (C) Structure of CDK12 targeting degrader T34. Immunoblotting analysis of CDK12 in Jurkat cells treated with various concentrations of T34 for 24 h.

### Broadening the Scope of FBXO22-Recruiting PROTACs

A critical question in the development of new E3 ligase recruiters is their modularity across diverse biological targets. To demonstrate that our aryl aldehyde-based recruitment of FBXO22 is a generalizable platform rather than a target-specific solution for NSD2, we designed and synthesized compound T34, a bifunctional degrader targeting CDK12 (Figure 4C). T34 was constructed by conjugating a validated CDK12-binding scaffold to our lead FBXO22-recruiting warhead. In Jurkat cells, T34 induced robust, dose-dependent degradation of CDK12 within 24 h, with the onset of degradation observed at concentrations as low as 1 μM. The successful transition from a histone methyltransferase (NSD2) to a transcriptional kinase (CDK12) confirms that our FBXO22-targeting ligands are highly modular. This target-agnostic behavior establishes the aryl aldehyde moiety as a versatile addition to the TPD toolkit.

## Conclusion

In this study, we report the development of novel aryl aldehyde-based PROTACs that recruit FBXO22 to induce targeted degradation of NSD2. Through a competition assay with diverse electrophilic warheads, we identified phenyl aldehyde as the optimal electrophilic handle, enabling robust NSD2 degradation activity in HEK293T cells. Systematic structure–activity optimization revealed the strict structural constrains required for effective FBXO22 recruitment. Small changes in linker length, aldehyde orientation, linker flexibility, and steric environment profoundly affected degradation outcomes. In particular, minor modifications proximal to the aldehyde, particularly those introducing steric bulk, were strongly unfavorable, whereas increasing electron deficiency at the meta-position is tolerated. These findings underscore the delicate balance governing covalent engagement of FBXO22 and provide guiding principle for future ligand design.

Beyond HEK293T cells, compound T9 demonstrated consistent NSD2 degradation activity across additional cancer cell lines, including H358, RPMI 8266, and PC3. Mechanistic interrogation using RNA interference and chemical inhibitors of the proteasome and neddylation enzyme confirmed that T9-induced NSD2 degradation is proteasome- and neddylation-dependent and requires functional FBXO22. Furthermore, ternary complex formation via NanoBRET assay and Co-immunoprecipitation experiments further demonstrated the formation of a ternary complex between T9, NSD2, and FBXO22, validating its PROTAC-like mechanism of action. Importantly, targeted mutagenesis pinpointed cysteine 326 (C326) in the C-terminal domain of FBXO22 as the essential nucleophilic residue for T9-mediated NSD2 degradation.

Our lead compound, T9, induces potent and selective degradation of the oncogenic histone methyltransferase NSD2, translating directly into significant functional outcomes, including the robust inhibition of cancer cell proliferation and migration. Furthermore, the successful design and validation of the CDK12-targeting degrader T34 provides direct evidence that our FBXO22 recruitment strategy is a modular and target-independent E3-ligase recruitment platform. This expands the TPD toolkit beyond conventional ligases, offering a versatile alternative for the development of PROTAC degraders against a range of therapeutically relevant proteins.

Altogether, these findings establish the feasibility of covalent FBXO22 recruitment via stable aryl aldehyde-based ligands, providing both a new chemical modality for FBXO22 engagement and a foundation for the design of next-generation covalent PROTACs. This work expands the toolbox of E3 ligase ligands and opens avenues for developing novel therapeutics targeting NSD2 and potentially other disease-relevant substrates through covalent FBXO22 recruitment.

## Synthesis

UNC8732, NSD2 intermediate (SM-1), and 8153L were prepared according to previously published procedures,^23,26,49^ and their respective spectroscopic signatures (^1^H NMR and LC–MS) were found to be consistent with the values reported therein. The synthetic route used for preparing key intermediates is shown in Scheme 1. The synthesis of electrophiles and all the PROTACs is summarized in Scheme 2. E1–E6 were synthesized via amide coupling, Boc deprotection, and subsequent amide condensation. E7 and E8 simply underwent a single amide condensation. The PROTACs are divided into three groups: T1–T10, T13, T18–T20, T21–T23, and T27–T33, which were obtained through amide coupling with the corresponding amines and acids. T11–T12, T14–T17, and T24–T26 underwent substitution reactions between amines and bromides. T21 is a carbamate, formed through a single carbamate formation and oxidation from alcohol to aldehyde. The synthetic route for the CDK12 degrader T34 is shown in Scheme 3.

**Scheme 1:**
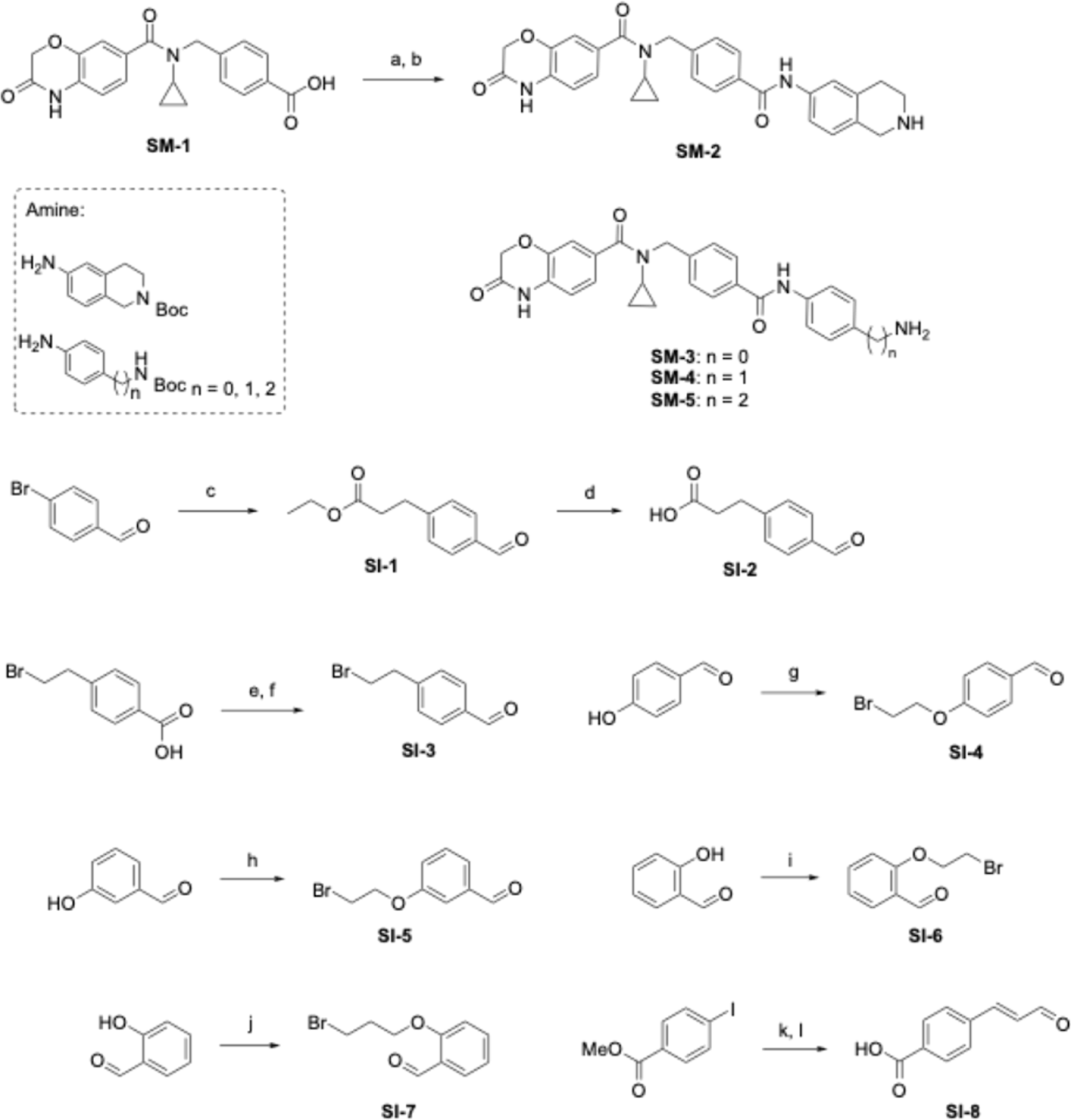
Preparation of Key Intermediates for the Synthesis of PROTACs^a^

a Reagent and conditions: (a) Amine, TCFH, NMI, DMF, rt; (b) HCl in dioxane/DCM, rt; (c) Acrolein diethyl acetal, Pd­(OAc)­2, TEA, TBACl, DMF, 90 °C; (d) LiOH·H2O, MeOH/H2O, rt; (e) BH3·THF, THF, 0 °C – rt; (f) MnO2, Chloroform, 70 °C; (g) 1,4- dibromoethane, K2CO3, DMF, rt; (h) 1,2-dibromoethane, Cs2CO3, DMSO, 60 °C; (i) 1,2-dibromoethane, Cs2CO3, DMF, 80 °C; (j) 1,3-dibromopropane, Cs2CO3, DMF, 80 °C; (k) Acrolein diethyl acetal, Pd­(OAc)­2, (*n*-Bu4N)­OAc, KCl, K2CO3, DMF, 90 °C; (l) LiOH, THF/H2O, rt.

**Scheme 2:**
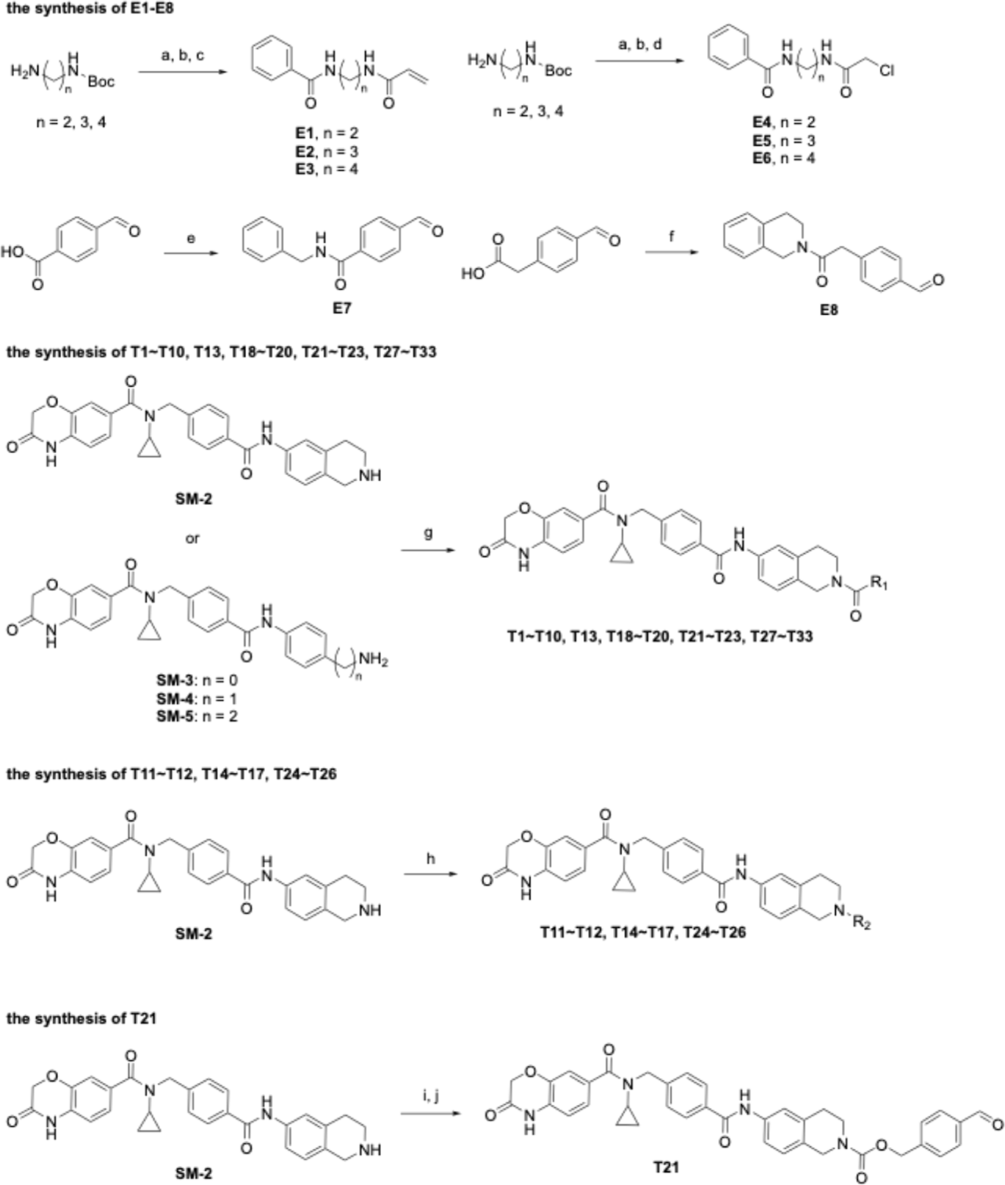
Synthesis of Electrophile E1–E8 and Compound T1–T33^a^

a Reagent and conditions: (a) benzoyl chloride, TEA, DCM, DMAP, 0 °C; (b) HCl in dioxane, DCM, rt, (c) acrylic acid, HATU, DIPEA, DMF, rt; (d) Chloroacetyl chloride, TEA, DCM, 0 °C; (e) phenylmethanamine, EDCI, DMAP, ACN, rt; (f) 1,2,3,4-tetrahydroisoquinoline, EDCI, HOBT, DMAP, DMF, rt; (g) Acid, EDCI, HOBt, DMAP, DMF, rt; (h) Bromide, NaHCO_3_, DMF, rt; (i) benzene dimethanol, CDI, TEA, NaH, DMF, rt; (j) DMP, DMF, rt.

**Scheme 3:**
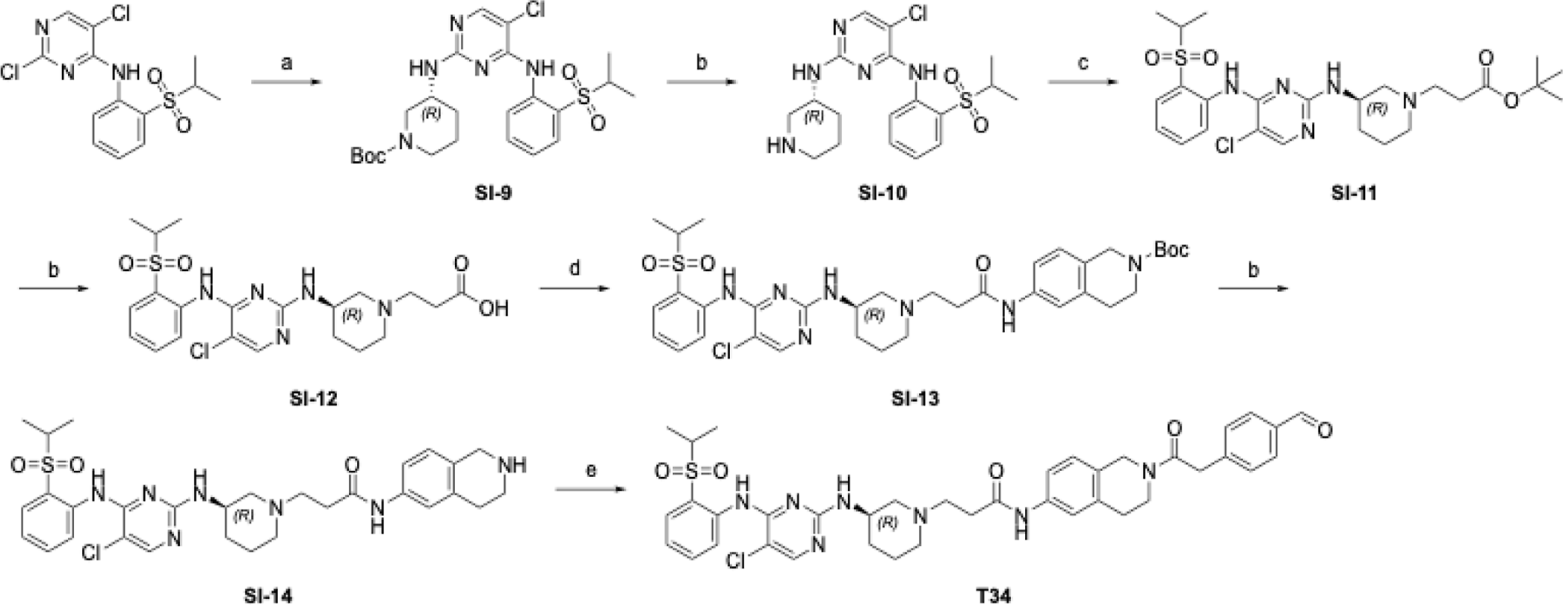
Synthesis of Compound T34^a^

a Reagent and conditions: (a) *tert*-butyl (*R*)-3-aminopiperidine-1-carboxylate, DIPEA, NMP, 0–110 °C; (b) TFA, DCM, rt, (c) *tert*-butyl 3-bromopropanoate, K_2_CO_3_, MeCN, rt; (d) *tert*-butyl 6-amino-3,4-dihydroisoquinoline-2­(1*H*)-carboxylate, HATU, DIPEA, DMF, rt; (e) 2-(4-formylphenyl)­acetic acid, EDCI, DMAP, DMF, rt.

## Experimental Section

### Chemistry

Reactions were carried out in oven-dried glassware prior to use. All reagents and solvents were used as received unless otherwise stated. Any anhydrous solvents used were purchased as “anhydrous” grade and used without further drying. “Room” or ambient temperature varied between 20 and 25 °C. Analytical thin-layer chromatography (TLC) was performed using aluminum plates precoated with silica gel impregnated with a fluorescent indicator (254 nm). TLC plates were visualized by illumination with a 254 nm UV lamp. Analytical LC–MS data for all compounds were acquired using an Agilent 1290 Infinity II system with the UV detector set to 254, 210, and 280 nm. Samples were automatically injected (3 μL) onto an Agilent ZORBAX 300SB-C18, 4.6 × 50 mm, 3.5 μM column at 25 °C. Mass spectra (MS) data were acquired in positive ion mode using an Agilent InfinityLab LC/MSD 6120 quadrupole mass spectrometer with an electrospray ionization (ESI) source. Normal-phase flash column chromatography was performed using a Teledyne Isco CombiFlash Nexgen 300+ with RediSep Rf silica columns, and The UV detector was set to 254 and 280 nm. Preparative HPLC was performed using a Shimadzu Nexera Prep system with the UV detector set at 210 and 254 nm. Samples were injected onto a Shimadzu shim-pack GIS 250 × 20 mm (5 μm) C18 column at room temperature. Mobile phases A (H_2_O + 0.1% Formic acid) and B (MeCN +0.1% Formic acid) were used with a flow rate of 20 mL/min. The liquid chromatography (LC) analysis of the final products was performed on a Shimadzu Nexera XR High-pressure gradient system using a Shimadzu Nexcol C18 column (50 × 3.0 mm, 5 μm); Short-time method: The mobile phases were mobile phase A (MiniQ water, with 0.1% formic acid) and mobile phase B (HPLC grade of acetonitrile, with 0.1% formic acid). The gradient was increased from 5% to 95% at 10 min, then held at isocratic 95% B for 5 min, and then immediately stepped back down to 5% for 5 min of re-equilibration. The flow rate was set at 0.8 mL/min. The column temperature was set at 30 °C. Long-time method: The mobile phases, setting temperature, and flow rate were the same as the prior one. The gradient started from 5% to 95% over 20 min, then held at isocratic 95% B for 5 min, and then immediately stepped back down to 5% for 5 min of equilibration.

All compounds evaluated in biochemical and biophysical assays had a purity of greater than 95%, as determined by HPLC and NMR (spectra provided in the Supporting Information). Nuclear Magnetic Resonance Spectroscopy (NMR) ^1^H and ^13^C NMR spectra were obtained on a Bruker 400 MHz spectrometer at 400 and 101 MHz, or on Kerry (Bruker Avance III 500 MHz) spectrometer at 500 and 126 MHz, respectively. Chemical shifts are reported in ppm, and coupling constants are reported in Hz with CDCl_3_ referenced at 7.26 (1*H*) and 77.1 ppm (13C), DMSO-*d*_6_ referenced at 2.50 (1*H*) and 39.5 ppm (13C), and MeOH-*d*_4_ referenced at 3.31 (1*H*) and 49.0 ppm (13C). Signal splitting patterns were described as singlet(s), doublet (d), triplet (t), quartet (q), or multiplet (m). High-resolution mass spectra (HRMS) were obtained using an Electron Spray Ionization (ESI) mass spectrometer at the Analytical Instrument Center, School of Pharmacy. UNC8153 (CAS No.: 2929304-61-8) was purchased from commercial sources and used without further purification.

#### *N*-(2-Acrylamidoethyl)­benzamide (**E1**)

To a solution of *tert*-butyl (2-aminoethyl)­carbamate (800 mg, 5.0 mmol, 1.0 equiv) in DCM (5.0 mL) was added DMAP (61 mg, 0.5 mmol, 0.1 equiv), TEA (1.4 mL, 10.0 mmol, 2.0 equiv), after cooling to 0 °C under an ice bath, benzoyl chloride (700 mg, 5.0 mmol, 1.0 equiv) was injected dropwise, the reaction was stirred at room temperature for 2 h, the mixture was filtered, washed with DCM, and then concentrated to dryness. The residue was redissolved in DCM and then treated with a solution of hydrochloric acid in dioxane, stirred for 3 h, and the organic solvent was then removed under reduced pressure. The crude material was purified using column chromatography (silica gel, DCM/MeOH = 1:0 to 4:1) to afford *N*-(2-aminoethyl)­benzamide (300 mg, 1.83 mmol, 37% yield over two steps). The compound (81 mg, 0.49 mmol, 1.0 equiv) was subsequently dissolved in anhydrous DMF (1.0 mL), followed by acrylic acid (34 μL, 0.49 mmol, 1.0 equiv), HATU (372 mg, 0.98 mmol, 2.0 equiv), and DIPEA (426 μL, 2.45 mmol, 5.0 equiv), the reaction was stirred overnight and purified by column chromatography (silica gel, DCM/MeOH = 1:0 to 9:1) to obtain **E1** (65.2 mg, 0.3 mmol, 61%). LC–MS (ESI) calcd for C_12_H_15_N_2_O_2_^+^ [M + H]^+^ *m*/*z* 219.2; found, 219.1. ^1^H NMR (400 MHz, DMSO): δ 8.57 (t, *J* = 5.2 Hz, 1H), 8.27 (t, *J* = 5.4 Hz, 1H), 7.86 (dt, *J* = 6.9, 1.5 Hz, 2H), 7.60–7.36 (m, 3H), 6.23 (dd, *J* = 17.1, 10.0 Hz, 1H), 6.10 (dd, *J* = 17.1, 2.3 Hz, 1H), 5.59 (dd, *J* = 10.0, 2.3 Hz, 1H), 3.35 (dq, *J* = 11.4, 5.7 Hz, 4H). ^13^C NMR (101 MHz, DMSO): δ 166.5, 165.0, 134.5, 131.8, 131.2, 128.3, 127.2, 125.1, 39.2, 38.4.

#### *N*-(3-Acrylamidopropyl)­benzamide (**E2**)

The procedure was the same as E1, Yield: 67.9 mg (26%). ^1^H NMR (400 MHz, DMSO): δ 8.48 (t, *J* = 5.7 Hz, 1H), 8.15 (t, *J* = 5.7 Hz, 1H), 7.95–7.78 (m, 2H), 7.57–7.38 (m, 3H), 6.23 (dd, *J* = 17.1, 10.1 Hz, 1H), 6.09 (dd, *J* = 17.1, 2.3 Hz, 1H), 5.58 (dd, *J* = 10.1, 2.3 Hz, 1H), 3.29 (q, *J* = 6.6 Hz, 2H), 3.20 (q, *J* = 6.6 Hz, 2H), 1.70 (p, *J* = 7.0 Hz, 2H). ^13^C NMR (101 MHz, DMSO): δ 78.6, 77.1, 47.0, 44.2, 43.5, 40.7, 39.5, 37.4, 50.5, 51.1, 58.3. LC–MS (ESI) calcd for C_13_H_17_N_2_O_2_^+^ [M + H]^+^ *m*/*z* 233.1; found, 233.2.

#### *N*-(4-Acrylamidobutyl)­benzamide (**E3**)

The procedure was the same as E1, Yield: 58.4 mg (23%). ^1^H NMR (400 MHz, DMSO): δ 8.45 (t, *J* = 5.5 Hz, 1H), 8.08 (t, *J* = 5.8 Hz, 1H), 7.88–7.77 (m, 2H), 7.55–7.48 (m, 1H), 7.45 (dd, *J* = 8.2, 6.4 Hz, 2H), 6.20 (dd, *J* = 17.1, 10.1 Hz, 1H), 6.06 (dd, *J* = 17.1, 2.3 Hz, 1H), 5.55 (dd, *J* = 10.1, 2.4 Hz, 1H), 3.26 (q, *J* = 6.3 Hz, 2H), 3.15 (q, *J* = 6.4 Hz, 2H), 1.50 (ddddd, *J* = 18.6, 9.2, 7.3, 4.7, 2.2 Hz, 4H). ^13^C NMR (101 MHz, DMSO): δ 166.1, 164.5, 134.7, 131.9, 131.0, 128.2, 127.1, 124.8, 38.3, 26.7, 26.6. LC–MS (ESI) calcd for C_14_H_19_N_2_O_2_^+^ [M + H]^+^ *m*/*z* 247.1; found, 247.2.

#### *N*-(2-(2-Chloroacetamido)­ethyl)­benzamide (**E4**)

To a solution of *tert*-butyl (2-aminoethyl)­carbamate (800 mg, 5.0 mmol, 1.0 equiv) in DCM (5.0 mL) was added DMAP (61 mg, 0.5 mmol, 0.1 equiv), TEA (1.4 mL, 10.0 mmol, 2.0 equiv), after cooling to 0 °C under an ice bath, benzoyl chloride (700 mg, 5.0 mmol, 1.0 equiv) was injected dropwise, the reaction was stirred at room temperature for 2 h, the mixture was filtered, washed with DCM, and then concentrated to dryness. The residue was redissolved in DCM and then treated with a solution of hydrochloric acid in dioxane, stirred for 3 h, and the organic solvent was then removed under reduced pressure. The crude material was purified using column chromatography (silica gel, DCM/MeOH = 1:0 to 4:1) to afford *N*-(2-aminoethyl)­benzamide (300 mg, 1.83 mmol, 37% yield over two steps). The compound (104 mg, 0.63 mmol, 1.0 equiv) was subsequently dissolved in DCM (1.0 mL), followed by Chloroacetyl chloride (51 μL, 0.63 mmol, 1.0 equiv), and TEA (175 μL, 1.26 mmol, 2.0 equiv), the reaction was stirred overnight and purified by column chromatography (silica gel, DCM/MeOH = 1:0 to 9:1) to obtain **E4** (107.3 mg, 0.45 mmol, 71%). LC–MS (ESI) calcd for C_11_H_14_ClN_2_O_2_^+^ [M + H]^+^ *m*/*z* 241.1; found, 241.0. ^1^H NMR (400 MHz, DMSO): δ 8.52 (t, *J* = 5.5 Hz, 1H), 8.35 (t, *J* = 5.6 Hz, 1H), 7.92–7.77 (m, 2H), 7.57–7.40 (m, 3H), 4.07 (s, 2H), 3.36 (q, *J* = 6.5 Hz, 2H), 3.32–3.24 (m, 2H). ^13^C NMR (101 MHz, DMSO): δ 166.5, 166.2, 134.5, 131.1, 128.2, 127.2, 42.7, 38.9, 38.8.

#### *N*-(3-(2-Chloroacetamido)­propyl)­benzamide (**E5**)

The procedure was the same as E4, Yield:123.2 mg (31%). ^1^H NMR (400 MHz, DMSO): δ 8.47 (t, *J* = 5.7 Hz, 1H), 8.27 (t, *J* = 5.8 Hz, 1H), 7.90–7.78 (m, 2H), 7.47 (dt, *J* = 14.8, 7.2 Hz, 3H), 4.06 (s, 2H), 3.28 (q, *J* = 6.6 Hz, 2H), 3.16 (q, *J* = 6.6 Hz, 2H), 1.68 (p, *J* = 7.0 Hz, 2H). ^13^C NMR (101 MHz, DMSO): δ 166.3, 165.9, 134.6, 131.1, 128.3, 127.1, 42.7, 36.9, 29.0. LC–MS (ESI) calcd for C_12_H_16_ClN_2_O_2_^+^ [M + H]^+^ *m*/*z* 255.1; found, 255.2.

#### *N*-(4-(2-Chloroacetamido)­butyl)­benzamide (**E6**)

The procedure was the same as E4, Yield: 116.5 mg (29%). ^1^H NMR (400 MHz, DMSO): δ 8.46 (t, *J* = 5.7 Hz, 1H), 8.22 (t, *J* = 5.8 Hz, 1H), 7.89–7.78 (m, 2H), 7.57–7.36 (m, 3H), 4.03 (s, 2H), 3.26 (q, *J* = 6.4 Hz, 2H), 3.11 (q, *J* = 6.4 Hz, 2H), 1.69–1.39 (m, 4H). ^13^C NMR (101 MHz, DMSO): δ 166.1, 165.8, 134.7, 131.0, 128.2, 127.1, 42.7, 38.8, 38.7, 26.6, 26.5. LC–MS (ESI) calcd for C_13_H_18_ClN_2_O_2_^+^ [M + H]^+^ *m*/*z* 269.1; found, 269.2.

#### *N*-benzyl-4-formylbenzamide (**E7**)

To a solution of phenylmethanamine (214.0 mg, 2.0 mmol, 1.0 equiv) and 4-formylbenzoic acid (300.0 mg, 2.0 mmol, 1.0 equiv) in acetonitrile (2.0 mL) was added EDCI (460.0 mg, 2.4 mmol, 1.2 equiv), DMAP (488.0 mg, 4.0 mmol, 2.0 equiv). The reaction was stirred at room temperature overnight, and the mixture was concentrated to dryness. Column chromatography was utilized for purification (eluting with hexane/ethyl acetate = 1:0 to 0:1). ^1^H NMR (400 MHz, DMSO): δ 10.09 (s, 1H), 9.27 (t, *J* = 6.0 Hz, 1H), 8.19–8.06 (m, 2H), 8.05–7.98 (m, 2H), 7.37–7.31 (m, 4H), 7.25 (ddt, *J* = 8.6, 6.0, 3.1 Hz, 1H), 4.52 (d, *J* = 6.0 Hz, 2H).^13^C NMR (101 MHz, DMSO): δ 192.88, 165.43, 139.35, 137.82, 129.45, 128.32, 128.02, 127.28, 126.83, 126.49, 42.79 LC–MS (ESI) calcd for C_15_H_14_NO_2_^+^ [M + H]^+^ *m*/*z* 240.1; found, 240.2.

#### 4-(2-(3,4-dihydroisoquinolin-2­(1*H*)-yl)-2-oxoethyl)­benzaldehyde (**E8**)

To a solution of 1,2,3,4-tetrahydroisoquinoline (0.02 mL, 0.16 mmol, 1.1 equiv) in DMF (3.0 mL) was added 2-(4-formylphenyl)­acetic acid (25.0 mg, 0.15 mmol, 1.0 equiv), EDCI (56.0 mg, 0.29 mmol, 1.9 equiv), HOBt monohydrate (14.0 mg, 0.10 mmol, 0.7 equiv), and DMAP (45.0 mg, 0.37 mmol, 2.5 equiv). The reaction mixture was stirred at room temperature. After 24 h, the reaction mixture was poured into H_2_O (50 mL), and the precipitate was filtered to obtain the crude product. The crude product was purified by reverse-phase chromatography (5–55% acetonitrile in water +0.1% FA) to afford compound E8 as a white solid. (20.0 mg, 0.071 mmol, 47%). ^1^H NMR (400 MHz, CDCl_3_): δ 9.97 (d, *J* = 7.1 Hz, 1H), 7.83 (m, 2H), 7.44 (m, 3H), 7.25–7.10 (m, 3H), 4.77 (s, 1H), 4.62 (s, 1H), 3.90 (s, 2H), 3.86 (t, *J* = 6.2 Hz, 1H), 3.68 (t, *J* = 6.0 Hz, 1H), 2.87 (t, *J* = 6.1 Hz, 1H), 2.75 (t, *J* = 6.0 Hz, 1H). ^13^C NMR (101 MHz, CDCl_3_): δ 192.0, 169.2, 142.1, 135.3, 133.8, 133.2, 132.2, 130.3, 130.2, 129.8, 129.7, 129.0, 128.4, 127.2, 126.8, 126.8, 126.5, 126.0, 47.9, 44.7, 43.9, 41.4, 41.3, 40.3, 29.4, 28.5. (Rotamers were observed in NMR) LCMS (ESI) calcd for C_18_H_18_NO_2_^+^ [M + H]^+^ *m*/*z* 280.1; found, 280.1.

### General Procedure

**A**: the synthesis of intermediates (**SM**-**2**–**5**) with different amines.

To an oven-dried flask charged with a still bar was treated with SM-1 (1.0 equiv), Amine (1.2 equiv), and NMI (CAS No.: 616-47-7, 2.1 equiv) in sequence. The mixture was dissolved in anhydrous DMF (0.1 M) and stirred for 1 min before TCFH (CAS No.: 94790-35-9, 1.5 equiv) was added. The reaction was stirred at room temperature overnight and then diluted with water when the acid was consumed, as determined by TLC and LC–MS. Ethyl acetate was used for extraction (three times), and the combined organic phase was washed with brine, dried over sodium sulfate, filtered, and concentrated under reduced pressure. The residue was purified by column chromatography on silica gel (Hexane: EtOAc = 1:0 to 0:1) to afford the corresponding Boc-protected product. The acquired compound was dissolved in dichloromethane (0.2 M), followed by the addition of an equal volume of HCl solution (4 M in dioxane). The reaction was then stirred at room temperature for 12 h. Thereafter, the mixture was concentrated in vacuo, diluted with an aqueous solution of saturated sodium bicarbonate, and extracted with a mixed solvent (DCM/MeOH = 9:1) three times. The organic layer was dried over sodium sulfate, filtered, and the volatiles were removed in vacuo. The crude compound was used for the next step without further purification.

### General Procedure

**B**: the synthesis of PROTACs with an amide linkage (**T1**–**T10**, **T13**, **T18**–**T20**, **T21**–**T23**, **T27**–**T33**).

To a scintillation vial with a stir bar was added acid (1.0 equiv), EDCI (CAS No.: 25952-53-8,1.2 equiv), and HOBt (CAS No.: 123333-53-9,1.2 equiv) and DMF (1–2 mL). The mixture was stirred at room temperature for 10 min. To the vial was then added SM-2 or any one from SM-3 ∼5 (1.2 equiv) followed by DMAP (CAS No.: 1122-58-3, 1.2 equiv). The reaction was stirred at room temperature overnight. The reaction was then diluted with water and extracted three times with ethyl acetate. The combined organic layers were washed with saturated sodium bicarbonate, once with brine, then dried over sodium sulfate, filtered, and concentrated in vacuo. The residue was then dissolved in acetonitrile, filtered through a 0.1 μm PVDF filter, and purified by preparative HPLC and dried under lyophilization to afford the target compound.

### General Procedure

**C**: the synthesis of PROTACs with an alkyl linkage (**T11**–**T12**, **T14**–**T17**, **T24**–**T26**).

To a solution of SM-2 (1.0 equiv) in DMF (0.1 M) was added sodium bicarbonate (2.0 equiv), followed by bromide (1.05 equiv). The reaction was conducted at room temperature for 10 h, then diluted with water and extracted with ethyl acetate 3 times. The combined organic layers were dried over sodium sulfate, filtered, and concentrated under reduced pressure. The afforded crude compound was redissolved in acetonitrile and filtered through a 0.1 μm PVDF filter; the filtrate was then loaded onto a C18 reversed-phase HPLC column for purification. The pure compound solution was dried over lyophilization to give the target compound.

### General Procedure

**D**: the synthesis of PROTACs with a carbamate linkage (**T21**).

To a solution of SM-2 (1.0 equiv) in DMF (0.1 M) was added CDI (2.0 equiv) and triethylamine (2.0 equiv). The mixture was stirred at room temperature for 30 min. To the vial was then added benzene dimethanol (3.0 equiv) followed by NaH (3.0 equiv). The reaction was stirred at room temperature for 10 h, then diluted with water and extracted with ethyl acetate 3 times. The combined organic layers were dried over sodium sulfate, filtered, and concentrated under reduced pressure. The residue was purified by column chromatography on silica gel (Dichloromethane: MeOH = 1:0 to 9:1) to afford the corresponding carbamate product. The acquired compound was dissolved in DMF (0.1 M), followed by the addition of DMP (2.0 equiv). The reaction was stirred at 80 °C for 10 h, then diluted with water and extracted with ethyl acetate 3 times. The combined organic layers were dried over sodium sulfate, filtered, and concentrated under reduced pressure. The afforded crude compound was redissolved in acetonitrile and filtered through a 0.1 μm PVDF filter; the filtrate was then loaded onto a C18 reversed-phase HPLC column for purification. The pure compound solution was dried over lyophilization to give the target compound.

#### *N*-cyclopropyl-*N*-(4-((4-((4-formylbenzamido)­methyl) phenyl)­carbamoyl)­benzyl)-3-oxo-3,4-dihydro-2*H*-benzo­[*b*] [1,4]­oxazine-7-Carboxamide­(**T1**)

Yield: 15.5 mg (43%) ^1^H ^1^H NMR (400 MHz, DMSO): δ 10.90 (s, 1H), 10.24 (s, 1H), 10.09 (s, 1H), 9.26 (t, *J* = 6.0 Hz, 1H), 8.08 (d, *J* = 8.1 Hz, 2H), 8.01 (d, *J* = 8.3 Hz, 2H), 7.94 (d, *J* = 8.1 Hz, 2H), 7.73 (d, *J* = 8.3 Hz, 2H), 7.44 (d, *J* = 8.0 Hz, 2H), 7.32 (d, *J* = 8.3 Hz, 2H), 7.21–7.13 (m, 2H), 6.93 (d, *J* = 8.0 Hz, 1H), 4.71 (s, 2H), 4.61 (s, 2H), 4.48 (d, *J* = 5.9 Hz, 2H), 2.84–2.74 (m, 1H), 0.57–0.45 (m, 4H). ^13^C NMR (101 MHz, DMSO): δ 193.4, 165.9, 165.7, 165.3, 146.8, 143.0, 142.7, 139.9, 138.5, 138.3, 135.0, 134.1, 129.9, 128.9, 128.5, 128.4, 128.1, 127.6, 122.3, 120.8, 115.8, 115.6, 67.2, 55.5, 50.4, 42.9, 10.0. HRMS (ESI) calcd for C_35_H_30_N_4_NaO_6_^+^ [M + Na]^+^ *m*/*z* 625.2058; found, 625.2044.

#### *N*-cyclopropyl-*N*-(4-((4-((2-(4-formylphenyl)­acetamid o)­methyl)­phenyl)­carbamoyl)­benzyl)-3-oxo-3,4-dihydro-2*H*-benzo­[*b*]­[1,4]­oxazine-7-Carboxamide (**T2**)

Yield: 11.4 mg (37%) ^1^H NMR (400 MHz, DMSO): δ 10.90 (s, 1H), 10.22 (s, 1H), 9.98 (s, 1H), 7.94 (d, *J* = 8.0 Hz, 2H), 7.86 (d, *J* = 7.8 Hz, 2H), 7.70 (d, *J* = 8.3 Hz, 2H), 7.51 (d, *J* = 7.8 Hz, 2H), 7.44 (d, *J* = 7.9 Hz, 2H), 7.28–7.10 (m, 4H), 6.93 (d, *J* = 8.1 Hz, 1H), 4.79–4.67 (m, 2H), 4.61 (s, 2H), 4.25 (d, *J* = 5.7 Hz, 2H), 3.61 (s, 2H), 2.84–2.74 (m, 1H), 0.69–0.26 (m, 4H). ^13^C NMR (101 MHz, DMSO): δ 192.7, 169.2, 165.2, 164.8, 143.6, 142.5, 142.1, 138.0, 134.7, 134.5, 133.6, 131.8, 129.9, 129.5, 128.4, 128.0, 127.5, 127.2, 121.9, 120.3, 115.3, 115.2, 110.5, 73.8, 68.8, 66.7, 42.4, 41.9, 13.4, 9.4. HRMS (ESI) calcd for C_36_H_32_N_4_NaO_6_^+^ [M + Na]^+^ *m*/*z* 639.2214; found, 639.2203.

#### 3-(4-formylphenyl)­propanoic Acid (**SI**-**2**)

An oven-dried flask charged with 4-bromobenzaldehyde (930 mg, 5.0 mmol, 1.0 equiv), Pd­(OAc)_2_ (33.7 mg, 0.15 mmol, 0.03 equiv) and TBACl (1.39 g, 5.0 mmol, 1.0 equiv) were evacuated and backfilled with argon three times. DMF (20 mL), TEA (1.4 mL, 10 mmol, 2.0 equiv), and 3,3-diethoxyprop-1-ene (2.3 mL, 15 mmol, 3.0 equiv) were subsequently injected into the reaction. The flask was sealed with parafilm, equipped with a balloon filled with argon, and allowed to stir at 90 °C in an oil bath. When the starting material was completely converted (monitored by TLC), the reaction was quenched with water, extracted with EtOAc three times, washed with brine, dried over sodium sulfate, filtered, concentrated, and then purified by flash column chromatography to afford SI-1, the ester was then treated with LiOH·H_2_O (147 mg, 3.5 mmol, 10.0 equiv), followed by mixed solvent (methanol/water = 1/1), the reaction was stirred at room temperature overnight, after the removal of methanol in vacuo, the aqueous solution was acidified by 1 N HCl to PH = 3, ethyl acetate was utilized for extraction and afford the crude **SI**-**2** (356 mg, 2.0 mmol, 40% over two steps). ^1^H NMR (400 MHz, CDCl_3_): δ 9.89 (s, 1H), 7.72 (d, *J* = 8.2 Hz, 1H), 7.29 (d, *J* = 8.1 Hz, 1H), 4.04 (q, *J* = 7.1 Hz, 1H), 2.95 (t, *J* = 7.6 Hz, 1H), 2.57 (t, *J* = 7.6 Hz, 1H), 1.14 (t, *J* = 7.1 Hz, 2H).

#### *N*-cyclopropyl-*N*-(4-((4-((3-(4-formylphenyl)­propana mido)­methyl)­phenyl)­carbamoyl)­benzyl)-3-oxo-3,4-dihydro-2*H*-benzo­[*b*]­[1,4]­oxazine-7-Carboxamide (**T3**)

Yield: 9.1 mg (29%). ^1^H NMR (400 MHz, DMSO): δ 10.90 (s, 1H), 10.21 (s, 1H), 9.97 (s, 1H), 8.33 (t, *J* = 5.9 Hz, 1H), 7.94 (d, *J* = 8.1 Hz, 2H), 7.87–7.76 (m, 2H), 7.72–7.61 (m, 2H), 7.51–7.38 (m, 4H), 7.19 (dd, *J* = 8.0, 1.8 Hz, 1H), 7.16–7.05 (m, 3H), 6.93 (d, *J* = 8.0 Hz, 1H), 4.71 (s, 2H), 4.62 (s, 2H), 4.22 (d, *J* = 5.8 Hz, 2H), 2.96 (t, *J* = 7.6 Hz, 2H), 2.84–2.74 (m, 1H), 2.59–2.48 (m, 2H, overlapped), 0.64–0.40 (m, 4H). ^13^C NMR (101 MHz, DMSO): δ 192.6, 170.9, 165.2, 164.8, 148.8, 142.5, 142.0, 137.8, 134.6, 134.4, 133.7, 131.8, 129.6, 129.2, 128.4, 128.0, 127.4, 127.2, 121.9, 120.2, 115.3, 115.2, 66.7, 50.2, 41.7, 36.3, 31.2, 9.5. HRMS (ESI) calcd for C_37_H_34_N_4_NaO_6_^+^ [M + Na]^+^ *m*/*z* 653.2371; found, 653.2374.

#### *N*-Cyclopropyl-*N*-(4-((4-(3-(4-formylphenyl)­propanam Ido)­phenyl)­carbamoyl)­benzyl)-3-oxo-3,4-dihydro-2*H*-benzo­[*b*]­[1,4]­oxazine-7-Carboxamide (**T4**)

Yield: 12.3 mg, (40%) ^1^H NMR (400 MHz, DMSO): δ 10.90 (s, 1H), 10.18 (s, 1H), 9.96 (s, 1H), 9.95 (s, 1H), 7.93 (d, *J* = 7.9 Hz, 2H), 7.84 (d, *J* = 7.8 Hz, 2H), 7.68 (d, *J* = 8.7 Hz, 2H), 7.54 (d, *J* = 8.6 Hz, 2H), 7.50 (d, *J* = 7.8 Hz, 2H), 7.44 (d, *J* = 7.9 Hz, 2H), 7.18 (dd, *J* = 8.0, 1.8 Hz, 1H), 7.14 (d, *J* = 1.7 Hz, 1H), 6.93 (d, *J* = 8.0 Hz, 1H), 4.71 (s, 2H), 4.61 (s, 2H), 3.02 (t, *J* = 7.6 Hz, 2H), 2.84–2.74 (m, 1H), 2.68 (t, *J* = 7.6 Hz, 2H), 0.60–0.41 (m, 4H). ^13^C NMR (101 MHz, DMSO): δ 192.7, 170.6, 169.8, 165.0, 164.9, 148.7, 142.5, 142.0, 135.0, 134.5, 134.4, 133.7, 131.8, 129.7, 129.1, 128.4, 127.9, 127.2, 121.9, 120.8, 119.3, 115.4, 115.2, 66.7, 52.5, 50.3, 37.2, 30.9, 9.5. HRMS (ESI) calcd for C_36_H_32_N_4_NaO_6_^+^ [M + Na]^+^ *m*/*z* 639.2214; found, 639.2213.

#### *N*-cyclopropyl-*N*-(4-((4-(2-(4-formylbenzamido)­ethyl) phenyl)­carbamoyl)­benzyl)-3-oxo-3,4-dihydro-2*H*-benzo­[*b*]­[1,4]­oxazine-7-Carboxamide (**T5**)

Yield: 14.5 mg, (47%). ^1^H NMR (400 MHz, DMSO): δ 10.90 (s, 1H), 10.19 (s, 1H), 10.08 (s, 1H), 8.79 (t, *J* = 5.6 Hz, 1H), 8.00 (s, 4H), 7.93 (d, *J* = 8.1 Hz, 2H), 7.73–7.66 (m, 2H), 7.44 (d, *J* = 7.9 Hz, 2H), 7.23 (d, *J* = 8.4 Hz, 2H), 7.20–7.10 (m, 2H), 6.93 (d, *J* = 8.0 Hz, 1H), 4.71 (s, 2H), 4.61 (s, 2H), 3.60–3.47 (m, 2H), 2.95–2.71 (m, 3H), 0.63–0.38 (m, 4H). ^13^C NMR (101 MHz, DMSO): δ 192.9, 165.3, 165.2, 164.8, 142.5, 142.0, 139.6, 137.4, 134.6, 133.7, 131.8, 129.4, 128.8, 128.4, 127.9, 127.9, 127.2, 121.9, 120.4, 115.3, 115.2, 66.7, 41.0, 34.4, 9.5. HRMS (ESI) calcd for C_36_H_32_N_4_NaO_6_^+^ [M + Na]^+^ *m*/*z* 639.2214; found, 639.2217.

#### *N*-cyclopropyl-*N*-(4-((4-((4-formyl-3-methoxybenza mido)­methyl)­phenyl)­carbamoyl)­benzyl)-3-oxo-3,4-dihydro-2*H*-benzo­[*b*]­[1,4]­oxazine-7-Carboxamide (**T6**)

Yield: 11.7 mg (37%). ^1^H NMR (400 MHz, DMSO): δ 10.89 (s, 1H), 10.39 (s, 1H), 10.23 (s, 1H), 9.25 (t, *J* = 5.9 Hz, 1H), 7.94 (d, *J* = 8.0 Hz, 2H), 7.77 (d, *J* = 8.0 Hz, 1H), 7.73 (d, *J* = 8.4 Hz, 2H), 7.68 (d, *J* = 1.4 Hz, 1H), 7.58 (d, *J* = 7.8 Hz, 1H), 7.44 (d, *J* = 7.9 Hz, 2H), 7.32 (d, *J* = 8.3 Hz, 2H), 7.18 (dd, *J* = 8.0, 1.8 Hz, 1H), 7.14 (d, *J* = 1.8 Hz, 1H), 6.93 (d, *J* = 8.0 Hz, 1H), 4.71 (s, 2H), 4.61 (s, 2H), 4.48 (d, *J* = 5.9 Hz, 2H), 3.99 (s, 3H), 2.84–2.74 (m, 1H), 0.58–0.42 (m, 4H). ^13^C NMR (101 MHz, DMSO): δ 189.0, 165.2, 165.1, 164.9, 161.3, 142.5, 142.1, 141.2, 138.0, 134.5, 133.6, 131.8, 128.4, 128.0, 127.9, 127.7, 127.2, 125.7, 121.9, 120.4, 119.4, 115.3, 115.2, 111.6, 66.7, 59.5, 56.2, 50.5, 42.5, 9.5. HRMS (ESI) calcd for C_36_H_32_N_4_NaO_7_^+^ [M + Na]^+^ *m*/*z* 655.2163; found, 655.2170.

#### *N*-cyclopropyl-*N*-(4-((4-((4-formyl-3-hydroxybenzamido)­methyl)­phenyl)­carbamoyl)­benzyl)-3-oxo-3,4-dihydro-2*H*-benzo­[*b*]­[1,4]­oxazine-7-carboxamide (**T7**)

Yield: 10.2 mg (33%) ^1^H NMR (400 MHz, DMSO): δ 10.88 (s, 1H), 10.32 (s, 1H), 10.22 (s, 1H), 9.13 (t, *J* = 6.0 Hz, 1H), 7.94 (d, *J* = 8.0 Hz, 2H), 7.71 (t, *J* = 8.9 Hz, 3H), 7.54–7.40 (m, 3H), 7.36 (d, *J* = 8.1 Hz, 1H), 7.29 (d, *J* = 8.2 Hz, 2H), 7.18 (d, *J* = 8.1 Hz, 1H), 7.14 (s, 1H), 6.93 (d, *J* = 8.0 Hz, 1H), 4.71 (s, 2H), 4.61 (s, 2H), 4.43 (d, *J* = 5.9 Hz, 2H), 2.84–2.74 (m, 1H), 0.66–0.33 (m, 4H). ^13^C NMR (101 MHz, DMSO): δ 190.8, 165.5, 165.2, 164.9, 142.5, 142.0, 141.3, 137.9, 134.6, 133.7, 131.8, 128.7, 128.4, 128.0, 127.6, 127.2, 124.1, 121.9, 120.3, 117.3, 116.9, 115.3, 115.2, 66.7, 53.5, 42.4, 9.5. HRMS (ESI) calcd for C_35_H_30_N_4_NaO_7_^+^ [M + Na]^+^ *m*/*z* 641.2007; found, 641.2005.

#### *N*-cyclopropyl-*N*-(4-((4-((3-fluoro-4-formylbenzamido)­me thyl)­phenyl)­carbamoyl)­benzyl)-3-oxo-3,4-dihydro-2*H*-benzo­[*b*]­[1,4]­oxazine-7-Carboxamide (**T8**)

Yield: 13.6 mg, (45%) as a white solid. ^1^H NMR (400 MHz, DMSO): δ 10.93 (s, 1H), 10.26 (s, 2H), 9.35 (t, *J* = 5.9 Hz, 1H), 7.95 (dd, *J* = 7.9, 6.0 Hz, 3H), 7.91–7.83 (m, 2H), 7.78–7.69 (m, 2H), 7.44 (d, *J* = 7.9 Hz, 2H), 7.32 (d, *J* = 8.4 Hz, 2H), 7.18 (dd, *J* = 8.1, 1.8 Hz, 1H), 7.14 (d, *J* = 1.8 Hz, 1H), 6.93 (d, *J* = 8.0 Hz, 1H), 4.71 (s, 2H), 4.61 (s, 2H), 4.47 (d, *J* = 5.8 Hz, 2H), 2.84–2.74 (m, 1H), 0.66–0.39 (m, 4H). ^13^C NMR (101 MHz, DMSO): δ 187.7, 187.6, 165.2, 164.8, 164.3, 164.1, 164.0, 161.7, 142.5, 142.0, 141.6, 141.6, 138.0, 134.2, 133.6, 131.8, 129.6, 129.6, 128.4, 128.0, 127.7, 127.2, 125.4, 125.4, 123.8, 123.8, 121.8, 120.3, 115.7, 115.5, 115.3, 115.2, 66.7, 50.2, 42.5, 9.5. HRMS (ESI) calcd for C_35_H_29_FN_4_NaO_6_^+^ [M + Na]^+^ *m*/*z* 643.1963; found, 643.1960.

#### *N*-cyclopropyl-*N*-(4-((2-(2-(4-formylphenyl)­acetyl)-1,2,3,4-tetrahydroisoquinolin-6-yl)­carbamoyl)­benzyl)-3-oxo-3,4-dihydro-2*H*-benzo­[*b*]­[1,4]­oxazine-7-carboxamide (**T9**)

Yield: 17.0 mg (32%). ^1^H NMR (400 MHz, DMSO): δ 10.87 (s, 1H), 10.17 (s, 1H), 9.98 (d, *J* = 3.9 Hz, 1H), 7.93 (d, *J* = 8.0 Hz, 2H), 7.85 (t, *J* = 8.0 Hz, 2H), 7.63 (d, *J* = 4.9 Hz, 1H), 7.59–7.52 (m, 1H), 7.52–7.40 (m, 4H), 7.22–7.11 (m, 3H), 6.93 (d, *J* = 8.1 Hz, 1H), 4.75–4.66 (m, 3H), 4.64–4.57 (m, 3H), 3.95 (s, 2H), 3.83–3.65 (m, 2H), 2.84–2.71 (m, 3H), 0.55 (d, *J* = 7.0 Hz, 2H), 0.52–0.40 (m, 2H). ^13^C NMR (101 MHz, DMSO): δ 192.7, 168.6, 165.2, 164.8, 142.5, 142.1, 134.6, 133.6, 131.8, 130.2, 130.1, 129.5, 129.4, 128.4, 127.9, 127.2, 126.6, 121.9, 120.0, 118.5, 115.3, 115.2, 111.3, 109.3, 108.8, 66.7, 43.5, 43.0, 29.0, 28.2, 9.5. HRMS (ESI) calcd for C_38_H_34_N_4_NaO_6_^+^ [M + Na]^+^ *m*/*z* 665.2371; found, 665.2356.

#### *N*-cyclopropyl-*N*-(4-((2-(2-(3-formylphenyl)­acetyl)-1,2,3,4-tetrahydroisoquinolin-6-yl)­carbamoyl)­benzyl)-3-oxo-3,4-dihydro-2*H*-benzo­[*b*]­[1,4]­oxazine-7-carboxamide (**T10**)

Yield: 11.0 mg (34%). ^1^H NMR (400 MHz, DMSO): δ 10.88 (s, 1H), 10.18 (d, *J* = 5.4 Hz, 1H), 9.99 (d, *J* = 6.8 Hz, 1H), 7.94 (d, *J* = 7.9 Hz, 2H), 7.79 (d, *J* = 8.0 Hz, 2H), 7.69–7.50 (m, 4H), 7.44 (d, *J* = 7.9 Hz, 2H), 7.22–7.11 (m, 3H), 6.93 (d, *J* = 8.0 Hz, 1H), 4.72 (d, *J* = 8.7 Hz, 3H), 4.61 (d, *J* = 4.3 Hz, 3H), 3.95 (d, *J* = 2.2 Hz, 2H), 3.80–3.65 (m, 2H), 2.85–2.73 (m, 3H), 0.65–0.38 (m, 4H). ^13^C NMR (126 MHz, DMSO): δ 193.2, 193.2, 168.9, 165.2, 165.2, 164.9, 142.5, 142.1, 137.6, 137.5, 137.3, 137.2, 136.2, 136.2, 135.7, 135.7, 135.0, 134.7, 133.6, 131.8, 130.2, 130.1, 129.1, 129.0, 128.8, 128.5, 128.4, 128.0, 127.9, 127.9, 127.2, 126.6, 126.4, 121.9, 120.1, 120.0, 118.5, 118.5, 115.4, 115.2, 66.7, 50.2, 46.5, 43.5, 43.0, 29.0, 28.2. Rotamers were observed in NMR spectra. HRMS (ESI) calcd for C_38_H_34_N_4_NaO_6_^+^ [M + Na]^+^ *m*/*z* 665.2371; found, 665.2363.

#### 4-(2-bromoethyl)­benzaldehyde (**SI**-**3**)

To a solution of 4-(2-bromoethyl)­benzoic acid (458 mg, 2.0 mmol, 1.0 equiv) in anhydrous THF (3.0 mL) was added BH_3_·THF (3 mL, 3.0 mmol, 1.5 equiv) under an ice bath. Then, the reaction was moved to room temperature and stirred for 12 h. Thereafter, the mixture was treated with aqueous ammonia chloride and extracted with ethyl acetate three times. The combined organic phase layer was dried over sodium sulfate and concentrated to dryness after filtration. Subsequently, the residue was redissolved in chloroform, and treated by Manganese oxide (261 mg, 3.0 mmol, 1.5 equiv). After additional 8 h stirring at 70 °C, quenched by sodium thiosulfate (saturated, aqueous), extracted by ethyl acetate, dried over sodium sulfate, concentrated and afford **SI**-**3** (204 mg, 0.95 mmol, 48% over two steps) without further purification. ^1^H NMR (400 MHz, CDCl_3_): δ 9.98 (s, 1H), 7.83 (d, *J* = 8.2 Hz, 2H), 7.38 (d, *J* = 8.0 Hz, 2H), 3.59 (t, *J* = 7.3 Hz, 2H), 3.24 (t, *J* = 7.3 Hz, 2H).

#### *N*-cyclopropyl-*N*-(4-((2-(4-formylphenethyl)-1,2,3,4-tetrahydroisoquinolin-6-yl)­carbamoyl)­benzyl)-3-oxo-3,4-dihydro-2*H*-benzo­[*b*]­[1,4]­oxazine-7-carboxamide (**T11**)

Yield: 9.8 mg (39%). ^1^H NMR (400 MHz, DMSO): δ 10.89 (s, 1H), 10.12 (s, 1H), 9.96 (s, 1H), 7.93 (d, *J* = 8.2 Hz, 2H), 7.86–7.81 (m, 2H), 7.56 (d, *J* = 2.1 Hz, 1H), 7.54–7.46 (m, 3H), 7.44 (d, *J* = 8.0 Hz, 2H), 7.18 (dd, *J* = 8.0, 1.8 Hz, 1H), 7.14 (d, *J* = 1.7 Hz, 1H), 7.02 (d, *J* = 8.4 Hz, 1H), 6.93 (d, *J* = 8.0 Hz, 1H), 4.71 (s, 2H), 4.61 (s, 2H), 3.60 (s, 1H), 2.95 (t, *J* = 7.4 Hz, 2H), 2.84–2.70 (m, 8H), 0.58–0.42 (m, 4H). ^13^C NMR (101 MHz, DMSO): δ 192.7, 165.2, 164.9, 148.2, 142.5, 142.0, 139.4, 137.1, 134.3, 134.3, 133.7, 131.8, 130.3, 129.6, 129.6, 128.4, 127.9, 127.2, 126.5, 121.9, 120.1, 118.1, 115.4, 66.8, 58.8, 55.1, 50.3, 48.6, 33.0, 28.9, 8.6. HRMS (ESI) calcd for C_38_H_36_N_4_NaO_5_^+^ [M + Na]^+^ *m*/*z* 651.2578; found, 651.2570.

#### *N*-cyclopropyl-*N*-(4-((2-(2-formylphenethyl)-1,2,3,4-tetrahydroisoquinolin-6-yl)­carbamoyl)­benzyl)-3-oxo-3,4-dihydro-2*H*-benzo­[*b*]­[1,4]­oxazine-7-carboxamide (**T12**)

Yield: 8.8 mg (35%) ^1^H NMR (400 MHz, DMSO): δ 10.88 (s, 1H), 10.17 (s, 1H), 10.12 (s, 1H), 7.93 (d, *J* = 8.2 Hz, 2H), 7.87–7.78 (m, 1H), 7.65–7.54 (m, 2H), 7.52–7.38 (m, 5H), 7.19 (dd, *J* = 8.1, 1.8 Hz, 1H), 7.14 (d, *J* = 1.8 Hz, 1H), 7.02 (d, *J* = 8.4 Hz, 1H), 6.93 (d, *J* = 8.0 Hz, 1H), 4.71 (s, 2H), 4.62 (s, 2H), 3.62 (s, 2H), 3.28 (s, 2H), 2.87–2.73 (m, 5H), 2.70 (t, *J* = 7.5 Hz, 2H), 0.74–0.34 (m, 4H). ^13^C NMR (101 MHz, DMSO): δ 191.9, 171.1, 165.6, 165.3, 164.3, 143.3, 143.0, 142.4, 137.6, 134.7, 134.3, 134.2, 132.3, 132.0, 131.4, 130.6, 128.9, 128.4, 127.6, 127.2, 126.9, 122.3, 120.5, 118.5, 115.8, 115.6, 59.8, 55.6, 50.7, 29.6, 29.2, 10.0. HRMS (ESI) calcd for C_38_H_37_N_4_O_5_^+^ [M + H]^+^ *m*/*z* 629.2758; found, 629.2763.

#### *N*-cyclopropyl-*N*-(4-((2-(3-(4-formylphenyl)­propanoyl)-1,2,3,4-tetrahydroisoquinolin-6-yl)­carbamoyl)­benzyl)-3-oxo-3,4-dihydro-2*H*-benzo­[*b*]­[1,4]­oxazine-7-carboxamide (**T13**)

Yield: 20.3 mg (60%). ^1^H NMR (400 MHz, DMSO): δ 10.83 (s, 1H), 10.12 (s, 1H), 9.88 (d, *J* = 9.5 Hz, 1H), 7.87 (d, *J* = 8.1 Hz, 2H), 7.79–7.68 (m, 2H), 7.62–7.53 (m, 1H), 7.52–7.29 (m, 5H), 7.17–7.00 (m, 3H), 6.86 (d, *J* = 8.1 Hz, 1H), 4.64 (s, 2H), 4.58–4.45 (m, 4H), 3.69–3.50 (m, 2H), 2.93–2.85 (m, 2H), 2.80–2.60 (m, 5H), 0.63–0.25 (m, 4H). ^13^C NMR (126 MHz, DMSO): δ 192.7, 192.6, 170.0, 165.2, 164.9, 149.1, 149.0, 142.5, 142.1, 137.5, 137.4, 135.0, 134.8, 134.3, 133.7, 131.8, 129.6, 129.5, 129.3, 129.3, 129.0, 128.6, 128.4, 127.9, 127.2, 126.6, 126.4, 121.9, 120.0, 120.0, 118.5, 118.4, 115.4, 115.2, 66.7, 46.0, 43.3, 42.5, 33.9, 33.5, 30.8, 30.8, 29.0, 28.3, 9.5. Rotamers were observed in NMR spectra. HRMS (ESI) calcd for C_39_H_36_N_4_O_6_^+^ [M + Na]^+^ *m*/*z* 679.2527; found, 679.2507.

#### 4-(2-bromoethoxy)­benzaldehyde (**SI**-**4**)

To a solution of 1,2-dibromoethane (1.1 mL, 12.70 mmol, 5.1 equiv) in DMF (5 mL) was added potassium carbonate (735.0 mg, 5.32 mmol, 2.1 equiv) and 4-hydroxybenzaldehyde (300.0 mg, 2.46 mmol, 1.0 equiv). The reaction mixture was stirred at room temperature. After 3 days, the mixture was partitioned between EtOAc and H_2_O, and extracted by EtOAc. The organic layers were dried over sodium sulfate and concentrated in vacuo. The residue was purified by column chromatography with hexane/EtOAc as eluent to afford **SI**-**4** as white solid. (316 mg, 1.38 mmol, 56%). ^1^H NMR (400 MHz, CDCl_3_): δ 9.90 (s, 1H), 7.85 (d, *J* = 8.8 Hz, 2H), 7.02 (d, *J* = 8.8 Hz, 2H), 4.38 (t, *J* = 6.2 Hz, 2H), 3.67 (t, *J* = 6.2 Hz, 2H). ^13^C NMR (101 MHz, CDCl_3_): δ 190.81, 163.09, 132.12, 130.56, 114.97, 68.04, 28.61.

#### *N*-cyclopropyl-*N*-(4-((2-(2-(4-formylphenoxy)­ethyl)-1,2,3,4-tetrahydroisoquinolin-6-yl)­carbamoyl)­benzyl)-3-oxo-3,4-dihydro-2*H*-benzo­[*b*]­[1,4]­oxazine-7-carboxamide (**T14**)

Yield: 17.0 mg (33%). ^1^H NMR (400 MHz, DMSO): δ 10.88 (s, 1H), 10.11 (s, 1H), 9.87 (s, 1H), 7.93 (d, *J* = 8.3 Hz, 2H), 7.87 (d, *J* = 8.7 Hz, 2H), 7.57 (d, *J* = 2.2 Hz, 1H), 7.49 (dd, *J* = 8.3, 2.2 Hz, 1H), 7.43 (d, *J* = 7.9 Hz, 2H), 7.22–7.12 (m, 4H), 7.02 (d, *J* = 8.4 Hz, 1H), 6.93 (d, *J* = 8.0 Hz, 1H), 4.71 (s, 2H), 4.61 (s, 2H), 4.31 (t, *J* = 5.7 Hz, 2H), 3.66 (s, 2H), 2.91 (t, *J* = 5.7 Hz, 2H), 2.80 (m, 5H), 0.54 (d, *J* = 7.3 Hz, 2H), 0.51–0.40 (m, 2H). ^13^C NMR (101 MHz, DMSO): δ 191.3, 165.1, 164.8, 163.5, 142.5, 142.0, 137.1, 134.2, 133.7, 131.8, 130.2, 129.6, 128.4, 127.9, 127.2, 126.5, 121.9, 120.1, 118.0, 115.4, 115.2, 115.0, 66.7, 66.2, 56.2, 55.3, 50.8, 28.9, 9.5. HRMS (ESI) calcd for C_38_H_37_N_4_O_6_^+^ [M + H]^+^ *m*/*z* 645.2708; found, 645.2699.

#### 3-(2-bromoethoxy)­benzaldehyde (**SI**-**5**)

To a solution of 3-hydroxybenzaldehyde (703 mg, 5.76 mmol, 1.0 equiv) in anhydrous DMSO (1.0 mL) was added Cs_2_CO_3_ (3.76 g, 11.5 mmol, 2.0 equiv), followed by 1,2-dibromoethane (3.23 g, 17.3 mmol, 3.0 equiv). The reaction was warmed to 60 °C overnight and then diluted with water. It was subsequently extracted with ethyl acetate three times. The combined organic layers were dried over sodium sulfate, filtered, and concentrated to dryness. The residue was purified via column chromatography using hexane/ethyl acetate (1:0 to 1:1) as eluent to afford **SI**-**5** (480 mg, 2.1 mmol, 36%). ^1^H NMR (400 MHz, CDCl_3_): δ 9.93 (s, 1H), 7.47–7.40 (m, 2H), 7.35 (dd, *J* = 2.8, 1.3 Hz, 1H), 7.17 (ddd, *J* = 7.5, 2.7, 1.7 Hz, 1H), 4.31 (t, *J* = 6.1 Hz, 2H), 3.64 (t, *J* = 6.1 Hz, 2H).

#### *N*-cyclopropyl-*N*-(4-((2-(2-(3-formylphenoxy)­ethyl)-1,2,3,4-tetrahydroisoquinolin-6-yl)­carbamoyl)­benzyl)-3-oxo-3,4-dihydro-2*H*-benzo­[*b*]­[1,4]­oxazine-7-carboxamide (**T15**)

Yield: 4.7 mg (18%). ^1^H NMR (400 MHz, DMSO): δ 10.17 (s, 1H), 9.94 (s, 1H), 7.98–7.90 (m, 2H), 7.61 (d, *J* = 2.2 Hz, 1H), 7.54 (dd, *J* = 8.3, 2.1 Hz, 1H), 7.51–7.48 (m, 2H), 7.46 (dd, *J* = 8.5, 2.9 Hz, 3H), 7.38 (d, *J* = 2.6 Hz, 1H), 7.34–7.26 (m, 1H), 7.25–7.18 (m, 2H), 7.09 (d, *J* = 8.4 Hz, 1H), 4.76–4.66 (m, 4H), 4.40–4.35 (m, 2H), 4.34–4.29 (m, 2H), 4.03 (s, 2H), 3.20–3.12 (m, 2H), 2.86–2.77 (m, 3H), 0.58–0.46 (m, 4H).^13^C NMR (101 MHz, DMSO): δ 192.9, 165.2, 164.3, 158.6, 144.2, 137.6, 133.7, 132.4, 130.4, 129.8, 127.9, 127.2, 126.7, 122.7, 122.0, 121.2, 120.3, 118.5, 115.7, 115.5, 113.7, 67.1, 65.1, 56.5, 50.1, 41.4, 28.9, 26.4, 9.5. HRMS (ESI) calcd for C_38_H_37_N_4_O_6_^+^ [M + H]^+^ *m*/*z* 645.2708; found, 645.2707.

#### 2-(2-bromoethoxy)­benzaldehyde (**SI**-**6**)

To a solution of 2-hydroxybenzaldehyde (976 mg, 8.0 mmol, 1.0 equiv) in anhydrous DMF (1.0 mL) was added Cs_2_CO_3_ (5.2 g, 16.0 mmol, 2.0 equiv), followed by 1,2-dibromoethane (4.5 g, 24.0 mmol, 3.0 equiv). The reaction was warmed to 80 °C overnight and then diluted with water. It was subsequently extracted with ethyl acetate three times. The combined organic layers were dried over sodium sulfate, filtered, and concentrated to dryness. The residue was purified via column chromatography using a gradient of hexane/ethyl acetate (1:0 to 1:1) as the eluent, affording **SI**-**6** (696 mg, 3.0 mmol, 38%). ^1^H NMR (400 MHz, CDCl_3_): δ 10.53 (d, *J* = 0.9 Hz, 1H), 7.83 (dd, *J* = 7.7, 1.9 Hz, 1H), 7.54 (ddd, *J* = 8.4, 7.3, 1.9 Hz, 1H), 7.08–7.02 (m, 1H), 6.95 (dd, *J* = 8.4, 1.0 Hz, 1H), 4.40 (t, *J* = 5.9 Hz, 2H), 3.70 (t, *J* = 5.9 Hz, 2H).

#### *N*-cyclopropyl-*N*-(4-((2-(2-(2-formylphenoxy)­ethyl)-1,2,3,4-tetrahydroisoquinolin-6-yl)­carbamoyl)­benzyl)-3-oxo-3,4-dihydro-2*H*-benzo­[*b*]­[1,4]­oxazine-7-carboxamide (**T16**)

Yield: 10.9 mg (35%). ^1^H NMR (400 MHz, DMSO): δ 10.81 (s, 1H), 10.35 (s, 1H), 10.05 (s, 1H), 7.86 (d, *J* = 8.0 Hz, 2H), 7.67–7.56 (m, 2H), 7.50 (d, *J* = 2.2 Hz, 1H), 7.42 (dd, *J* = 8.3, 2.2 Hz, 1H), 7.37 (d, *J* = 7.9 Hz, 2H), 7.23 (d, *J* = 8.4 Hz, 1H), 7.12 (dd, *J* = 8.0, 1.9 Hz, 1H), 7.07 (d, *J* = 1.8 Hz, 1H), 7.02 (t, *J* = 7.4 Hz, 1H), 6.95 (d, *J* = 8.4 Hz, 1H), 6.86 (d, *J* = 8.0 Hz, 1H), 4.64 (s, 2H), 4.55 (s, 2H), 4.29 (t, *J* = 5.6 Hz, 2H), 3.63 (s, 2H), 2.91 (t, *J* = 5.6 Hz, 2H), 2. 2.65 (s, 5H), 0.53–0.35 (m, 4H). ^13^C NMR (101 MHz, DMSO): δ 189.3, 165.1, 164.8, 160.9, 142.5, 142.0, 137.1, 136.4, 134.1, 133.7, 131.8, 130.0, 128.4, 127.9, 127.6, 127.2, 126.5, 124.4, 121.9, 120.8, 120.1, 118.0, 115.4, 115.1, 113.9, 66.7, 59.6, 56.1, 55.2, 50.7, 28.8, 9.5. HRMS (ESI) calcd for C_40_H_42_N_3_O_5_^+^ [M + H]^+^ *m*/*z* 645.2708; found, 645.2694.

#### 2-(3-bromopropoxy)­benzaldehyde (**SI**-**7**)

To a solution of 2-hydroxybenzaldehyde (610 mg, 5.0 mmol, 1.0 equiv) in anhydrous DMF (5.0 mL) was added Cs_2_CO_3_ (3.26 g, 10.0 mmol, 2.0 equiv), followed by 1,3-dibromopropane (2.01 g, 10.0 mmol, 2.0 equiv). The reaction was warmed to 80 °C overnight and then diluted with water. It was subsequently extracted with ethyl acetate three times. The combined organic layers were dried over sodium sulfate, filtered, and concentrated to dryness. The residue was purified via column chromatography using a gradient of hexane/ethyl acetate (1:0 to 1:1) as the eluent, affording **SI**-**7** (486 mg, 2.0 mmol, 40%). ^1^H NMR (400 MHz, CDCl3): δ 10.47 (s, 1H), 7.82 (dd, *J* = 7.7, 1.9 Hz, 1H), 7.54 (ddd, *J* = 8.9, 7.4, 1.9 Hz, 1H), 7.15–6.77 (m, 3H), 4.22 (t, *J* = 5.8 Hz, 3H), 3.62 (t, *J* = 6.3 Hz, 3H), 2.50–2.19 (m, 2H).

#### *N*-cyclopropyl-*N*-(4-((2-(3-(2-formylphenoxy)­propyl)-1,2,3,4-tetrahydroisoquinolin-6-yl)­carbamoyl)­benzyl)-3-oxo-3,4-dihydro-2*H*-benzo­[*b*]­[1,4]­oxazine-7-carboxamide (**T17**)

Yield: 9.4 mg (37%). ^1^H NMR (400 MHz, DMSO): δ 10.88 (s, 1H), 10.43 (s, 1H), 10.11 (d, *J* = 2.3 Hz, 1H), 7.93 (d, *J* = 8.1 Hz, 2H), 7.69 (dd, *J* = 7.7, 1.8 Hz, 1H), 7.64 (ddd, *J* = 8.9, 7.3, 1.9 Hz, 1H), 7.56 (d, *J* = 2.1 Hz, 1H), 7.53–7.39 (m, 3H), 7.24 (d, *J* = 8.4 Hz, 1H), 7.18 (dd, *J* = 8.1, 1.8 Hz, 1H), 7.14 (d, *J* = 1.7 Hz, 1H), 7.11–7.00 (m, 2H), 6.94 (t, *J* = 7.6 Hz, 1H), 4.71 (s, 2H), 4.61 (s, 2H), 4.22 (t, *J* = 6.2 Hz, 2H), 3.56 (s, 2H), 2.88–2.74 (m, 3H), 2.74–2.61 (m, 4H), 2.13–1.94 (m, 2H), 0.69–0.34 (m, 4H). ^13^C NMR (101 MHz, CDCl_3_): δ 189.3, 165.1, 164.8, 161.1, 142.5, 142.0, 137.1, 136.5, 134.3, 133.7, 131.8, 130.3, 128.4, 127.9, 127.6, 127.2, 126.5, 124.3, 121.9, 120.6, 120.0, 118.0, 115.3, 115.1, 113.6, 66.7, 55.3, 54.1, 50.5, 29.0, 26.2, 9.5. HRMS (ESI) calcd for C_39_H_39_N_4_O_6_^+^ [M + H]^+^ *m*/*z* 659.2864; found, 659.2867.

#### *N*-cyclopropyl-*N*-(4-((2-(2-(4-formylphenoxy)­acetyl)-1,2,3,4-tetrahydroisoquinolin-6-yl)­carbamoyl)­benzyl)-3-oxo-3,4-dihydro-2*H*-benzo­[*b*]­[1,4]­oxazine-7-carboxamide (**T18**)

Yield: 17.0 mg (65%). ^1^H NMR (400 MHz, DMSO): δ 10.88 (s, 1H), 10.20 (d, *J* = 6.7 Hz, 1H), 9.87 (s, 1H), 7.94 (d, *J* = 8.1 Hz, 2H), 7.85 (d, *J* = 8.8 Hz, 2H), 7.73–7.51 (m, 2H), 7.45 (d, *J* = 7.9 Hz, 2H), 7.24–7.09 (m, 5H), 6.93 (d, *J* = 8.1 Hz, 1H), 5.11 (s, 2H), 4.80–4.64 (m, 3H), 4.63–4.57 (m, 3H), 3.76–3.65 (m, 2H), 3.17 (d, *J* = 3.8 Hz, 1H), 2.93 (t, *J* = 6.0 Hz, 1H), 2.84–2.74 (m, 1H), 0.55 (d, *J* = 6.9 Hz, 2H), 0.52–0.41 (m, 2H). ^13^C NMR (101 MHz, DMSO): δ 191.3, 165.7, 165.7, 165.2, 164.8, 163.2, 142.5, 142.1, 137.6, 134.7, 133.6, 131.8, 131.6, 129.8, 128.4, 127.9, 127.2, 126.7, 121.9, 120.0, 118.6, 115.4, 115.2, 66.7, 66.0, 48.6, 43.4, 41.7, 28.8, 28.2, 9.5. HRMS (ESI) calcd for C_38_H_34_N_4_NaO_7_^+^ [M + Na]^+^ *m*/*z* 681.2320; found, 681.2302.

#### *N*-cyclopropyl-*N*-(4-((2-(2-(3-formylphenoxy)­acetyl)-1,2,3,4-tetrahydroisoquinolin-6-yl)­carbamoyl)­benzyl)-3-oxo-3,4-dihydro-2*H*-benzo­[*b*]­[1,4]­oxazine-7-carboxamide (**T19**)

Yield: 14.0 mg (44%). ^1^H NMR (500 MHz, DMSO): δ 10.20 (d, *J* = 7.8 Hz, 1H), 9.96 (d, *J* = 2.0 Hz, 1H), 7.94 (d, *J* = 8.0 Hz, 2H), 7.68–7.51 (m, 4H), 7.47–7.40 (m, 3H), 7.30 (tt, *J* = 7.0, 3.2 Hz, 1H), 7.18 (dd, *J* = 8.5, 3.2 Hz, 2H), 7.14 (d, *J* = 1.7 Hz, 1H), 6.93 (d, *J* = 8.0 Hz, 1H), 5.04 (d, *J* = 2.3 Hz, 2H), 4.77–4.65 (m, 3H), 4.63–4.57 (m, 1H), 3.74–3.66 (m, 2H), 2.92 (t, *J* = 5.9 Hz, 1H), 2.79 (t, *J* = 6.0 Hz, 2H), 0.58–0.43 (m, 4H). ^13^C NMR (126 MHz, DMSO): δ 193.6, 171.3, 166.7, 166.7, 165.9, 165.5, 159.3, 143.1, 142.7, 138.2, 135.6, 135.3, 134.3, 132.4, 130.9, 129.0, 128.8, 128.6, 127.8, 127.3, 127.1, 123.5, 122.5, 122.1, 120.8, 120.7, 119.2, 116.0, 115.8, 114.7, 114.5, 67.4, 66.7, 50.9, 45.6, 44.0, 42.4, 32.3, 29.5, 28.8, 10.2. Rotamers were observed in NMR spectra. HRMS (ESI) calcd for C_38_H_34_N_4_NaO_7_^+^ [M + Na]^+^ *m*/*z* 681.2320; found, 659. 681.2307.

#### *N*-cyclopropyl-*N*-(4-((2-(2-(2-formylphenoxy)­acetyl)-1,2,3,4-tetrahydroisoquinolin-6-yl)­carbamoyl)­benzyl)-3-oxo-3,4-dihydro-2*H*-benzo­[*b*]­[1,4]­oxazine-7-carboxamide (**T20**)

Yield: 13.0 mg (49%). ^1^H NMR (400 MHz, DMSO): δ 10.89 (s, 1H), 10.47 (d, *J* = 8.2 Hz, 1H), 10.20 (d, *J* = 6.5 Hz, 1H), 7.94 (d, *J* = 7.9 Hz, 2H), 7.74–7.52 (m, 4H), 7.45 (d, *J* = 7.9 Hz, 2H), 7.24–7.12 (m, 4H), 7.08 (dd, *J* = 7.5, 7.5 Hz, 1H), 6.93 (d, *J* = 8.0 Hz, 1H), 5.18 (s, 2H), 4.75–4.65 (m, 3H), 4.64–4.56 (m, 3H), 3.75–3.66 (m, 2H), 2.92 (t, *J* = 5.9 Hz, 1H), 2.84–2.76 (m, 2H), 0.55 (d, *J* = 7.0 Hz, 2H), 0.51–0.40 (m, 2H). ^13^C NMR (101 MHz, DMSO): δ 189.4, 165.9, 165.2, 164.8, 160.8, 157.4, 142.5, 142.1, 137.6, 136.1, 133.6, 131.8, 128.4, 127.9, 127.5, 127.2, 126.6, 124.5, 121.9, 121.0, 120.0, 118.6, 115.3, 115.2, 114.2, 66.7, 66.3, 48.6, 44.9, 43.4, 41.6, 28.8, 9.5. HRMS (ESI) calcd for C_38_H_34_N_4_NaO_7_^+^ [M + Na]^+^ *m*/*z* 681.2320; found, 681.2300.

#### 4-Formylbenzyl 6-(4-((*N*-cyclopropyl-3-oxo-3,4-dihydro-2*H*-benzo­[*b*]­[1,4]­oxazine-7-carboxamido)­methyl)­benzamido)-3,4-dihydroisoquinoline-2­(1*H*)-carboxylate (**T21**)

Yield: 6.0 mg (15%). ^1^H NMR (500 MHz, DMSO): δ 10.87 (s, 1H), 10.18 (s, 1H), 10.01 (s, 1H), 7.93 (dd, *J* = 8.0, 5.7 Hz, 4H), 7.65 (s, 1H), 7.60 (d, *J* = 7.9 Hz, 2H), 7.58–7.52 (m, 1H), 7.44 (d, *J* = 8.0 Hz, 2H), 7.18 (dd, *J* = 8.9, 3.3 Hz, 2H), 7.14 (d, *J* = 1.8 Hz, 1H), 6.93 (d, *J* = 8.0 Hz, 1H), 5.24 (s, 2H), 4.71 (br s, 2H), 4.66–4.59 (m, 3H), 4.57–4.50 (m, 1H), 3.76–3.56 (m, 2H), 2.89–2.73 (m, 3H), 0.62–0.37 (m, 4H). ^13^C NMR (101 MHz, DMSO): δ 192.8, 170.6, 167.1, 165.2, 164.8, 154.5, 143.9, 142.5, 142.1, 137.5, 135.6, 134.6, 133.6, 131.8, 129.7, 128.4, 127.9, 127.7, 127.2, 126.5, 121.9, 120.1, 118.5, 115.3, 115.1, 66.7, 65.6, 63.1, 46.3, 45.1, 30.4, 20.9, 17.3, 9.5. HRMS (ESI) calcd for C_38_H_34_N_4_NaO_7_^+^ [M + Na]^+^ *m*/*z* 681.2320; found, 681.2309.

#### *N*-cyclopropyl-*N*-(4-((2-(4-formylbenzoyl)-1,2,3,4-tetrahydroisoquinolin-6-yl)­carbamoyl)­benzyl)-3-oxo-3,4-dihydro-2*H*-benzo­[*b*]­[1,4]­oxazine-7-carboxamide (**T22**)

Yield: 21.0 mg (33%). ^1^H NMR (400 MHz, CDCl_3_): δ 10.04 (s, 1H), 9.37 (s, 1H), 8.67 (s, 1H), 7.93 (d, *J* = 7.6 Hz, 2H), 7.84 (d, *J* = 8.0 Hz, 2H), 7.74–7.52 (m, 3H), 7.47–7.28 (m, 3H), 7.16–7.00 (m, 3H), 6.80 (d, *J* = 8.2 Hz, 1H), 4.84 (s, 1H), 4.74 (s, 2H), 4.57 (s, 2H), 4.48 (s, 1H), 4.07–3.88 (m, 1H), 3.67–3.52 (m, 1H), 3.00–2.88 (m, 1H), 2.88–2.75 (m, 1H), 2.69–2.57 (m, 1H), 0.60 (d, *J* = 6.9 Hz, 2H), 0.52–0.41 (m, 2H) ^13^C NMR (101 MHz, DMSO): δ 192.8, 165.2, 164.9, 142.5, 142.1, 141.7, 137.5, 136.6, 134.4, 133.6, 131.8, 129.7, 128.4, 127.9, 127.5, 127.2, 126.8, 121.9, 120.2, 118.7, 115.4, 115.2, 66.7, 54.2, 44.6, 43.8, 29.0, 9.5. HRMS (ESI) calcd for C_37_H_32_N_4_NaO_6_^+^ [M + Na]+ *m*/*z* 651.2214; found, 651.2208.

#### *N*-cyclopropyl-*N*-(4-((2-(3-formylbenzoyl)-1,2,3,4-tetrahydroisoquinolin-6-yl)­carbamoyl)­benzyl)-3-oxo-3,4-dihydro-2*H*-benzo­[*b*]­[1,4]­oxazine-7-carboxamide (**T23**)

Yield: 32.0 mg (51%). ^1^H NMR (400 MHz, DMSO): δ 10.88 (s, 1H), 10.20 (s, 1H), 10.07 (s, 1H), 8.06–7.97 (m, 2H), 7.94 (d, *J* = 7.9 Hz, 2H), 7.80 (s, 1H), 7.72 (d, *J* = 7.6 Hz, 1H), 7.70–7.65 (m, 1H), 7.64–7.54 (m, 1H), 7.44 (d, *J* = 7.9 Hz, 2H), 7.25 (s, 1H), 7.19 (dd, *J* = 8.0, 1.8 Hz, 1H), 7.14 (d, *J* = 1.8 Hz, 1H), 6.93 (d, *J* = 8.1 Hz, 1H), 4.81–4.68 (m, 3H), 4.62 (s, 2H), 4.60–4.47 (m, 1H), 4.00–3.78 (m, 1H), 3.65–3.51 (m, 1H), 2.96–2.72 (m, 3H), 0.59–0.41 (m, 4H). ^13^C NMR (101 MHz, CDCl_3_): δ 191.6, 165.9, 165.5, 162.7, 143.1, 141.8, 137.0, 136.5, 134.1, 132.8, 132.4, 131.1, 129.6, 128.1, 127.9, 122.2, 120.8, 119.3, 116.0, 115.8, 67.2, 36.6, 31.6, 29.8, 10.2. HRMS (ESI) calcd for C_37_H_32_N_4_NaO_6_^+^ [M + Na]+ *m*/*z* 651.2214; found, 651.2205.

#### *N*-cyclopropyl-*N*-(4-((2-(4-formylbenzyl)-1,2,3,4-tetrahydroisoquinolin-6-yl)­carbamoyl)­benzyl)-3-oxo-3,4-dihydro-2*H*-benzo­[*b*]­[1,4]­oxazine-7-carboxamide (**T24**)

Yield: 10.0 mg (41%). ^1^H NMR (400 MHz, DMSO): δ 10.88 (s, 1H), 10.12 (s, 1H), 10.00 (s, 1H), 7.97–7.85 (m, 4H), 7.66–7.55 (m, 3H), 7.51–7.39 (m, 3H), 7.18 (d, *J* = 8.3 Hz, 1H), 7.14 (d, *J* = 1.7 Hz, 1H), 6.98 (d, *J* = 8.4 Hz, 1H), 6.93 (d, *J* = 8.0 Hz, 1H), 4.71 (s, 2H), 4.61 (s, 2H), 3.76 (s, 2H), 3.54 (s, 2H), 2.91–2.75 (m, 3H), 2.74–2.65 (m, 2H), 0.54 (d, *J* = 6.9 Hz, 2H), 0.51–0.40 (m, 2H). ^13^C NMR (101 MHz, DMSO): δ 192.8, 165.2, 164.9, 145.9, 142.5, 142.0, 137.2, 135.3, 134.2, 133.7, 131.8, 130.0, 129.6, 129.3, 128.4, 127.9, 127.2, 126.5, 123.5, 121.9, 120.1, 118.1, 115.4, 115.2, 110.4, 66.7, 61.4, 55.1, 50.3, 28.9, 9.5. HRMS (ESI) calcd for C_37_H_35_N_4_O_5_^+^ [M + H]+ *m*/*z* 615.2602; found, 615.2591.

#### *N*-cyclopropyl-*N*-(4-((2-(3-formylbenzyl)-1,2,3,4-tetrahydroisoquinolin-6-yl)­carbamoyl)­benzyl)-3-oxo-3,4-dihydro-2*H*-benzo­[*b*]­[1,4]­oxazine-7-carboxamide (**T25**)

Yield: 22.0 mg (60%). ^1^H NMR (400 MHz, DMSO): δ 10.88 (s, 1H), 10.12 (s, 1H), 10.03 (s, 1H), 8.00–7.88 (m, 3H), 7.83 (d, *J* = 7.6 Hz, 1H), 7.71 (d, *J* = 7.6 Hz, 1H), 7.64–7.52 (m, 2H), 7.52–7.36 (m, 3H), 7.18 (d, *J* = 8.0 Hz, 1H), 7.14 (s, 1H), 6.98 (d, *J* = 8.4 Hz, 1H), 6.93 (d, *J* = 8.1 Hz, 1H), 4.71 (s, 2H), 4.61 (s, 2H), 3.75 (s, 2H), 3.54 (s, 2H), 2.86–2.75 (m, 3H), 2.71 (t, *J* = 5.9 Hz, 2H), 0.54 (d, *J* = 6.7 Hz, 2H), 0.51–0.40 (m, 2H). ^13^C NMR (101 MHz, DMSO): δ 193.3, 165.1, 164.9, 142.5, 142.0, 139.8, 137.2, 136.3, 134.9, 134.2, 133.7, 131.8, 130.0, 129.5, 129.2, 128.4, 128.4, 127.9, 127.2, 126.5, 121.9, 120.1, 118.0, 115.4, 115.2, 66.7, 61.1, 55.1, 50.2, 28.9, 9.6. HRMS (ESI) calcd for C_37_H_35_N_4_O_5_^+^ [M + H]+ *m*/*z* 615.2602; found, 615.2589.

#### *N*-cyclopropyl-*N*-(4-((2-(2-formylbenzyl)-1,2,3,4-tetrahydroisoquinolin-6-yl)­carbamoyl)­benzyl)-3-oxo-3,4-dihydro-2*H*-benzo­[*b*]­[1,4]­oxazine-7-carboxamide (**T26**)

Yield: 7.0 mg (23%). ^1^H NMR (400 MHz, DMSO): δ 10.88 (s, 1H), 10.39 (s, 1H), 10.11 (s, 1H), 7.98–7.88 (m, 2H), 7.81 (dd, *J* = 7.7, 1.5 Hz, 1H), 7.63 (td, *J* = 7.5, 1.5 Hz, 1H), 7.59–7.39 (m, 6H), 7.22–7.11 (m, 2H), 6.97 (d, *J* = 8.4 Hz, 1H), 6.92 (d, *J* = 8.1 Hz, 1H), 4.70 (s, 2H), 4.61 (s, 2H), 4.01 (s, 2H), 3.57 (s, 2H), 2.84–2.63 (m, 5H), 0.54 (d, *J* = 7.0 Hz, 2H), 0.51–0.40 (m, 2H). ^13^C NMR (101 MHz, DMSO): δ 192.3, 165.1, 164.8, 163.5, 142.5, 142.0, 141.2, 137.2, 134.8, 134.2, 133.7, 133.4, 130.6, 129.9, 128.4, 128.35, 127.9, 127.2, 126.5, 121.9, 120.1, 118.0, 115.4, 115.2, 66.7, 58.4, 54.8, 49.7, 28.9, 9.5. HRMS (ESI) calcd for C_37_H_35_N_4_O_5_^+^ [M + H]+ *m*/*z* 615.2602; found, ; found, 615.2584.

#### (E)-4-(3-oxoprop-1-en-1-yl)­benzoic Acid (**SI**-**8**)

To the mixture of methyl 4-iodobenzoate (203.0 mg, 0.77 mmol, 1.0 equiv), Pd­(OAc)_2_ (54.0 mg, 0.24 mmol, 0.3 equiv), [*n*-Bu_4_N]­OAc (460.0 mg, 1.53 mmol, 2.0 equiv), KCl (64.0 mg, 0.86 mmol, 1.1 equiv), and K_2_CO_3_ (220.0 mg, 1.59 mmol, 2.1 equiv) was added DMF (7.0 mL) and acrolein diethyl acetal (0.17 mL, 1.12 mmol, 1.4 equiv) under argon. The reaction mixture was stirred at 90 °C overnight. The mixture was then filtered through a short pad of Celite and the filtrate was concentrated in vacuo. The residue was purified by column chromatography with EtOAc/Hexane as the eluent to afford the desired intermediate. To the solution of intermediate in THF/H_2_O (2 mL/2 mL) was added LiOH (70 mg, 2.92 mmol, 3.8 equiv) and the reaction mixture was stirred at room temperature overnight. The mixture was then acidified with AcOH to adjust the pH to 4 and extracted with dichloromethane. The organic layer was dried over Na_2_SO_4_ and concentrated in vacuo to afford **SI**-**8** as yellow solid. (121 mg, 0.69 mmol, 89%). ^1^H NMR (400 MHz, DMSO): δ 13.05 (s, 1H), 9.72 (d, *J* = 7.7 Hz, 1H), 8.00 (d, *J* = 8.2 Hz, 2H), 7.86 (d, *J* = 8.0 Hz, 2H), 7.80 (d, *J* = 15.9 Hz, 1H), 6.94 (dd, *J* = 16.0, 7.7 Hz, 1H). ^13^C NMR (101 MHz, DMSO): δ 194.5, 166.8, 151.6, 138.1, 132.7, 130.3, 129.9, 128.8. LC–MS (ESI) calcd for C_10_H_7_O_3_^−^ [M- H]^−^ *m*/*z* 175.1; found, 175.1.

#### (E)-*N*-Cyclopropyl-3-oxo-*N*-(4-((2-(4-(3-oxoprop-1-en-1-yl)­benzoyl)-1,2,3,4-tetrahydroisoquinolin-6-yl)­carbamoyl)­benzyl)-3,4-dihydro-2*H*-benzo­[*b*]­[1,4]­oxazine-7-Carboxamide (**T27**)

Yield: 8.5 mg (22%). ^1^H NMR (400 MHz, DMSO): δ 10.88 (s, 1H), 10.19 (s, 1H), 9.71 (d, *J* = 7.7 Hz, 1H), 7.94 (d, *J* = 7.9 Hz, 2H), 7.89–7.74 (m, 3H), 7.66 (d, *J* = 2.1 Hz, 1H), 7.63–7.49 (m, 3H), 7.44 (d, *J* = 8.0 Hz, 2H), 7.32–7.08 (m, 3H), 7.01–6.87 (m, 2H), 4.79–4.67 (m, 3H), 4.61 (s, 2H), 4.58–4.46 (m, 1H), 3.87 (s, 1H), 3.61–3.49 (m, 1H), 2.94–2.72 (m, 3H), 0.64–0.37 (m, 4H). ^13^C NMR (101 MHz, DMSO): δ 194.5, 165.3, 164.9, 152.2, 142.5, 142.1, 133.6, 131.8, 129.4, 128.9, 128.4, 128.0, 127.5, 127.2, 121.9, 120.2, 118.7, 115.4, 115.2, 66.7, 43.9, 29.2, 9.5. HRMS (ESI) calcd for C_39_H_34_N_4_NaO_6_^+^ [M + Na]+ *m*/*z* 677.2371; found, 677.2354.

#### (E)-*N*-cyclopropyl-*N*-(4-((2-(3-(4-formylphenyl)­acryloyl)-1,2,3,4-tetrahydroisoquinolin-6-yl)­carbamoyl)­benzyl)-3-oxo-3,4-dihydro-2*H*-benzo­[*b*]­[1,4]­oxazine-7-carboxamide (**T28**)

Yield: 14.0 mg (43%). ^1^H NMR (400 MHz, DMSO): δ 10.89 (s, 1H), 10.20 (d, *J* = 3.5 Hz, 1H), 10.03 (s, 1H), 8.10–7.88 (m, 6H), 7.70–7.51 (m, 4H), 7.44 (d, *J* = 7.9 Hz, 2H), 7.25–7.16 (m, 2H), 7.14 (d, *J* = 1.8 Hz, 1H), 6.93 (d, *J* = 8.1 Hz, 1H), 4.92 (s, 1H), 4.80–4.66 (m, 3H), 4.61 (s, 2H), 3.97 (t, *J* = 5.9 Hz, 1H), 3.80 (d, *J* = 6.6 Hz, 1H), 2.93 (d, *J* = 6.0 Hz, 1H), 2.87–2.73 (m, 2H), 0.69–0.35 (m, 4H). ^13^C NMR (101 MHz, DMSO): δ 192.6, 170.6, 165.2, 164.8, 164.5, 142.5, 142.1, 140.9, 140.1, 136.4, 133.6, 131.8, 129.8, 128.7, 128.4, 127.9, 127.2, 126.4, 121.9, 120.1, 115.3, 115.2, 66.7, 55.0, 50.4, 44.1, 42.8, 29.5, 28.2, 9.5. HRMS (ESI) calcd for C_39_H_34_N_4_NaO_6_^+^ [M + Na]^+^ *m*/*z* 677.2371; found, 677.2372.

#### *N*-cyclopropyl-*N*-(4-((2-(2-(4-formyl-3-methoxyphenyl)­acetyl)-1,2,3,4-tetrahydroisoquinolin-6-yl)­carbamoyl)­benzyl)-3-oxo-3,4-dihydro-2*H*-benzo­[*b*]­[1,4]­oxazine-7-carboxamide (**T29**)

Yield: 10.8 mg (32%). ^1^H NMR (400 MHz, DMSO): δ 10.90 (s, 1H), 10.31 (d, *J* = 8.3 Hz, 1H), 10.20 (d, *J* = 5.3 Hz, 1H), 7.94 (d, *J* = 7.9 Hz, 2H), 7.70–7.52 (m, 3H), 7.45 (d, *J* = 7.8 Hz, 2H), 7.23–7.07 (m, 4H), 6.99–6.90 (m, 2H), 4.82–4.66 (m, 3H), 4.65–4.55 (m, 3H), 4.02–3.82 (m, 5H), 3.79–3.68 (m, 2H), 2.86–2.74 (m, 3H), 0.64–0.43 (m, 4H). ^13^C NMR (101 MHz, DMSO): δ 188.7, 168.5, 165.2, 164.8, 161.4, 145.4, 142.5, 142.1, 137.5, 134.9, 134.6, 133.6, 131.8, 128.8, 128.4, 127.9, 127.7, 127.2, 122.6, 121.9, 121.8, 120.1, 120.0, 118.5, 115.3, 115.2, 113.6, 68.6, 66.7, 55.9, 45.2, 43.5, 43.0, 29.0, 9.5. Rotamers were confirmed in NMR spectra. HRMS (ESI) calcd for C_39_H_36_N_4_NaO_7_^+^ [M + Na]^+^ *m*/*z* 695.2476; found, 695.2461.

#### *N*-cyclopropyl-*N*-(4-((2-(2-(4-formyl-3-hydroxyphenyl)­acetyl)-1,2,3,4-tetrahydroisoquinolin-6-yl)­carbamoyl)­benzyl)-3-oxo-3,4-dihydro-2*H*-benzo­[*b*]­[1,4]­oxazine-7-carboxamide (**T30**)

Yield: 1.4 mg (9%). ^1^H NMR (400 MHz, DMSO): δ 10.89 (s, 1H), 10.25–10.10 (m, 2H), 7.94 (d, *J* = 8.2 Hz, 2H), 7.78 (q, *J* = 7.7 Hz, 1H), 7.69–7.62 (m, 1H), 7.62–7.51 (m, 1H), 7.44 (d, *J* = 8.1 Hz, 2H), 7.35–7.24 (m, 2H), 7.22–7.11 (m, 3H), 6.93 (d, *J* = 8.1 Hz, 1H), 4.80–4.67 (m, 3H), 4.66–4.53 (m, 3H), 4.02–3.92 (m, 2H), 3.76 (t, *J* = 6.0 Hz, 1H), 3.70 (t, *J* = 5.7 Hz, 1H), 2.89–2.73 (m, 3H), 0.62–0.41 (m, 4H). ^13^C NMR (126 MHz, DMSO): δ 191.4, 173.2, 168.6, 165.3, 164.9, 160.6, 144.9, 142.5, 142.1, 137.5, 135.0, 134.7, 133.7, 131.9, 129.3, 128.8, 128.4, 128.0, 127.2, 126.7, 121.9, 120.1, 118.6, 118.5, 115.4, 115.2, 91.5, 66.8, 46.6, 43.6, 43.1, 29.0, 28.3, 9.5. Rotamers were confirmed in NMR spectra. HRMS (ESI) calcd for C_38_H_35_N_4_O_7_^+^ [M + H]^+^ *m*/*z* 659.2500; found, 659.2482.

#### *N*-cyclopropyl-*N*-(4-((2-(2-(4-formyl-3-methylphenyl)­acetyl)-1,2,3,4-tetrahydroisoquinolin-6-yl)­carbamoyl)­benzyl)-3-oxo-3,4-dihydro-2*H*- benzo­[*b*]­[1,4]­oxazine-7-Carboxamide (**T31**)

Yield: 10.0 mg (38%). ^1^H NMR (400 MHz, DMSO): δ 10.92 (s, 1H), 10.28–10.14 (m, 2H), 7.94 (d, *J* = 7.9 Hz, 2H), 7.76 (t, *J* = 8.6 Hz, 1H), 7.64 (d, *J* = 6.3 Hz, 1H), 7.61–7.51 (m, 1H), 7.44 (d, *J* = 7.9 Hz, 2H), 7.33–7.26 (m, 1H), 7.25–7.10 (m, 4H), 6.94 (d, *J* = 8.1 Hz, 1H), 4.83–4.66 (m, 3H), 4.64–4.67 (m, 3H), 3.88 (s, 2H), 3.74 (t, *J* = 6.1 Hz, 1H), 3.70 (t, *J* = 6.0 Hz, 1H), 2.85–2.73 (m, 3H), 2.62–2.55 (m, 3H), 0.54 (d, *J* = 7.0 Hz, 2H), 0.51–0.40 (m, 2H). ^13^C NMR (101 MHz, DMSO): δ 192.9, 168.6, 165.2, 164.9, 142.5, 142.3, 142.26, 142.1, 140.0, 134.6, 133.6, 132.6, 132.4, 131.8, 131.5, 128.4, 127.9, 127.4, 127.2, 126.6, 121.9, 120.0, 118.6, 115.4, 115.2, 66.7, 43.5, 43.0, 29.0, 19.0, 9.54. HRMS (ESI) calcd for C_39_H_37_N_4_O_6_^+^ [M + H]+ *m*/*z* 657.2708; found, 657.2704.

#### *N*-cyclopropyl-*N*-(4-((2-(2-(3-fluoro-4-formylphenyl)­acetyl)-1,2,3,4-tetrahydroisoquinolin-6-yl)­carbamoyl)­benzyl)-3-oxo-3,4-dihydro-2*H*-benzo­[*b*]­[1,4]­oxazine-7-carboxamide (**T32**)

Yield: 5.5 mg (10%). ^1^H NMR (400 MHz, DMSO): δ 10.88 (s, 1H), 10.19 (d, *J* = 5.0 Hz, 1H), 10.17 (s, 1H), 7.94 (d, *J* = 8.2 Hz, 2H), 7.63 (s, 1H), 7.61–7.52 (m, 2H), 7.44 (d, *J* = 7.8 Hz, 2H), 7.23–7.12 (m, 3H), 6.93 (d, *J* = 8.1 Hz, 1H), 6.88 (s, 1H), 6.82 (d, *J* = 7.7 Hz, 1H), 4.76–4.65 (m, 3H), 4.64–4.56 (m, 3H), 3.82 (s, 2H), 3.71 (q, *J* = 6.0 Hz, 2H), 2.85–2.72 (m, 3H), 0.60–0.43 (m, 4H). ^13^C NMR (126 MHz, DMSO): δ 187.6, 170.7, 168.2, 165.3, 164.9, 162.1, 142.5, 142.1, 137.6, 137.5, 135.0, 134.7, 133.7, 131.8, 129.2, 128.8, 128.4, 128.0, 127.2, 126.7, 121.9, 120.1, 120.1, 118.6, 118.5, 115.4, 115.2, 66.8, 46.5, 43.6, 43.0, 29.0, 28.2, 9.6. Rotamers were confirmed in NMR spectra. HRMS (ESI) calcd for C_38_H_34_FN_4_O_6_^+^ [M + H]^+^ *m*/*z* 661.2457; found, 661.2431.

#### *N*-cyclopropyl-*N*-(4-((2-(2-(2-fluoro-4-formylphenyl)­acetyl)-1,2,3,4-tetrahydroisoquinolin-6-yl)­carbamoyl)­benzyl)-3-oxo-3,4-dihydro-2*H*-benzo­[*b*]­[1,4]­oxazine-7-carboxamide (**T33**)

Yield: 2.6 mg (5%). ^1^H NMR (400 MHz, DMSO) δ 10.89 (s, 1H), 10.20 (d, *J* = 6.5 Hz, 1H), 9.98 (d, *J* = 1.8 Hz, 1H), 7.98–7.90 (m, 2H), 7.76–7.69 (m, 1H), 7.70–7.63 (m, 2H), 7.63–7.50 (m, 2H), 7.45 (d, *J* = 8.1 Hz, 2H), 7.22–7.12 (m, 3H), 6.93 (d, *J* = 8.1 Hz, 1H), 4.80–4.68 (m, 3H), 4.64–4.59 (m, 3H), 3.98 (s, 2H), 3.80 (t, *J* = 6.0 Hz, 1H), 3.70 (t, *J* = 5.9 Hz, 1H), 2.96–2.87 (m, 1H), 2.85–2.75 (m, 2H), 0.61–0.42 (m, 4H). ^13^C NMR (126 MHz, DMSO): δ 192.0, 170.7, 167.6, 165.3, 164.9, 162.3, 162.1, 160.1, 142.6, 142.1, 137.7, 137.6, 135.1, 134.8, 133.7, 131.9, 128.9, 128.4, 128.0, 127.2, 126.7, 126.5, 125.6, 121.9, 120.2, 120.1, 118.6, 118.6, 115.4, 115.2, 115.0, 114.8, 66.8, 55.0, 48.7, 46.3, 43.6, 42.9, 34.1, 33.8, 30.8, 29.0, 28.3, 9.5. Rotamers were confirmed in NMR spectra. HRMS (ESI) calcd for C_38_H_34_FN_4_O_6_^+^ [M + H]^+^ *m*/*z* 661.2457; found, 661.2470.

### (*R*)-3-(3-((5-chloro-4-((2-(isopropylsulfonyl)­phenyl)­amino)­pyrimidin-2-yl)­amino)­piperidin-1-yl)-*N*-(2-(2-(4-formylphenyl)­acetyl)-1,2,3,4-tetrahydroisoquinolin-6-yl)­propenamide (**T34**)

A solution of 2,5-dichloro-*N*-(2-(isopropylsulfonyl)­phenyl)­pyrimidin-4-amine (865 mg, 2.5 mmol, 1.0 equiv) in *N*-Methyl-2-pyrrolidone (20 mL) was treated with *tert*-butyl (R)-3-aminopiperidine-1-carboxylate (550 mg, 2.75 mmol, 1.1 equiv) under an ice bath. Subsequently, *N*,*N*-Diisopropylethylamine (1.3 mL, 7.5 mmol, 3.0 equiv) was added. The reaction mixture was then stirred at 110 °C for 12 h. The mixture was concentrated under vacuum. The residue was purified through column chromatography using ethyl acetate/hexane (1:0 to 1:1) as the eluent to afford the desired intermediate, SI-9. To a solution of SI-9 in DCM (10 mL), TFA (5 mL) was added. The mixture was stirred at room temperature for 3 h. After removing the solvent and excess TFA, the residue was treated with aqueous sodium bicarbonate to adjust the pH to approximately 8. Using a DCM/MeOH (9:1) mixture to extract the corresponding amine, SI-10 was obtained (808 mg, 1.98 mmol, 79%, 2 steps) after concentration under vacuum.

To a solution of SI-10 (82 mg, 0.2 mmol, 1.0 equiv) in anhydrous DMF (2.0 mL) was added potassium carbonate (82.8 mg, 0.6 mmol, 3.0 equiv), followed by *tert*-butyl 3-bromopropanoate (50.1 mg, 0.24 mmol, 1.2 equiv). The mixture was stirred at room temperature overnight. After the complete removal of solvent, the residue was purified via silica gel column chromatography (eluting with hexane/ethyl acetate, 1:0 to 1:3) to afford SI-11 (34.5 mg, 0.064 mmol, 32%).

A solution of compound SI-11 (34.5 mg, 0.064 mmol) in DCM (1.0 mL) was treated with TFA (1 mL), and after 3 h of stirring, the mixture was analyzed by LC–MS and then concentrated to dryness, affording the crude product SI-12. This crude compound was then dissolved in anhydrous DMF (2.0 mL), followed by *tert*-butyl 6-amino-3,4-dihydroisoquinoline-2­(1*H*)-carboxylate (19.1 mg, 0.077 mmol, 1.2 equiv), HATU (29.3 mg, 0.077, 1.2 equiv), and DIPEA (16.5 mg, 0.128, 2.0 equiv), the mixture was stirred at room temperature overnight. After concentration, the residue was purified by column chromatography (silica gel, hexane/ethyl acetate, 1:0–0:1) to afford SI-13 (37.1 mg, 0.052 mmol, 81% over two steps).

The compound SI-13 (37.1 mg, 0.052 mmol, 1.0 equiv) was then treated with DCM (1.0 mL) and TFA (1.0 mL), stirred at room temperature for 4 h. After being indicated by LC–MS, the mixture was concentrated for the next steps directly. To a solution of crude compound in DMF (1.0 mL) was treated with 2-(4-formylphenyl)­acetic acid (12.8 mg, 0.078 mmol, 1.5 equiv), EDCI (12.0 mg, 0.062 mmol, 1.2 equiv) and DMAP (12.0 mg, 0.052 mmol, 1.0 equiv). The reaction was stirred at room temperature overnight, and the purification via reversed-phase HPLC (Gradient, 20–60% Acetonitrile in water with 0.1% formic acid) was completed after confirmation by LC–MS, yielding compound T34 (8.7 mg, 0.011 mmol, 22% over two steps). ^1^H NMR (400 MHz, DMSO): δ 9.97 (s, 1H), 9.96 (s, 1H), 9.46 (s, 1H), 9.01–8.45 (m, 1H), 8.19–8.17 (m, 1H), 7.89–7.76 (m, 3H), 7.68 (s, 1H), 7.51–7.39 (m, 3H), 7.36–7.03 (m, 4H), 4.66 (s, 1H), 4.55 (s, 1H), 3.99–3.89 (m, 2H), 3.71 (t, *J* = 5.9 Hz, 1H), 3.66 (t, *J* = 6.0 Hz, 1H)., 3.49–3.39 (m, 3H), 3.03–2.98 (m, 1H), 2.93–2.80 (m, 1H), 2.79–2.65 (m, 4H), 2.30–1.93 (m, 2H), 1.91–1.80 (m, 1H), 1.78–1.66 (m, 1H), 1.60–1.44 (m, 1H), 1.41–1.26 (m, 1H), 1.16 (d, *J* = 6.8 Hz, 6H). ^13^C NMR (101 MHz, DMSO): δ 192.7, 169.8, 168.6, 154.6, 143.2, 138.4, 137.4, 135.0, 134.7, 134.6, 130.9, 130.2, 130.1, 129.5, 129.4, 126.7, 123.0, 122.7, 118.7, 117.3, 57.7, 54.9, 53.7, 52.6, 46.5, 43.5, 42.9, 29.5, 28.9, 14.9. HRMS (ESI) calcd for C_39_H_45_ClN_7_O_5_S^+^ [M + H]^+^ *m*/*z* 758.2886; found, 758.2903.

### Materials and Reagents

Anti-HA tag (RRID: AB_1549585), anti-GAPDH (RRID: AB_1642205), anti-Vinculin (RRID: AB_10559207), antimouse IgG (RRID: AB_330924), and mouse antirabbit IgG (RRID: AB_10892860) antibodies were purchased from Cell Signaling Technology. The anti-NSD2 antibody (RRID: AB_1310816) was purchased from Abcam. Anti-β Actin antibody (RRID/AB_2714189) was obtained from Santa Cruz. The anti-FBXO22 antibody (RRID: AB_2104403) was obtained from Proteintech. FBXO22 siRNA was purchased from Horizon, and Pierce anti-HA magnetic beads were obtained from Thermo Scientific.

### Cell Lines

H358 (RRID: CVCL_1559), RPMI8226 (RRID: CVCL_0014), PC3 (RRID: CVCL_E2RM), and HEK293T (RRID: CVCL_0063) were obtained from ATCC. HEK293T was purchased in March 2021, but the date of purchase of rest of the cell lines are unclear. This will not impact our results and conclusions, since we confirmed that they are contamination free. The HEK 293T FBXO22 knockout (k.o.) cells, along with HEK 293T FBXO22 k.o. Wild-type HA-FBXO22 knock-in and HEK 293T FBXO22 k.o. C326A HA-FBXO22 knock-in cells, were kindly provided by Dr. Xiaoyu Zhang.

293T-NSD2-HiBiT cell line is constructed based on the methods described below: The HiBiT tag was fused to the C-terminus of the NSD2 gene in HEK293T cells using the CRISPR-Cas9 system previously reported. (Nature Protocols, 2013; 8, 2281–2308) In brief, guide RNA oligonucleotides targeting NSD2 were cloned into the Cas9 expression plasmid pSpCas9–2A-GFP (Addgene). The plasmid then was cotransfected with a donor DNA oligonucleotide into HEK293T cells using FuGENE HD Transfection Reagent (Promega). GFP-positive cells were isolated by fluorescence-activated cell sorting (FACS), and single-cell clones were screened for HiBiT signal using the Nano-Glo HiBiT Lytic Detection System (Promega).

Guide RNA oligonucleotides targeting NSD2:
Forward: 5′-CACCGAGAGGGCAAATAGCGCCAGG-3′
Reverse: 5′-AAACCCTGGCGCTATTTGCCCTCTC-3′
Donor DNA sequence: AGAAGCCCCCCCCAGAGCCAGGGAAGCCGAAGGGGAAGAGGCGGCGGCGGAGGGGCTGGCGGAGAGTCACAGAGGGCAAAGGTGTAAGCGGTTGGAGATTGTTCAAGAAAATCTCCTAGAGGCGGCCGCTTGGCCGGATCCAGGGGCGGTGCAGGGCGGCCGGCCCTGCCTGCGGGAGAGGGCGAGCATGAACTG

These cell lines, along with the 293T-NSD2-HiBiT cells, were cultured in DMEM (Corning) supplemented with 10% (v/v) fetal bovine serum (FBS). H358, PC3, and RPMI8226 cells were cultured in RPMI-1640 medium (Corning) supplemented with 10% (v/v) FBS, 10 mM HEPES, and 10 mM sodium pyruvate. All the cell lines are contamination free.

### Western Blot

Cells were harvested after treatment and lysed in RIPA buffer supplemented with cOmplete EDTA-free Protease Inhibitor Cocktail (MilliporeSigma). Lysates were centrifuged, and total protein concentration was determined using a BCA assay (Thermo Scientific). Samples were mixed with Laemmli sample buffer and boiled at 95 °C for 5 min. Proteins were separated on tris-glycine SDS-PAGE gels and transferred to 0.22 μm PVDF membranes (Bio-Rad). Membranes were blocked with 5% nonfat milk in TBS-T and incubated overnight at 4 °C with primary antibodies (1:1000 dilution in 5% BSA in TBS-T). HRP-conjugated secondary antibodies (1:5000 dilution in 1% BSA in TBS-T) were incubated for 1 h at room temperature. Membranes were developed using ECL Prime Western Blotting Detection Reagent (Bio-Rad) and imaged on ChemiDoc MP Imaging System.

### HiBiT Screening

293T-NSD2-HiBiT cells were seeded into white, clear-bottom 96-well plates and treated with DMSO or test compounds once the cells reached ∼90% confluency. Following 4–24 h of treatment, NSD2-HiBiT signal was measured using the Nano-Glo HiBiT Lytic Assay (Promega), according to the manufacturer’s instructions.

### Proteomic Analysis

#### Sample Preparation

H358 cells were seeded into 6-well plates and treated with DMSO or test compounds once the cells reached ∼80–90% confluency. Following 4 h of treatment. Cells were collected with scraping and lysed in lysis buffer (50 mM HEPPS, pH 7.4, 4 M Urea, 0.5% SDS, and 1 U/uL benzonase, EDTA-free protease inhibitor) for 30 min at room temperature. Protein concentration was measured by BCA. Lysate containing ∼10 μg protein was performed SP3-based cleanup as previously described.^50,51^ Briefly describe here, 2 μL SP3 beads (1:1 mixture) and ethanol supplemented with 20 mM dithiothreitol (DTT) was added to lysate for final 50% ethanol and shake for 15 min. Beads were washed once with 80% ethanol, resuspended in 50 mM HEPES (pH 7.4) supplemented with 20 mM iodoacetamide (IAA) and then incubated in dark for 30 min. Ethanol supplemented with 20 mM DTT was added to lysate for final 50% ethanol followed by shaking for 15 min. The beads were then washed 3 times with 80% ethanol. Addition of 0.1 μg Lys-C and 0.1 μg trypsin in 200 mM EPPS (pH 8.5) was followed by overnight incubation at 37 °C. Digested peptides were resuspended in 150 μL 1% FA and desalted with StageTip.

#### LC–MS based DIA Analysis

Resulted peptides were resuspended in LC–MS loading buffer (1% ACN and 1% FA) and loaded on a 100 μm capillary column packed with 30 cm of Accucore 150 resin (2.6 μm, 150 Å; Thermo Fisher Scientific). Peptides were separated using a 90 min method on a NanoLC-1200 UPLC system. Data was acquired by using a data-independent acquisition (DIA) method on an Orbitrap Eclipse mass spectrometer coupled with a FAIMS Pro device. Data were collected alternating between a set of two FAIMS compensation voltages (CVs) at −35 and −45. MS1 scans were collected in the Orbitrap with a resolution setting of 120 K, a mass range of 400–1000 *m*/*z*, standard AGC target and maximum injection time at auto mode. MS2 scans were acquired in loop control mode with time of 3 s. Peptide precursors were isolated at a window of 10 *m*/*z* for total 60 windows and fragmented using HCD with a collision energy of 30. MS2 scans were collected in the Orbitrap with a resolution of 15K, scan range 145–1450, and a 800% AGC with a maximum injection time of 25 ms.

#### Data Analysis

Raw files were searched using standard DIA_SpecLib_Quant workflow in FragPipe (V23.1) with the Uniprot human proteome (UP000005640) appended with contaminants and reverse decoy sequences.^52–54^ Precursor error tolerance was 20 ppm and fragment error tolerance was 20 ppm of Static modifications include carboxyamidomethylation (+57.0215) on Cys side chains. Methionine oxidation (+15.9949) and *N*-terminal acetylation (+42.0106) were allowed as variable modifications. Protein was restricted to 1% false discovery rate (FDR) and quantified by DIA-IN (1.8.2 beta 8). Proteome-wide differential analysis was performed by LIMMA package.^55^ All RAW files were uploaded to MassIVE database with accession number MSV000100336 and can accessed with link: ftp://MSV000100336@massive-ftp.ucsd.edu.

### Mutagenesis

Human FBXO22 cDNA with an *N*-terminal StrepII × 2-Flag × 2 tag (RRID: Addgene_159127) was obtained from Addgene. The FBXO22 C326A mutant was generated using the Q5 Site-Directed Mutagenesis Kit (New England Biolabs) following the manufacturer’s protocol.

### Co-Immunoprecipitation

293T HA-FBXO22 cells were seeded in 10 cm dishes and grown to 70–80% confluency. Cells were pretreated with MLN4294 for 1 h, followed by cotreatment with degraders for 4 h. After treatment, cells were washed three times with ice-cold PBS and lysed for 20 min in RIPA buffer supplemented with cOmplete EDTA-free Protease Inhibitor Cocktail (MilliporeSigma). Lysates were clarified by centrifugation at 16,000 rcf for 15 min, and protein concentration was measured using the BCA assay (Thermo Scientific). For input samples, 30 μg of protein was mixed with 4× LDS sample buffer and boiled at 95 °C for 5 min.

For immunoprecipitation, 20 μL of Anti-HA magnetic beads (Thermo Fisher Scientific) were washed three times with TBS. 1 mg of protein lysate was adjusted to 200 μL and incubated with the beads overnight at 4 °C on a rotator. The next day, beads were washed three times with TBS-T and eluted by boiling in 2× LDS sample buffer at 95 °C for 10 min. Samples were analyzed by immunoblotting.

### Cellular NanoBRET Ternary Complex Formation (TCF), Ubiquitination and Proteasomal Recruitment Assays

To evaluate ternary complex formation between NSD2 and FBXO22, HEK293-LgBiT cells (1 × 10̂6) were cultured in Dulbecco’s Modified Eagle Medium supplemented with 10% FBS. Cells were transfected in six-well plates using FuGENE 4K (Promega) with 100 ng HiBiT-NSD2 construct and 3 μg of either Wild Type or Cysteine mutant HaloTag-FBXO22 plasmids. For the assessment of live-cell NSD2 ubiquitination and proteasomal recruitment, HEK293-LgBiT cells (1 × 10̂6) were similarly cultured and transfected with 100 ng HiBiT-NSD2 and 3 μg HaloTag-Ubiquitin or HaloTag-PSMD3 vectors. The following day, transfected cells (2 × 10̂4) were replated triplicate into white 96-well tissue culture plates with 90 μL assay medium (Opti-MEM Reduced Serum Medium, no phenol red +5% FBS), with or without HaloTag NanoBRET 618 Ligand (Promega), and incubated overnight at 37 °C in 5% CO2. To monitor end point ternary complex formation, ubiquitination, and proteasomal recruitment, 10 μL of 10X concentrated drugs were added and incubated for 6 h at 37 °C, 5% CO2. Subsequently, 25 μL of 5X NanoBRET Nano-Glo substrate (Promega) was added, and BRET signal was measured using the VICTOR Nivo Multimode Plate Reader (PerkinElmer, USA). Dual-filtered luminescence signals were collected using a 460/480 nm bandpass filter (donor signal from HiBiT-NSD2 fusion protein) and a 610 nm long pass filter (acceptor signal from HaloTag NanoBRET 618 ligand), with an integration time of 0.5 s. For all NanoBRET assays, background-subtracted NanoBRET ratios were reported in milliBRET units.

## Supplementary Material







## Notes

Molecular formula strings and biological activities of NSD2 and CDK 12 PROTACs (CSV)
Proteomics data including protein quant table and DE analysis (XLSX)
Supporting Information figure includes immunoblotting analysis of NSD2 in PC3, RPMI8226, and H358 cells, as well as the knockdown of FBXO22 by siRNA in PC3 cells; Immunoblotting of NSD2 isoforms across various cell lines; FBXO22 expression levels in different cell lines; NanoBRET analysis of NSD2 recruitment to PSMD3; NanoBRET analysis of NSD2 recruitment to FBXO22 (WT and mutant); Co-IP analysis of NSD2 and HA-FBXO22; Immunoblot of transfected 293T cells upon different FBXO22 mutation types; unadjusted global proteomics volcano plots; and chemical stability of T9 in GSH. HPLC analysis, NMR spectra for compounds reported in this study, and HRMS data of T9 and T34 (PDF)

## Abbreviations

ALL: acute lymphoblastic leukemia
CDI: 1,1′-Carbonyldiimidazole
CRBN: cereblon
DCM: Dichloromethane
DIPEA: *N*,*N*-Diisopropylethylamine
DMAP: 4-(Dimethylamino)­pyridine
DMF: Dimethylformamide
DMP: Dess–Martin periodinane
DMSO: Dimethyl sulfoxide
EDCI: 1-Ethyl-3-(3-(dimethylamino)­propyl)­carbodiimide
FBXO22: the F-box only protein 22
HATU: Hexafluorophosphate Azabenzotriazole Tetramethyl Uronium
HOBT: 1-Hydroxybenzotriazole
NMI: 1-Methylimidazole
NSD2: nuclear receptor-binding SET domain-containing 2
POI: protein of interest
PROTACs: proteolysis-targeting chimeras
TBACl: Tetrabutylammonium chloride
TCF: Ternary Complex Formation
TCFH: Chloro-N,N,N′,N′-tetramethylformamidinium hexafluorophosphate
TEA: Triethylamine
THF: Tetrahydrofuran
TPD: Targeted Protein Degradation
UPS: ubiquitin–proteasome system
VHL: the von Hippel-Lindau protein
