# Supplementary material for: Aryl Aldehyde-Anchored Small Molecules Recruit FBXO22 for Targeted Degradation of NSD2
Source: J Med Chem. 2026 Jun 29;69(13):15317–38. doi: 10.1021/acs.jmedchem.6c00020 (PMC13370883; doi:10.1021/acs.jmedchem.6c00020)
Supplement: Supplementary file 3 [file jm6c00020_si_003.pdf]

Supplementary Information

Aryl Aldehyde-anchored Small Molecules Recruit FBXO22 for Targeted Degradation of NSD2

Hua Tang [a§], Yaxian Liao [b§], Tsung-Yu Yeh [a§], Kazuya Nishibayashi [a], Milad Rouhimoghadam [c], Ka Yang [d], Chunrong Li [a], Regina Stasser de Gonzalez [a], Yuan Zhao [a], Nina J. Hawkins [a], Justin M. Reitsma [c], Steven P. Gygi [d], and Weiping Tang [ab*]

a Lachman Institute for Pharmaceutical Development, School of Pharmacy, University of Wisconsin-Madison, Madison, Wisconsin 53705, United States

b Department of Chemistry, University of Wisconsin-Madison, Madison, Wisconsin 53706, United States

c Technology & Therapeutic Platforms, AbbVie Incorporated, North Chicago, Illinois 60064, United States

d Department of Cell Biology, Harvard Medical School, Boston, Massachusetts 02115, United States

§ H.T., Y.L., and T.Y.Y. contributed equally to this work

[*] Correspondence: E-mail: weiping.tang@wisc.edu; orcid.org/0000-0002-0039-3196.

Table of Contents

Supplementary figure 3

Table S1. Stability of T9 in the presence of 10 mM GSH[α] 8

HPLC analysis of compounds 9

HRMS spectrum of T9 44

HRMS spectrum of T34 44

NMR spectra of degraders 61

Supplementary figure

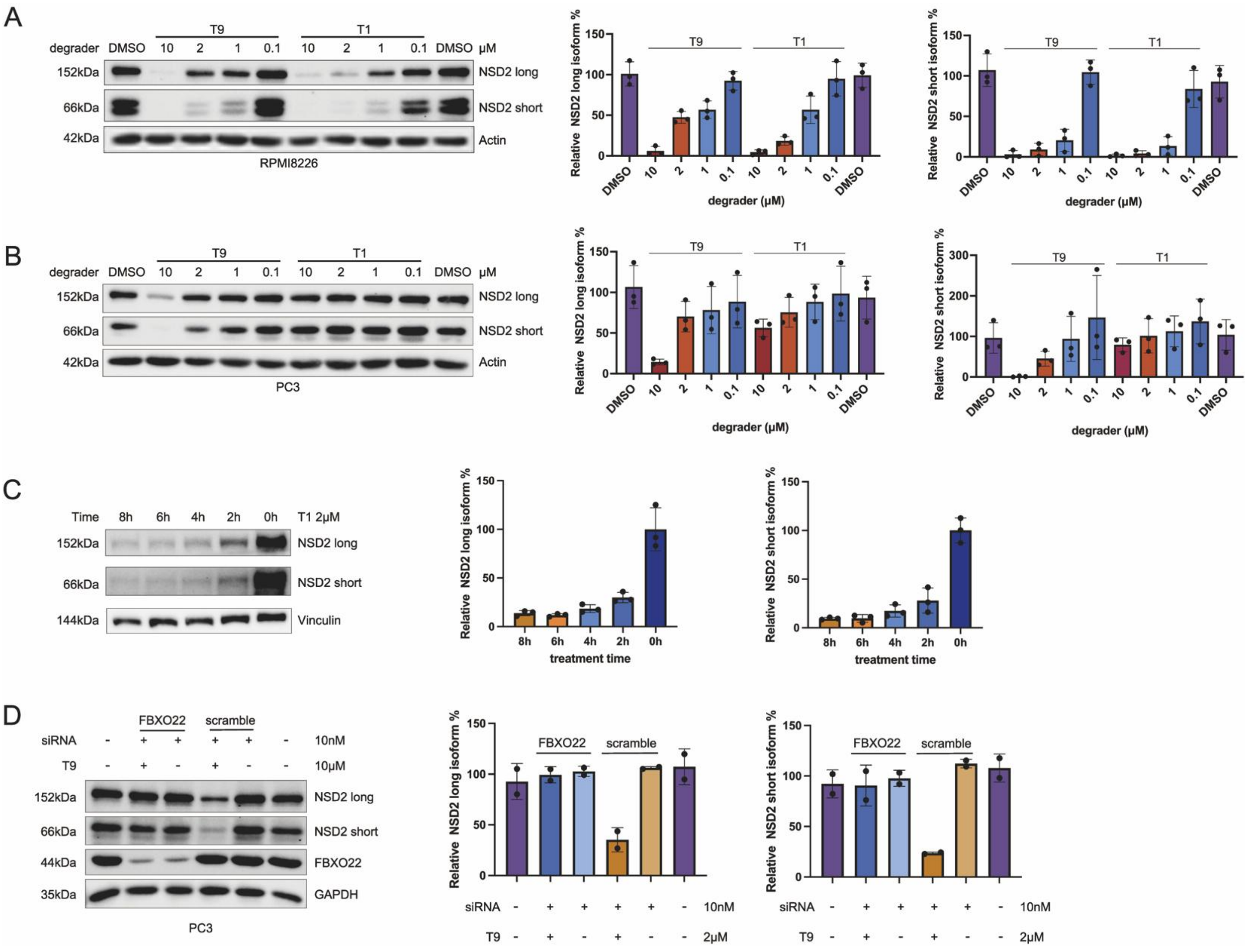

Figure S1. A) Immunoblotting analysis of NSD2 in RPMI8226 cells treated with various concentrations of T1 and T9 for 4 hours. The graph represents quantification of the long and short NSD2 isoforms/Actin protein content (n=3 biological independent samples). B) Immunoblotting analysis of NSD2 in PC3 cells treated with various concentrations of T1 and T9 for 4 hours. The graph represents quantification of the long and short NSD2 isoforms/Actin protein content (n=3 biological independent samples). C) Immunoblotting analysis of NSD2 in H358 cells treated with 2 μM of T1 degrader for 0, 2, 4, 6, 8 h time points. D) Knockdown of FBXO22 by siRNA in PC3 cells. T9 was added 48 h post-knockdown. The graph represents quantification of the long and short NSD2 isoforms/GAPDH protein content (n=2 biological independent samples).

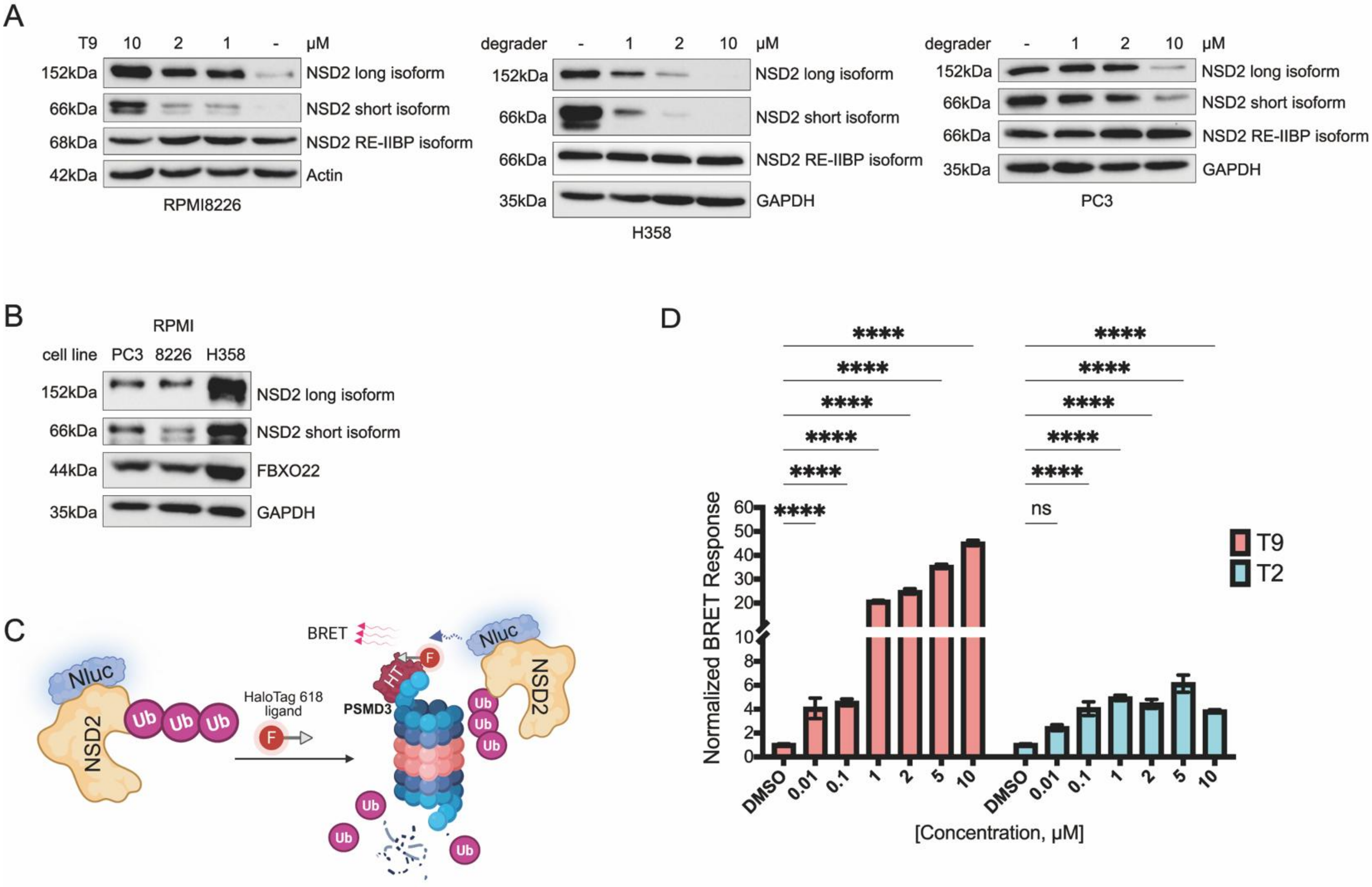

Figure S2. A) Immunoblotting analysis of NSD2 short isoform, long isoform and RE-IIBP isoform level after treatment with varying concentration of T9 for 4h. B) Immunoblotting analysis of NSD2 short and long isoform level and FBXO22 expression level in PC3, RPMI8226, and H358 cells. C) Schematic of NanoBRET mechanism measuring NSD2-PSMD3 protein-protein interaction, created in BioRender. Rouhimoghadam, M. (2026) https://BioRender.com/7s40kpl. D) NanoBRET analysis of NSD2-PSMD3 interaction in 293T cells treated with varying concentrations of T2 and T9 (n=3 biological independent samples, statistical significance was determined by two-way ANOVA; ****p < 0.0001; ns = not significant).

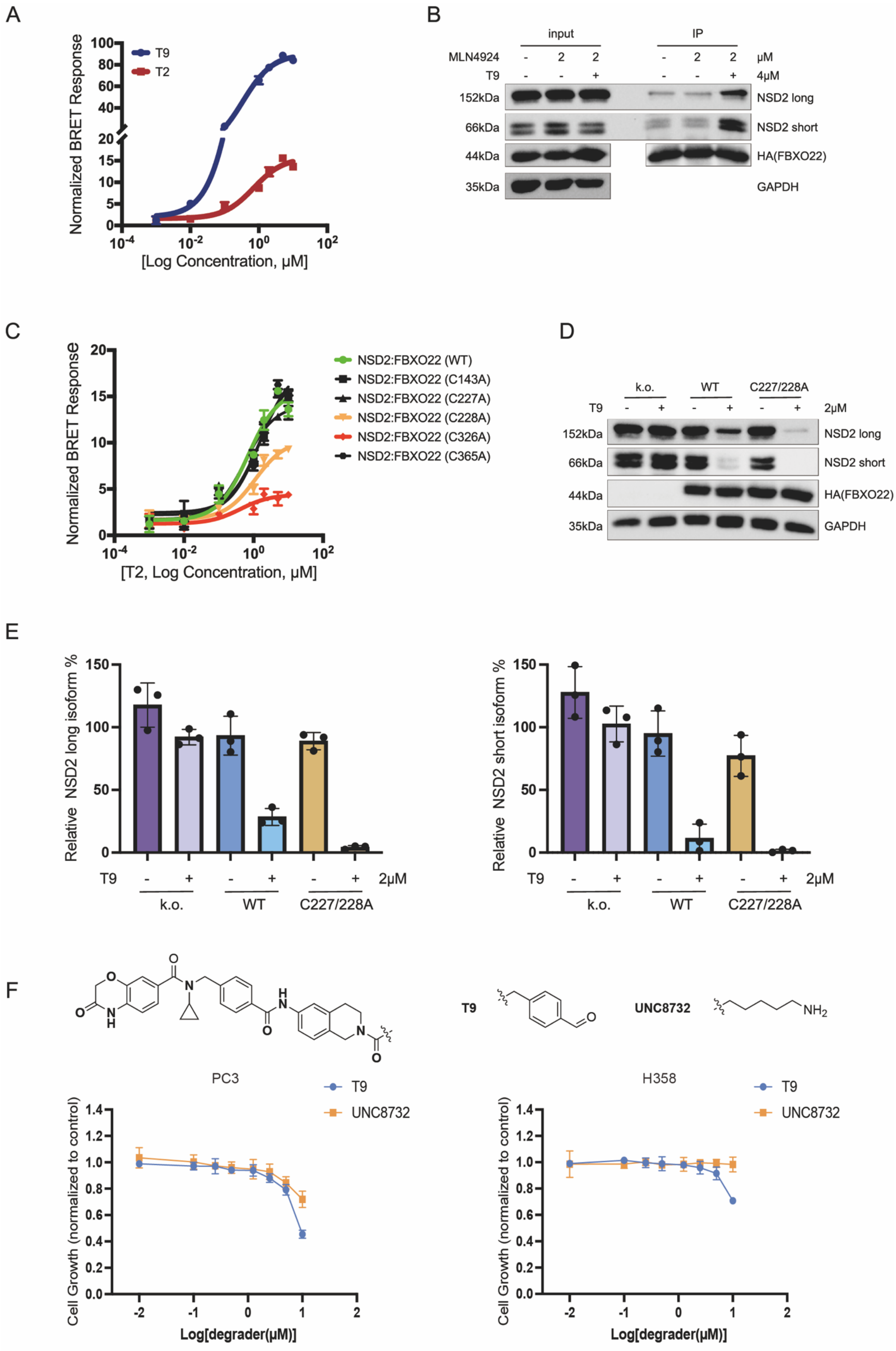
A
Normalized BRET Response
T9
T2
[Log Concentration, μM]
B
input
IP
MLN4924
T9
μM
4μM
152kDa
66kDa
44kDa
35kDa
NSD2 long
NSD2 short
HA(FBXO22)
GAPDH
C
Normalized BRET Response
NSD2:FBXO22 (WT)
NSD2:FBXO22 (C143A)
NSD2:FBXO22 (C227A)
NSD2:FBXO22 (C228A)
NSD2:FBXO22 (C326A)
NSD2:FBXO22 (C365A)
[T2, Log Concentration, μM]
D
k.o.
WT
C227/228A
T9
2μM
152kDa
66kDa
44kDa
35kDa
NSD2 long
NSD2 short
HA(FBXO22)
GAPDH
E
Relative NSD2 long isoform %
Relative NSD2 short isoform %
T9
2μM
k.o.
WT
C227/228A
F
T9
UNC8732
PC3
H358
T9
UNC8732
Cell Growth (normalized to control)
Log[degrader(μM)]

Figure S3. A) NanoBRET analysis of the interaction between NSD2 and wild type FBXO22 in 293T cells treated with varying concentrations of T9 and T2. (n=3 biological independent samples, statistical significance was determined by two-way ANOVA; ****$p < 0.0001$; ns = not significant). B) Co-immunoprecipitation of HA-FBXO22 and NSD2 following the pretreatment of DMSO or MLN4924 for 1h, co-treated with DMSO or T9 for another 4 h. C) NanoBRET analysis of the interaction between NSD2 and FBXO22 cysteine mutants in 293T cells treated with varying concentrations of T2. (n=3 biological independent samples, statistical significance was determined by two-way ANOVA; ****$p < 0.0001$; ns = not significant). D) Immunoblot of transfected 293T cells for different FBXO22 mutation type. FBXO22 k.o. 293T cells were lentivirus-transfected with empty vector (k.o.) wild-type HA-FBXO22 (WT), or double mutant C227/228A HA-FBXO22. E) Graph represents quantification of D) showing the long and short NSD2 isoforms/GAPDH protein content (n=3 biological independent samples). F) 3-day antiproliferative effects of T9 and UNC8732 at indicated concentrations (up to 10 μM) in PC3 (left) and H358 (right) cells. (n=3 biological independent samples)

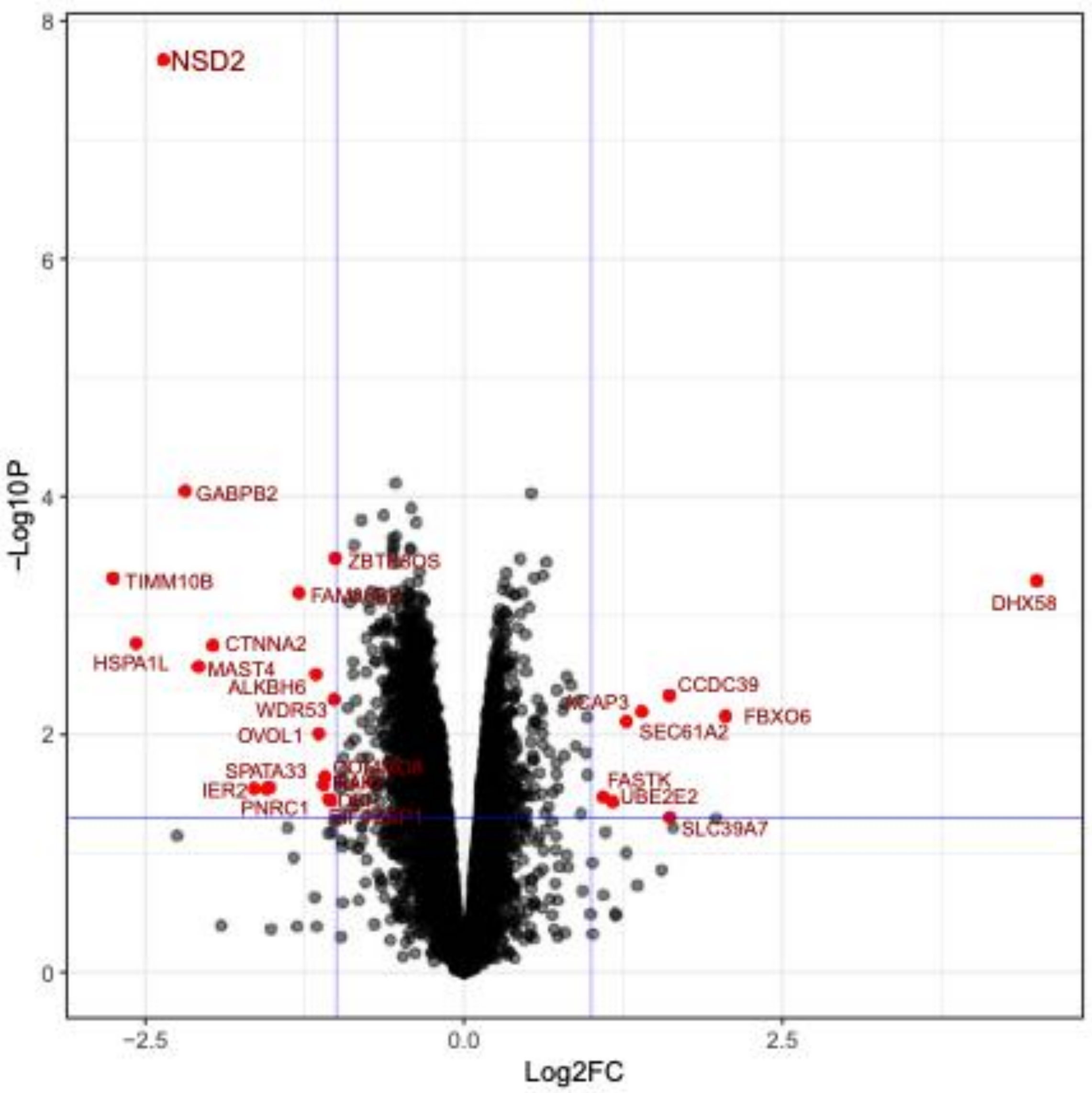

Figure S4. The unadjusted volcano plot for global proteomics analysis of T9 in H358 cells (N = 3) following a 4-hour treatment with DMSO or 4 μM T9.

Table S1. Stability of T9 in the presence of 10 mM GSH[α]

Time points	Remaining T9 (%)
5 min	87%
30 min	46%
1 h	61%
2 h	60%
12 h	62%

α: T9 (10 μM) was incubated with a physiological excess of GSH (10 mM) at 37°C, and the percentage of the remaining T9 was monitored at the timepoints 5 min, 30 min, 1h, 2h, and 12 h via analytical HPLC (water/acetonitrile, 5%–95%).

HPLC analysis of compounds

HPLC analysis of T1

<Chromatogram>

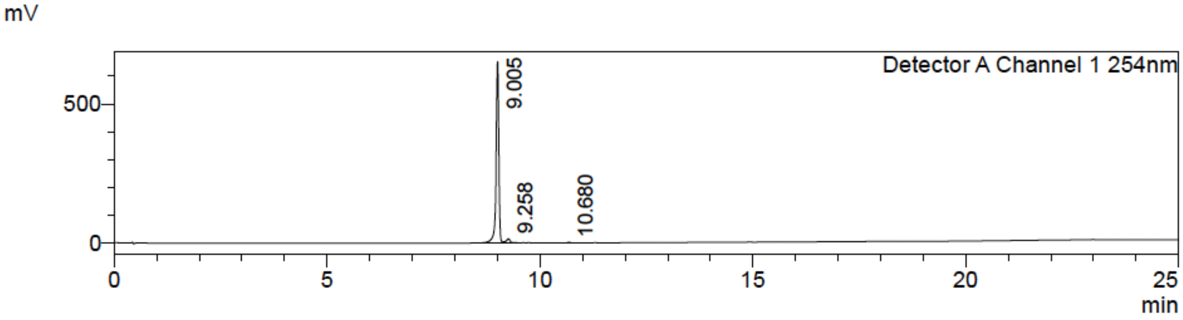

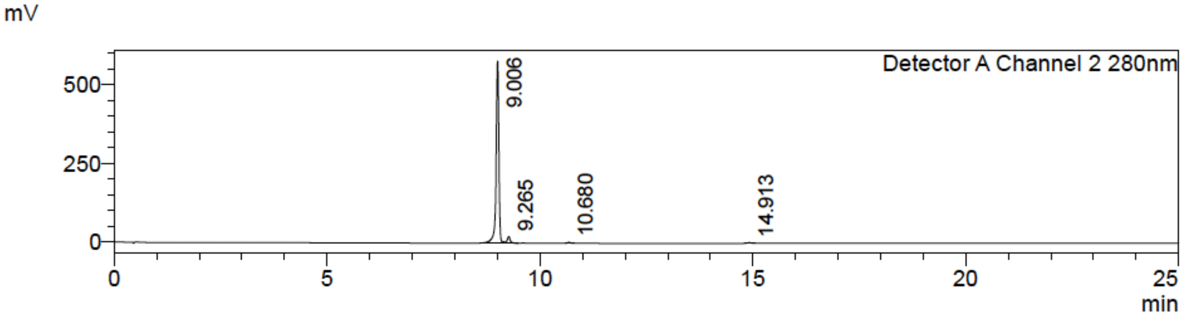

<Peak Table>

Detector A Channel 1 254nm

Peak#	Ret. Time	Height	Width at 5% Height	Area	Area%
1	9.005	649529	0.165	2942405	96.761
2	9.258	14797	--	88825	2.921
3	10.680	2137	0.147	9664	0.318
Total		666463		3040893	100.000

Detector A Channel 2 280nm

Peak#	Ret. Time	Height	Width at 5% Height	Area	Area%
1	9.006	576239	0.166	2621703	95.468
2	9.265	20197	--	98966	3.604
3	10.680	2884	0.150	13141	0.479
4	14.913	1929	0.205	12354	0.450
Total		601249		2746164	100.000

HPLC analysis of T2

<Chromatogram>

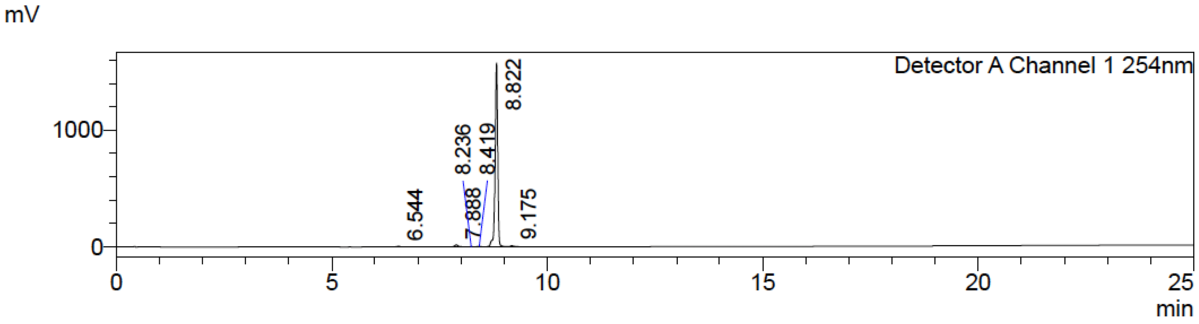

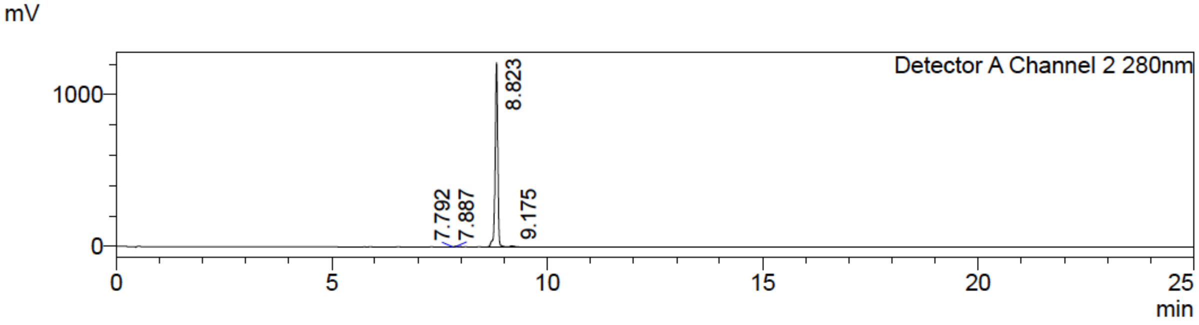

<Peak Table>

Detector A Channel 1 254nm

Peak#	Ret. Time	Height	Width at 5% Height	Area	Area%
1	6.544	5657	0.179	30873	0.454
2	7.888	15430	0.216	85457	1.257
3	8.236	1207	--	4988	0.073
4	8.419	3584	0.194	18161	0.267
5	8.822	1566434	0.142	6611076	97.211
6	9.175	7154	--	50192	0.738
Total		1599466		6800747	100.000

Detector A Channel 2 280nm

Peak#	Ret. Time	Height	Width at 5% Height	Area	Area%
1	7.792	2027	--	10693	0.206
2	7.887	3477	--	16330	0.315
3	8.823	1206891	0.141	5119213	98.613
4	9.175	6139	--	44966	0.866
Total		1218534		5191202	100.000

HPLC analysis of T3

<Chromatogram>

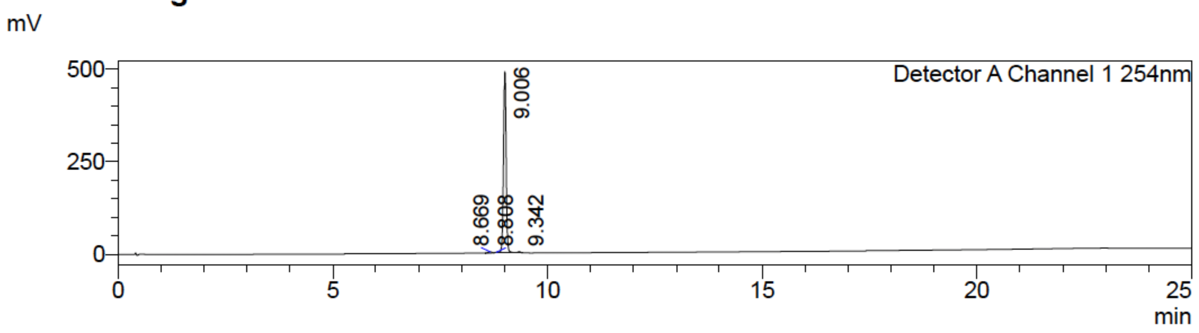

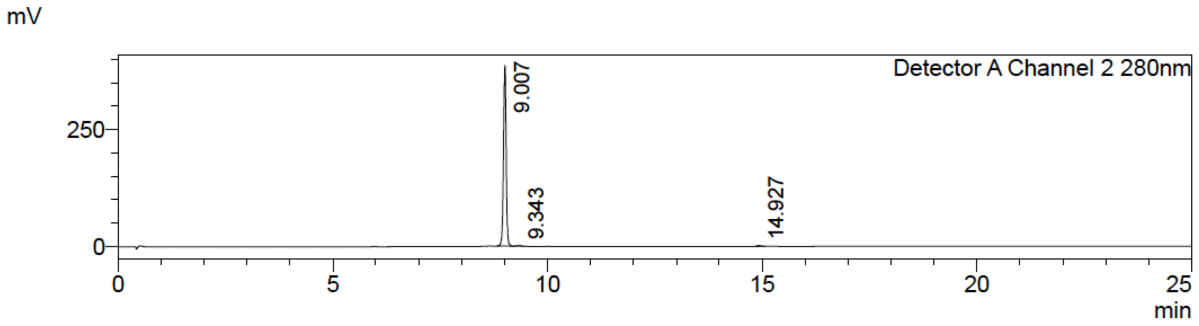

<Peak Table>

Detector A Channel 1 254nm

Peak#	Ret. Time	Height	Width at 5% Height	Area	Area%
1	8.669	2814	--	17155	0.793
2	8.808	1722	--	10623	0.491
3	9.006	487837	0.146	2118848	97.974
4	9.342	3147	--	16036	0.741
Total		495520		2162663	100.000

Detector A Channel 2 280nm

Peak#	Ret. Time	Height	Width at 5% Height	Area	Area%
1	9.007	382597	0.149	1675379	98.847
2	9.343	1606	0.136	6352	0.375
3	14.927	2110	0.198	13192	0.778
Total		386313		1694924	100.000

HPLC analysis of T4

<Chromatogram>

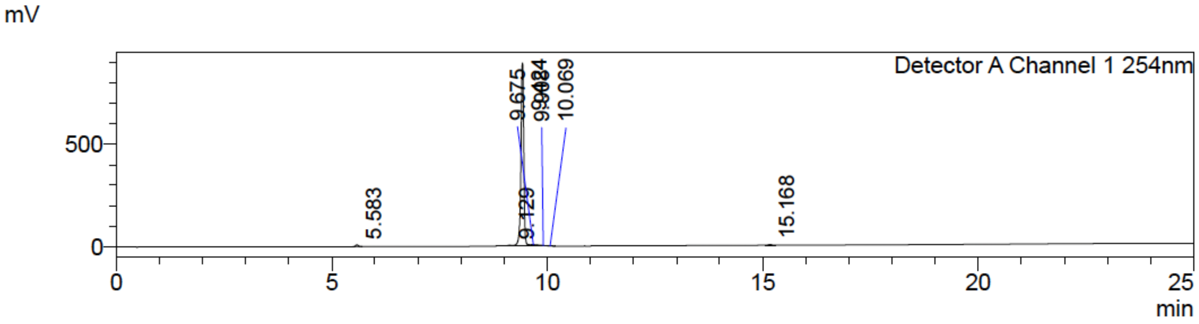

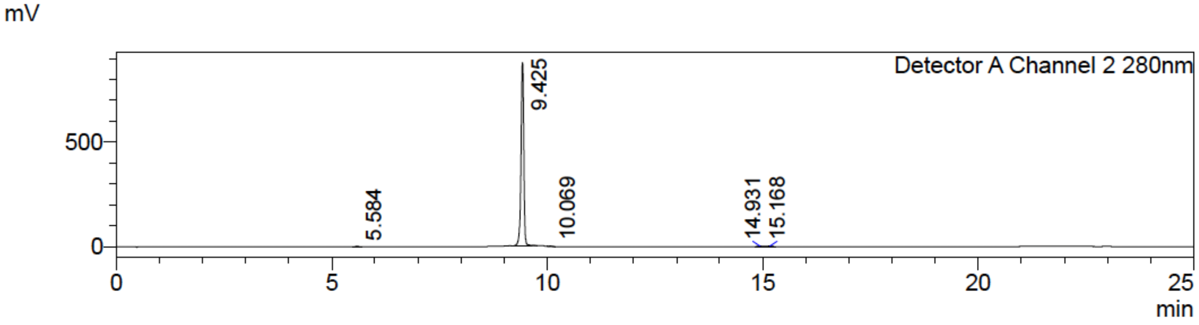

<Peak Table>

Detector A Channel 1 254nm

Peak#	Ret. Time	Height	Width at 5% Height	Area	Area%
1	5.583	7967	0.146	35423	0.856
2	9.129	2086	--	9493	0.229
3	9.424	883385	0.158	4051121	97.911
4	9.675	1944	--	7653	0.185
5	9.908	1251	--	6409	0.155
6	10.069	1393	0.130	5799	0.140
7	15.168	3610	0.194	21672	0.524
Total		901637		4137570	100.000

Detector A Channel 2 280nm

Peak#	Ret. Time	Height	Width at 5% Height	Area	Area%
1	5.584	2325	0.144	10283	0.259
2	9.425	866000	0.157	3922516	98.694
3	10.069	1789	0.128	7340	0.185
4	14.931	2253	--	15025	0.378
5	15.168	3038	--	19248	0.484
Total		875405		3974411	100.000

HPLC analysis of T5

<Chromatogram>

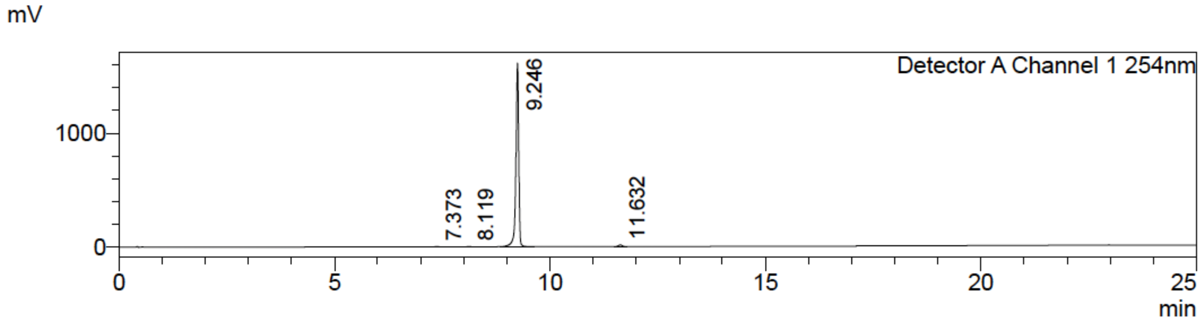

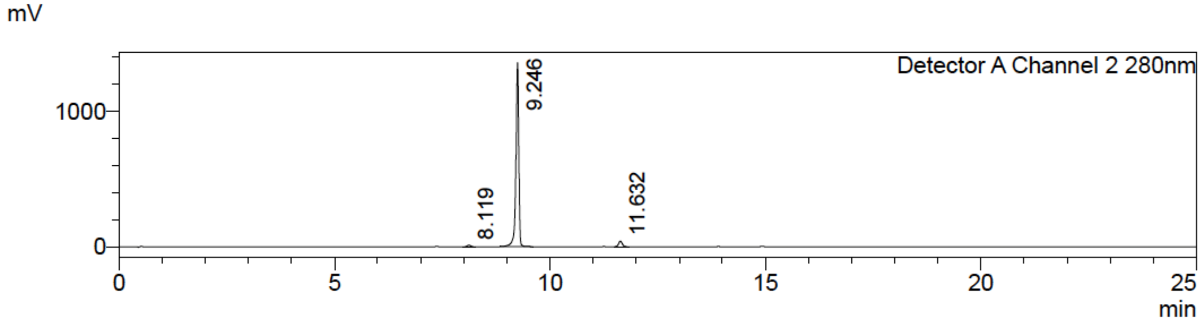

<Peak Table>

Detector A Channel 1 254nm

Peak#	Ret. Time	Height	Width at 5% Height	Area	Area%
1	7.373	2382	0.171	12710	0.171
2	8.119	1701	0.175	9222	0.124
3	9.246	1602451	0.166	7336085	98.725
4	11.632	12729	0.187	72848	0.980
Total		1619264		7430865	100.000

Detector A Channel 2 280nm

Peak#	Ret. Time	Height	Width at 5% Height	Area	Area%
1	8.119	10756	0.187	60349	0.928
2	9.246	1350896	0.167	6215919	95.538
3	11.632	39626	0.191	229926	3.534
Total		1401278		6506194	100.000

HPLC analysis of T6

<Chromatogram>

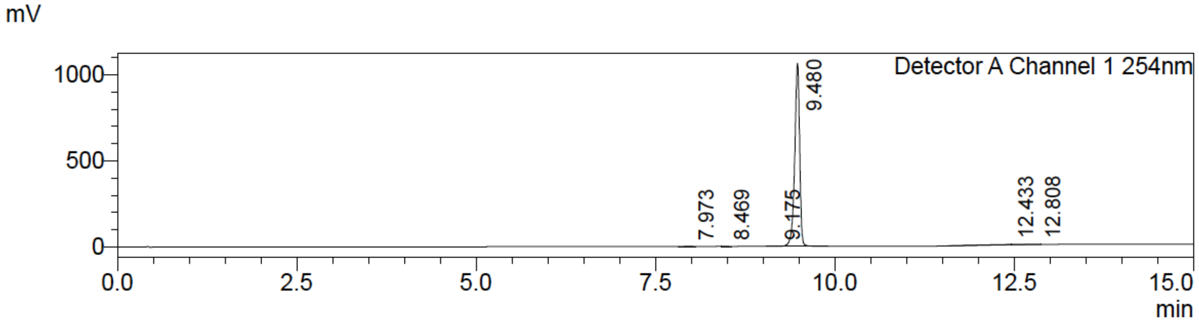

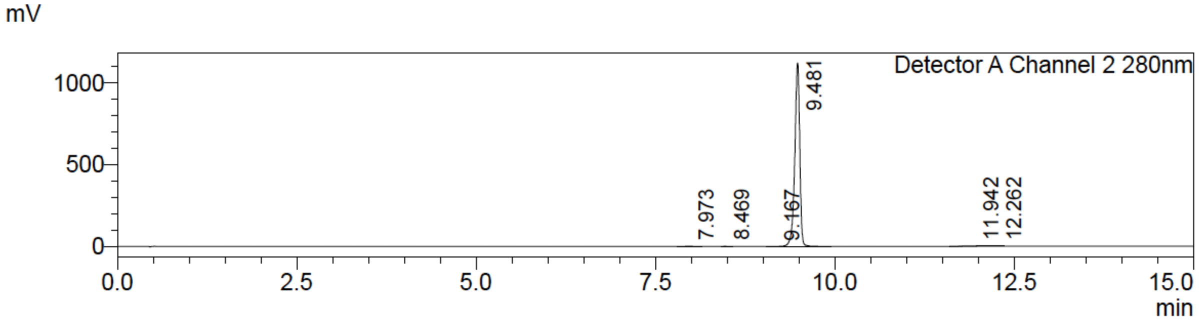

<Peak Table>

Detector A Channel 1 254nm

Peak#	Ret. Time	Height	Width at 5% Height	Area	Area%
1	7.973	1478	--	8809	0.168
2	8.469	488	--	2030	0.039
3	9.175	440	--	2274	0.043
4	9.480	1058769	0.171	5109388	97.685
5	12.433	2369	--	83189	1.590
6	12.808	452	--	24784	0.474
Total		1063995		5230474	100.000

Detector A Channel 2 280nm

Peak#	Ret. Time	Height	Width at 5% Height	Area	Area%
1	7.973	1466	0.215	8209	0.151
2	8.469	581	--	2462	0.045
3	9.167	431	--	2384	0.044
4	9.481	1117896	0.171	5410144	99.607
5	11.942	256	--	3035	0.056
6	12.262	288	--	5231	0.096
Total		1120918		5431465	100.000

HPLC analysis of T7

<Chromatogram>

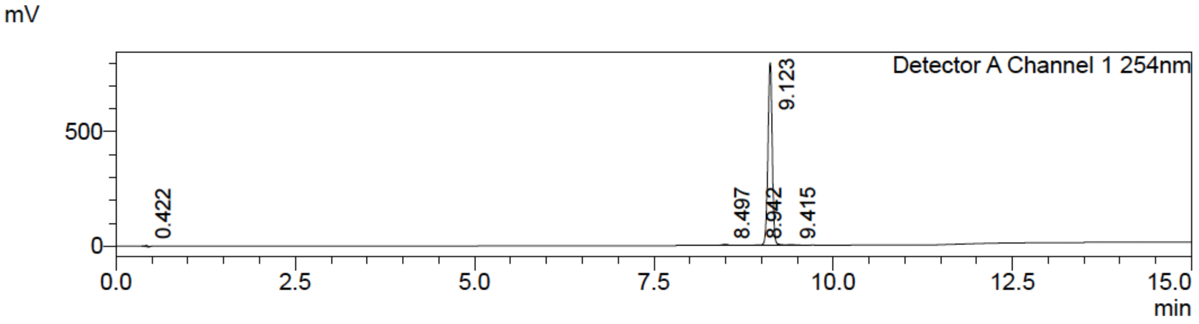

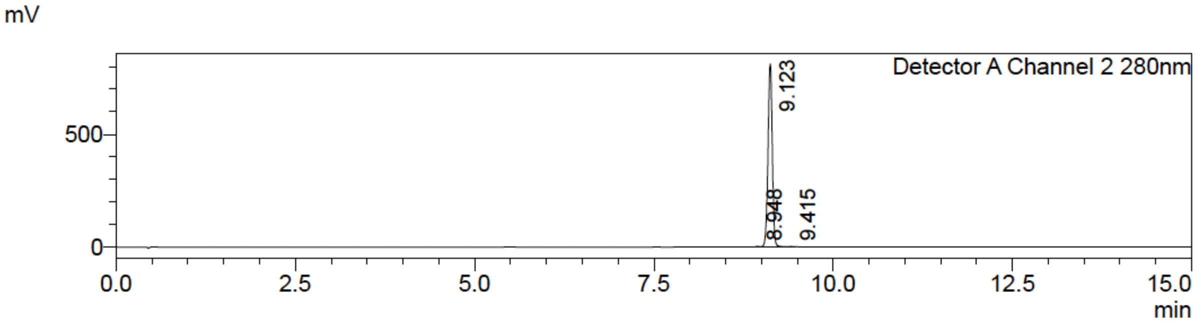

<Peak Table>

Detector A Channel 1 254nm

Peak#	Ret. Time	Height	Width at 5% Height	Area	Area%
1	0.422	4640	0.072	9926	0.290
2	8.497	4550	0.169	22875	0.669
3	8.942	1653	--	7384	0.216
4	9.123	794179	0.141	3355142	98.191
5	9.415	2951	--	21621	0.633
Total		807974		3416947	100.000

Detector A Channel 2 280nm

Peak#	Ret. Time	Height	Width at 5% Height	Area	Area%
1	8.948	3281	--	13438	0.390
2	9.123	805010	0.141	3413769	99.175
3	9.415	2652	--	14943	0.434
Total		810943		3442150	100.000

HPLC analysis of T8

<Chromatogram>

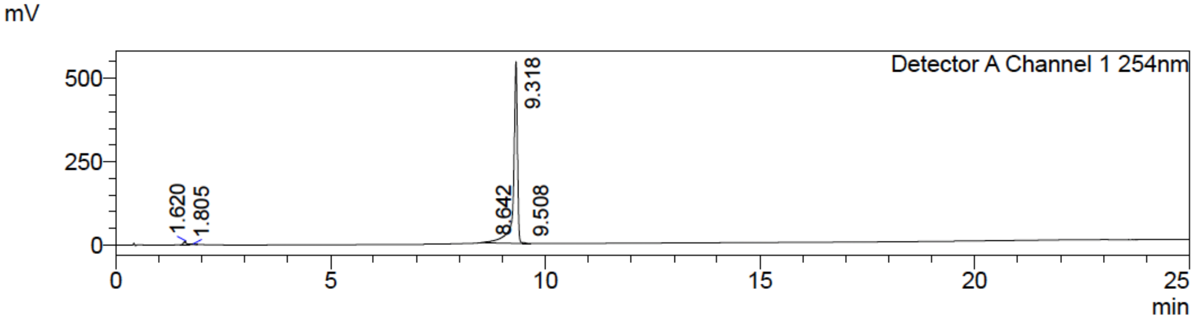

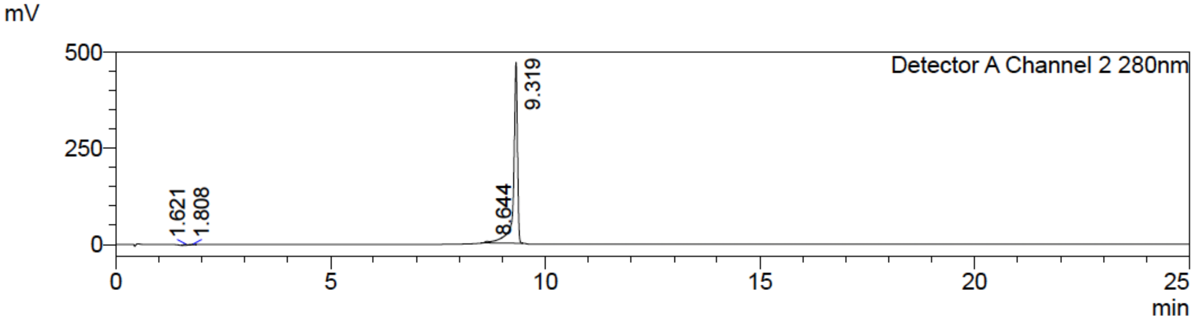

<Peak Table>

Detector A Channel 1 254nm

Peak#	Ret. Time	Height	Width at 5% Height	Area	Area%
1	1.620	11315	--	43828	1.236
2	1.805	1834	--	10812	0.305
3	8.642	3682	--	28797	0.812
4	9.318	538497	0.306	3450881	97.333
5	9.508	2494	--	11113	0.313
Total		557823		3545430	100.000

Detector A Channel 2 280nm

Peak#	Ret. Time	Height	Width at 5% Height	Area	Area%
1	1.621	3660	--	13154	0.438
2	1.808	1621	--	9715	0.323
3	8.644	4395	--	30023	1.000
4	9.319	466363	0.300	2950406	98.239
Total		476039		3003298	100.000

HPLC analysis of T9

<Chromatogram>

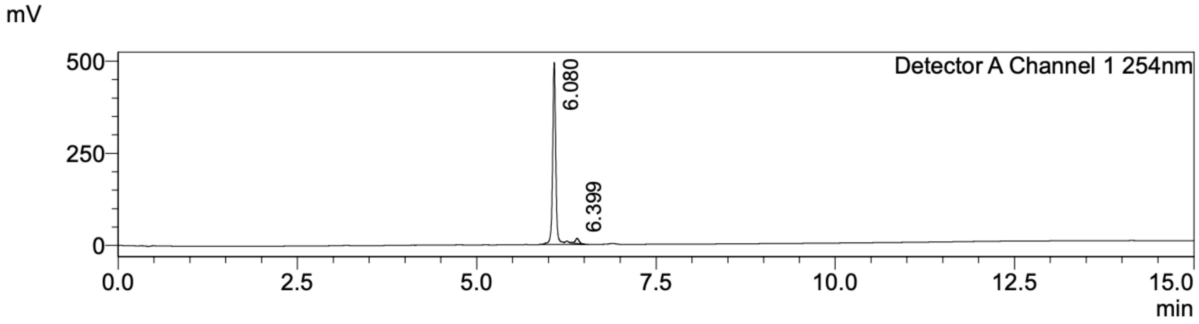

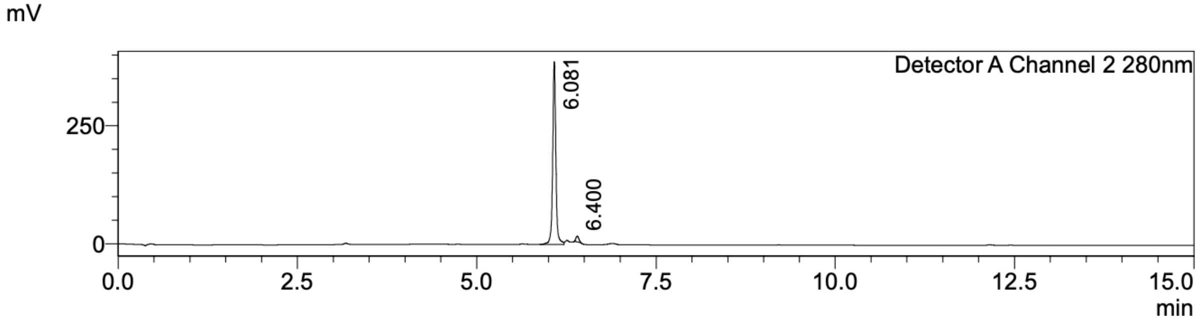

<Peak Table>

Detector A Channel 1 254nm

Peak#	Ret. Time	Height	Width at 5% Height	Area	Area%
1	6.080	489349	0.109	1548918	96.444
2	6.399	14253	--	57118	3.556
Total		503602		1606035	100.000

Detector A Channel 2 280nm

Peak#	Ret. Time	Height	Width at 5% Height	Area	Area%
1	6.081	383891	0.115	1190112	97.178
2	6.400	12106	--	34558	2.822
Total		395997		1224669	100.000

HPLC analysis of T10

<Chromatogram>

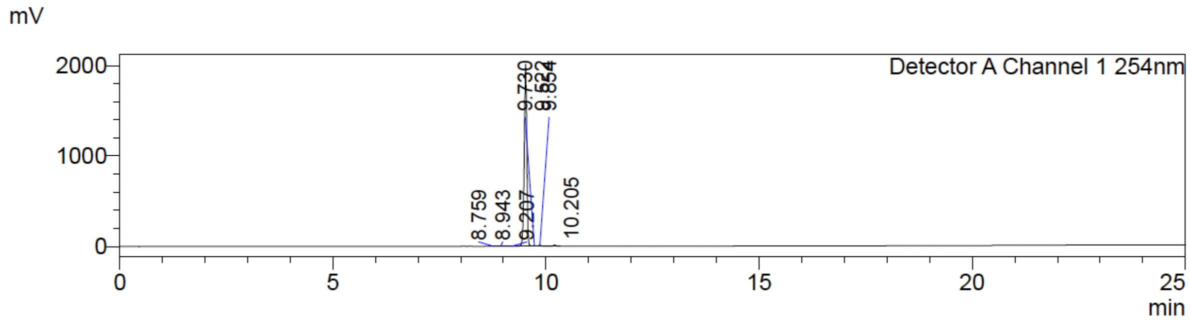

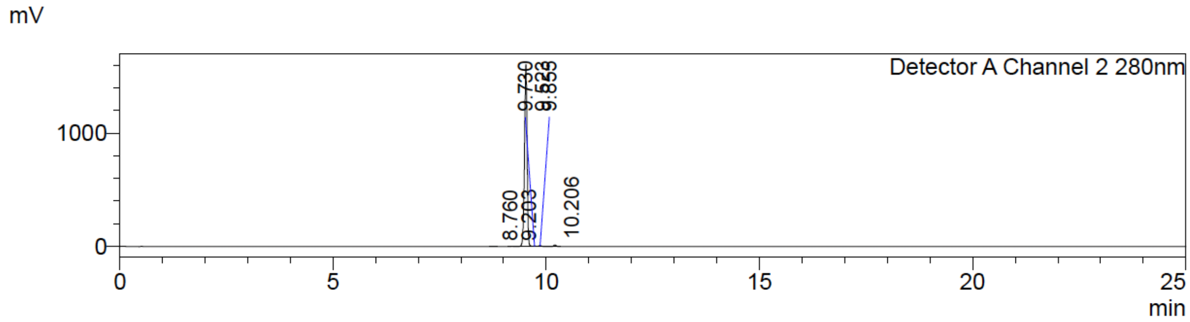

<Peak Table>

Detector A Channel 1 254nm

Peak#	Ret. Time	Height	Width at 5% Height	Area	Area%
1	8.759	2097	--	8783	0.102
2	8.943	1511	--	8799	0.102
3	9.207	2656	--	16879	0.196
4	9.522	2004011	0.143	8447327	97.857
5	9.730	7180	--	35643	0.413
6	9.854	10251	--	58195	0.674
7	10.205	12875	0.144	56688	0.657
Total		2040582		8632314	100.000

Detector A Channel 2 280nm

Peak#	Ret. Time	Height	Width at 5% Height	Area	Area%
1	8.760	1631	0.128	6529	0.093
2	9.203	1880	0.160	9284	0.133
3	9.523	1600667	0.144	6838754	97.746
4	9.730	5647	--	28389	0.406
5	9.855	8870	--	49606	0.709
6	10.206	14722	0.142	63924	0.914
Total		1633418		6996486	100.000

HPLC analysis of T11

<Chromatogram>

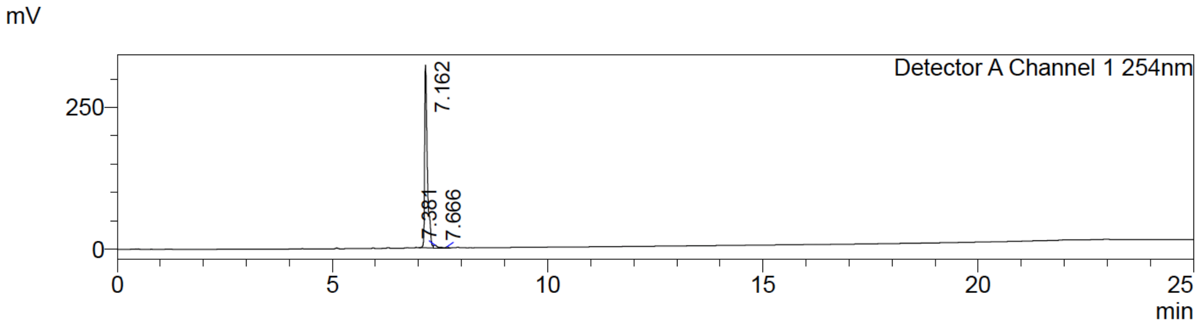

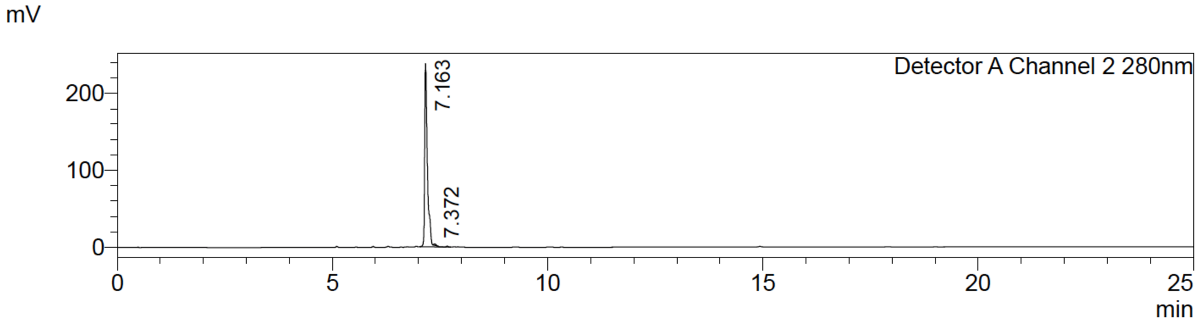

<Peak Table>

Detector A Channel 1 254nm

Peak#	Ret. Time	Height	Width at 5% Height	Area	Area%
1	7.162	319126	0.176	1422729	97.602
2	7.381	4865	--	25485	1.748
3	7.666	2070	--	9469	0.650
Total		326062		1457684	100.000

Detector A Channel 2 280nm

Peak#	Ret. Time	Height	Width at 5% Height	Area	Area%
1	7.163	236069	0.176	1068213	99.418
2	7.372	1738	0.103	6252	0.582
Total		237807		1074465	100.000

HPLC analysis of T12

<Chromatogram>

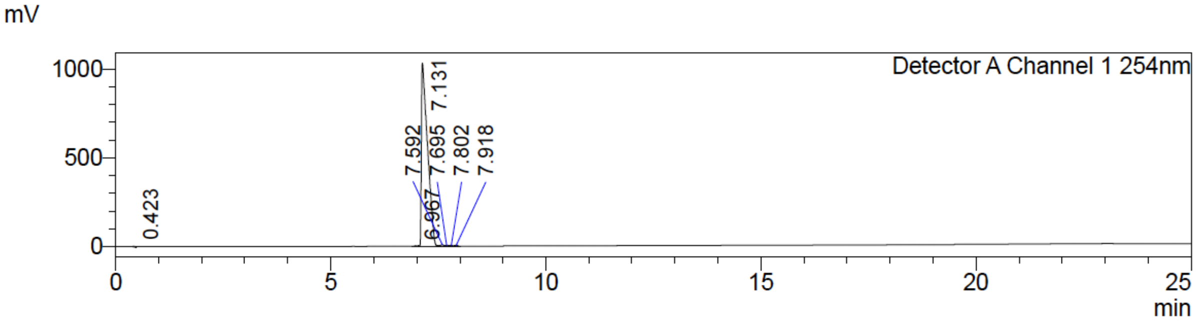

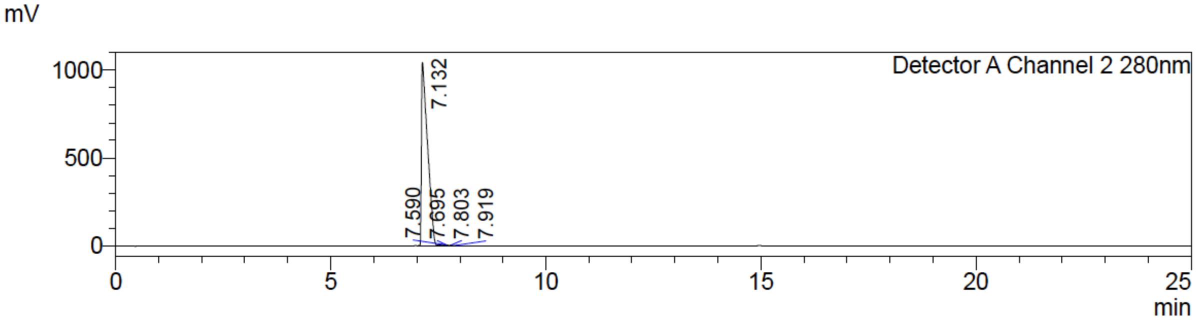

<Peak Table>

Detector A Channel 1 254nm

Peak#	Ret. Time	Height	Width at 5% Height	Area	Area%
1	0.423	3724	0.077	9056	0.088
2	6.967	1257	--	5478	0.053
3	7.131	1027892	0.319	10194359	98.897
4	7.592	8131	--	44004	0.427
5	7.695	4648	--	20069	0.195
6	7.802	4744	--	16960	0.165
7	7.918	5247	--	18135	0.176
Total		1055644		10308062	100.000

Detector A Channel 2 280nm

Peak#	Ret. Time	Height	Width at 5% Height	Area	Area%
1	7.132	1040481	0.319	10395329	99.433
2	7.590	6822	0.139	27301	0.261
3	7.695	3585	--	11497	0.110
4	7.803	4353	0.086	12678	0.121
5	7.919	2512	0.094	7750	0.074
Total		1057753		10454554	100.000

HPLC analysis of T13

<Chromatogram>

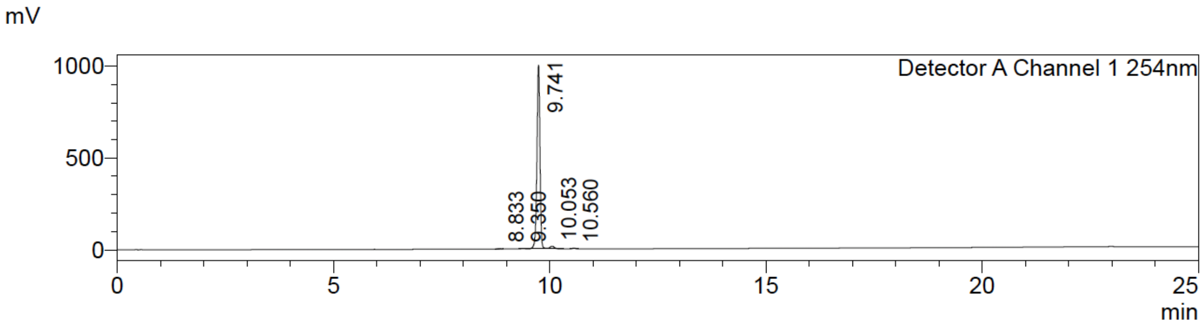

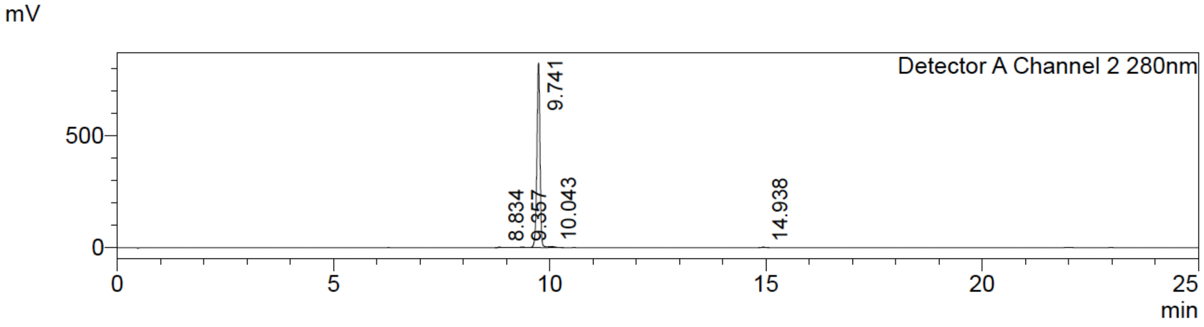

<Peak Table>

Detector A Channel 1 254nm

Peak#	Ret. Time	Height	Width at 5% Height	Area	Area%
1	8.833	2974	0.134	12554	0.249
2	9.350	1126	--	10279	0.204
3	9.741	993056	0.166	4892196	97.032
4	10.053	14374	--	109423	2.170
5	10.560	3345	0.160	17375	0.345
Total		1014875		5041827	100.000

Detector A Channel 2 280nm

Peak#	Ret. Time	Height	Width at 5% Height	Area	Area%
1	8.834	2437	0.131	10173	0.244
2	9.357	2164	0.163	11325	0.271
3	9.741	816397	0.169	4089052	97.972
4	10.043	5497	--	49394	1.183
5	14.938	2182	0.203	13739	0.329
Total		828677		4173682	100.000

HPLC analysis of T14

<Chromatogram>

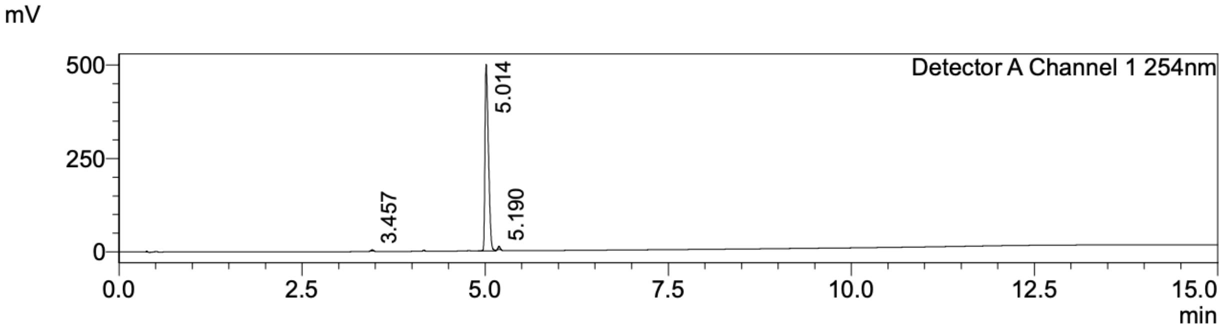

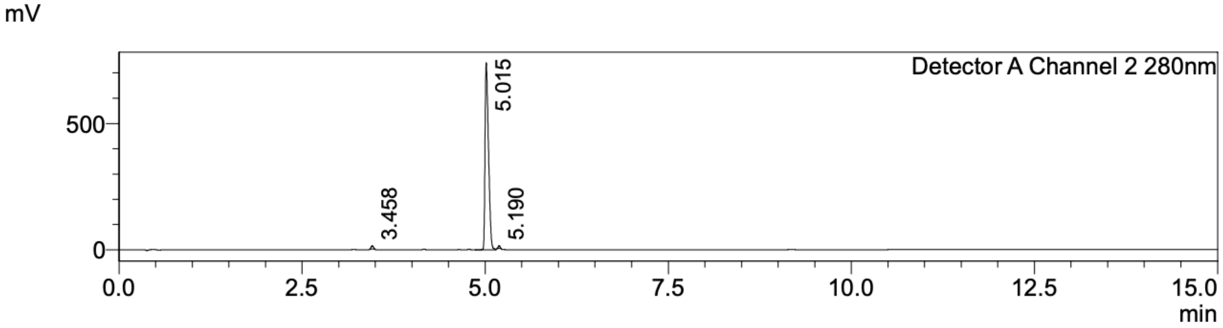

<Peak Table>

Detector A Channel 1 254nm

Peak#	Ret. Time	Height	Width at 5% Height	Area	Area%
1	3.457	2888	--	5277	0.318
2	5.014	494768	0.106	1638383	98.838
3	5.190	8251	--	13985	0.844
Total		505907		1657645	100.000

Detector A Channel 2 280nm

Peak#	Ret. Time	Height	Width at 5% Height	Area	Area%
1	3.458	12474	--	28061	1.135
2	5.015	733211	0.106	2426463	98.119
3	5.190	10816	--	18450	0.746
Total		756501		2472974	100.000

HPLC analysis of T15

<Chromatogram>

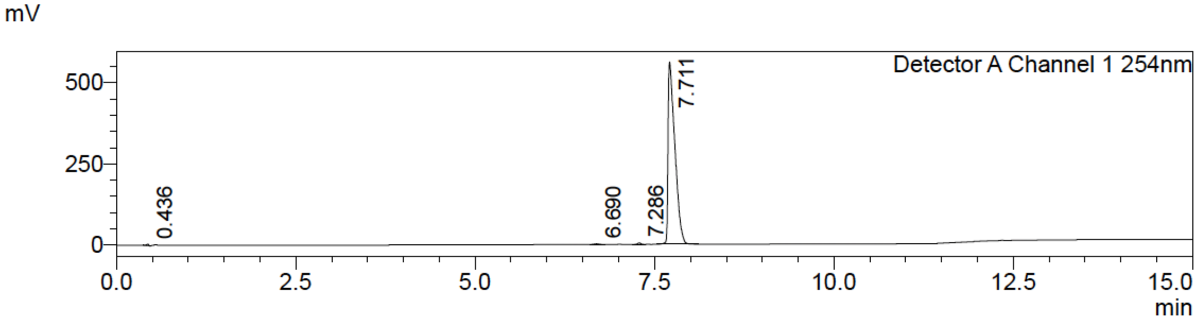

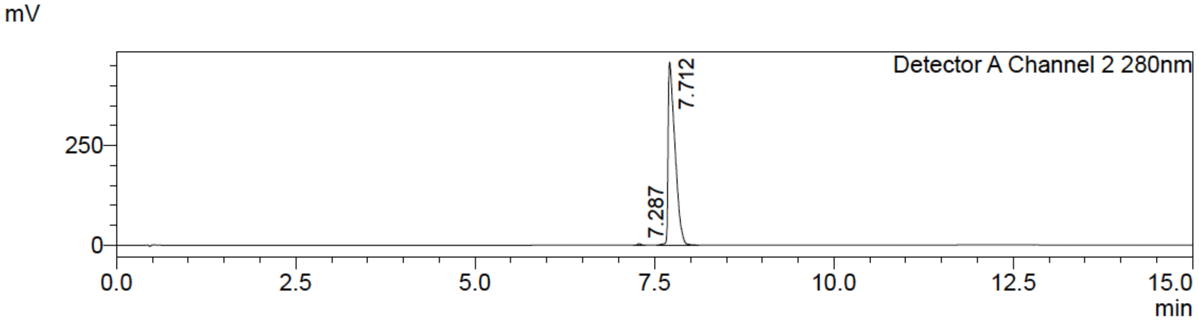

<Peak Table>

Detector A Channel 1 254nm

Peak#	Ret. Time	Height	Width at 5% Height	Area	Area%
1	0.436	4013	0.078	8955	0.240
2	6.690	2278	0.159	11405	0.306
3	7.286	4117	0.106	13204	0.354
4	7.711	555322	0.224	3695117	99.100
Total		565730		3728681	100.000

Detector A Channel 2 280nm

Peak#	Ret. Time	Height	Width at 5% Height	Area	Area%
1	7.287	3370	0.105	10693	0.351
2	7.712	455419	0.226	3036185	99.649
Total		458789		3046878	100.000

HPLC analysis of T16

<Chromatogram>

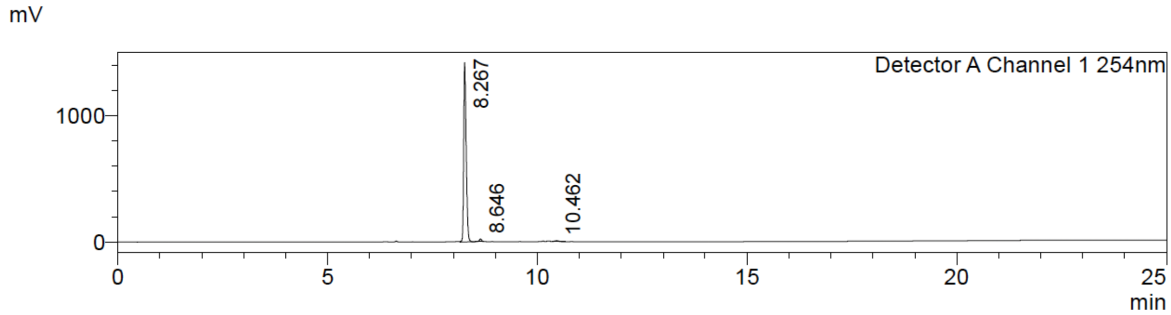

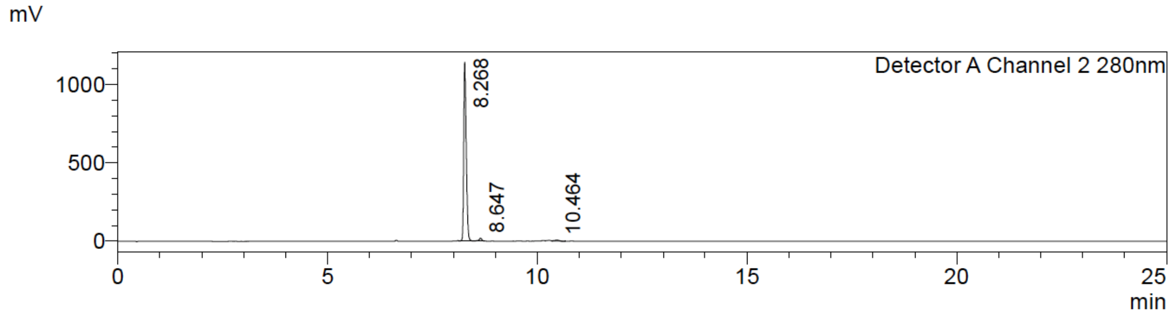

<Peak Table>

Detector A Channel 1 254nm

Peak#	Ret. Time	Height	Width at 5% Height	Area	Area%
1	8.267	1395251	0.140	5916372	97.312
2	8.646	22435	--	105633	1.737
3	10.462	8199	--	57804	0.951
Total		1425885		6079809	100.000

Detector A Channel 2 280nm

Peak#	Ret. Time	Height	Width at 5% Height	Area	Area%
1	8.268	1126674	0.140	4791822	96.881
2	8.647	19280	--	99978	2.021
3	10.464	7506	--	54298	1.098
Total		1153460		4946098	100.000

HPLC analysis of T17

<Chromatogram>

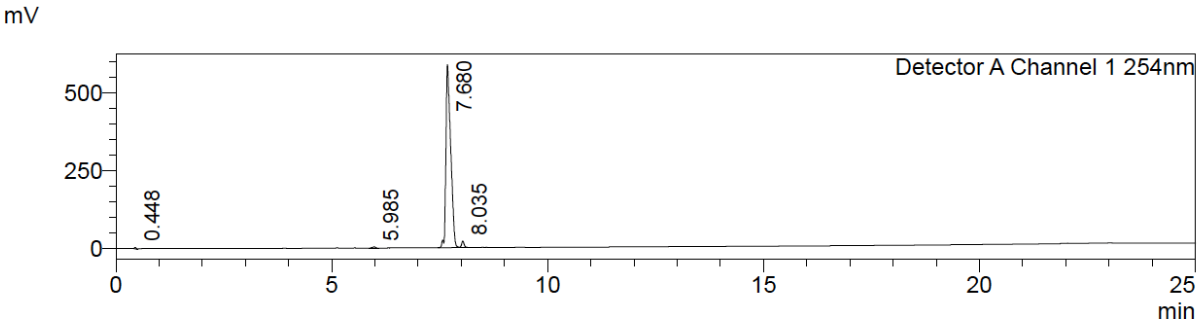

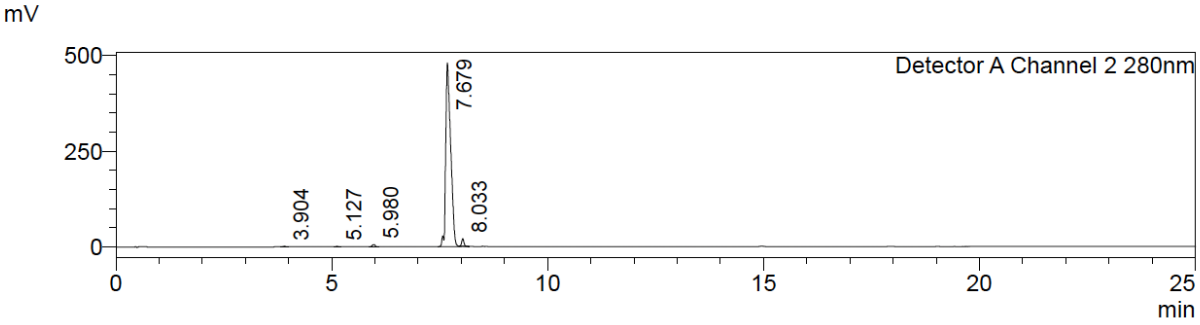

<Peak Table>

Detector A Channel 1 254nm

Peak#	Ret. Time	Height	Width at 5% Height	Area	Area%
1	0.448	4533	0.077	11825	0.257
2	5.985	3774	0.149	17520	0.381
3	7.680	584777	0.245	4492339	97.593
4	8.035	20121	--	81433	1.769
Total		613205		4603117	100.000

Detector A Channel 2 280nm

Peak#	Ret. Time	Height	Width at 5% Height	Area	Area%
1	3.904	1678	0.128	6677	0.169
2	5.127	1509	0.119	5575	0.141
3	5.980	5904	0.145	28422	0.720
4	7.679	478083	0.250	3834423	97.166
5	8.033	19405	0.122	71172	1.804
Total		506579		3946268	100.000

HPLC analysis of T18

<Chromatogram>

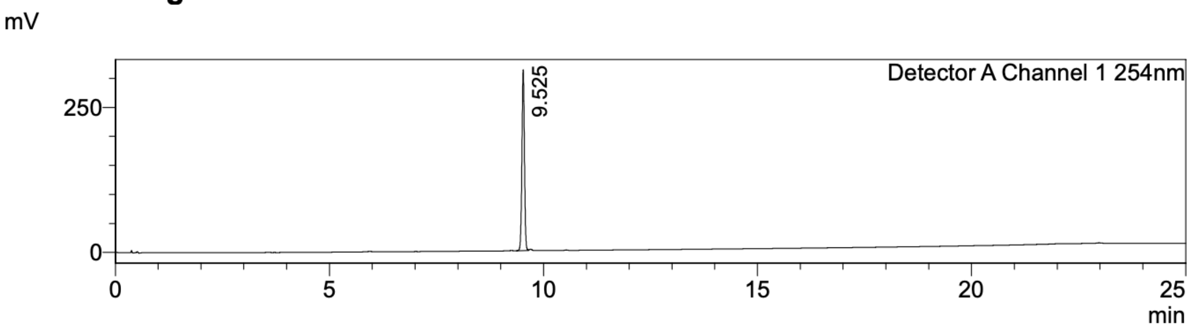

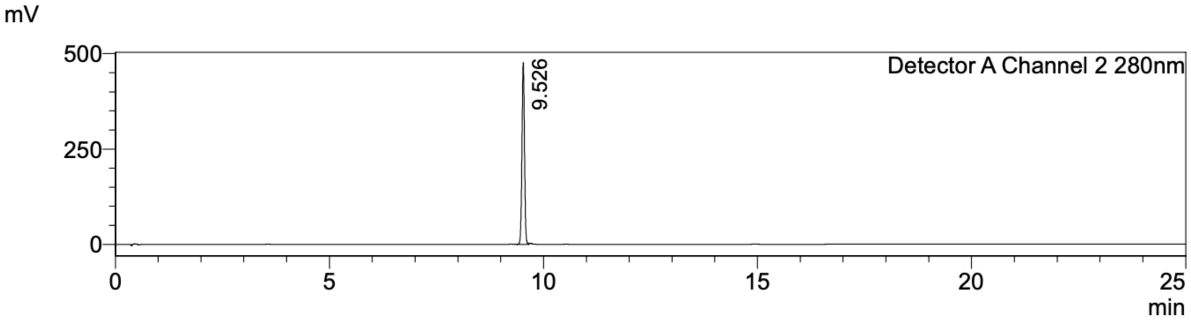

<Peak Table>

Detector A Channel 1 254nm

Peak#	Ret. Time	Height	Width at 5% Height	Area	Area%
1	9.525	307397	0.137	1276843	100.000
Total		307397		1276843	100.000

Detector A Channel 2 280nm

Peak#	Ret. Time	Height	Width at 5% Height	Area	Area%
1	9.526	469607	0.136	1949127	100.000
Total		469607		1949127	100.000

HPLC analysis of T19

<Chromatogram>

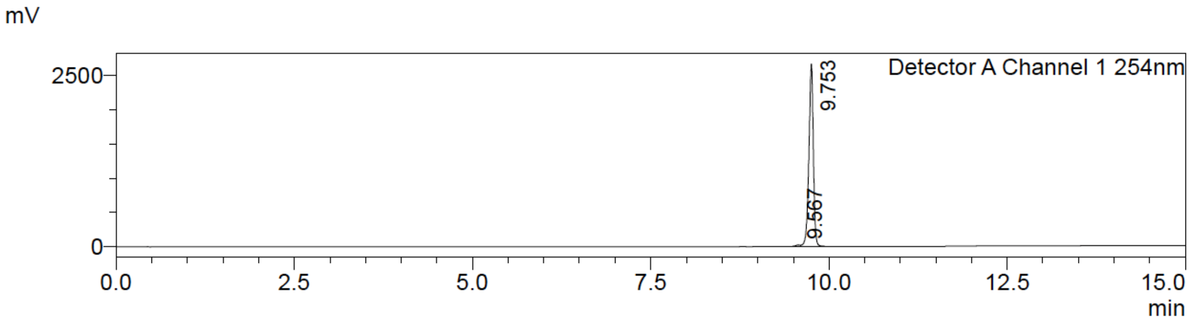

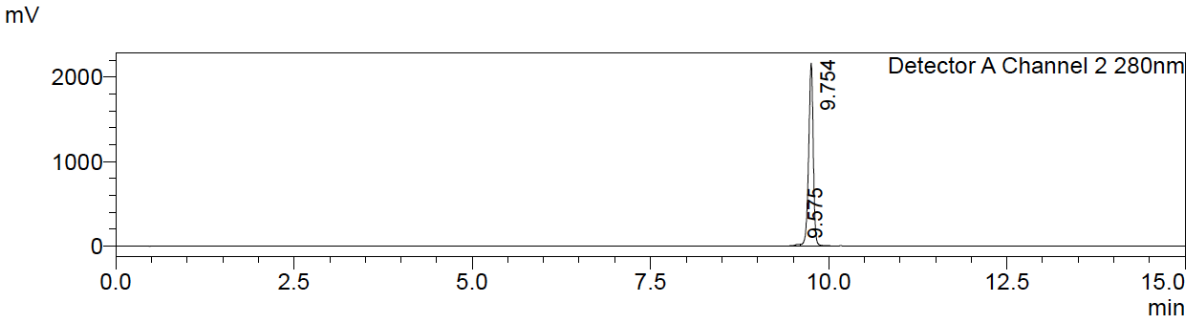

<Peak Table>

Detector A Channel 1 254nm

Peak#	Ret. Time	Height	Width at 5% Height	Area	Area%
1	9.567	21817	--	106010	0.906
2	9.753	2643047	0.155	11599950	99.094
Total		2664864		11705960	100.000

Detector A Channel 2 280nm

Peak#	Ret. Time	Height	Width at 5% Height	Area	Area%
1	9.575	18378	--	86408	0.879
2	9.754	2151610	0.158	9748794	99.121
Total		2169988		9835203	100.000

HPLC analysis of T20

<Chromatogram>

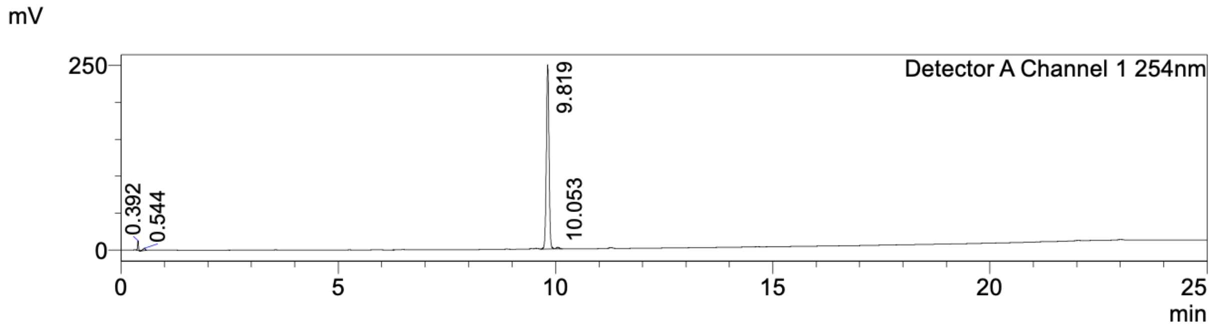

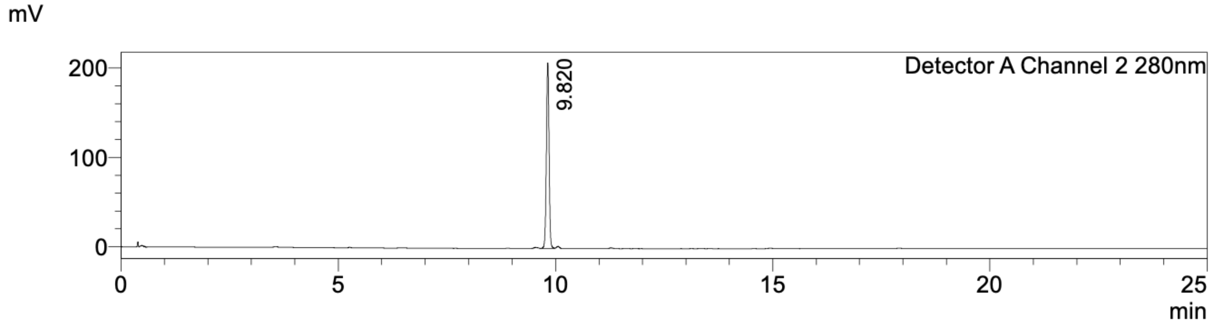

<Peak Table>

Detector A Channel 1 254nm

Peak#	Ret. Time	Height	Width at 5% Height	Area	Area%
1	0.392	11747	0.050	18537	1.678
2	0.544	2977	0.113	10142	0.918
3	9.819	246563	0.143	1064803	96.399
4	10.053	2319	--	11100	1.005
Total		263605		1104582	100.000

Detector A Channel 2 280nm

Peak#	Ret. Time	Height	Width at 5% Height	Area	Area%
1	9.820	206639	0.144	893185	100.000
Total		206639		893185	100.000

HPLC analysis of T21

<Chromatogram>

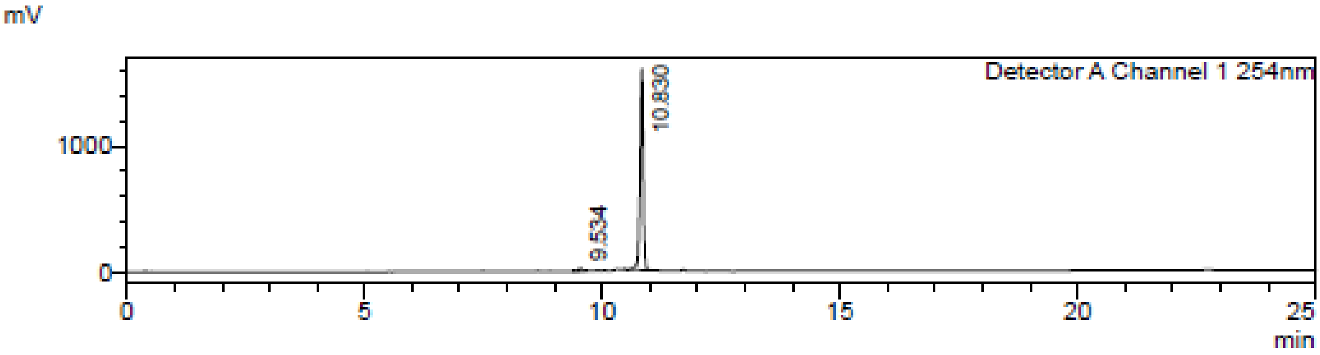

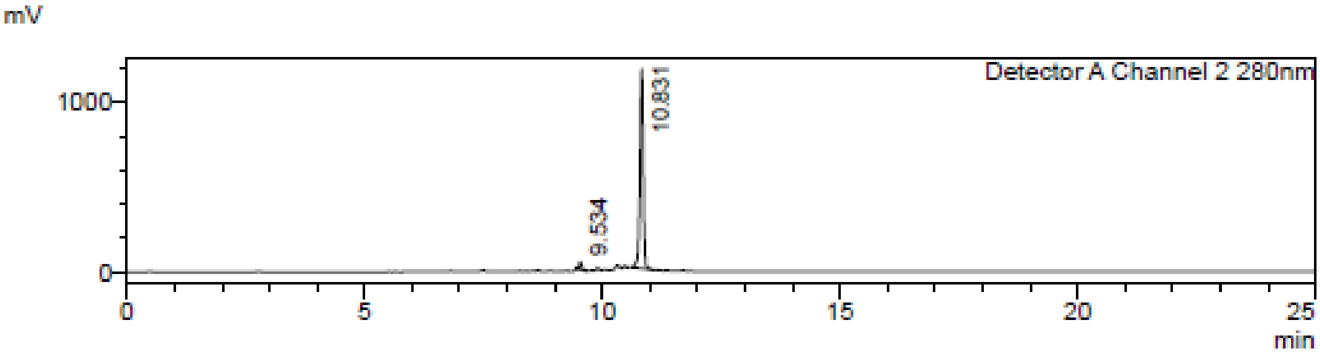

<Peak Table>

Detector A Channel 1 254nm

Peak#	Ret. Time	Height	Width at 5% Height	Area	Area%
1	9.534	22403	0.201	108477	1.360
2	10.830	1586725	0.167	7867593	98.640
Total		1609128		7976070	100.000

Detector A Channel 2 280nm

Peak#	Ret. Time	Height	Width at 5% Height	Area	Area%
1	9.534	43948	0.105	147694	2.457
2	10.831	1171578	0.172	5862620	97.543
Total		1215526		6010314	100.000

HPLC analysis of T22

<Chromatogram>

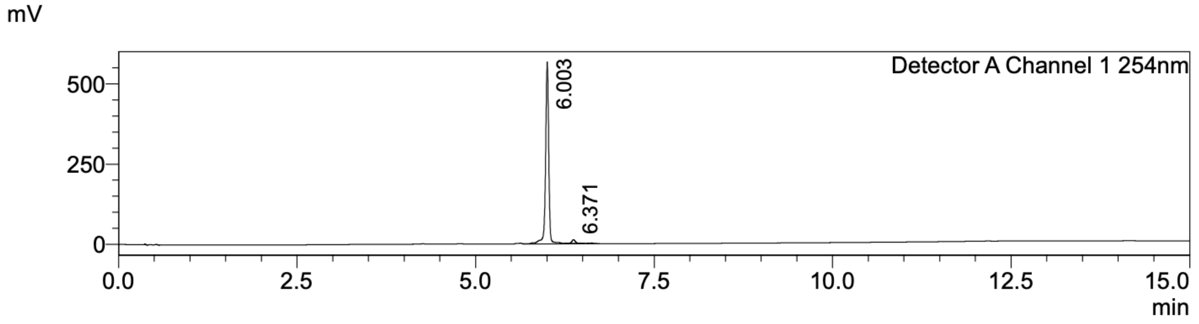

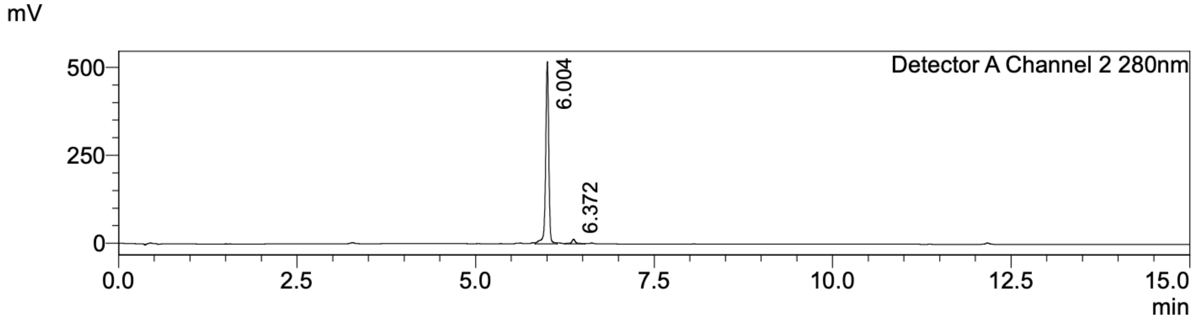

<Peak Table>

Detector A Channel 1 254nm

Peak#	Ret. Time	Height	Width at 5% Height	Area	Area%
1	6.003	557156	0.104	1774868	97.455
2	6.371	11935	0.230	46346	2.545
Total		569092		1821214	100.000

Detector A Channel 2 280nm

Peak#	Ret. Time	Height	Width at 5% Height	Area	Area%
1	6.004	513322	0.104	1574624	96.424
2	6.372	12958	--	58401	3.576
Total		526280		1633024	100.000

HPLC analysis of T23

<Chromatogram>

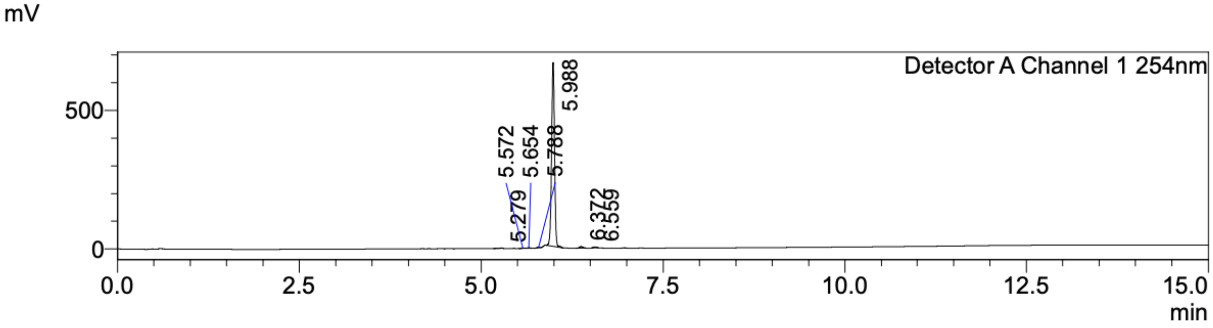

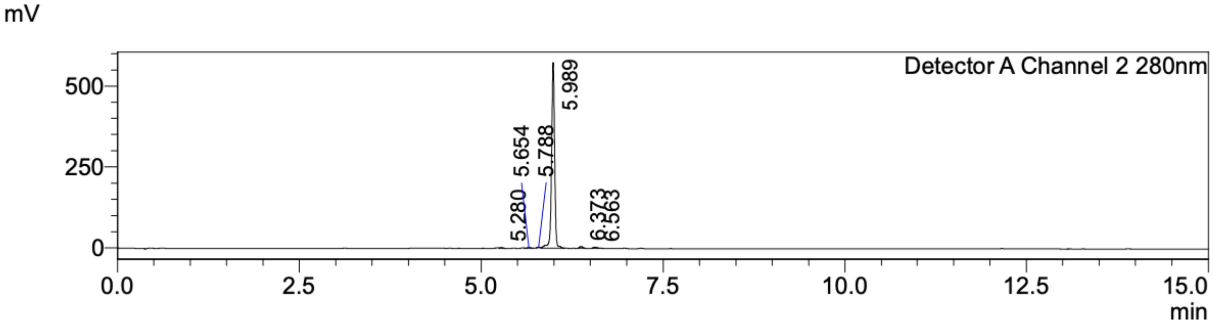

<Peak Table>

Detector A Channel 1 254nm

Peak#	Ret. Time	Height	Width at 5% Height	Area	Area%
1	5.279	1745	0.126	7165	0.374
2	5.572	1510	0.059	3156	0.165
3	5.654	1963	0.069	4659	0.243
4	5.788	1862	0.065	4359	0.227
5	5.988	658316	0.094	1869175	97.474
6	6.372	5777	0.103	18553	0.967
7	6.559	3538	0.086	10543	0.550
Total		674710		1917609	100.000

Detector A Channel 2 280nm

Peak#	Ret. Time	Height	Width at 5% Height	Area	Area%
1	5.280	2024	0.060	4037	0.234
2	5.654	2428	0.080	5872	0.340
3	5.788	1746	--	3663	0.212
4	5.989	567310	0.099	1687035	97.808
5	6.373	5018	0.083	13947	0.809
6	6.563	2505	--	10283	0.596

HPLC analysis of T24

<Chromatogram>

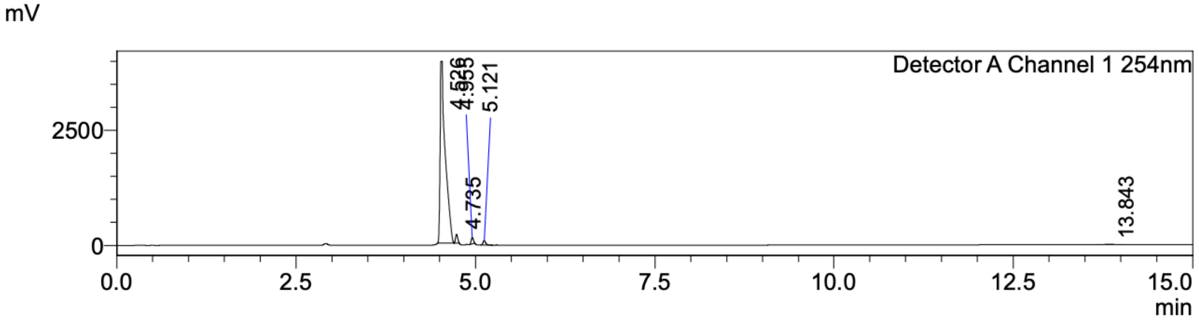

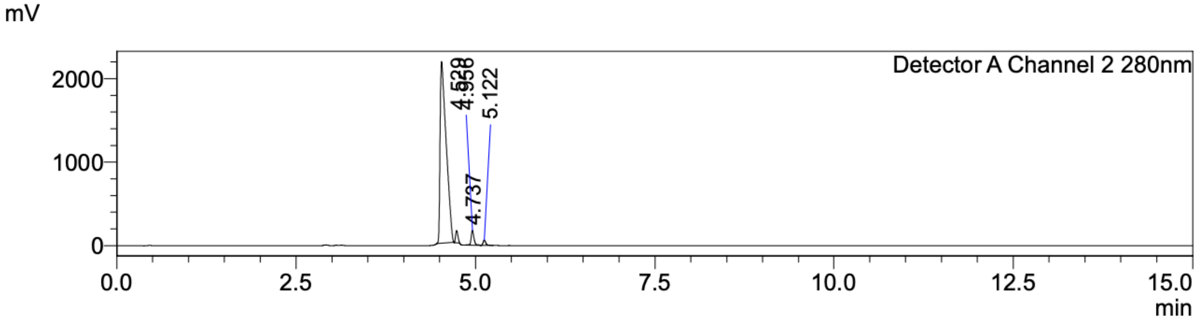

<Peak Table>

Detector A Channel 1 254nm

Peak#	Ret. Time	Height	Width at 5% Height	Area	Area%
1	4.526	3950101	0.181	19653678	95.062
2	4.735	178327	0.061	382321	1.849
3	4.955	135337	0.063	293064	1.418
4	5.121	96801	0.107	260085	1.258
5	13.843	11608	0.246	85346	0.413
Total		4372174		20674493	100.000

Detector A Channel 2 280nm

Peak#	Ret. Time	Height	Width at 5% Height	Area	Area%
1	4.529	2157138	0.188	12769002	92.833
2	4.737	142890	0.067	326828	2.376
3	4.956	174145	0.090	488349	3.550
4	5.122	62918	0.112	170568	1.240
Total		2537089		13754747	100.000

HPLC analysis of T25

<Chromatogram>

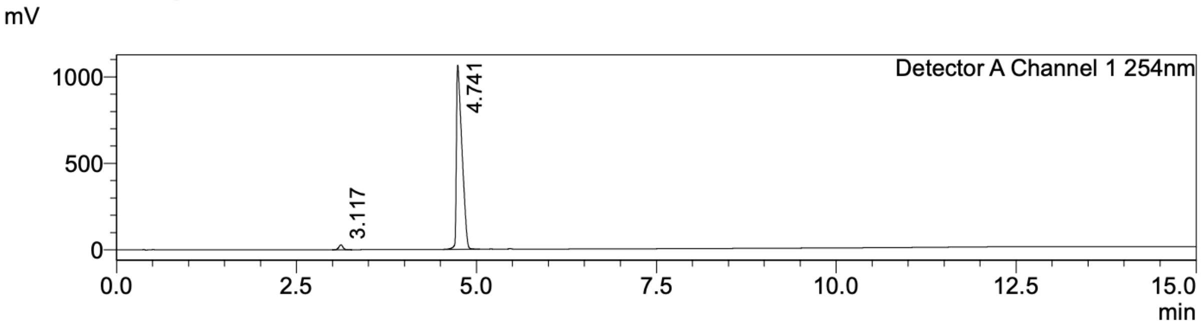

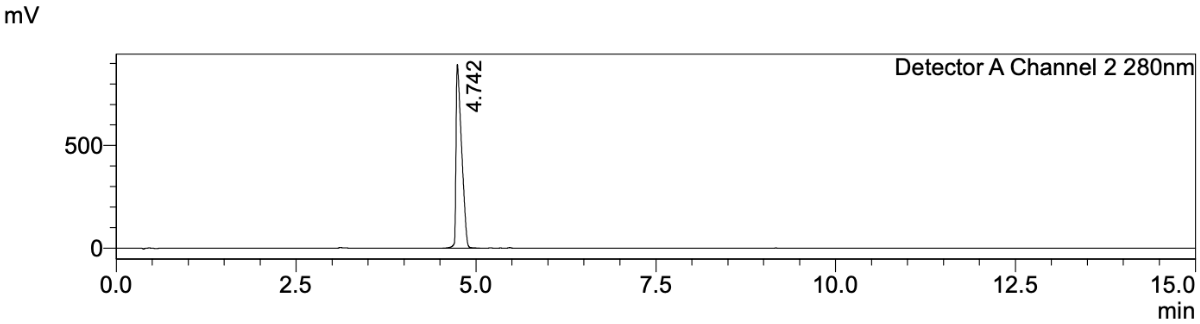

<Peak Table>

Detector A Channel 1 254nm

Peak#	Ret. Time	Height	Width at 5% Height	Area	Area%
1	3.117	27968	0.132	108904	1.869
2	4.741	1055608	0.172	5718860	98.131
Total		1083576		5827764	100.000

Detector A Channel 2 280nm

Peak#	Ret. Time	Height	Width at 5% Height	Area	Area%
1	4.742	885109	0.172	4829349	100.000
Total		885109		4829349	100.000

HPLC analysis of T26

<Chromatogram>

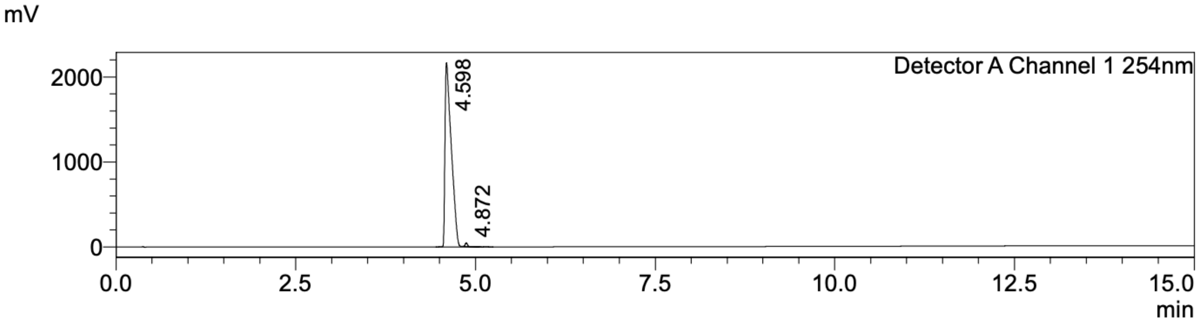

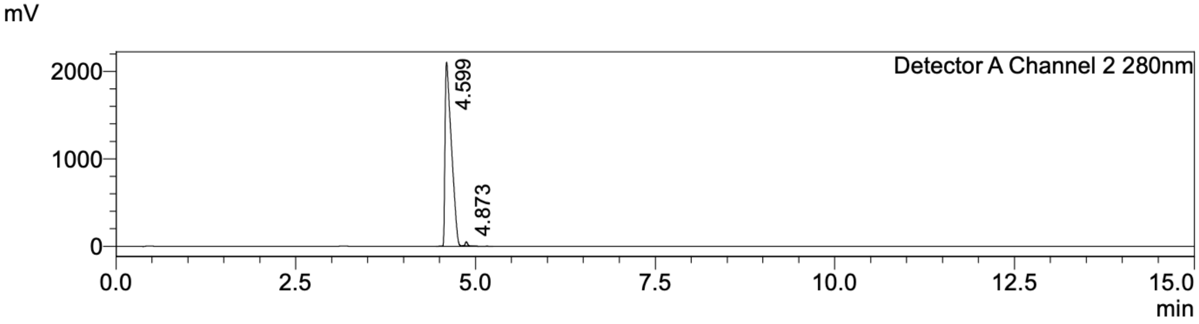

<Peak Table>

Detector A Channel 1 254nm

Peak#	Ret. Time	Height	Width at 5% Height	Area	Area%
1	4.598	2155601	0.195	13175861	99.234
2	4.872	44061	0.072	101766	0.766
Total		2199662		13277627	100.000

Detector A Channel 2 280nm

Peak#	Ret. Time	Height	Width at 5% Height	Area	Area%
1	4.599	2097249	0.196	13004468	99.126
2	4.873	49254	0.073	114668	0.874
Total		2146503		13119136	100.000

HPLC analysis of T27

<Chromatogram>

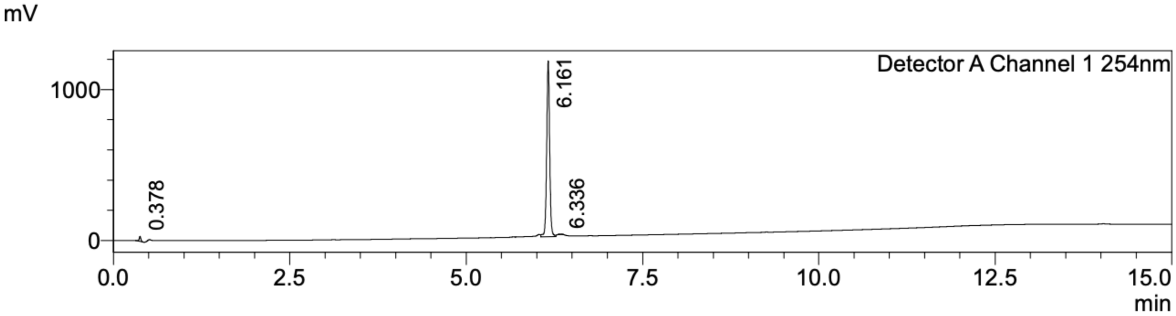

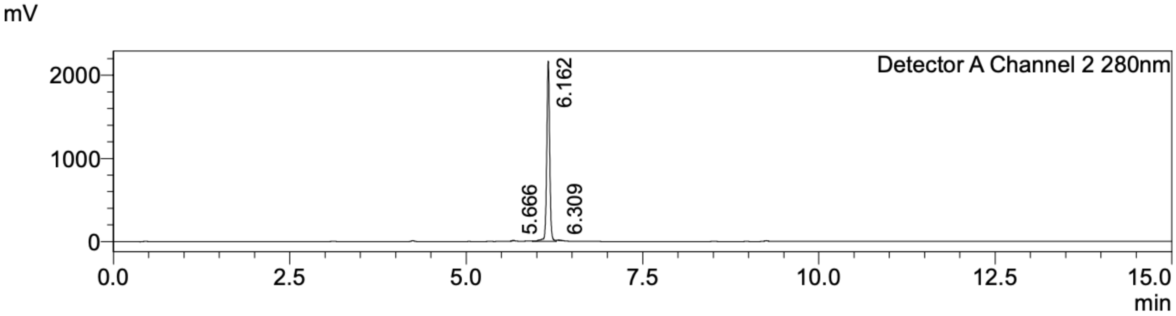

<Peak Table>

Detector A Channel 1 254nm

Peak#	Ret. Time	Height	Width at 5% Height	Area	Area%
1	0.378	31892	0.075	56140	1.611
2	6.161	1145958	0.101	3418867	98.083
3	6.336	3701	--	10676	0.306
Total		1181552		3485683	100.000

Detector A Channel 2 280nm

Peak#	Ret. Time	Height	Width at 5% Height	Area	Area%
1	5.666	4181	--	7423	0.117
2	6.162	2140425	0.099	6346668	99.878
3	6.309	83	--	315	0.005
Total		2144689		6354405	100.000

HPLC analysis of T28

<Chromatogram>

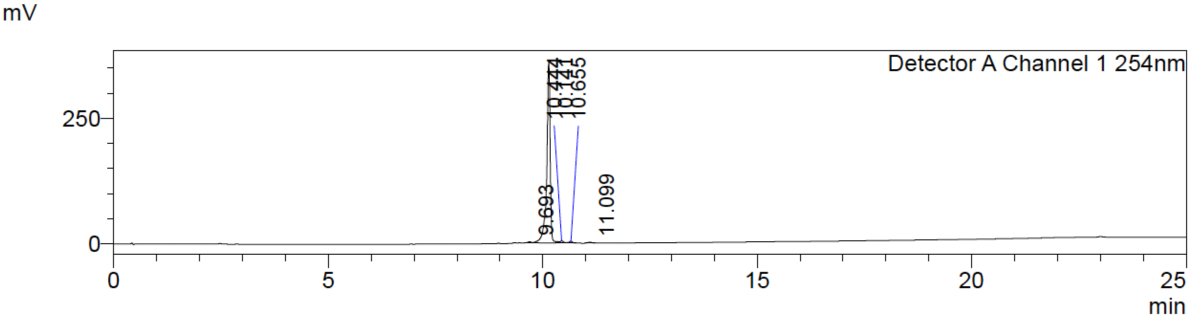

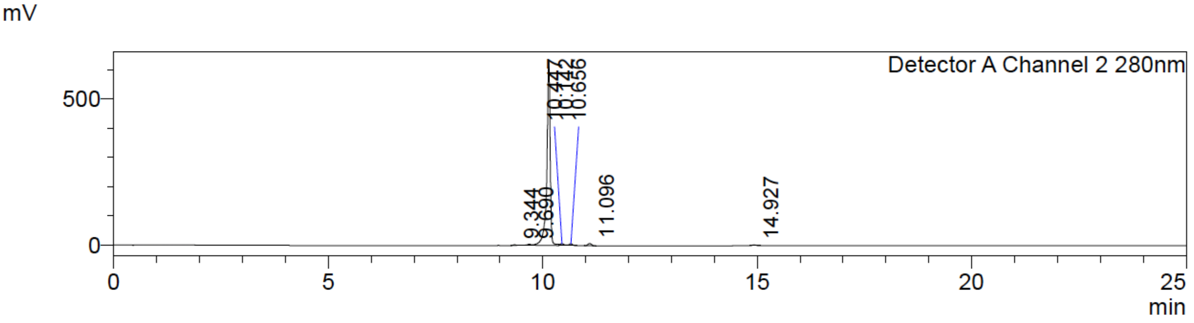

<Peak Table>

Detector A Channel 1 254nm

Peak#	Ret. Time	Height	Width at 5% Height	Area	Area%
1	9.693	2208	--	10610	0.514
2	10.141	360276	0.232	1981500	96.072
3	10.444	4281	--	35959	1.743
4	10.655	3998	--	21995	1.066
5	11.099	2237	0.178	12454	0.604
Total		373000		2062519	100.000

Detector A Channel 2 280nm

Peak#	Ret. Time	Height	Width at 5% Height	Area	Area%
1	9.344	2171	0.127	8458	0.237
2	9.690	3327	--	19074	0.534
3	10.142	621126	0.228	3409014	95.522
4	10.447	6267	--	37056	1.038
5	10.656	6552	--	36705	1.028
6	11.096	7621	0.189	43509	1.219
7	14.927	2352	0.206	15026	0.421
Total		649416		3568842	100.000

HPLC analysis of T29

<Chromatogram>

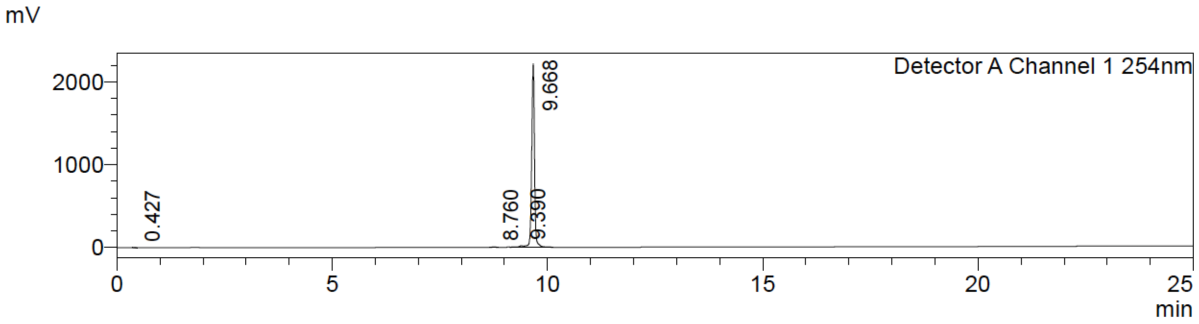

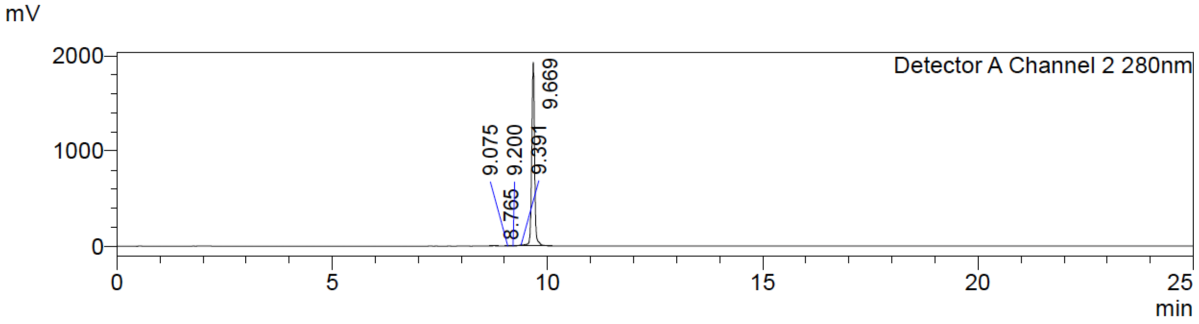

<Peak Table>

Detector A Channel 1 254nm

Peak#	Ret. Time	Height	Width at 5% Height	Area	Area%
1	0.427	3718	0.080	8859	0.087
2	8.760	2663	0.152	13586	0.134
3	9.390	16077	--	115004	1.132
4	9.668	2184198	0.156	10025243	98.648
Total		2206657		10162692	100.000

Detector A Channel 2 280nm

Peak#	Ret. Time	Height	Width at 5% Height	Area	Area%
1	8.765	3283	0.156	16711	0.184
2	9.075	1118	--	6564	0.072
3	9.200	1375	--	12740	0.140
4	9.391	12410	--	79709	0.876
5	9.669	1917469	0.161	8985691	98.729
Total		1935654		9101414	100.000

HPLC analysis of T30

<Chromatogram>

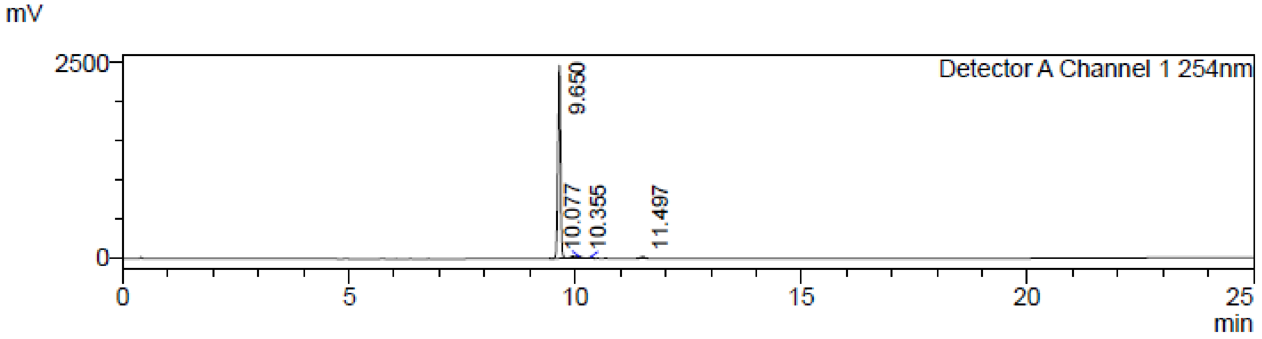

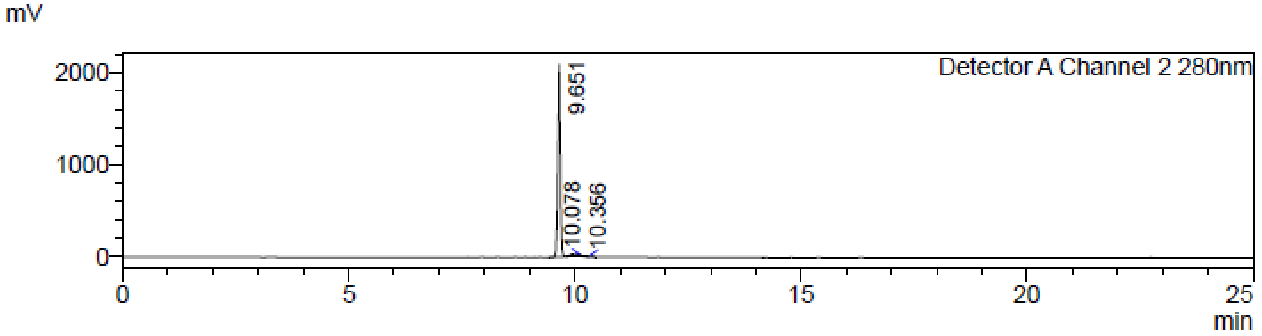

<Peak Table>

Detector A Channel 1 254nm

Peak#	Ret. Time	Height	Width at 5% Height	Area	Area%
1	9.650	2436311	0.139	10044690	95.549
2	10.077	22943	0.325	276051	2.626
3	10.355	17291	0.120	67257	0.640
4	11.497	21997	0.178	124628	1.186
Total		2498542		10512626	100.000

Detector A Channel 2 280nm

Peak#	Ret. Time	Height	Width at 5% Height	Area	Area%
1	9.651	2052692	0.140	8653793	95.562
2	10.078	26697	0.325	323349	3.571
3	10.356	19759	0.125	78533	0.867
Total		2099148		9055675	100.000

HPLC analysis of T31

<Chromatogram>

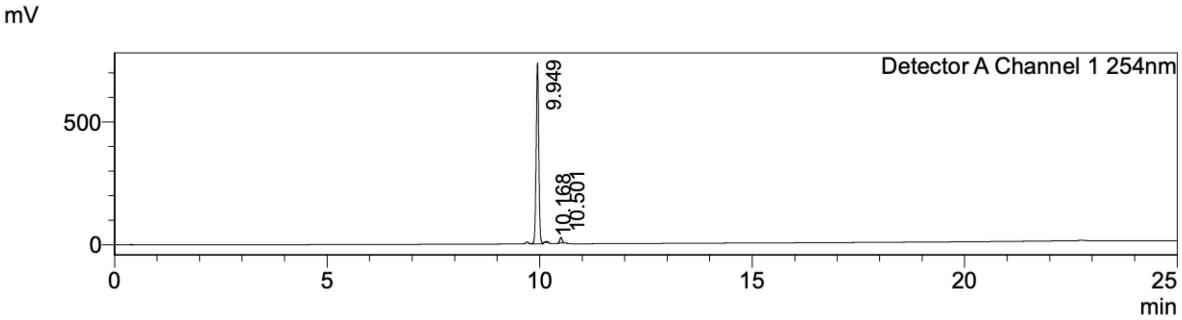

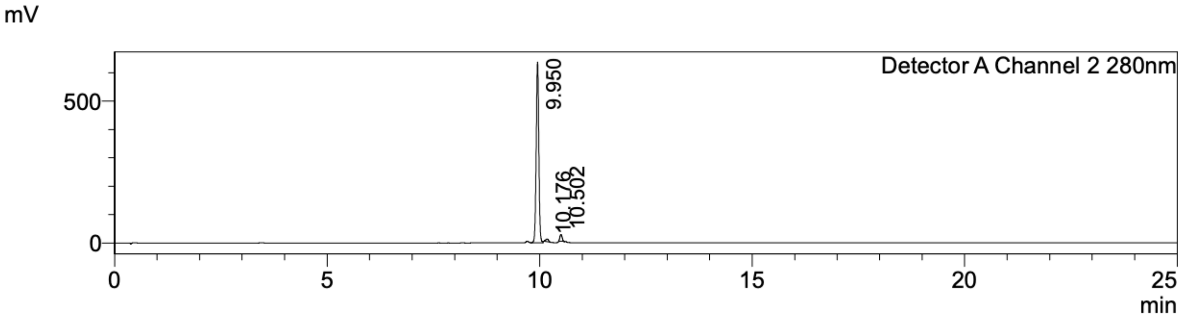

<Peak Table>

Detector A Channel 1 254nm

Peak#	Ret. Time	Height	Width at 5% Height	Area	Area%
1	9.949	730989	0.143	3118064	96.793
2	10.168	4132	--	15440	0.479
3	10.501	22052	0.119	87868	2.728
Total		757172		3221371	100.000

Detector A Channel 2 280nm

Peak#	Ret. Time	Height	Width at 5% Height	Area	Area%
1	9.950	631528	0.143	2706859	95.377
2	10.176	9236	0.129	41707	1.470
3	10.502	24110	0.107	89499	3.154
Total		664874		2838065	100.000

HPLC analysis of T32

<Chromatogram>

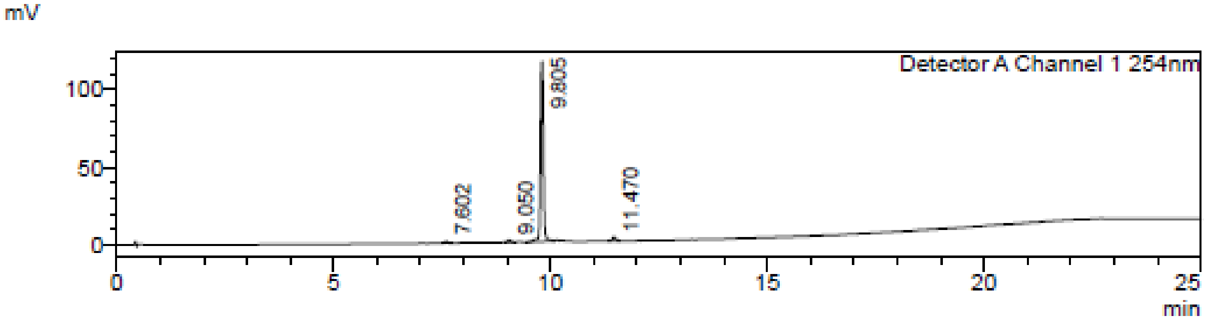

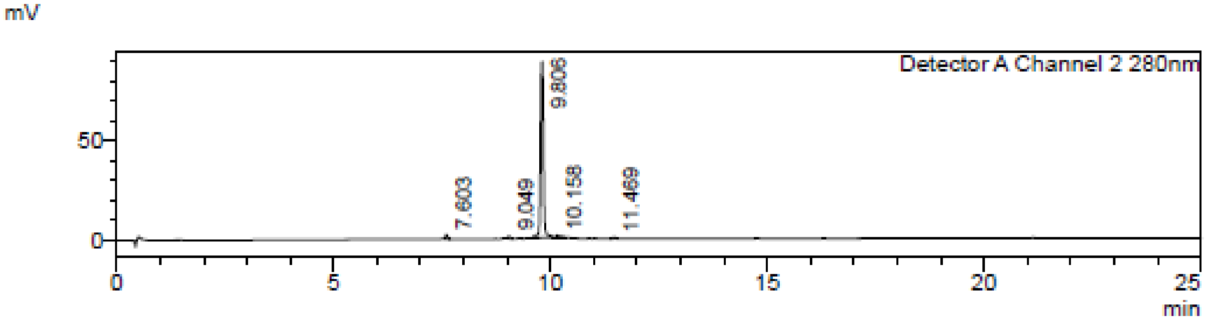

<Peak Table>

Detector A Channel 1 254nm

Peak#	Ret. Time	Height	Width at 5% Height	Area	Area%
1	7.602	1255	0.116	4544	0.825
2	9.050	1447	0.146	6158	1.119
3	9.805	115280	0.150	524386	95.263
4	11.470	2625	0.194	15372	2.793
Total		120606		550459	100.000

Detector A Channel 2 280nm

Peak#	Ret. Time	Height	Width at 5% Height	Area	Area%
1	7.603	2110	0.104	7037	1.647
2	9.049	976	0.115	3625	0.849
3	9.806	88513	0.152	405490	94.931
4	10.158	994	0.244	8344	1.953
5	11.469	541	0.145	2645	0.619
Total		93134		427141	100.000

HPLC analysis of T33

<Chromatogram>

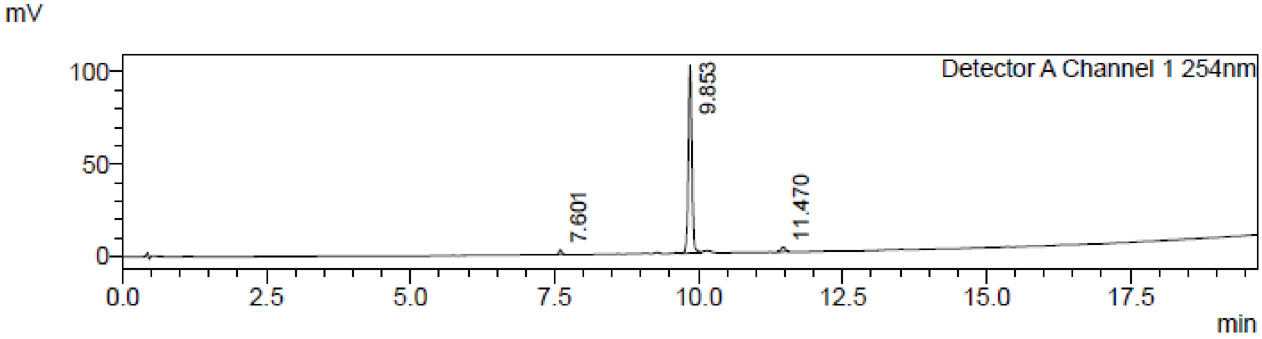

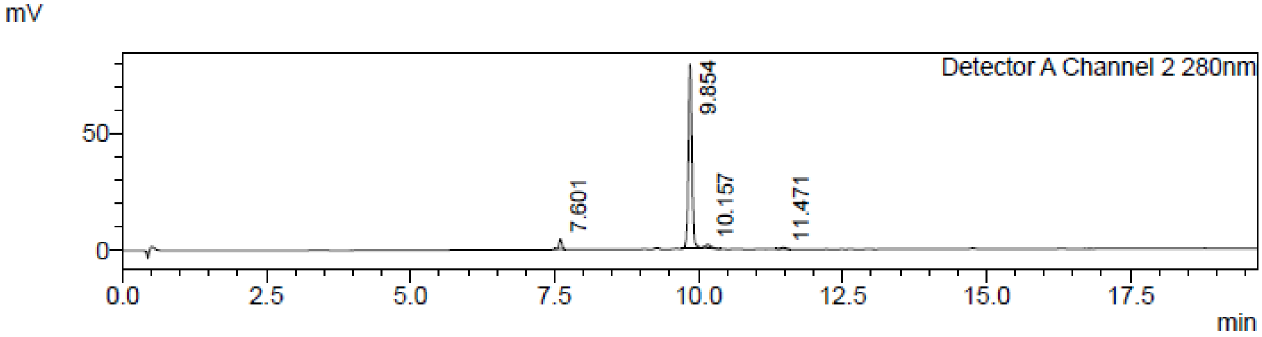

<Peak Table>

Detector A Channel 1 254nm

Peak#	Ret. Time	Height	Width at 5% Height	Area	Area%
1	7.601	2395	0.106	8167	1.763
2	9.853	100755	0.148	442572	95.531
3	11.470	2339	0.163	12538	2.706
Total		105489		463277	100.000

Detector A Channel 2 280nm

Peak#	Ret. Time	Height	Width at 5% Height	Area	Area%
1	7.601	4269	0.109	14894	4.003
2	9.854	78455	0.149	345566	92.878
3	10.157	1280	0.211	8392	2.255
4	11.471	580	0.172	3214	0.864
Total		84584		372065	100.000

HPLC analysis of T34

<Chromatogram>

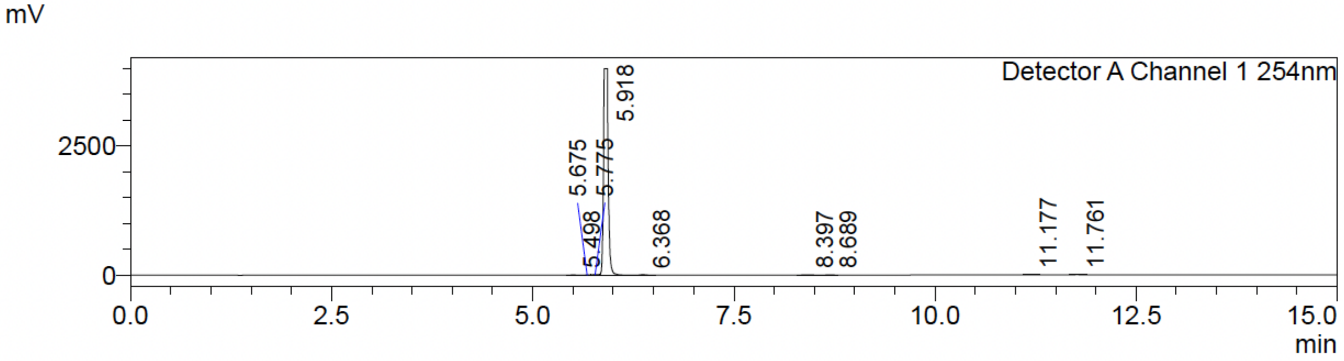

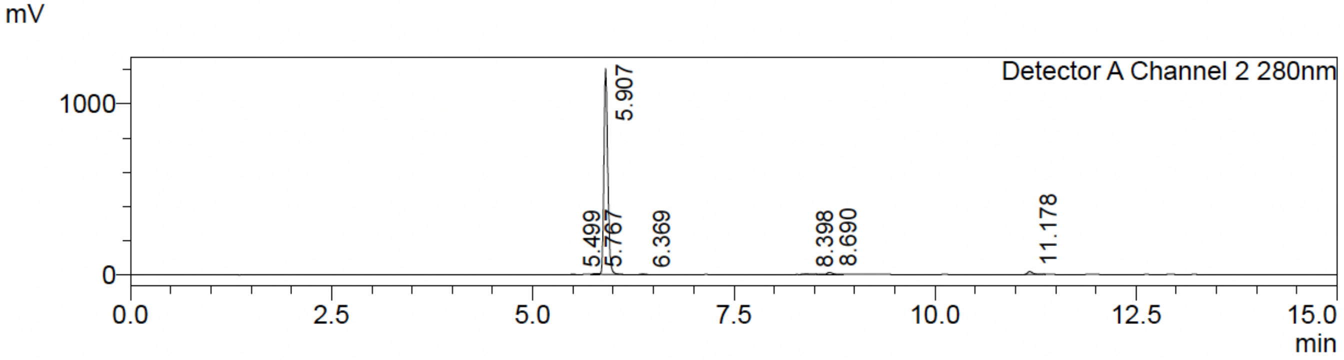

<Peak Table>

Detector A Channel 1 254nm

Peak#	Ret. Time	Height	Width at 5% Height	Area	Area%
1	5.498	8897	--	31272	0.191
2	5.675	4637	--	26273	0.160
3	5.775	17026	--	90800	0.554
4	5.918	3997509	0.116	16085083	98.179
5	6.368	12471	--	57230	0.349
6	8.397	7501	0.193	39495	0.241
7	8.689	2225	--	11376	0.069
8	11.177	2816	0.151	12195	0.074
9	11.761	6677	0.151	29664	0.181
Total		4059759		16383387	100.000

Detector A Channel 2 280nm

Peak#	Ret. Time	Height	Width at 5% Height	Area	Area%
1	5.499	1932	0.098	5734	0.137
2	5.767	4114	--	20729	0.496
3	5.907	1194639	0.114	3993503	95.531
4	6.369	3236	0.141	13192	0.316

Peak#	Ret. Time	Height	Width at 5% Height	Area	Area%
5	8.398	2819	0.164	13886	0.332
6	8.690	11978	0.165	54373	1.301
7	11.178	17697	0.163	78907	1.888
Total		1236416		4180324	100.000

HRMS spectrum of T9

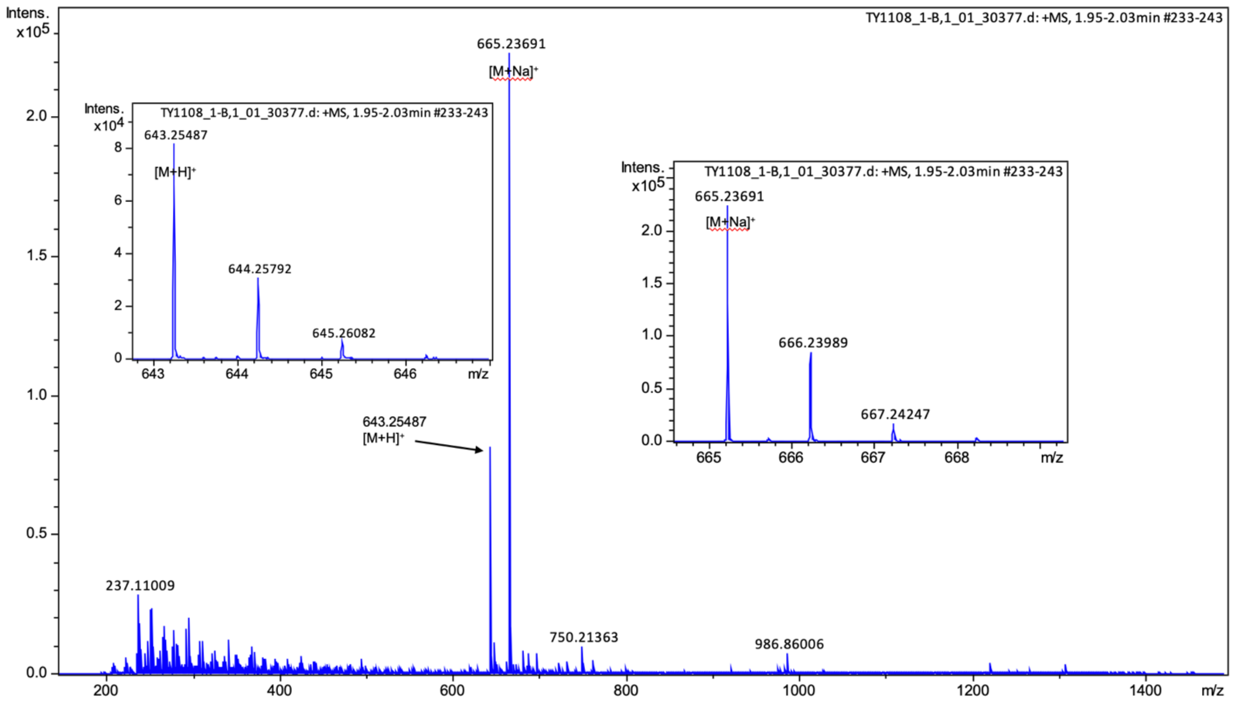

HRMS spectrum of T34

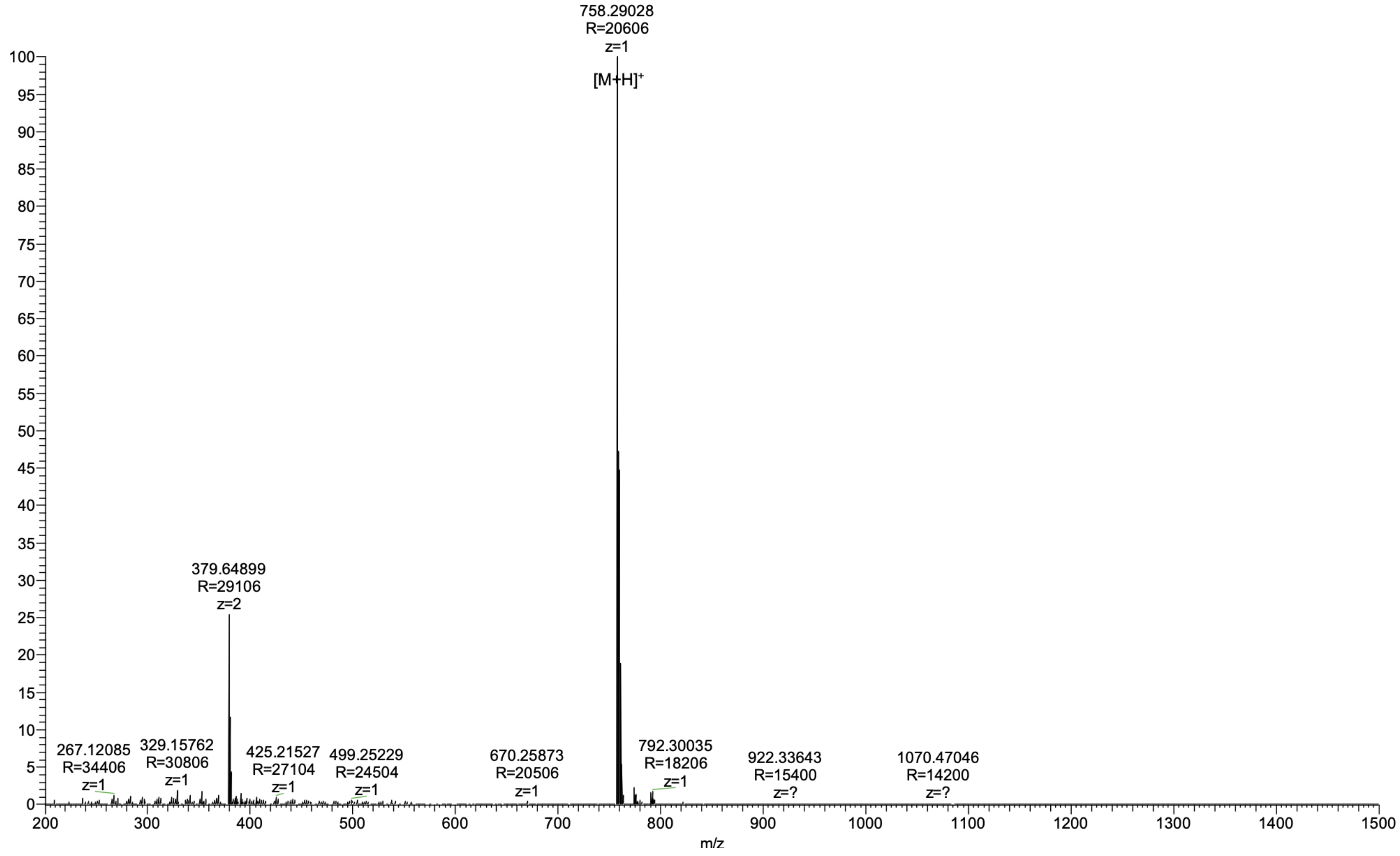

NMR spectra of degraders

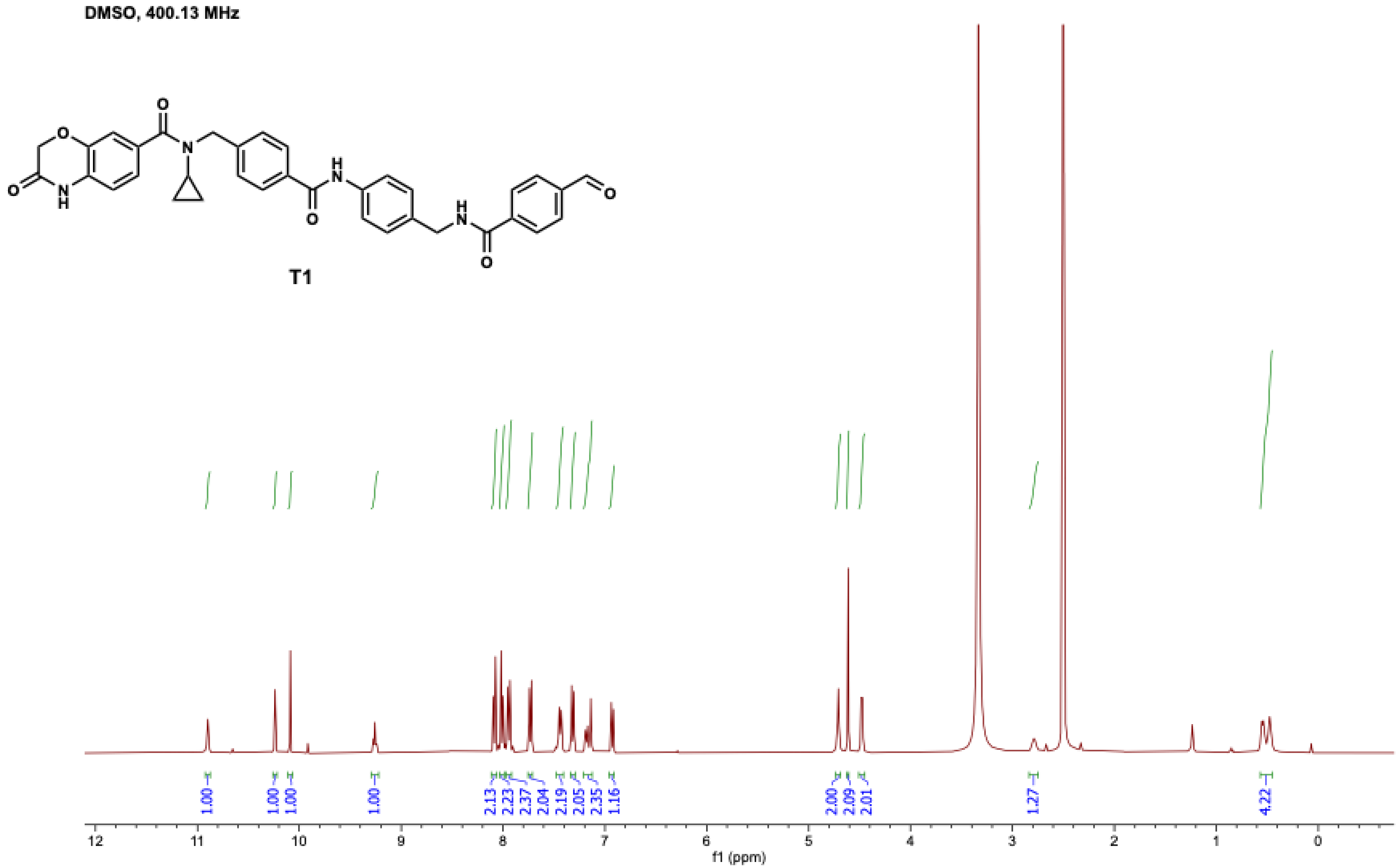

^{1}H NMR spectrum of T1

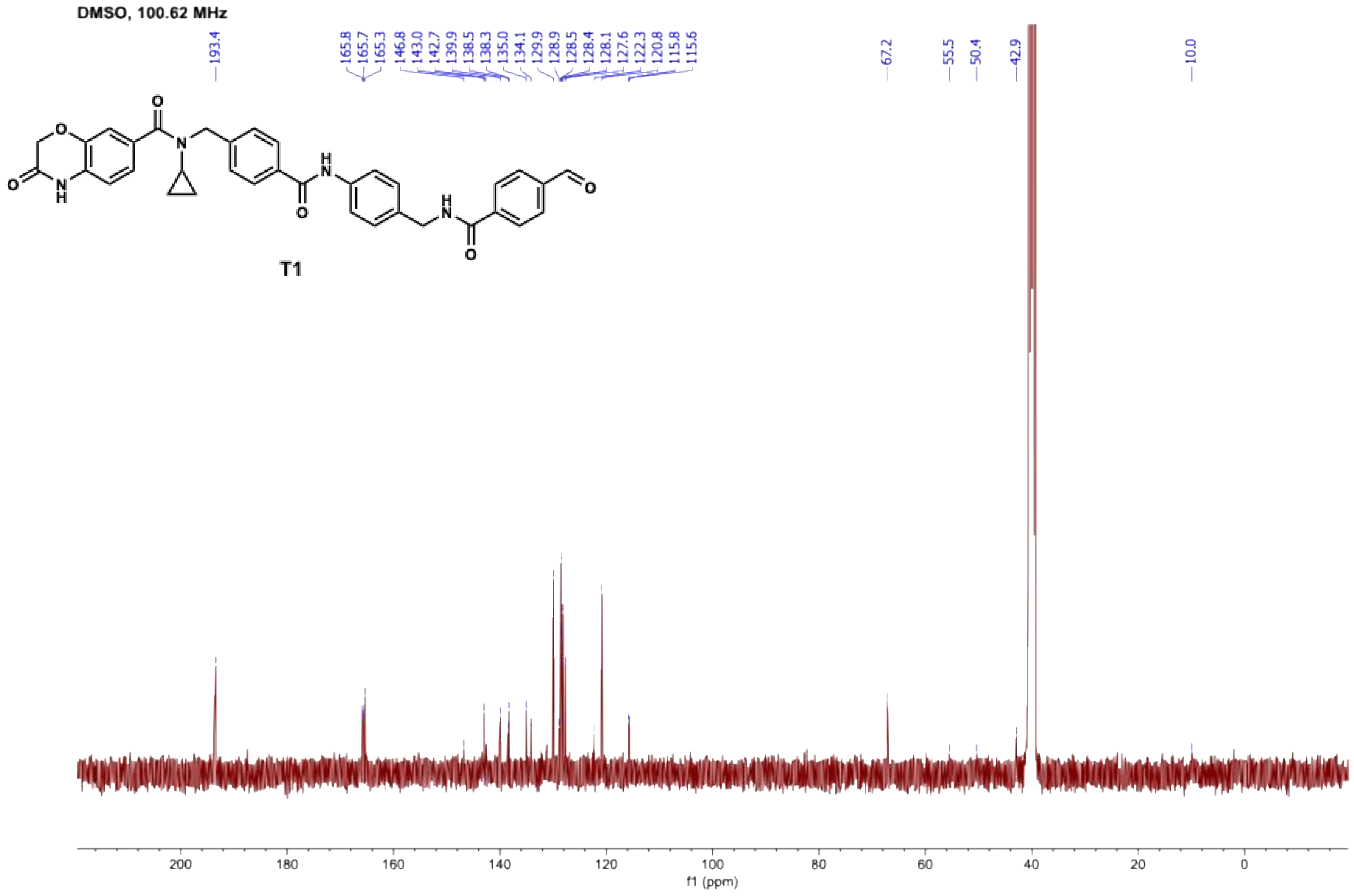

^{13}C NMR spectrum of T1

DMSO, 400.13 MHz

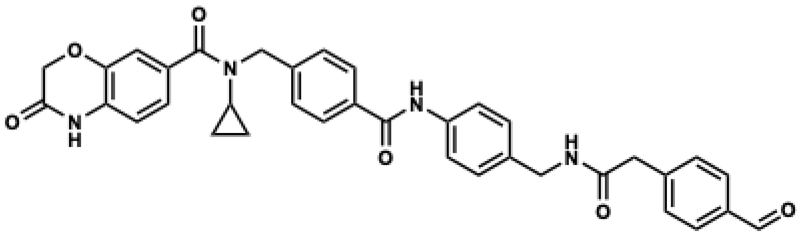

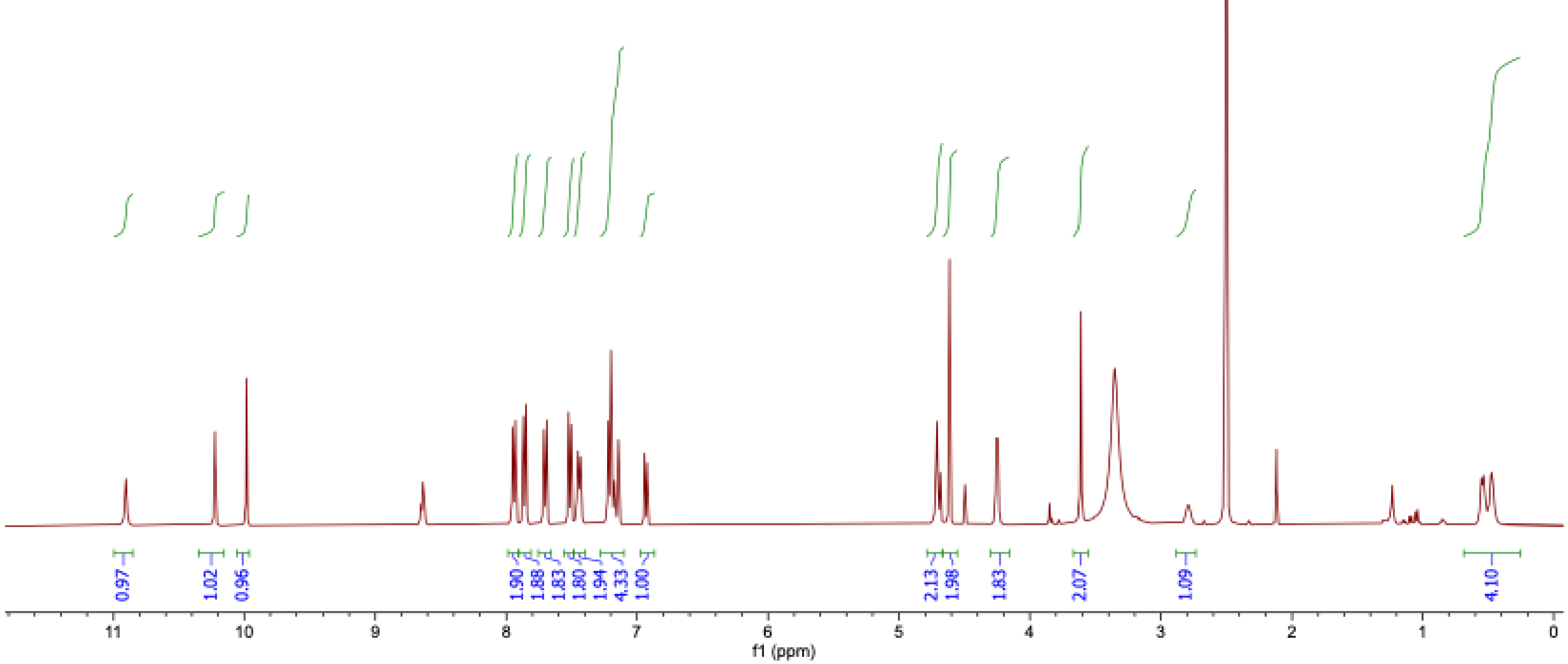

^{1}H NMR spectrum of T2

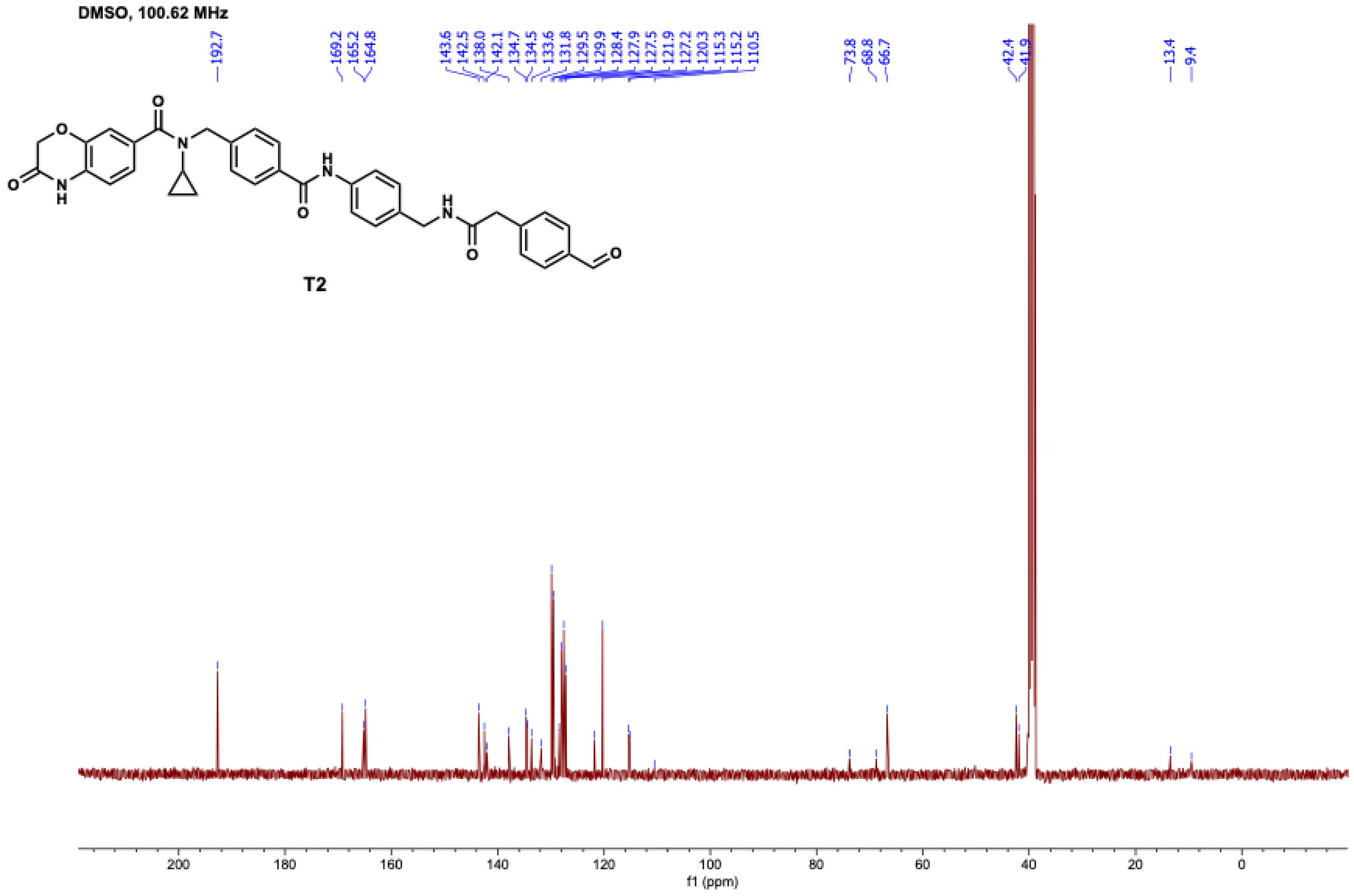

^{13}C NMR spectrum of T2

DMSO, 400.13 MHz

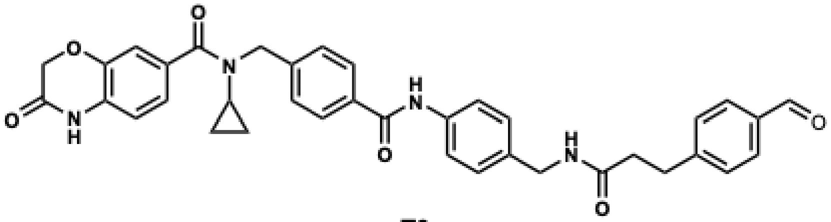

T3

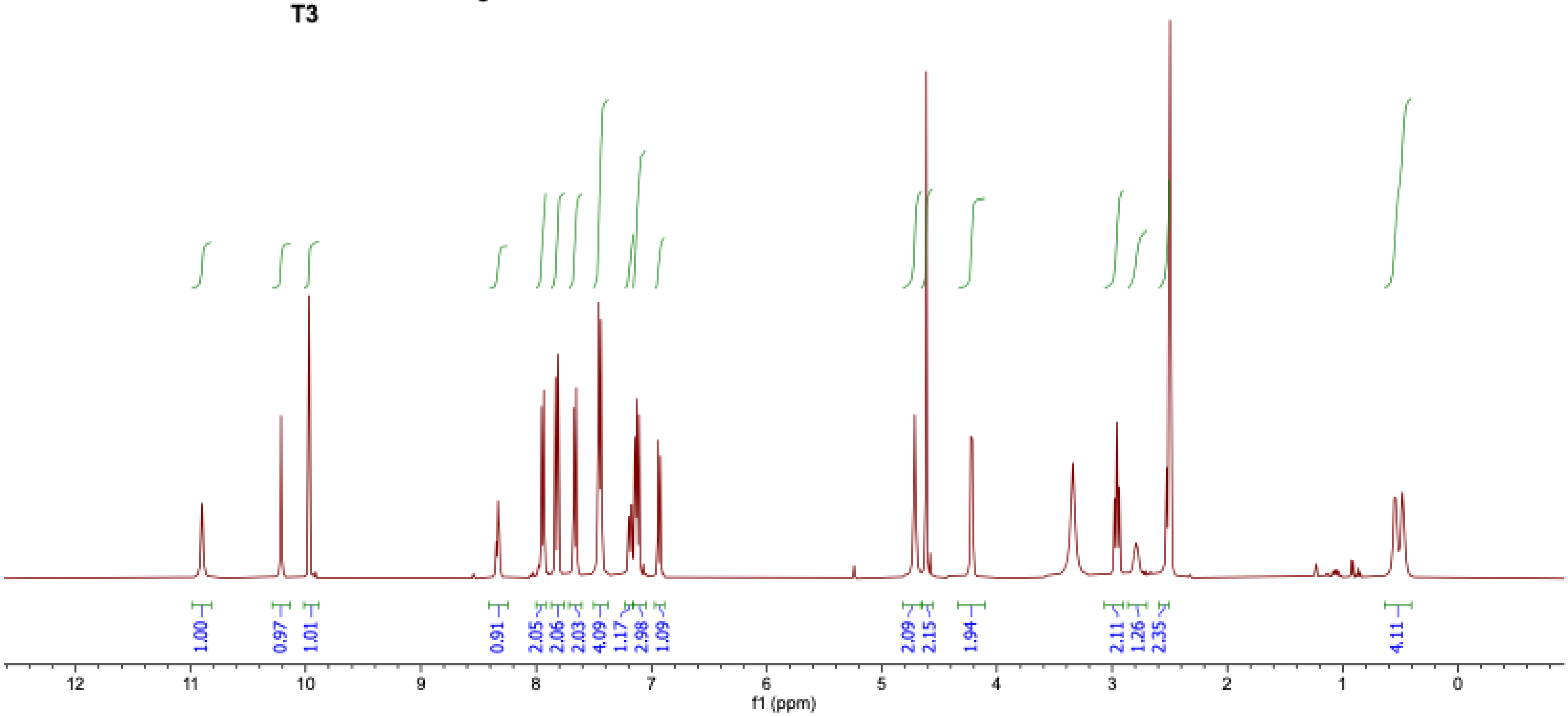

^{1}H NMR spectrum of T3

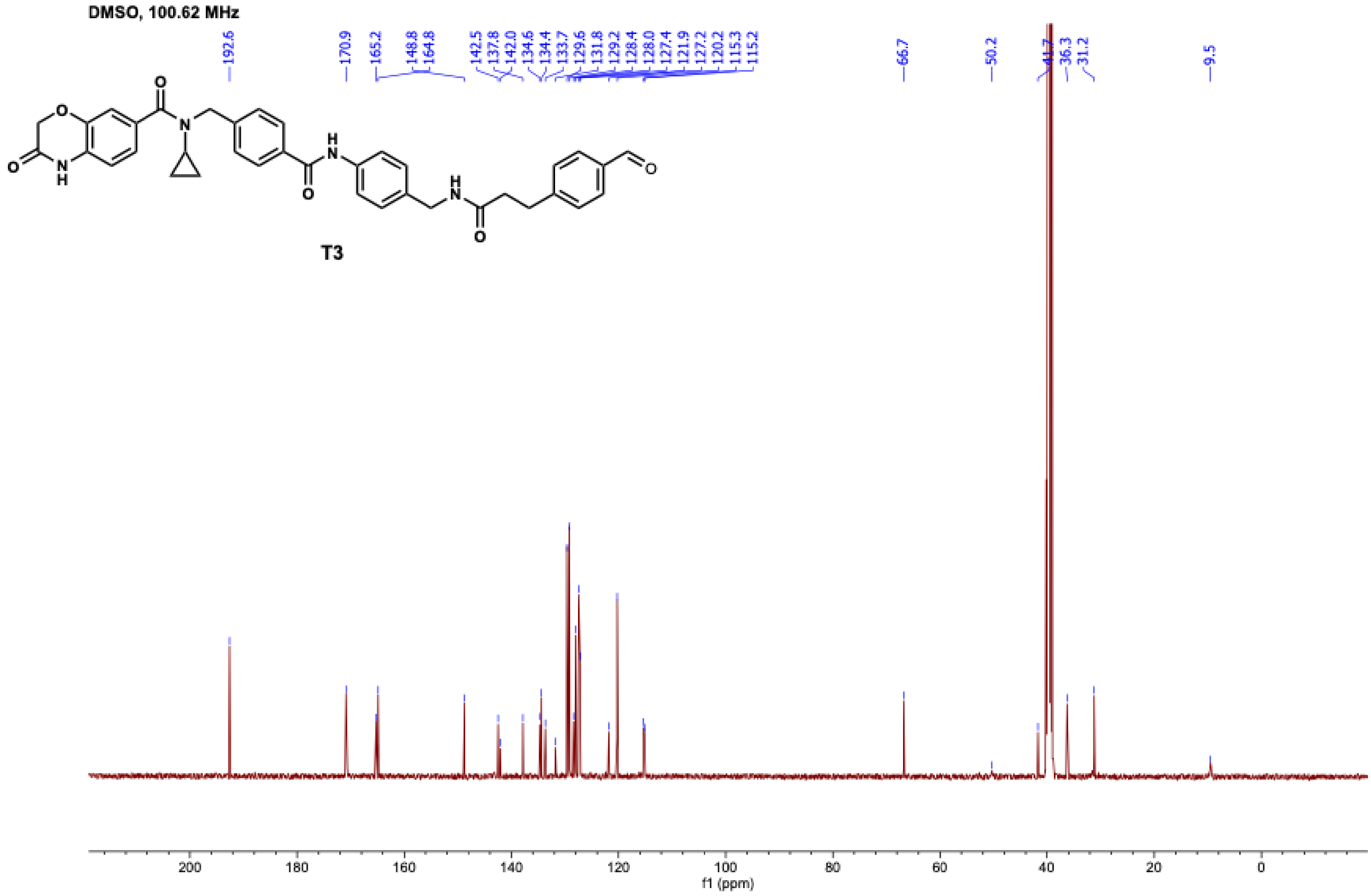

^{13}C NMR spectrum of T3

DMSO, 400.13 MHz

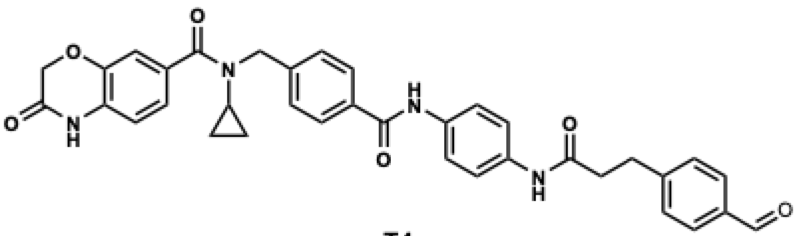

T4

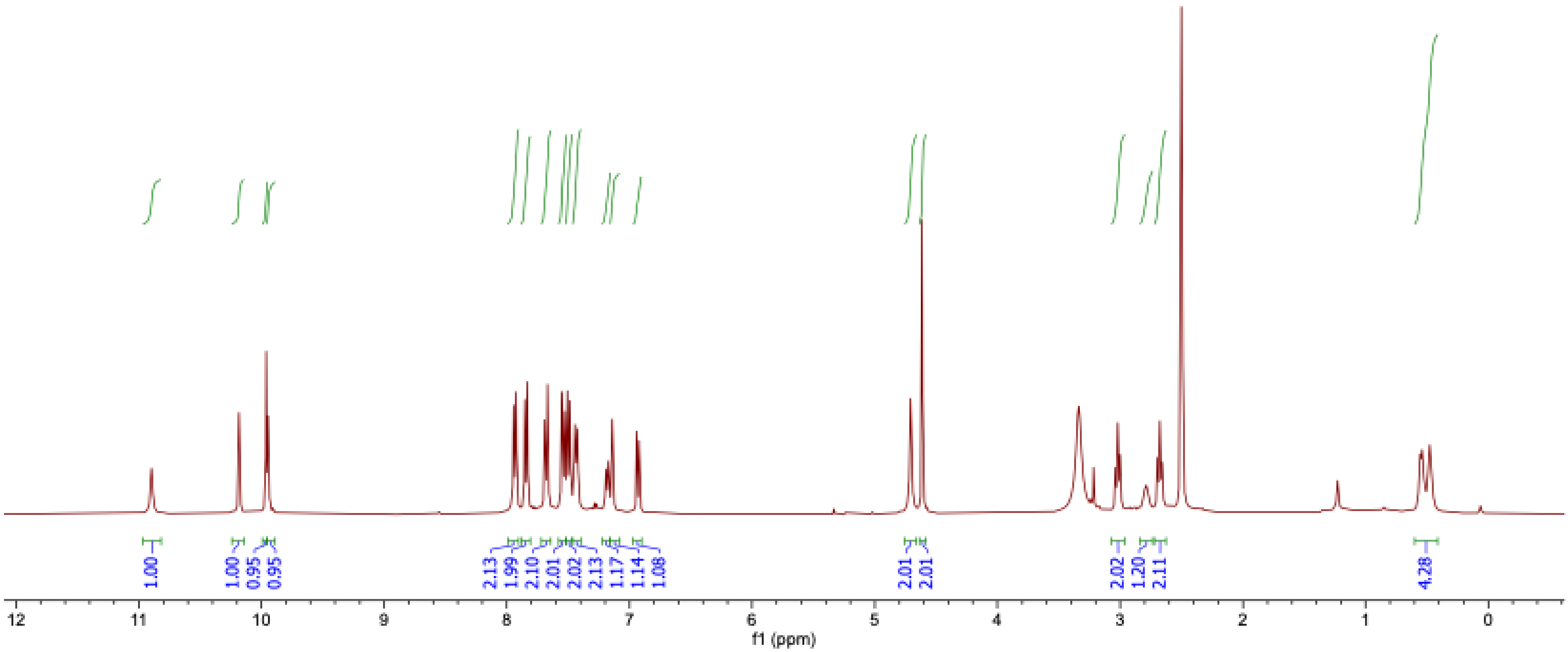

^{1}H NMR spectrum of T4

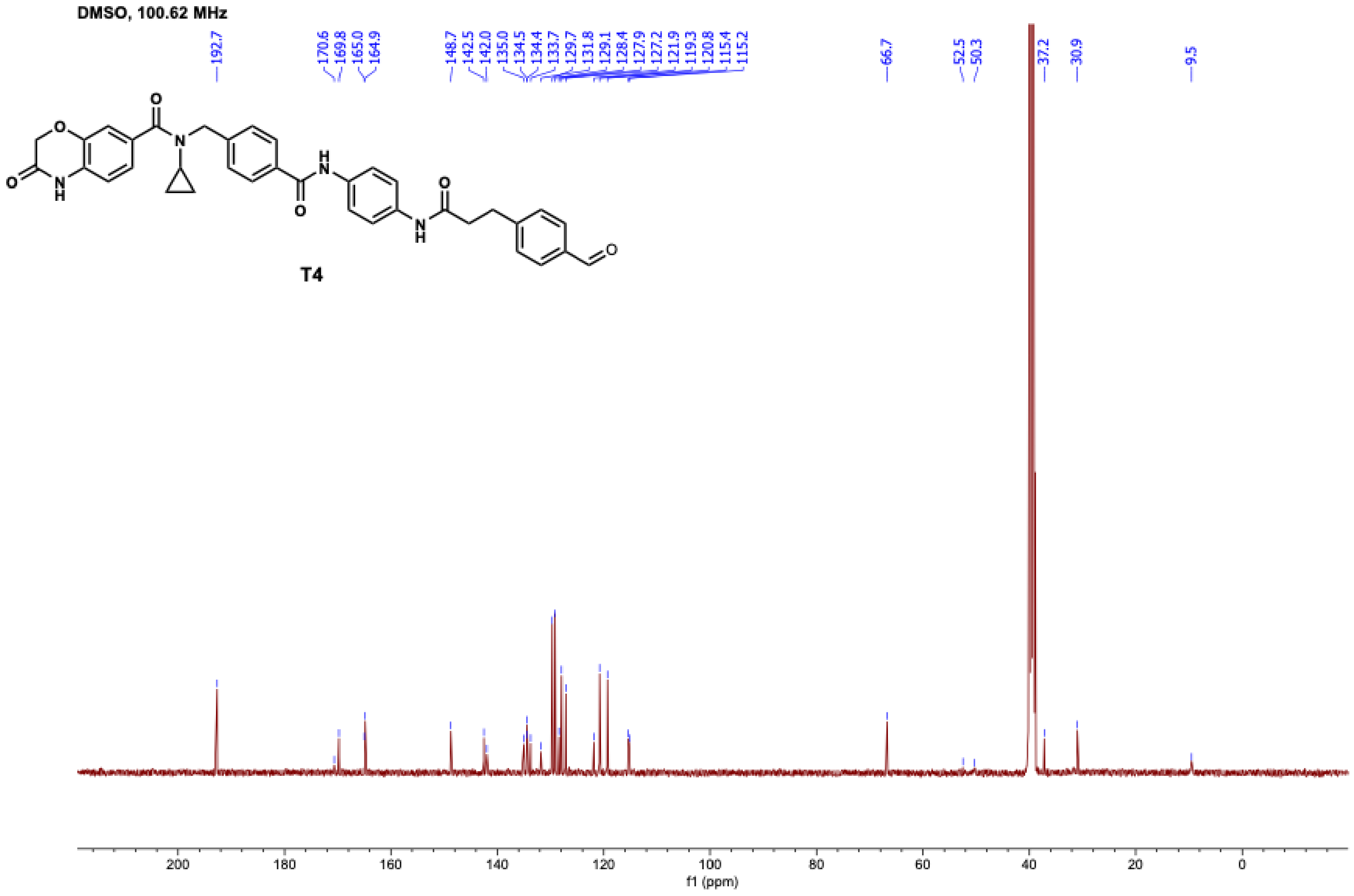

^{13}C NMR spectrum of T4

DMSO, 400.13 MHz

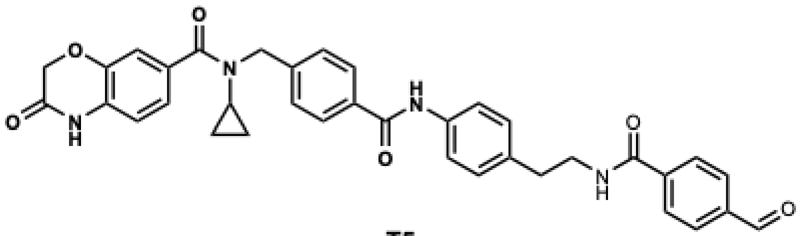

T5

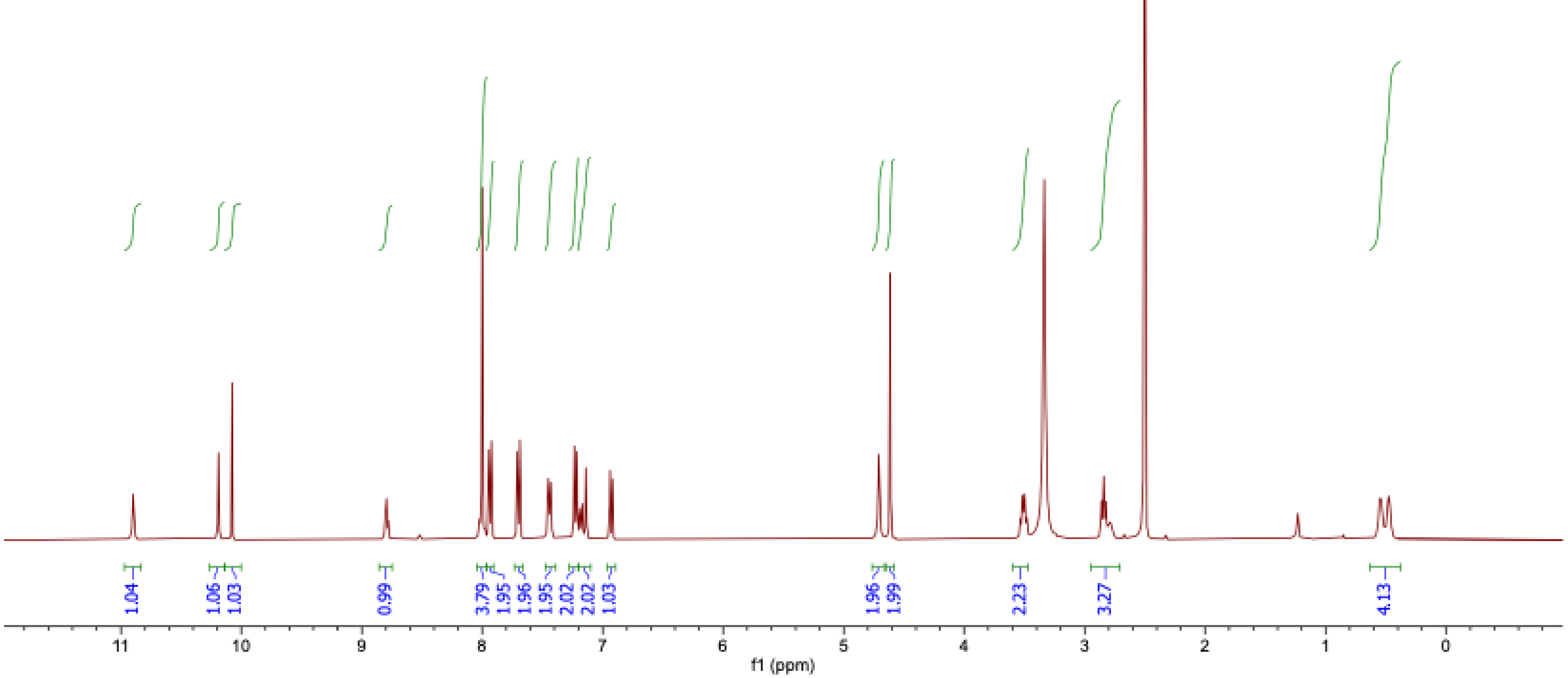

^{1}H NMR spectrum of T5

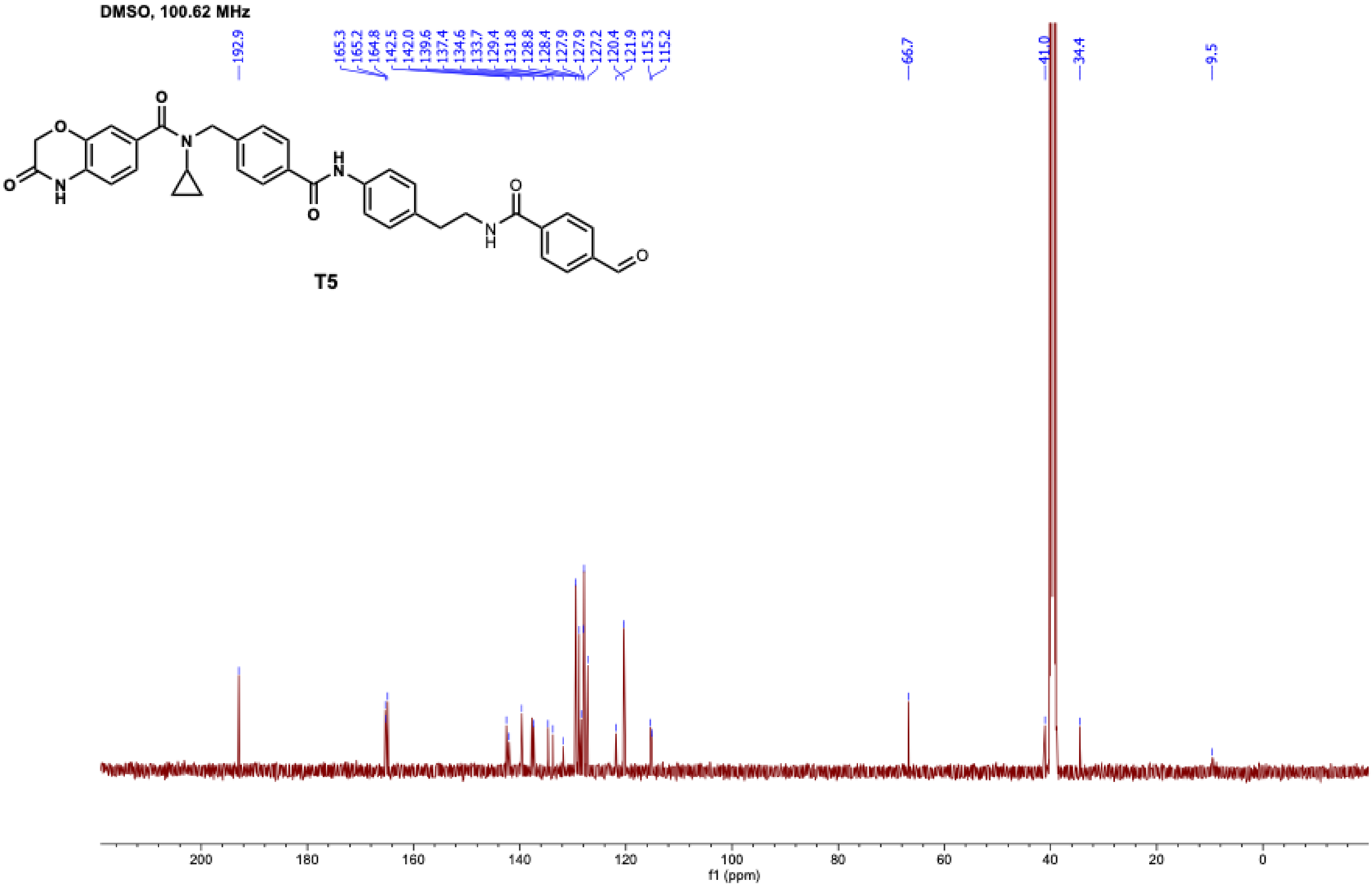

T5

^{13}C NMR spectrum of T5

DMSO, 400.13 MHz

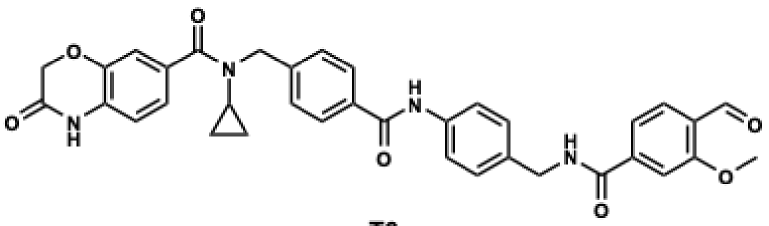

T6

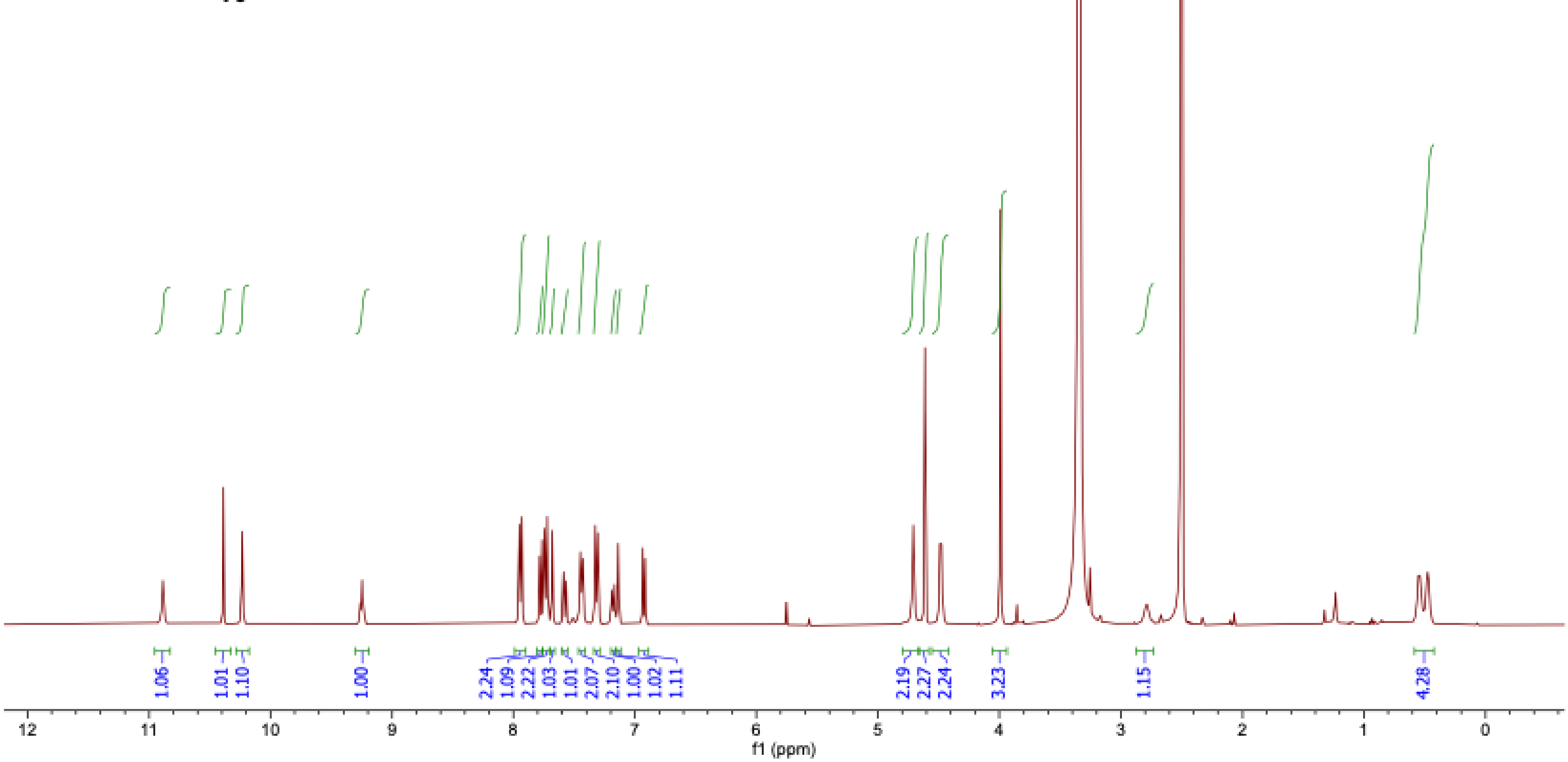

^{1}H NMR spectrum of T6

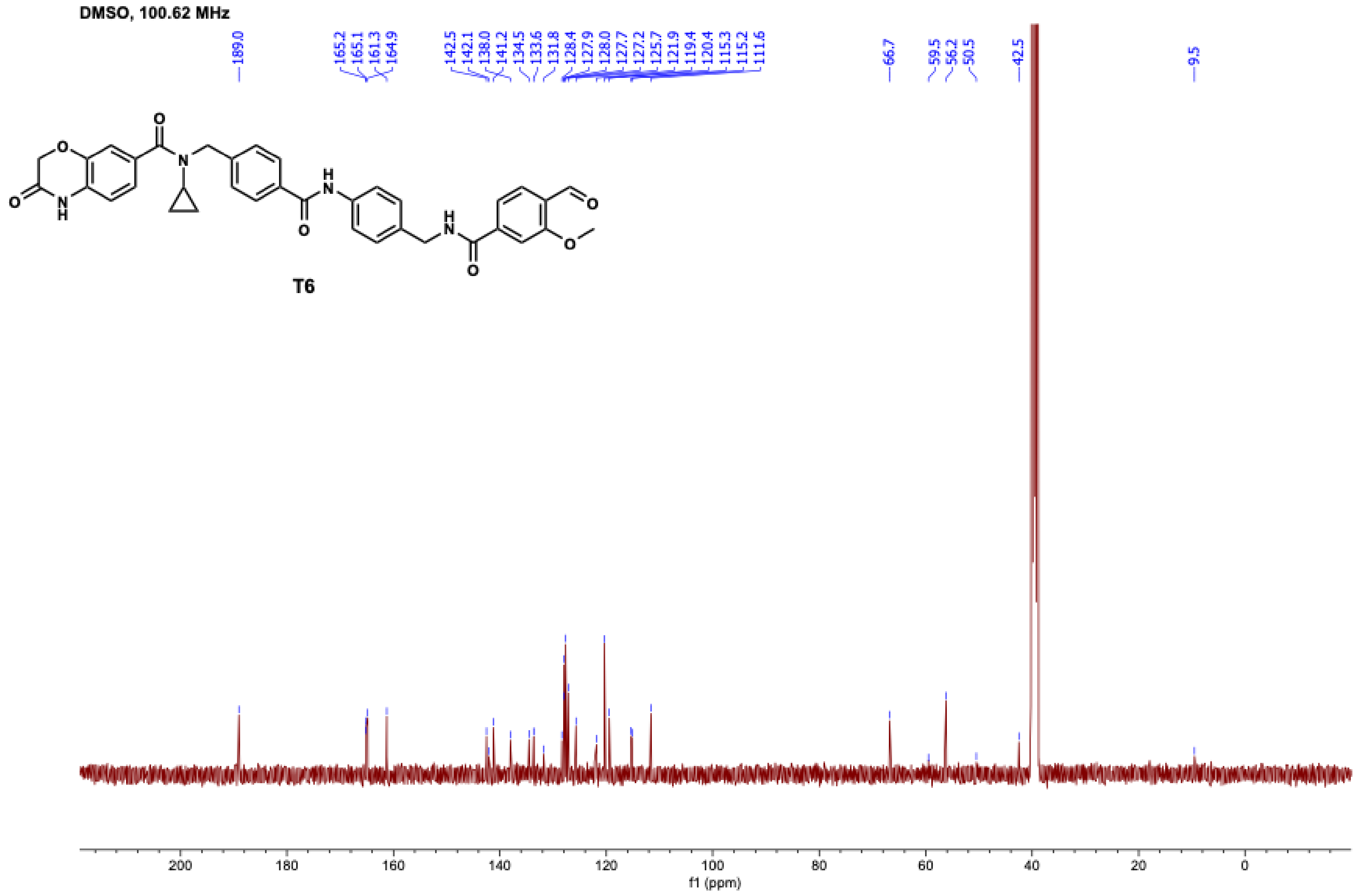

^{13}C NMR spectrum of T6

DMSO, 400.13 MHz

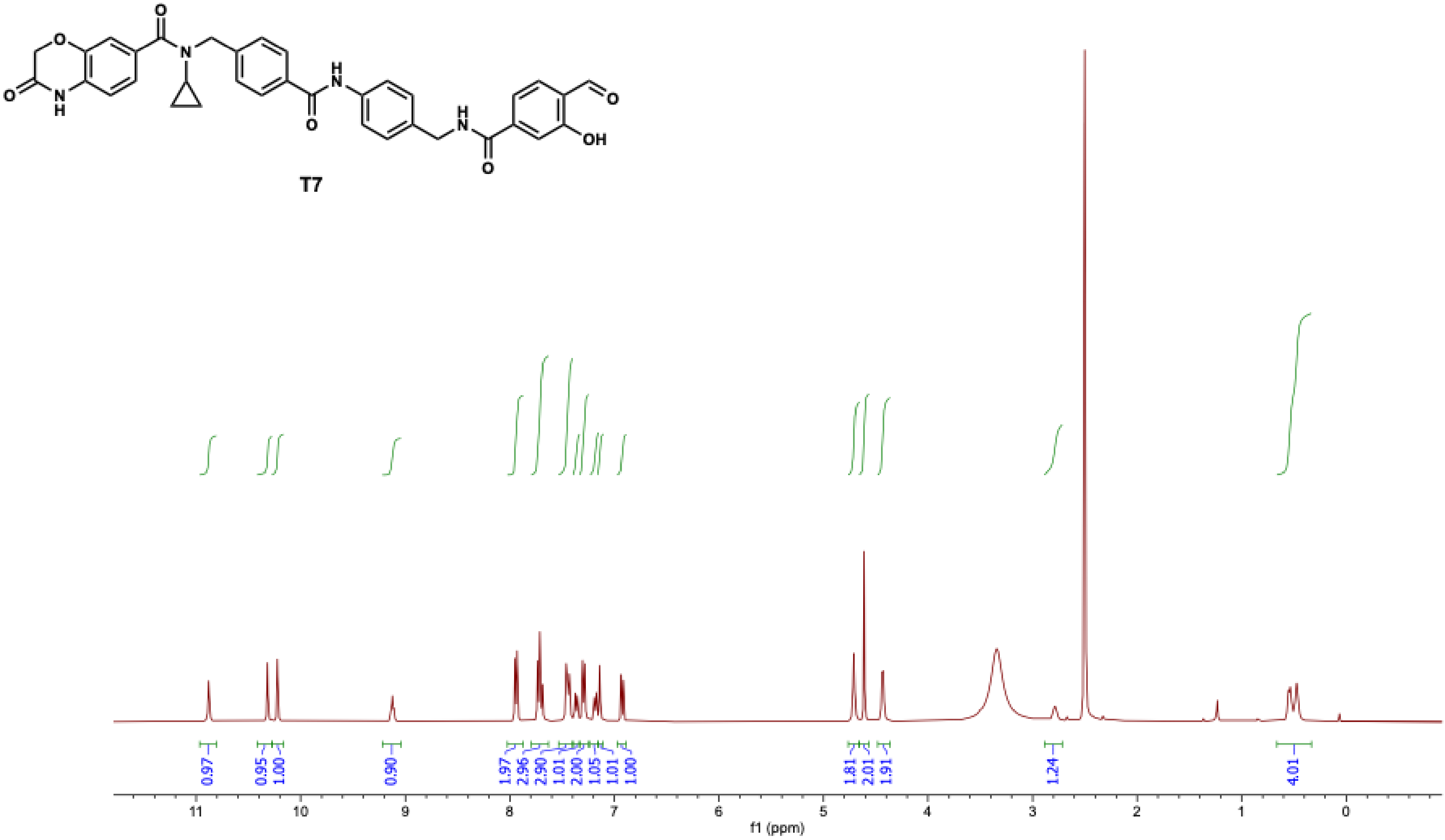

^{1}H NMR spectrum of T7

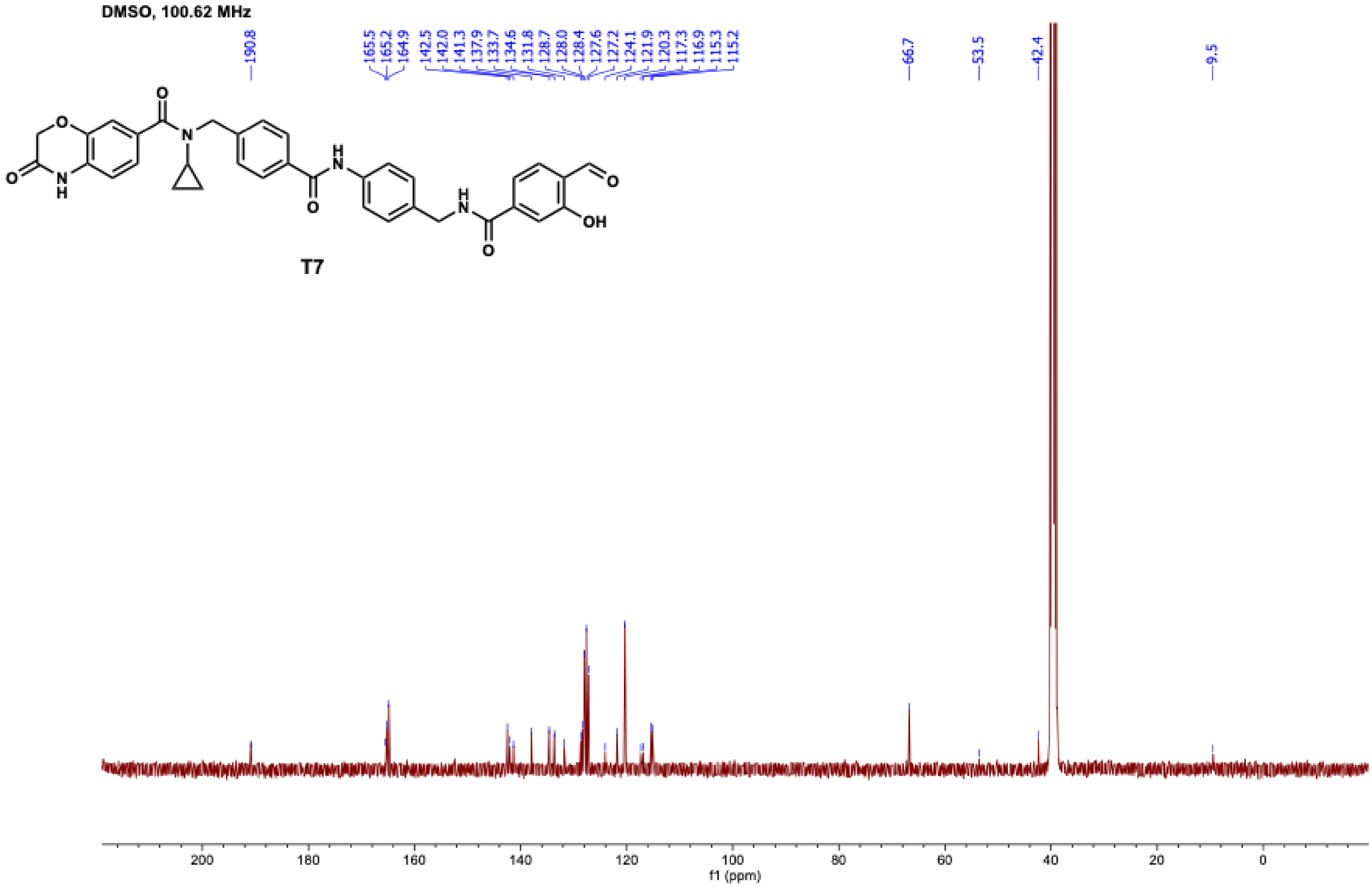

^{13}C NMR spectrum of T7

DMSO, 400.13 MHz

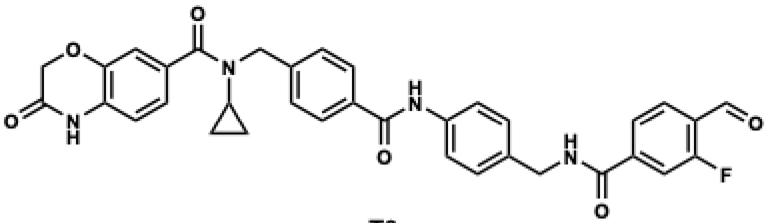

T8

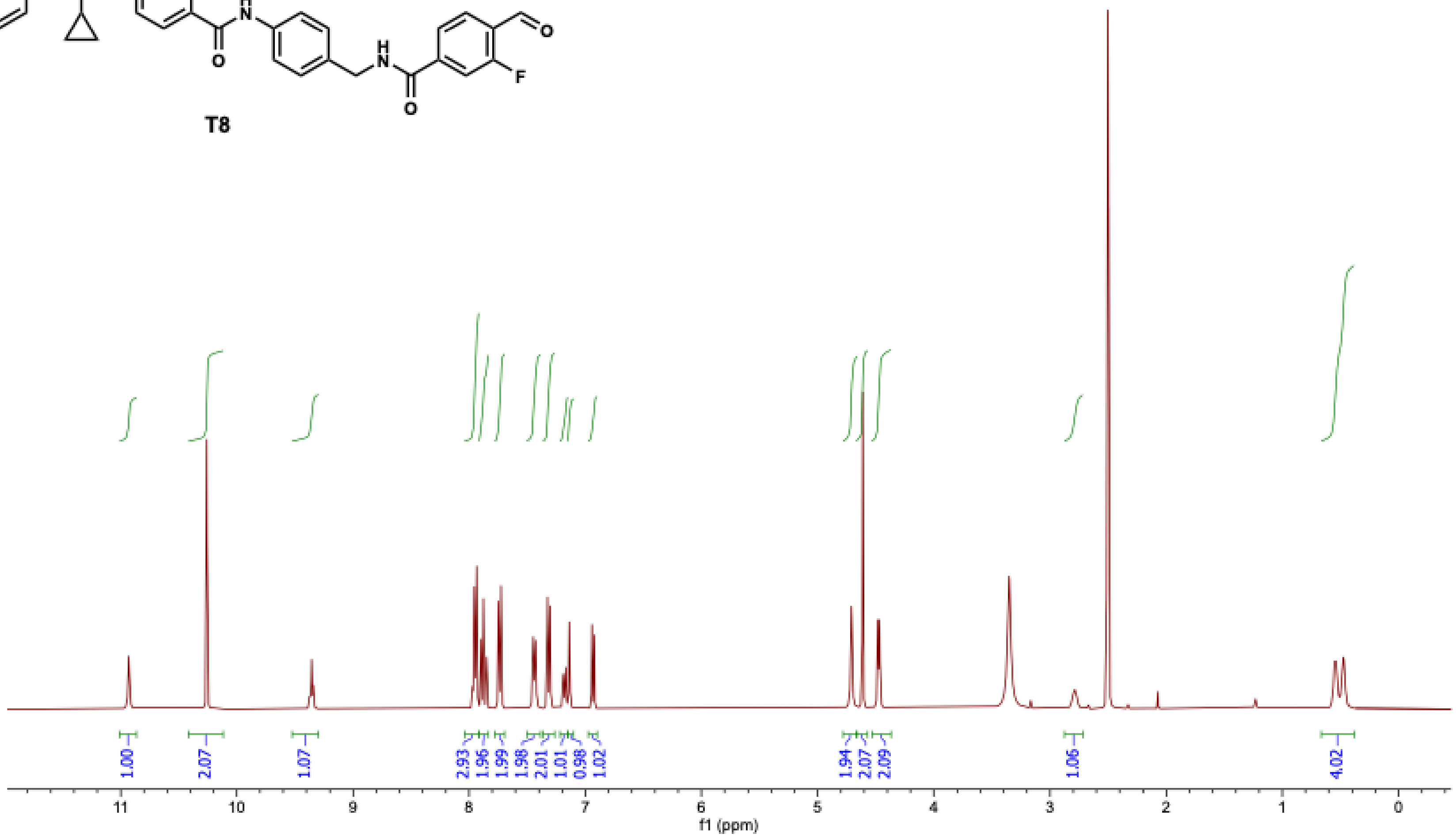

^{1}H NMR spectrum of T8

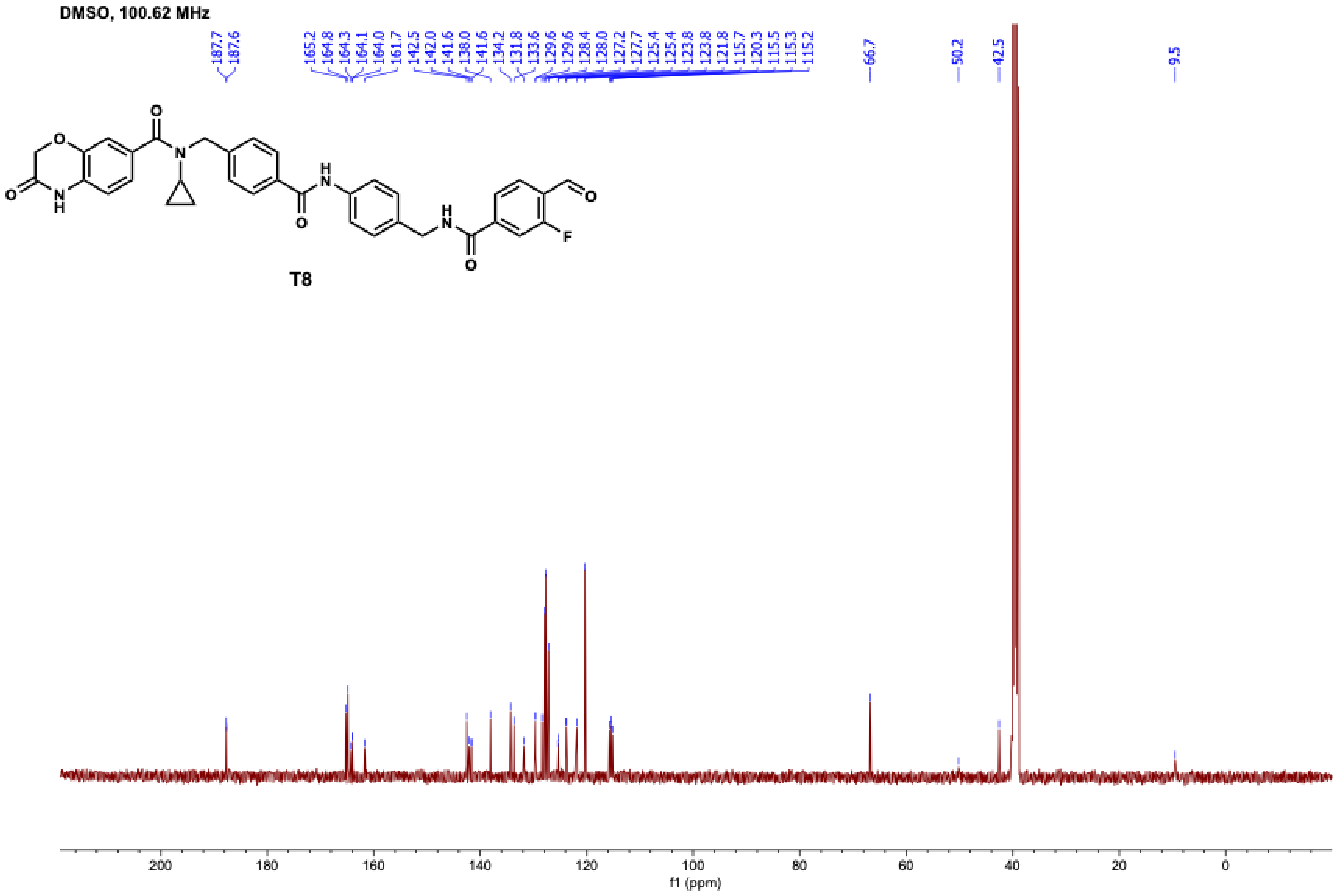

^{13}C NMR spectrum of T8

DMSO, 400.13 MHz

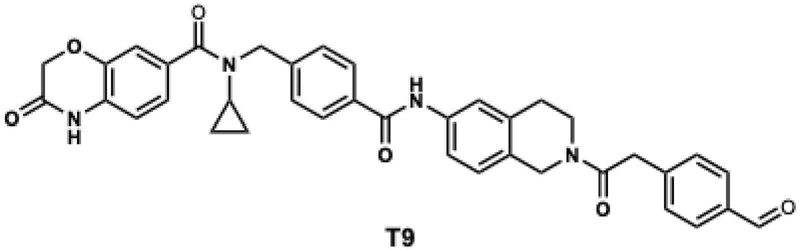

T9

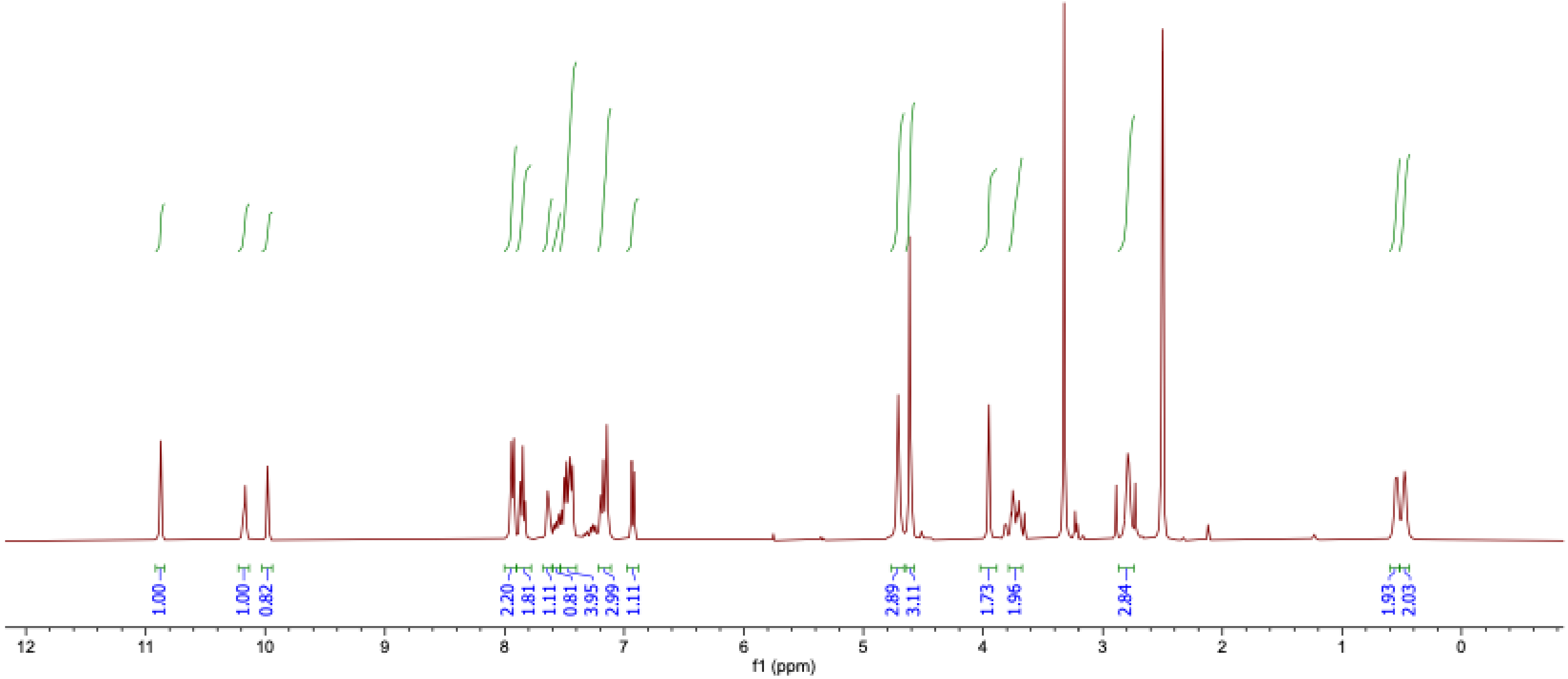

^{1}H NMR spectrum of T9

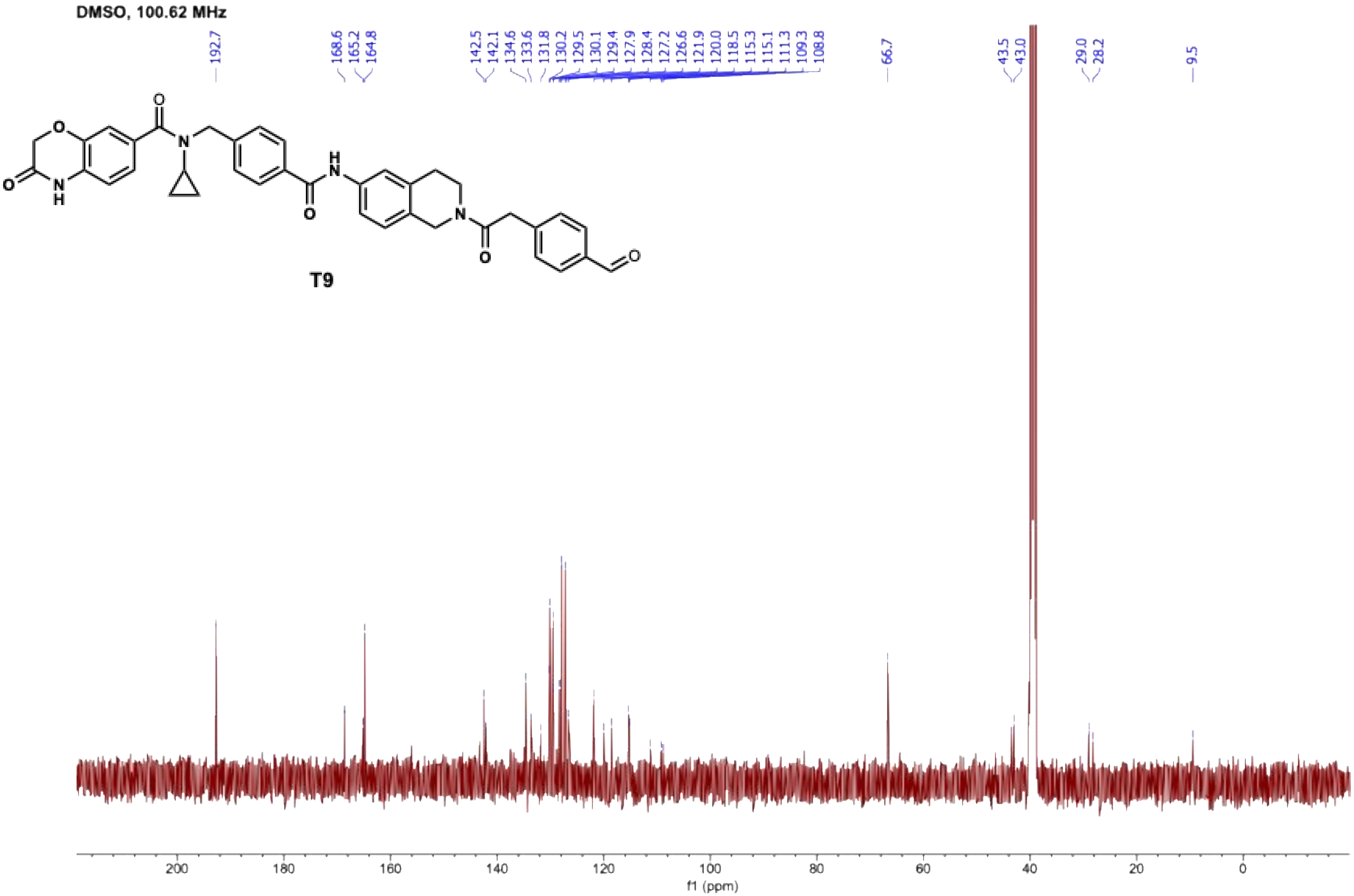

^{13}C NMR spectrum of T9

DMSO, 400.13 MHz

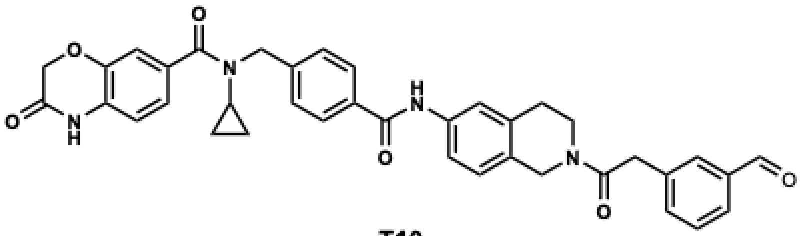

T10

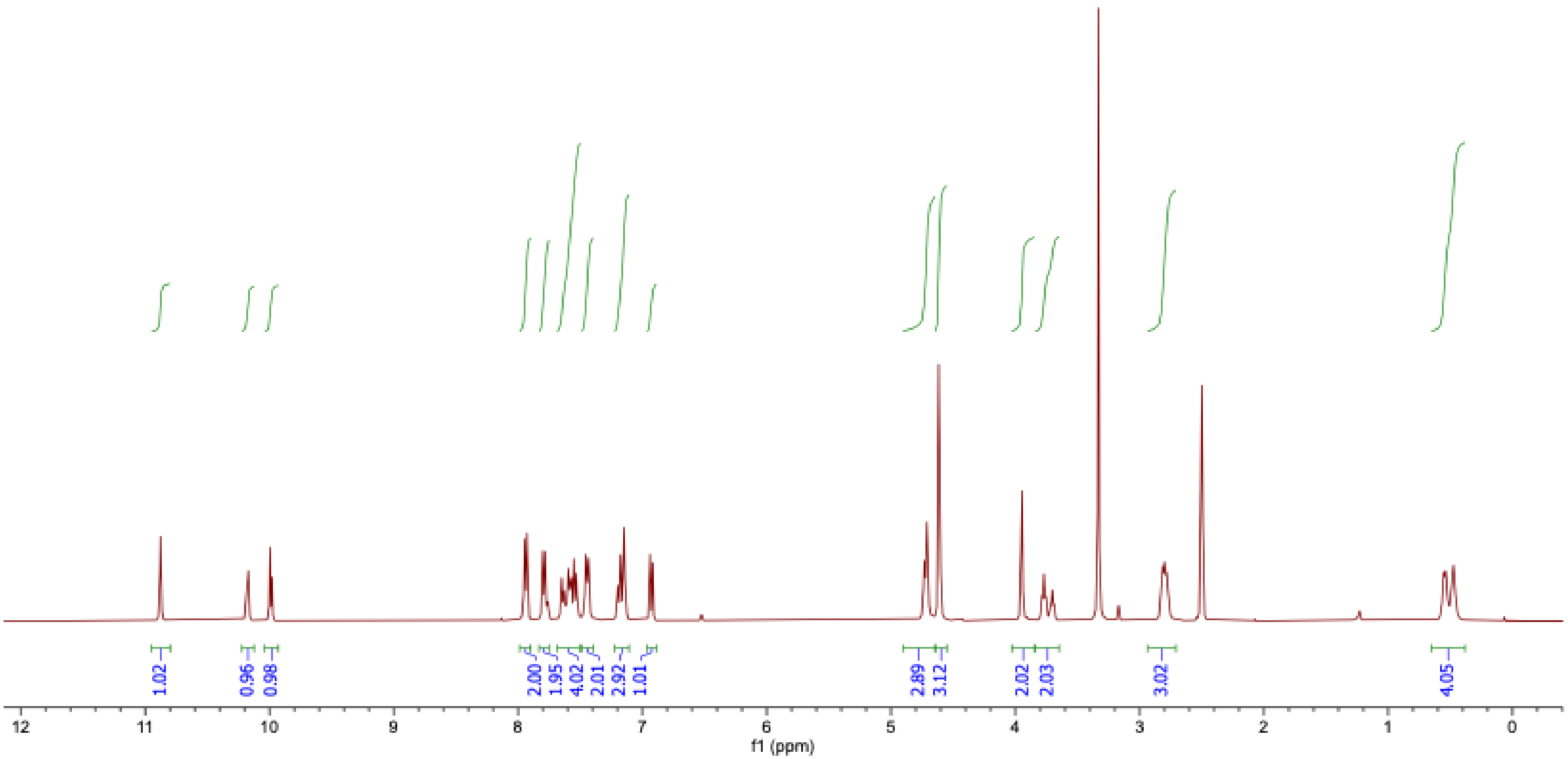

¹H NMR spectrum of T10

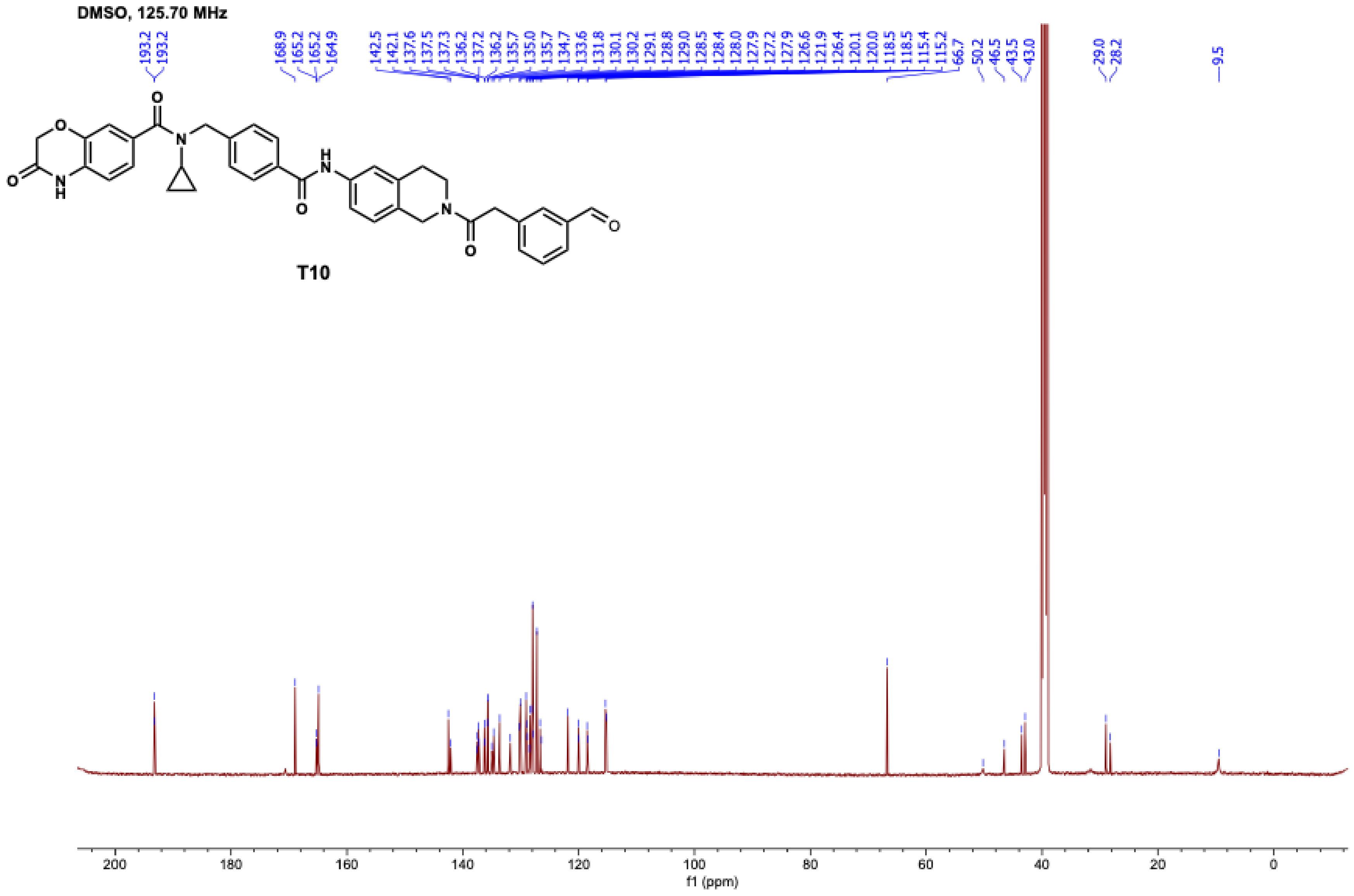

T10

^{13}C NMR spectrum of T10

DMSO, 400.13 MHz

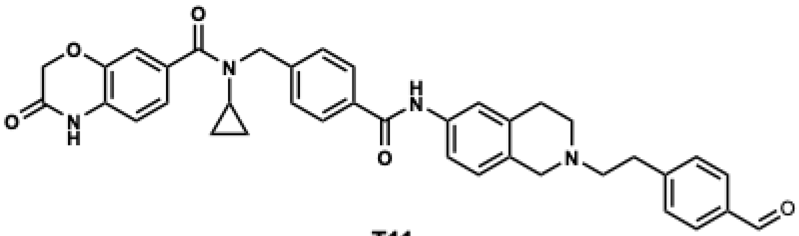

T11

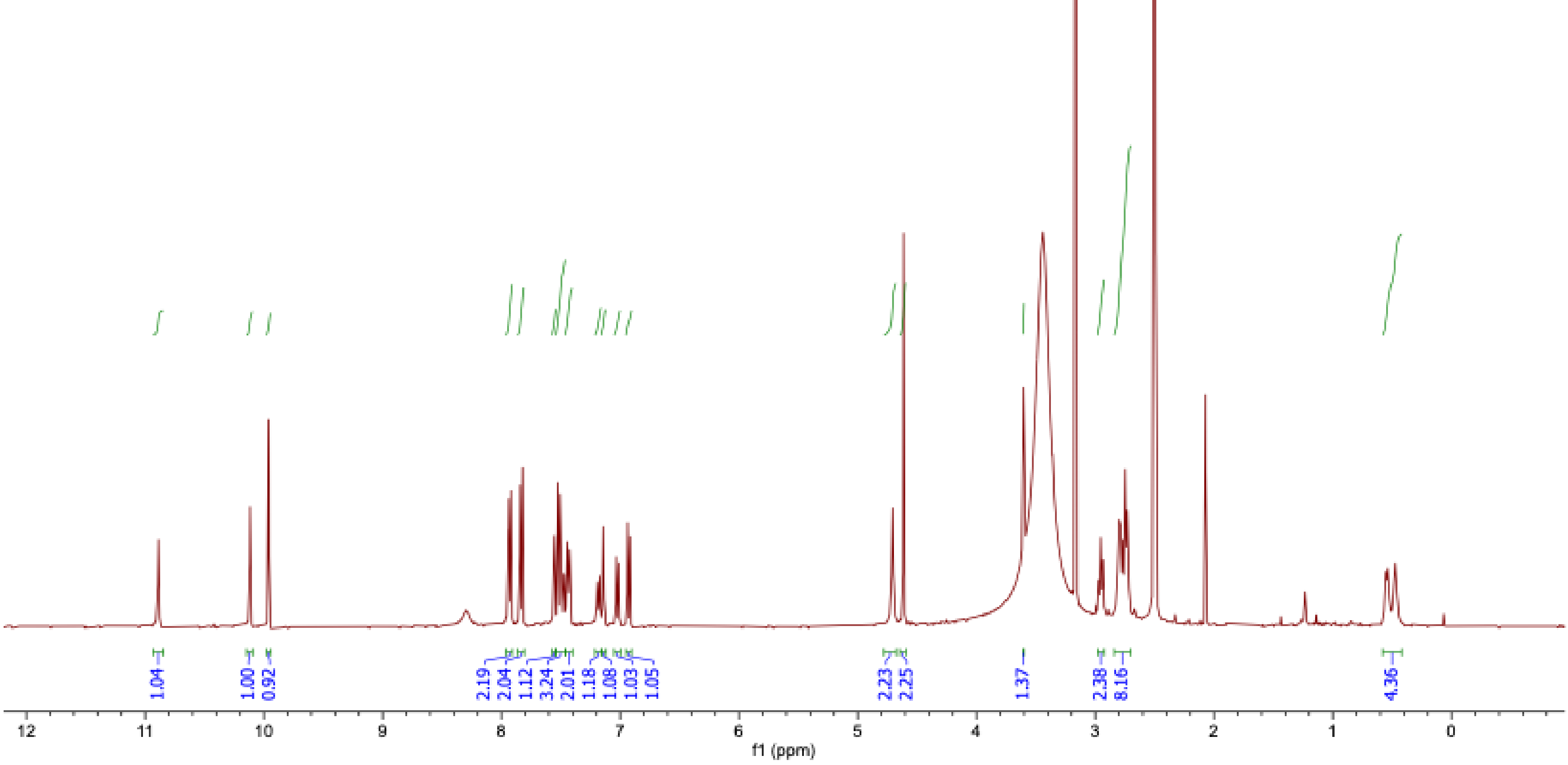

^{1}H NMR spectrum of T11

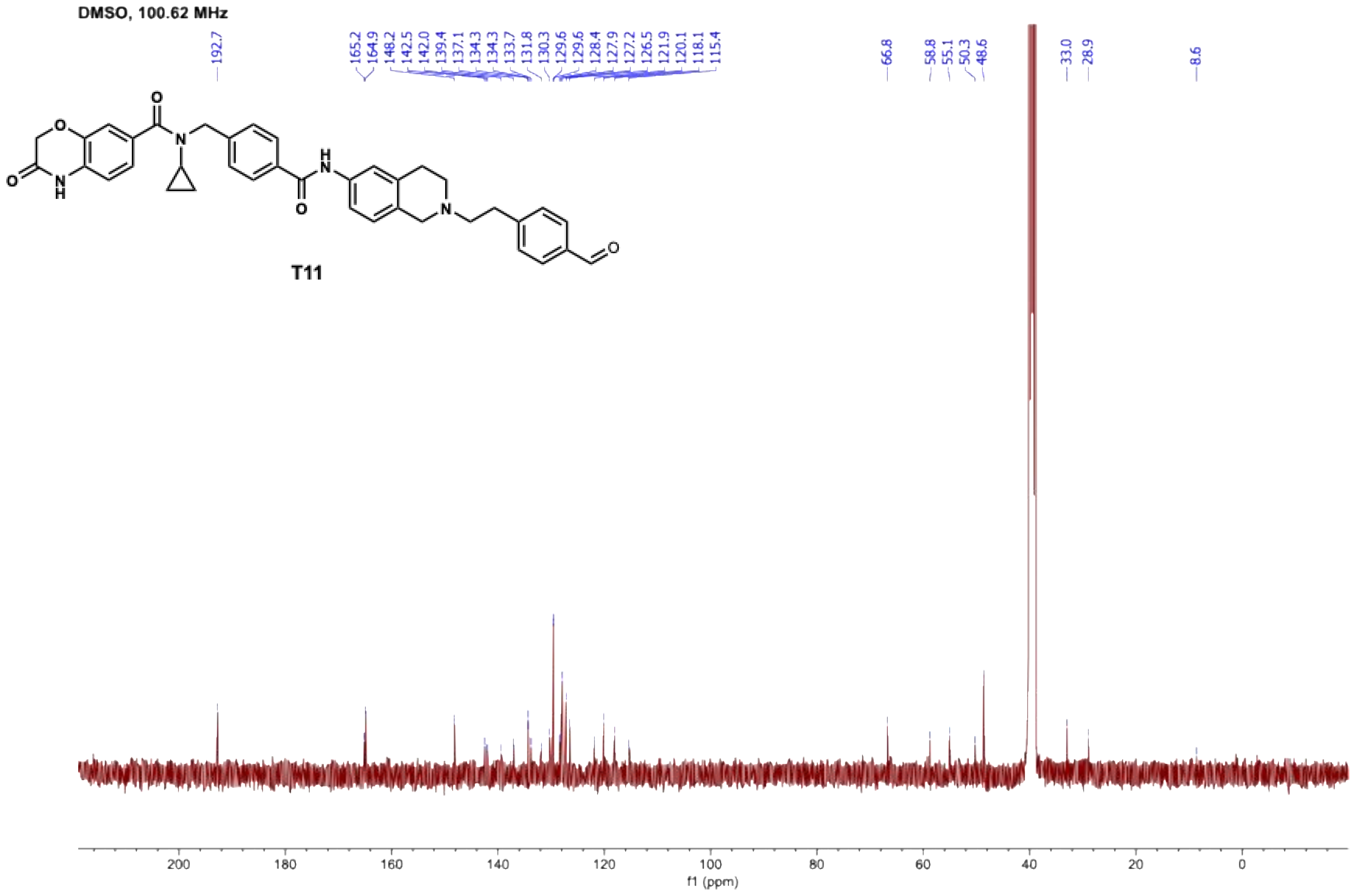

^{13}C NMR spectrum of T11

DMSO, 400.13 MHz

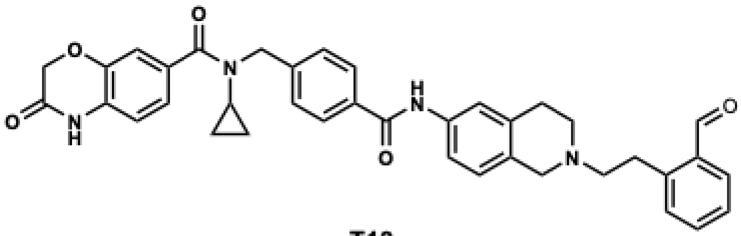

T12

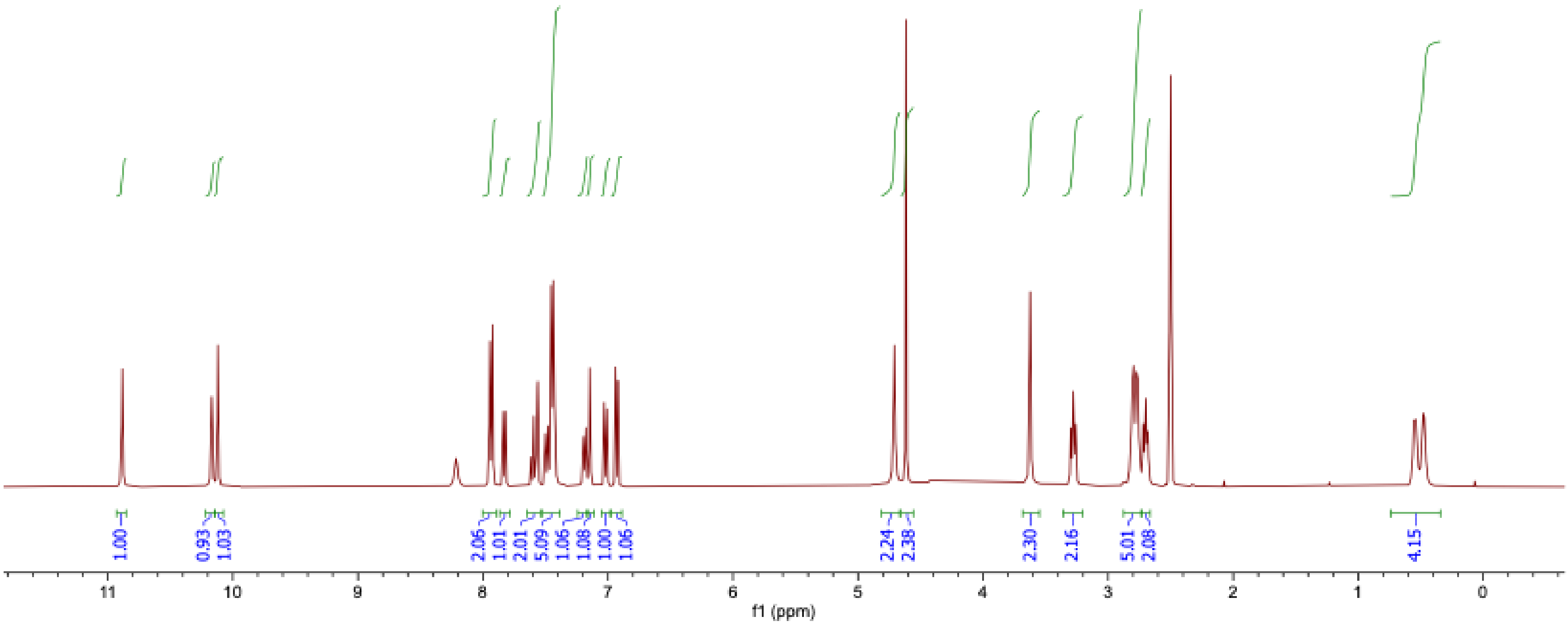

^{1}H NMR spectrum of T12

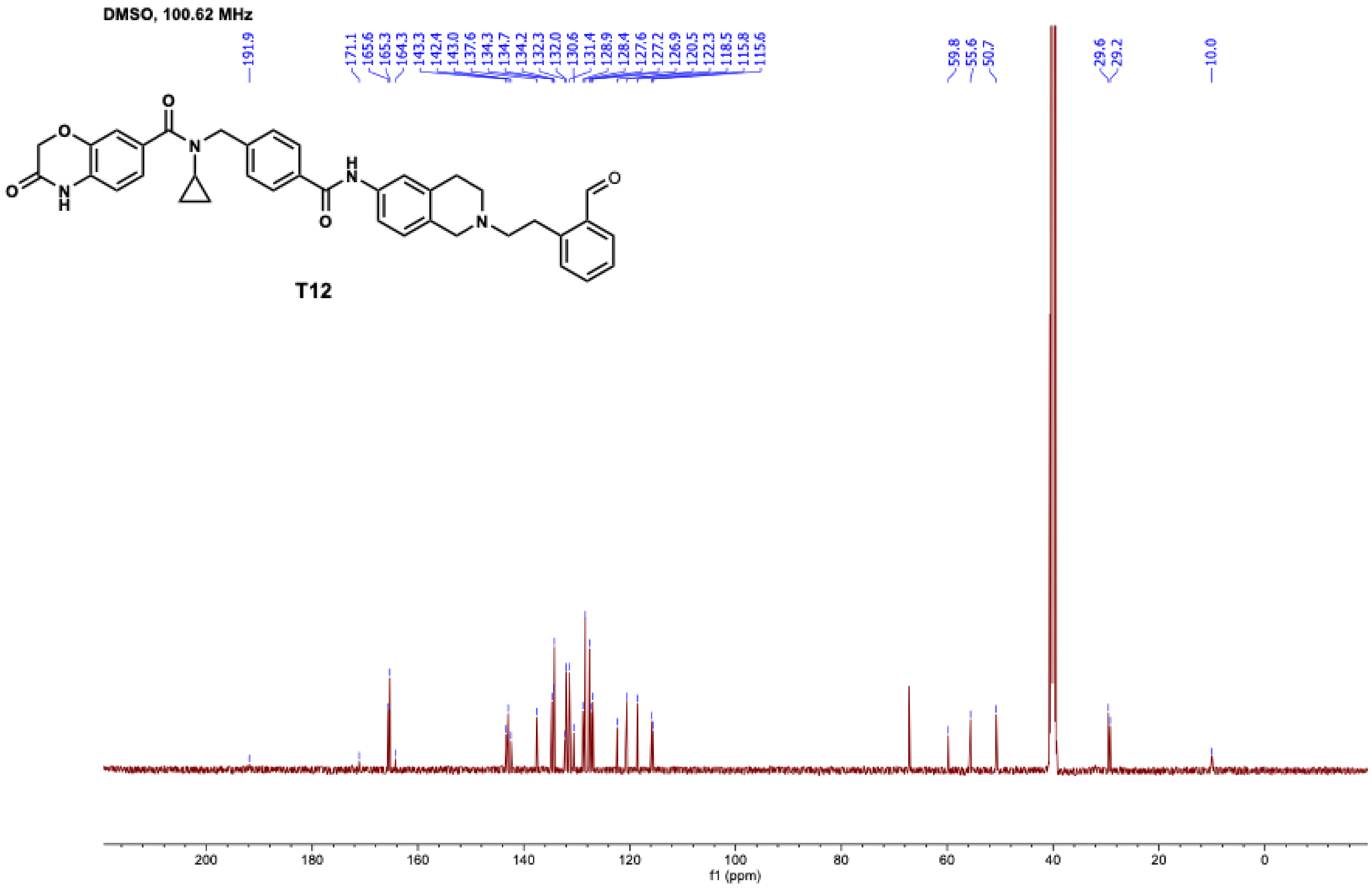

^{13}C NMR spectrum of T12

DMSO, 400.13 MHz

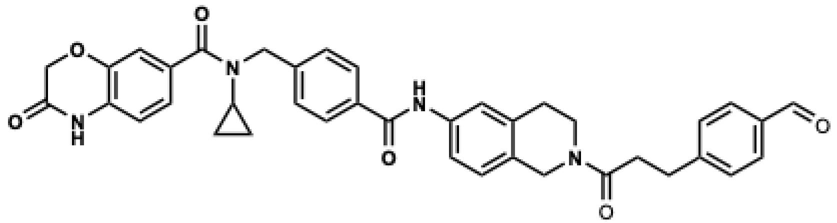

T13

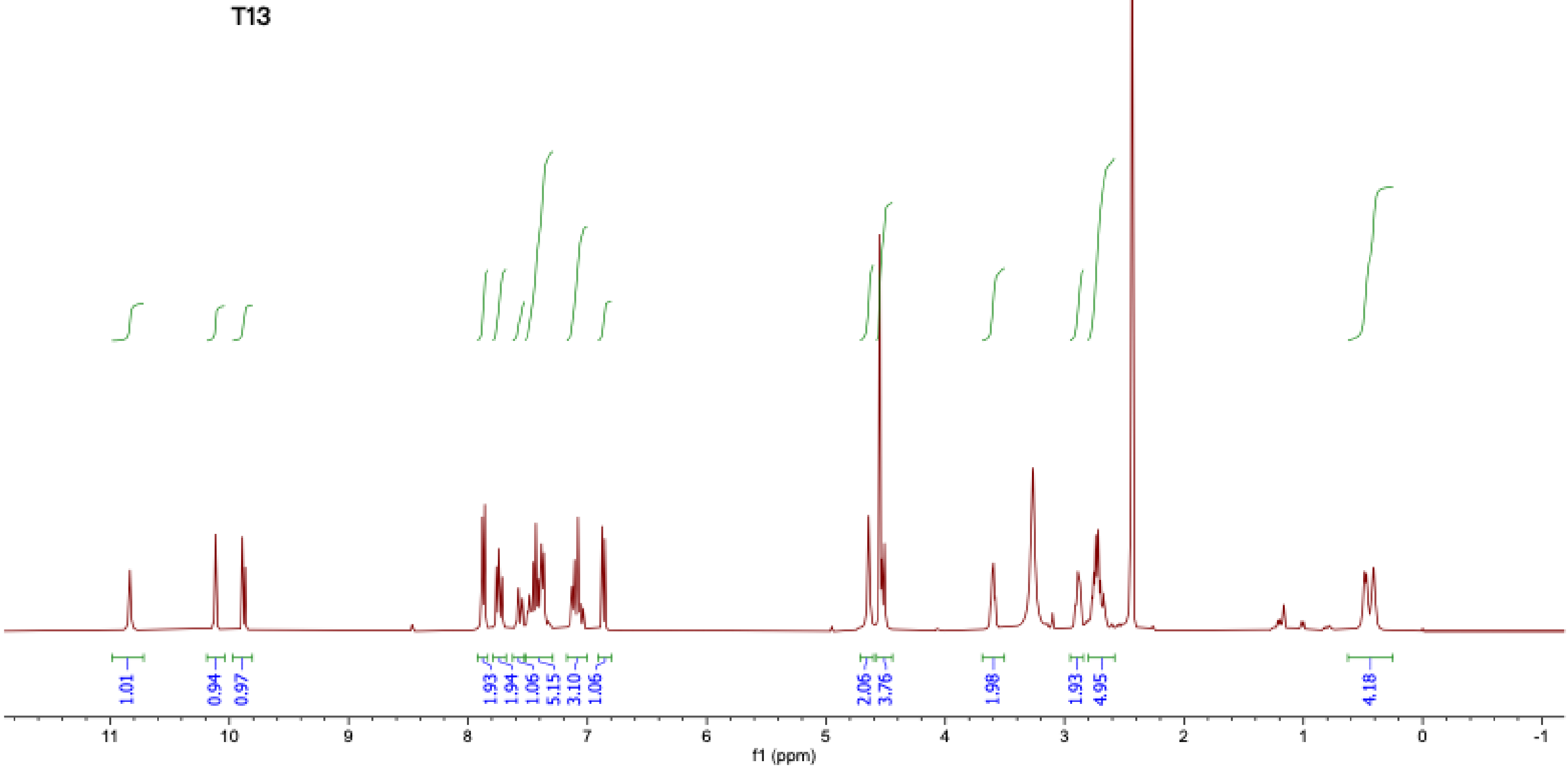

^{1}H NMR spectrum of T13

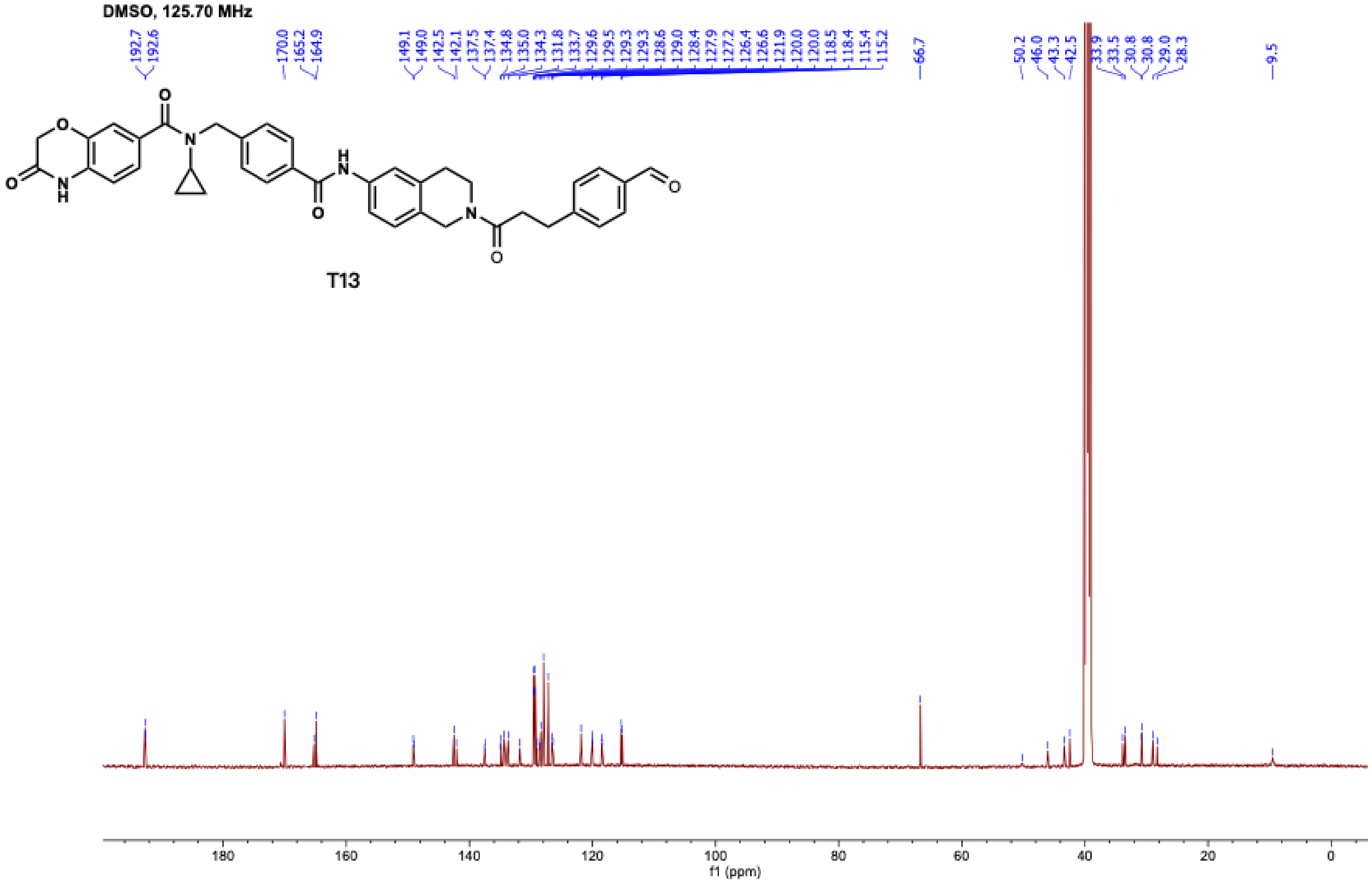

^{13}C NMR spectrum of T13

DMSO, 400.13 MHz

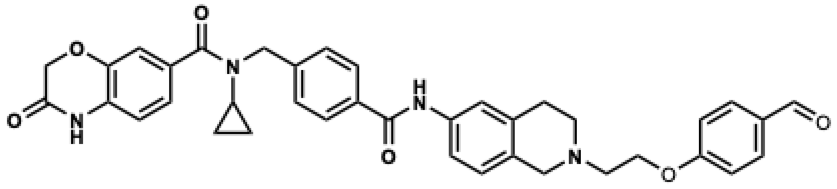

T14

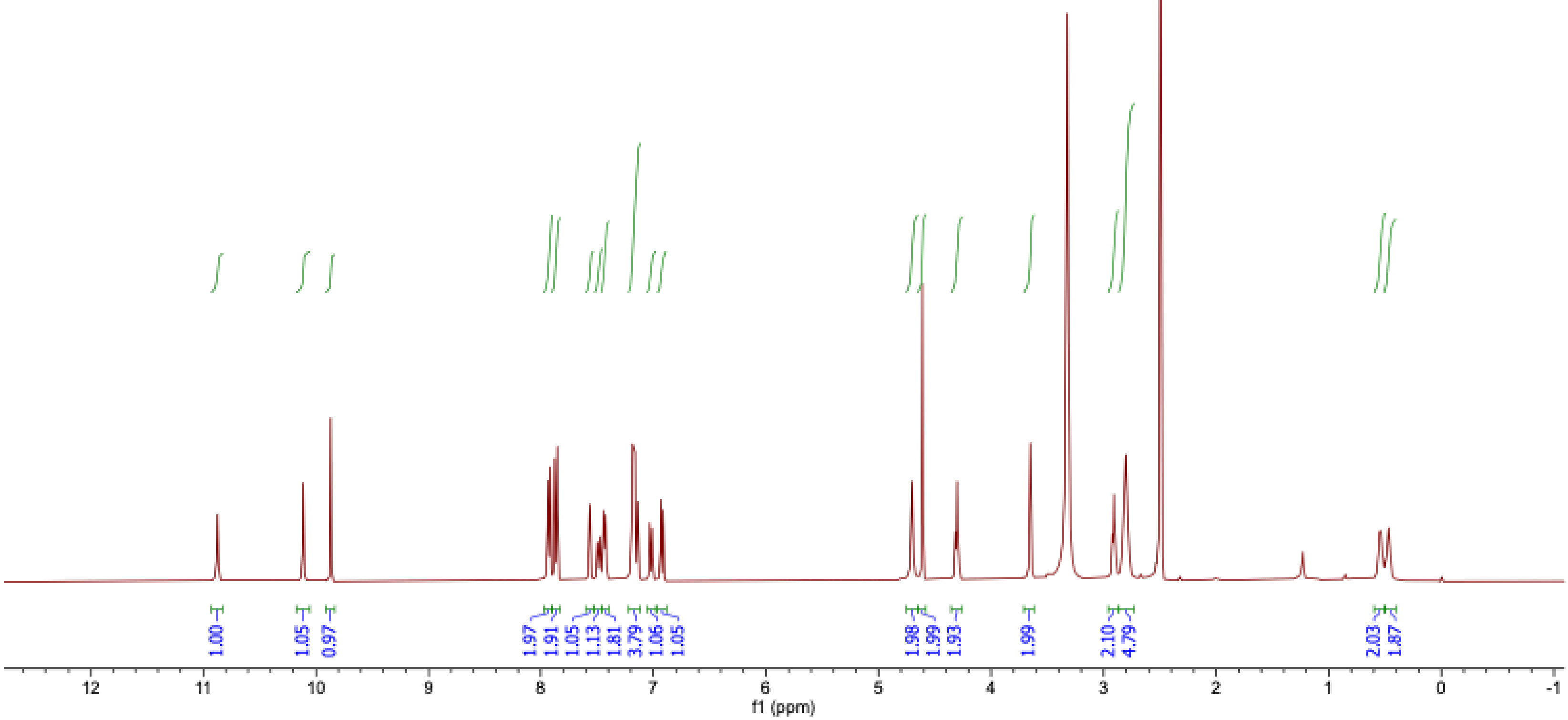

^{1}H NMR spectrum of T14

DMSO, 100.62 MHz

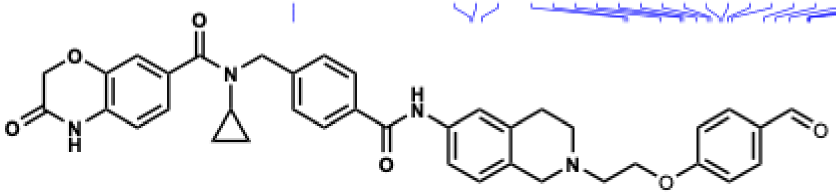

T14

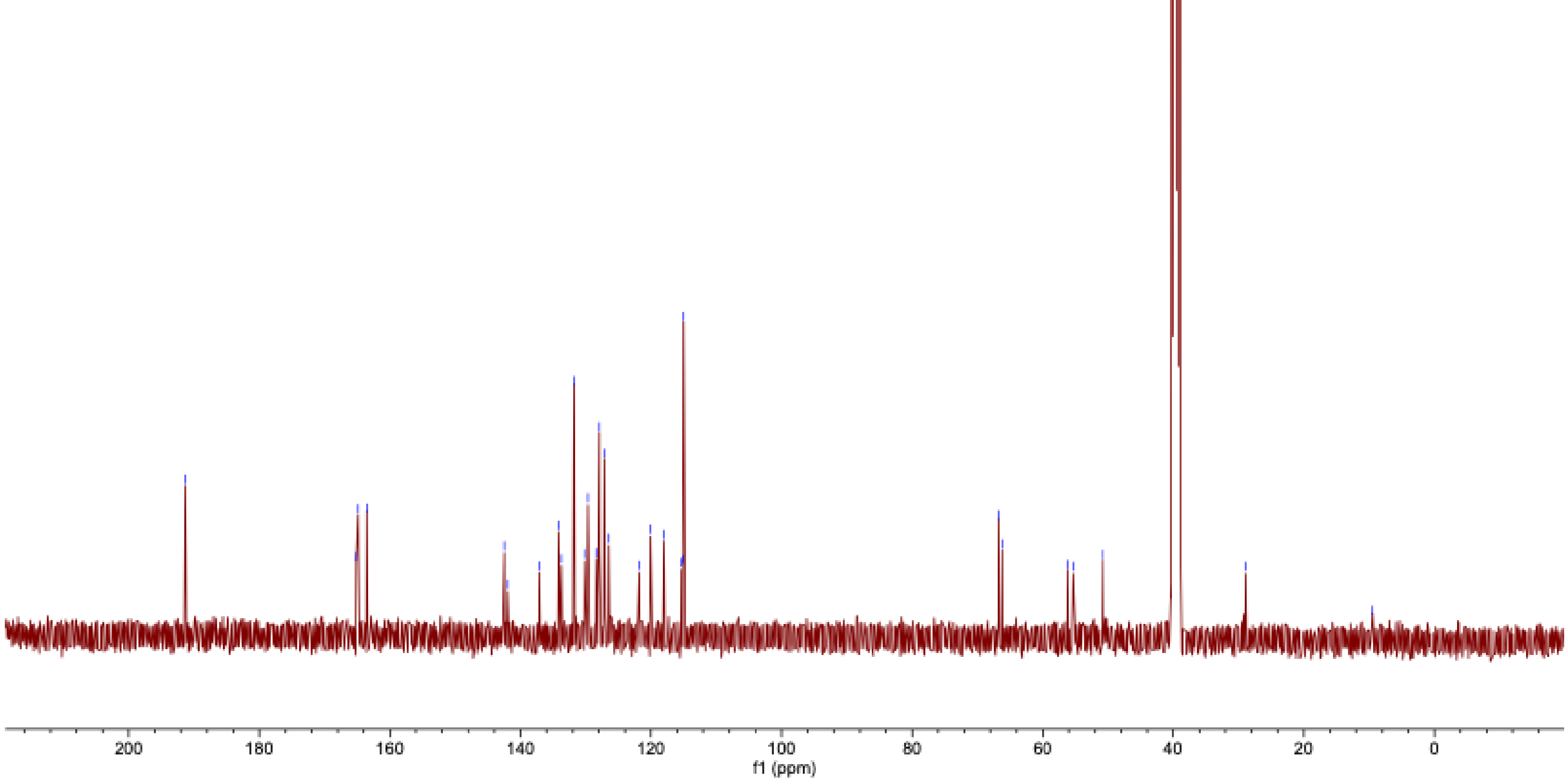

^{13}C NMR spectrum of T14

DMSO, 400.13 MHz

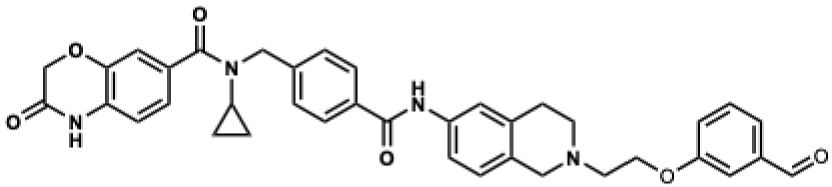

T15

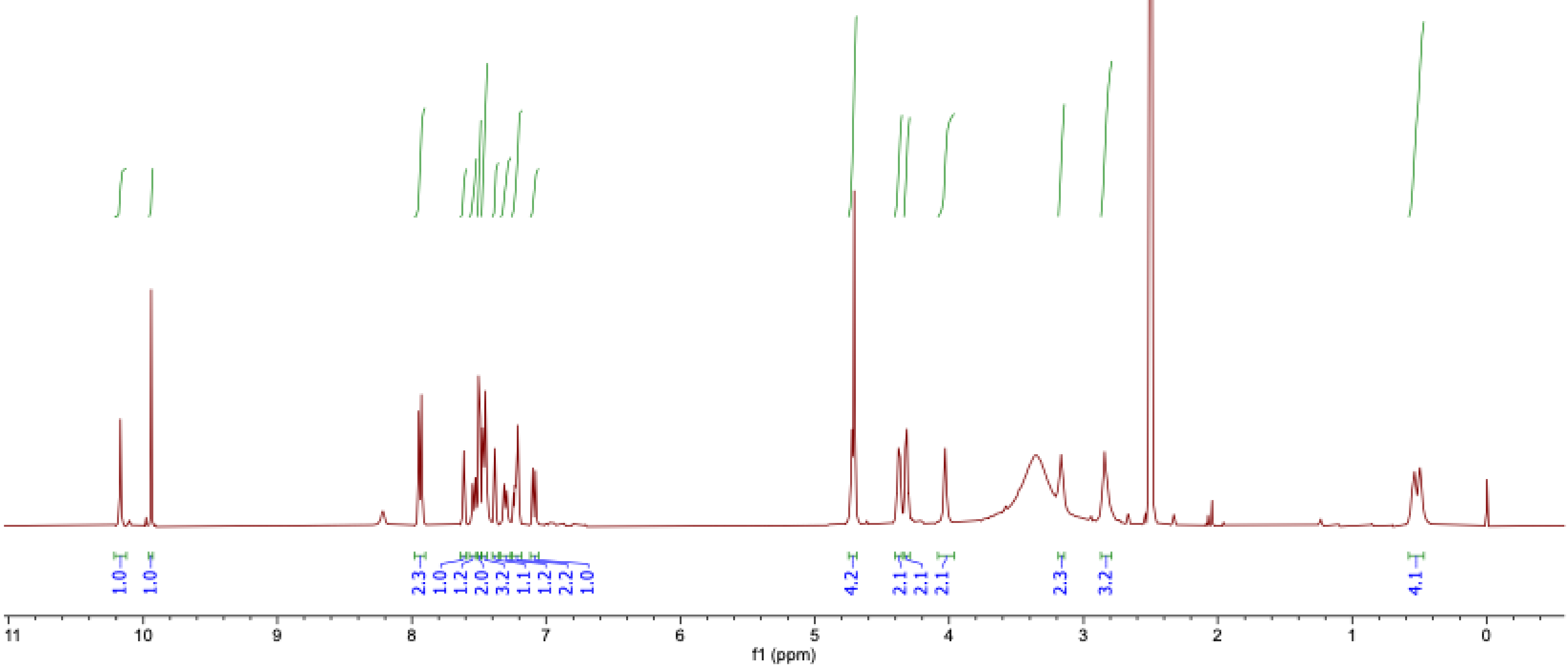

^{1}H NMR spectrum of T15

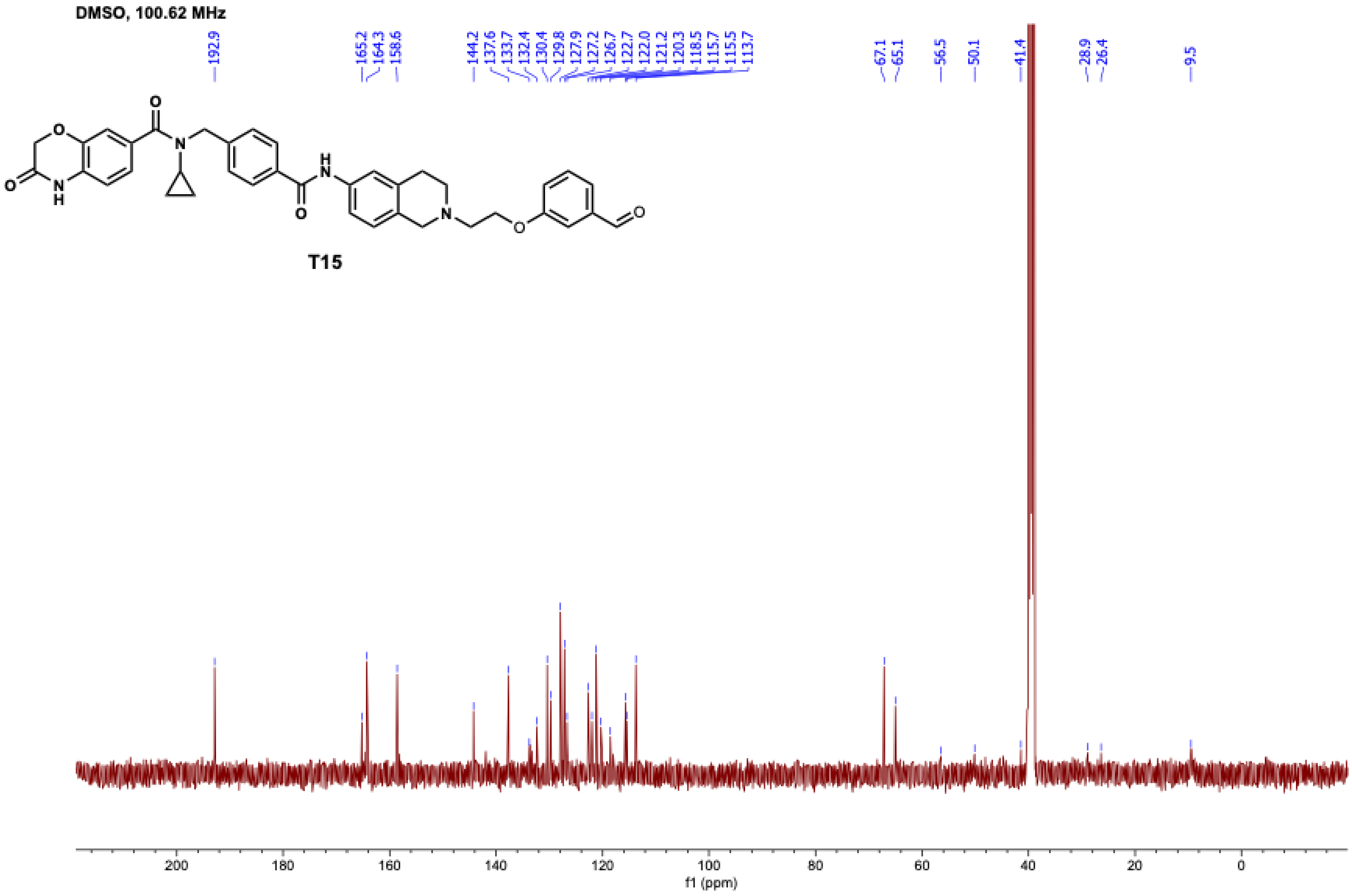

^{13}C NMR spectrum of T15

DMSO, 400.13 MHz

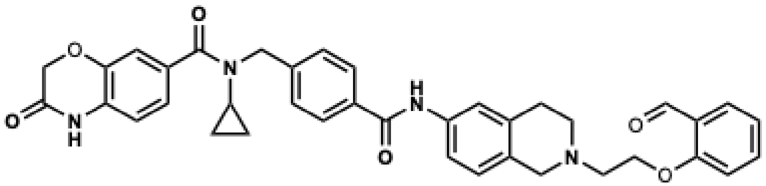

T16

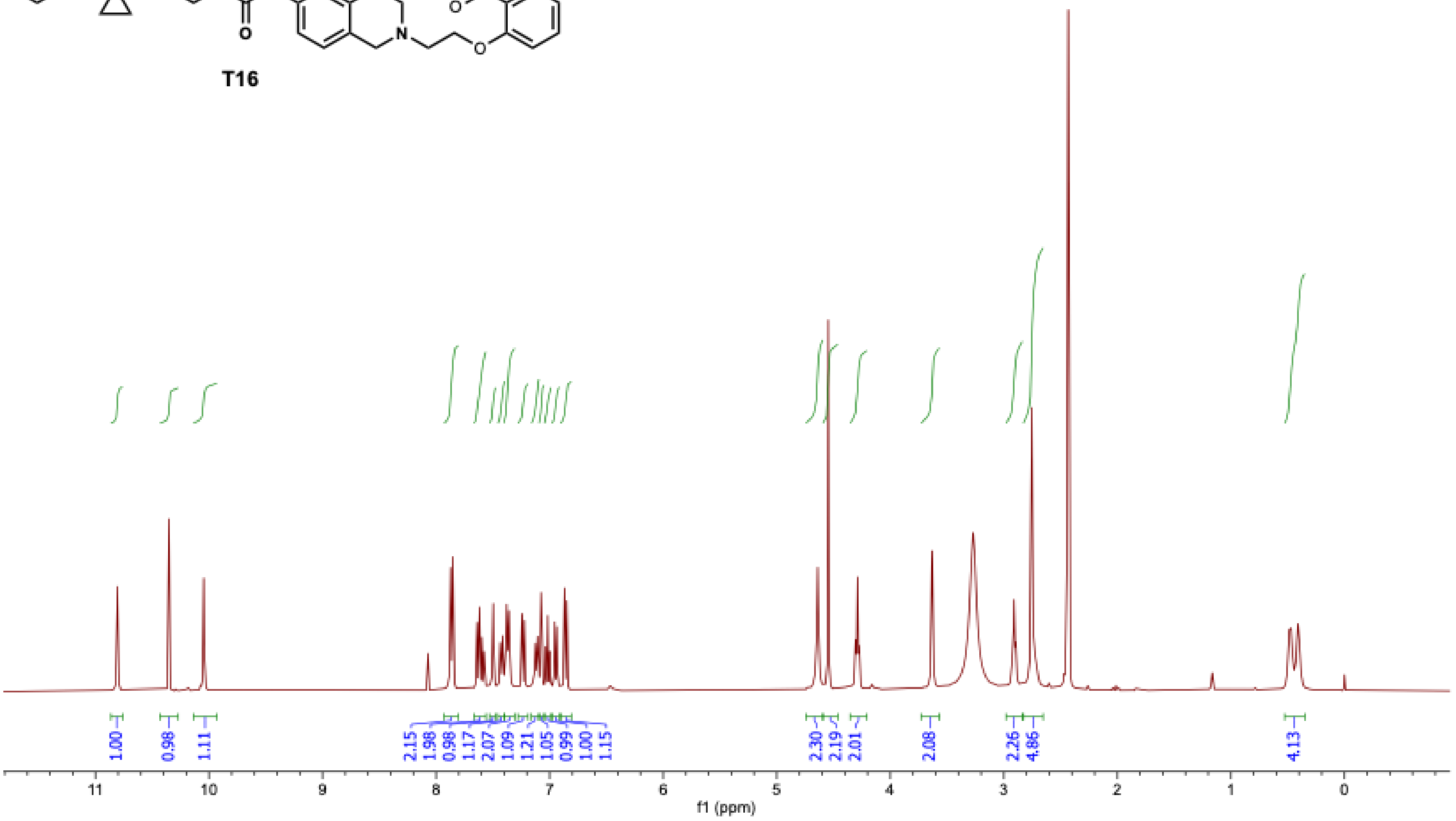

^{1}H NMR spectrum of T16

DMSO, 100.62 MHz

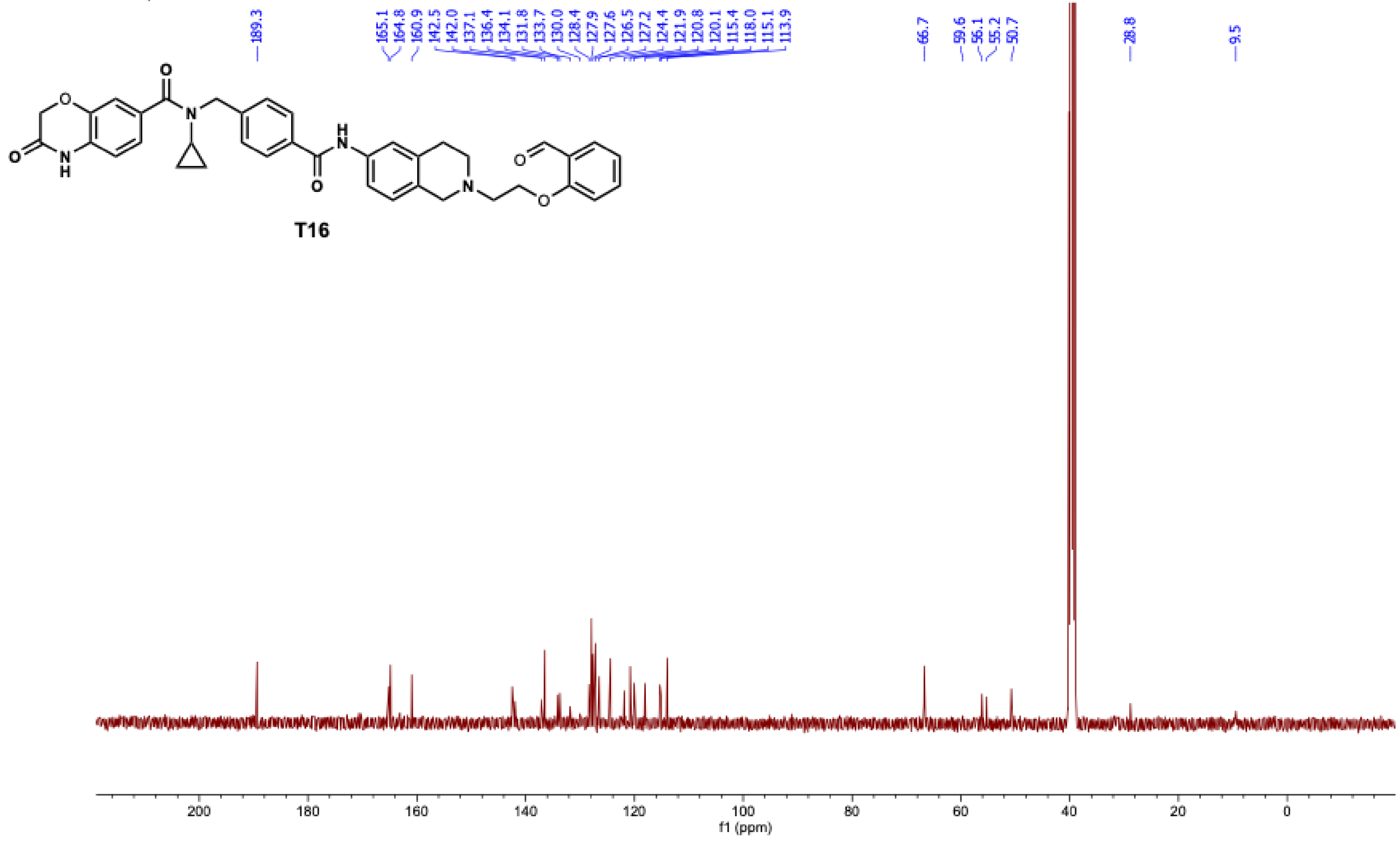

T16

^{13}C NMR spectrum of T16

DMSO, 400.13 MHz

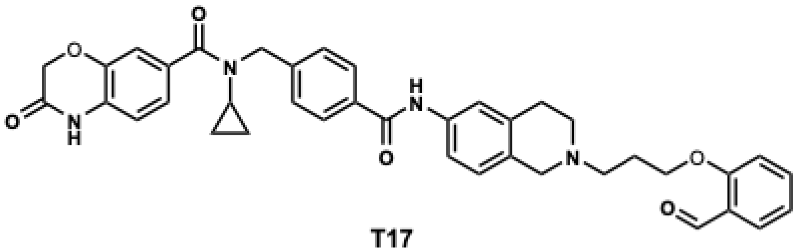

T17

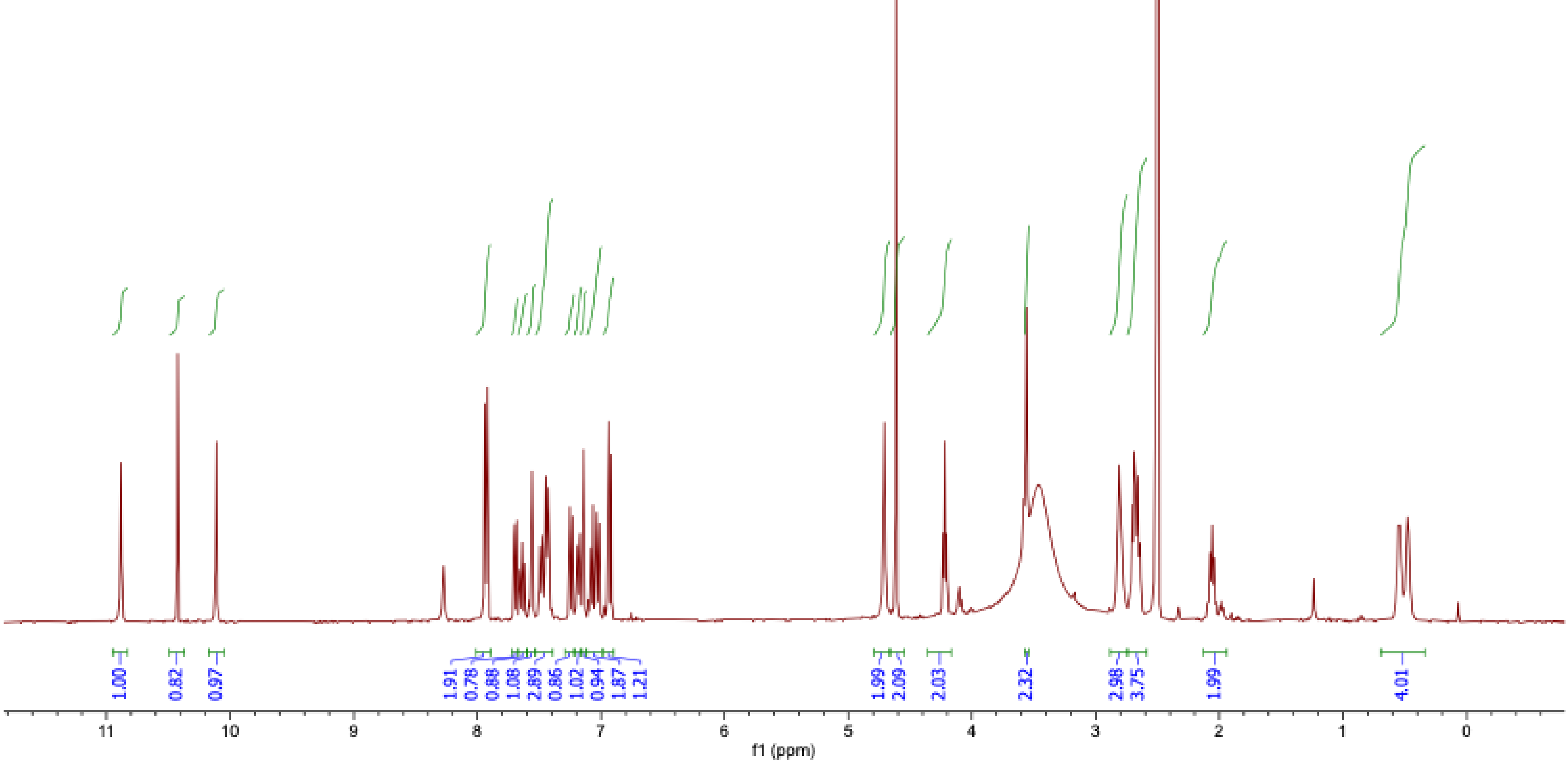

^{1}H NMR spectrum of T17

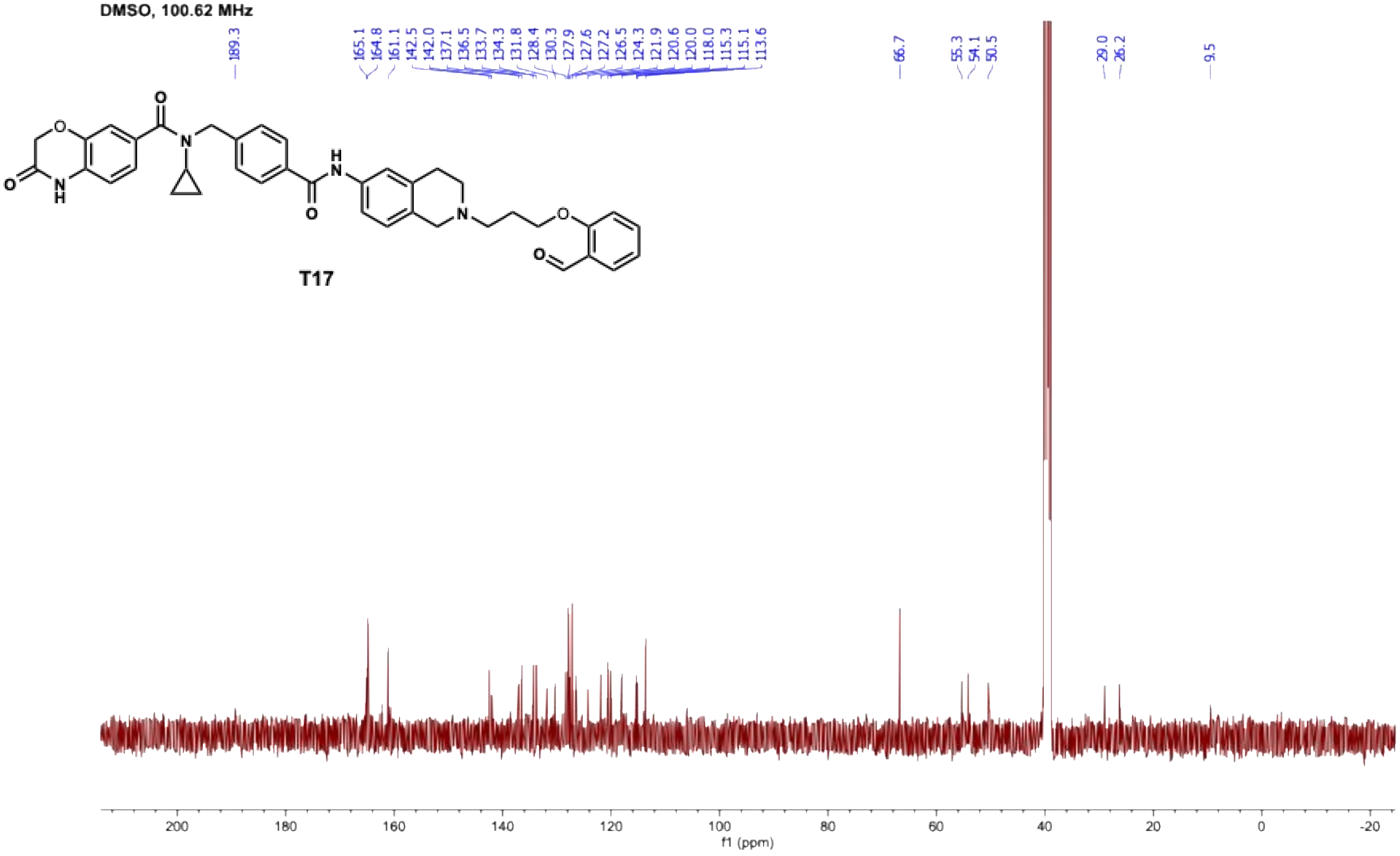

^{13}C NMR spectrum of T17

DMSO, 400.13 MHz

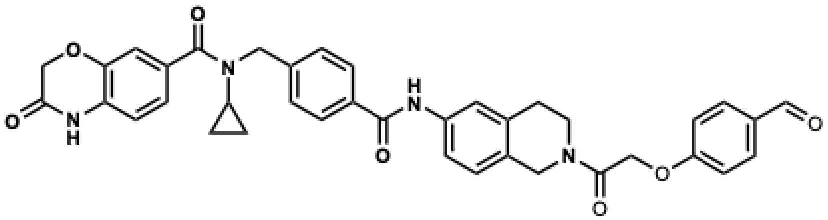

T18

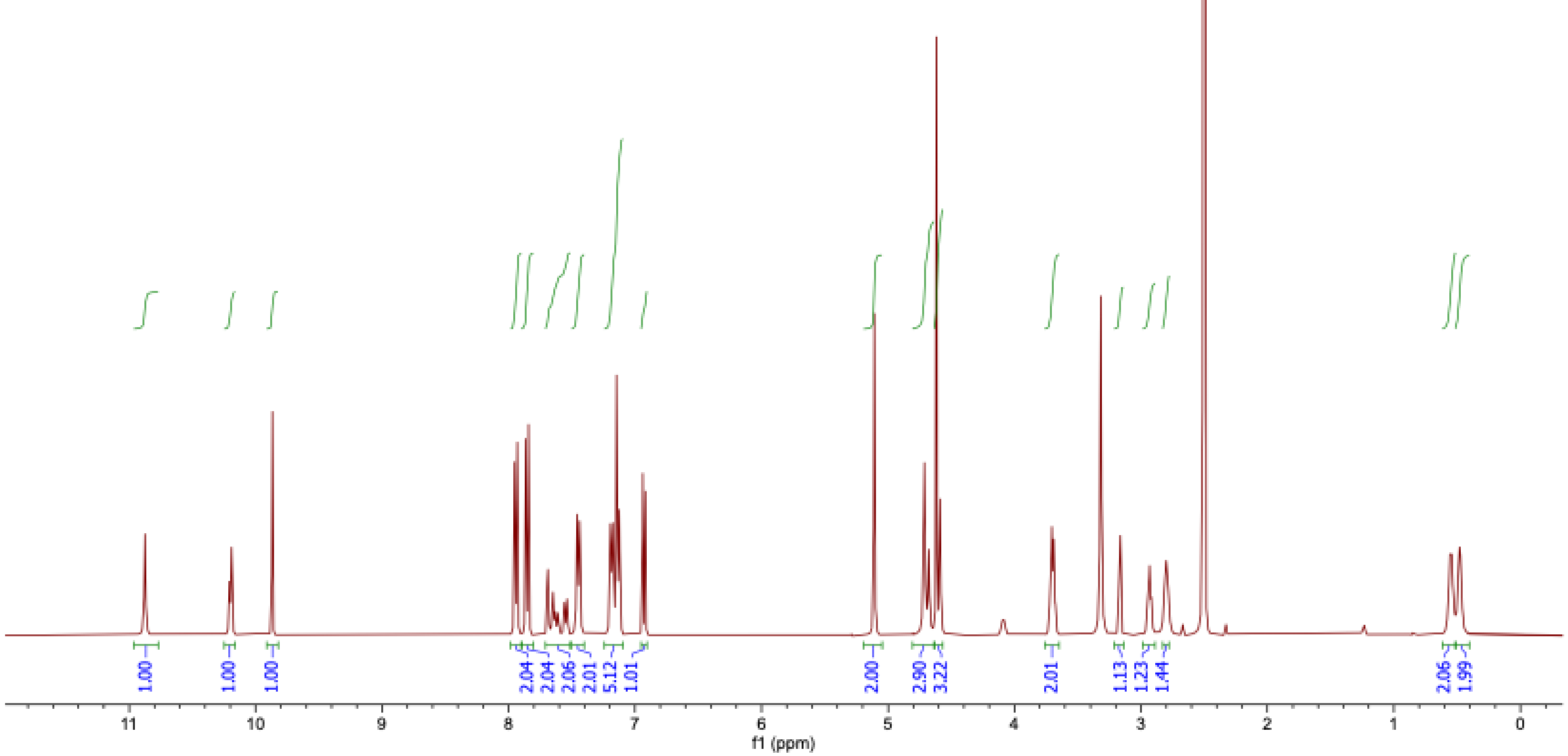

^{1}H NMR spectrum of T18

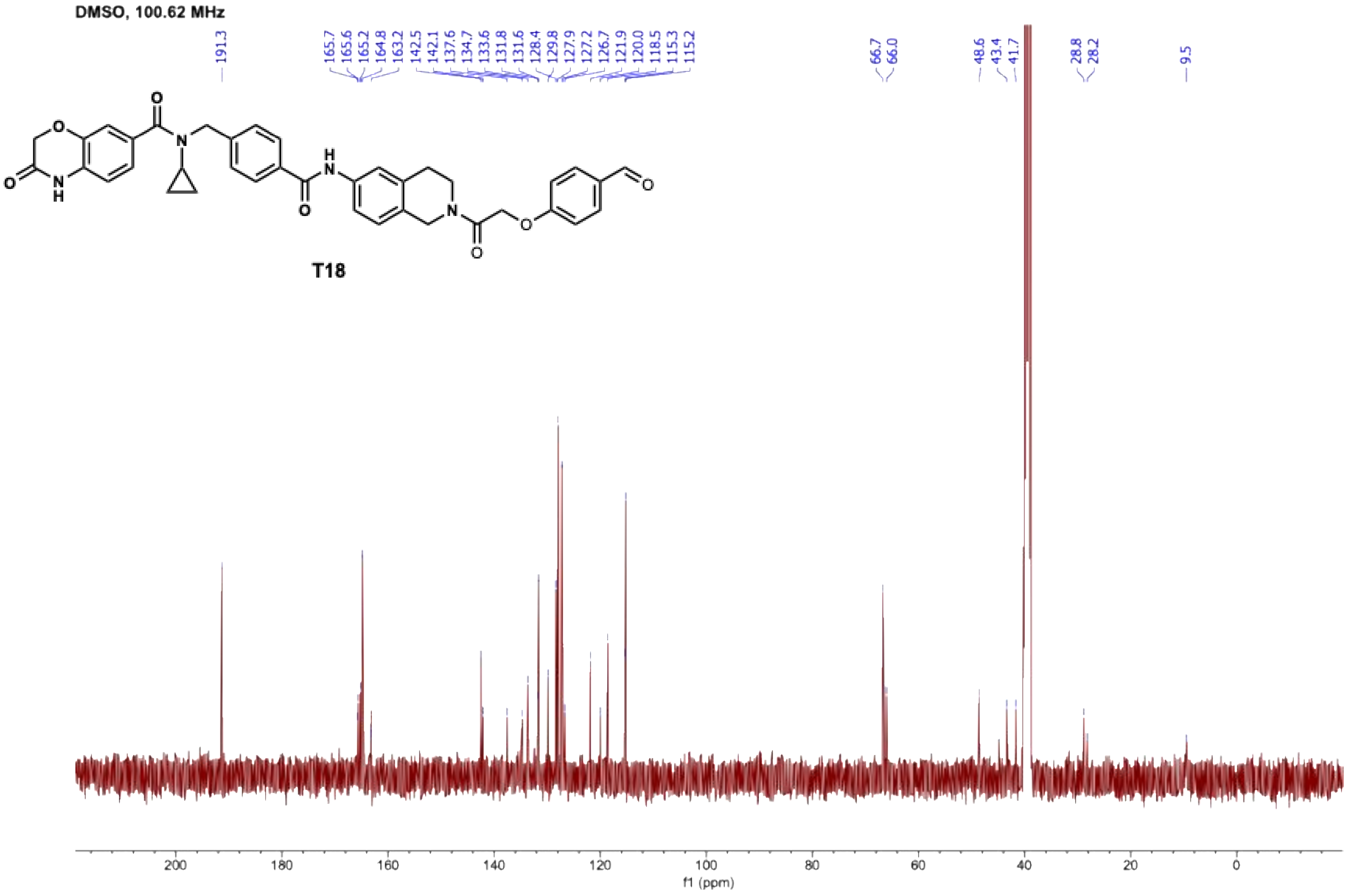

^{13}C NMR spectrum of T18

DMSO, 499.84 MHz

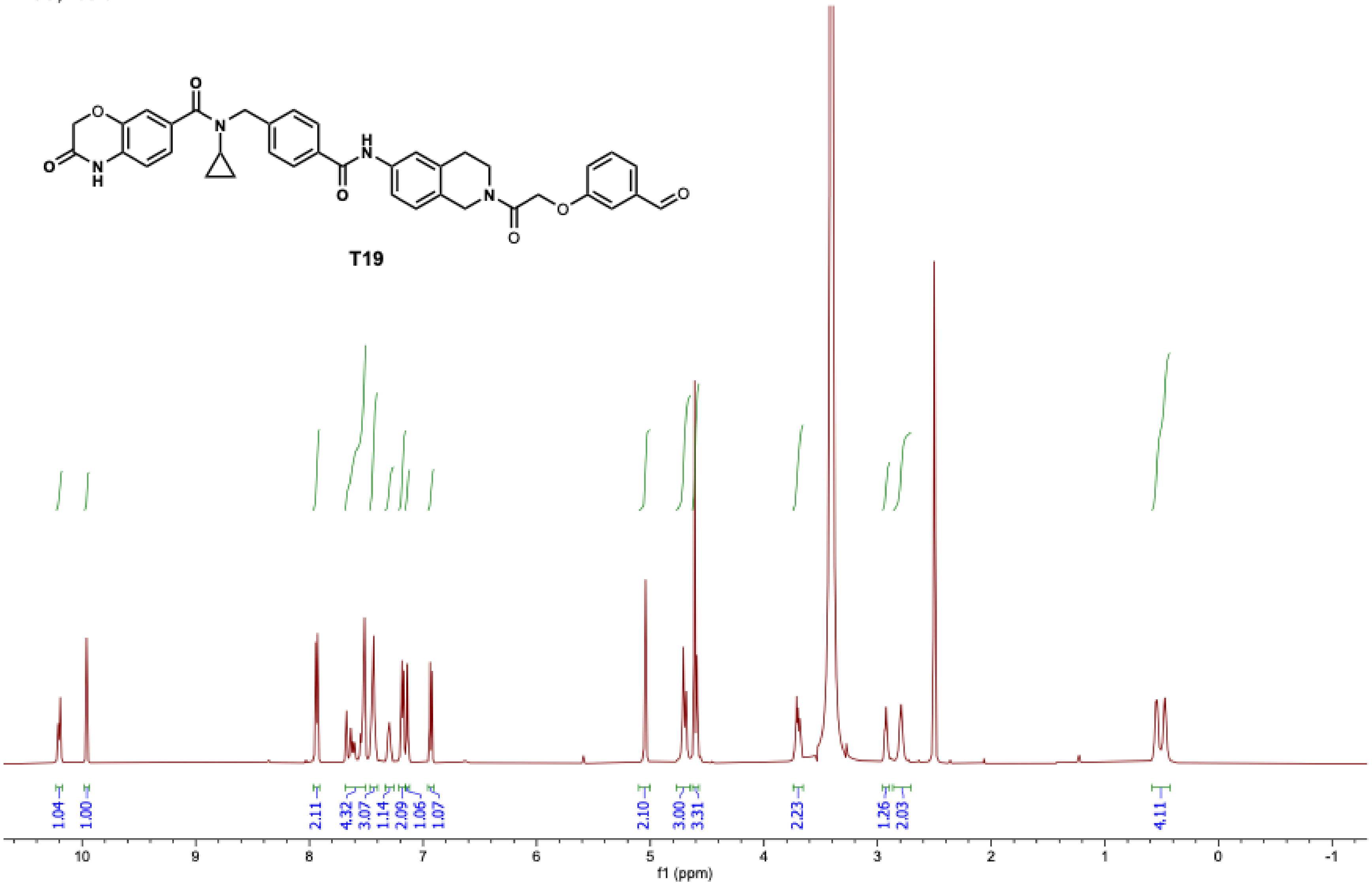

^{1}H NMR spectrum of T19

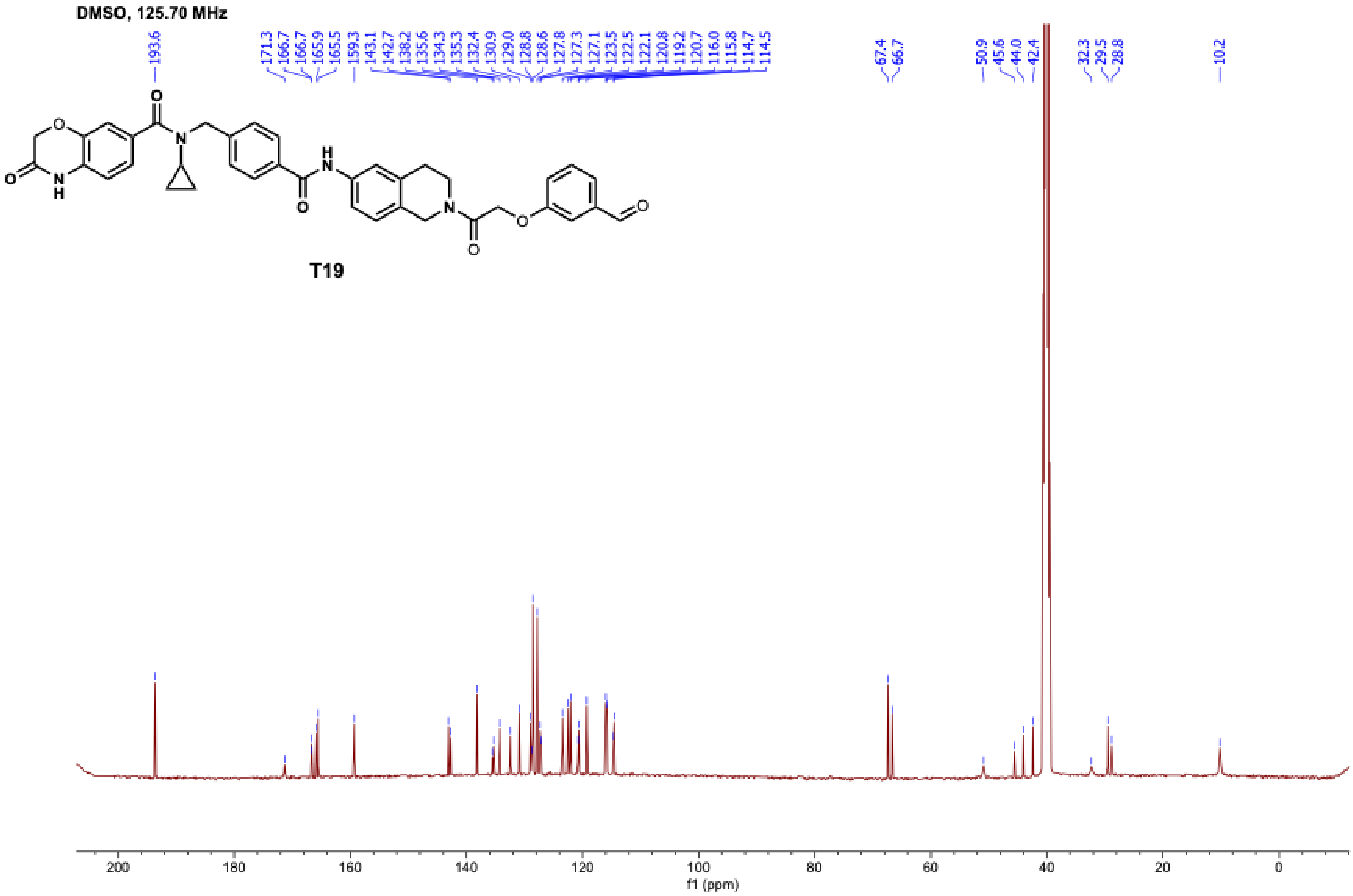

^{13}C NMR spectrum of T19

DMSO, 400.13 MHz

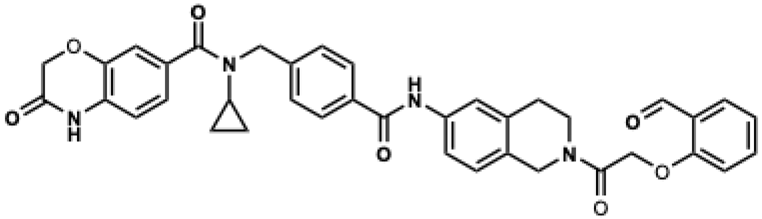

T20

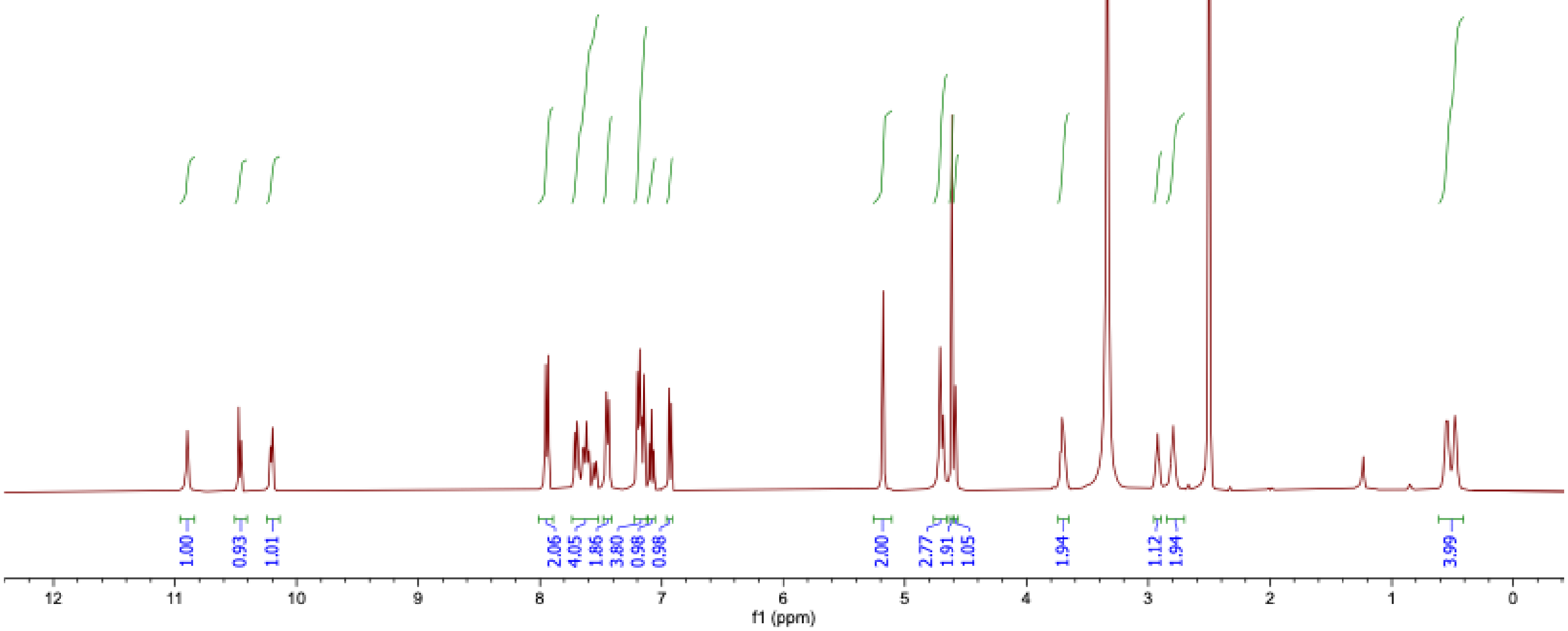

^{1}H NMR spectrum of T20

DMSO, 100.62 MHz

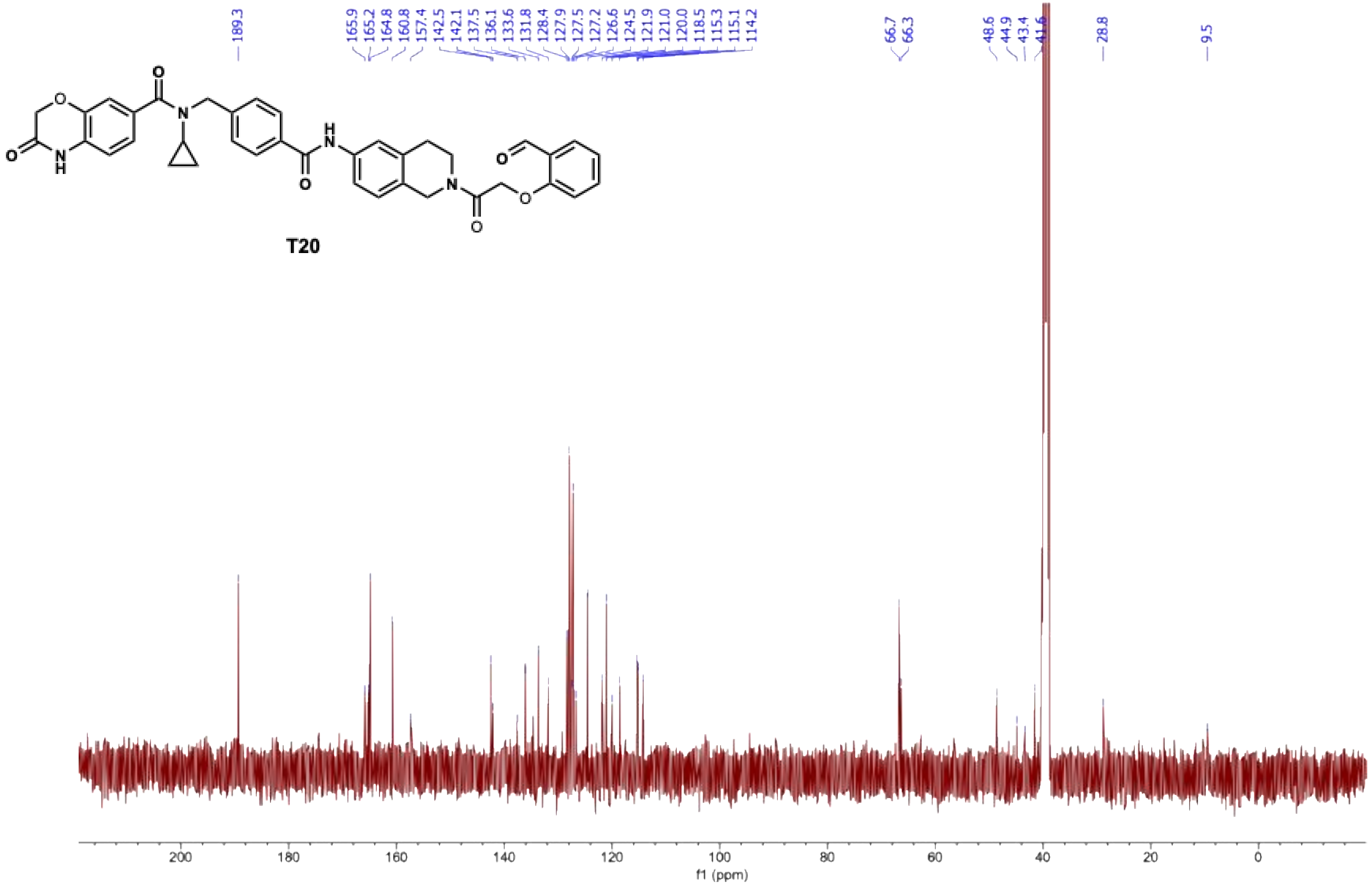

^{13}C NMR spectrum of T20

DMSO, 400.13 MHz

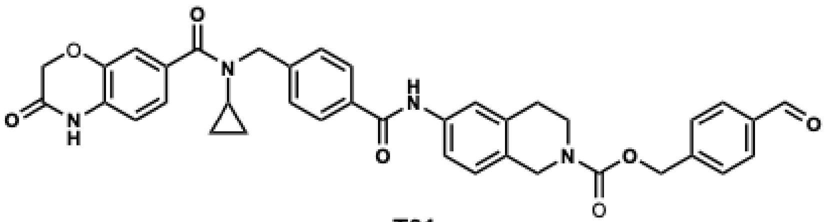

T21

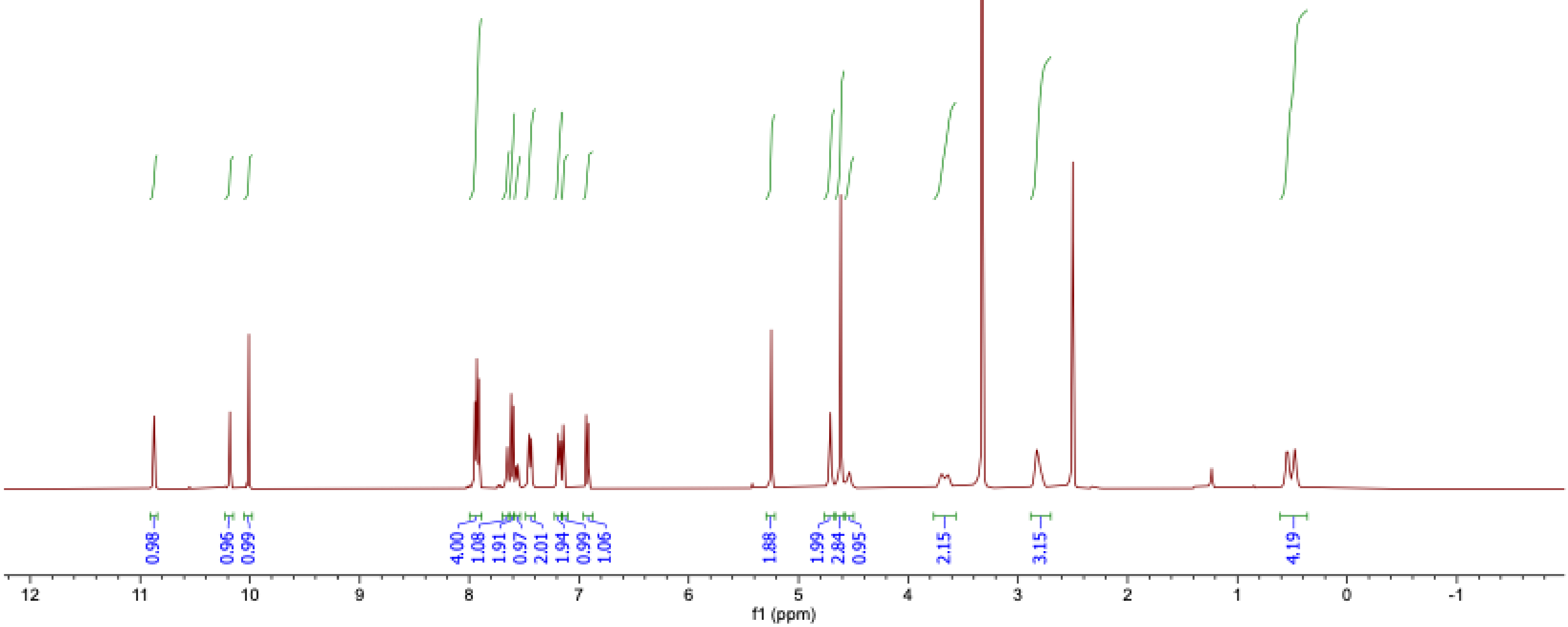

^{1}H NMR spectrum of T21

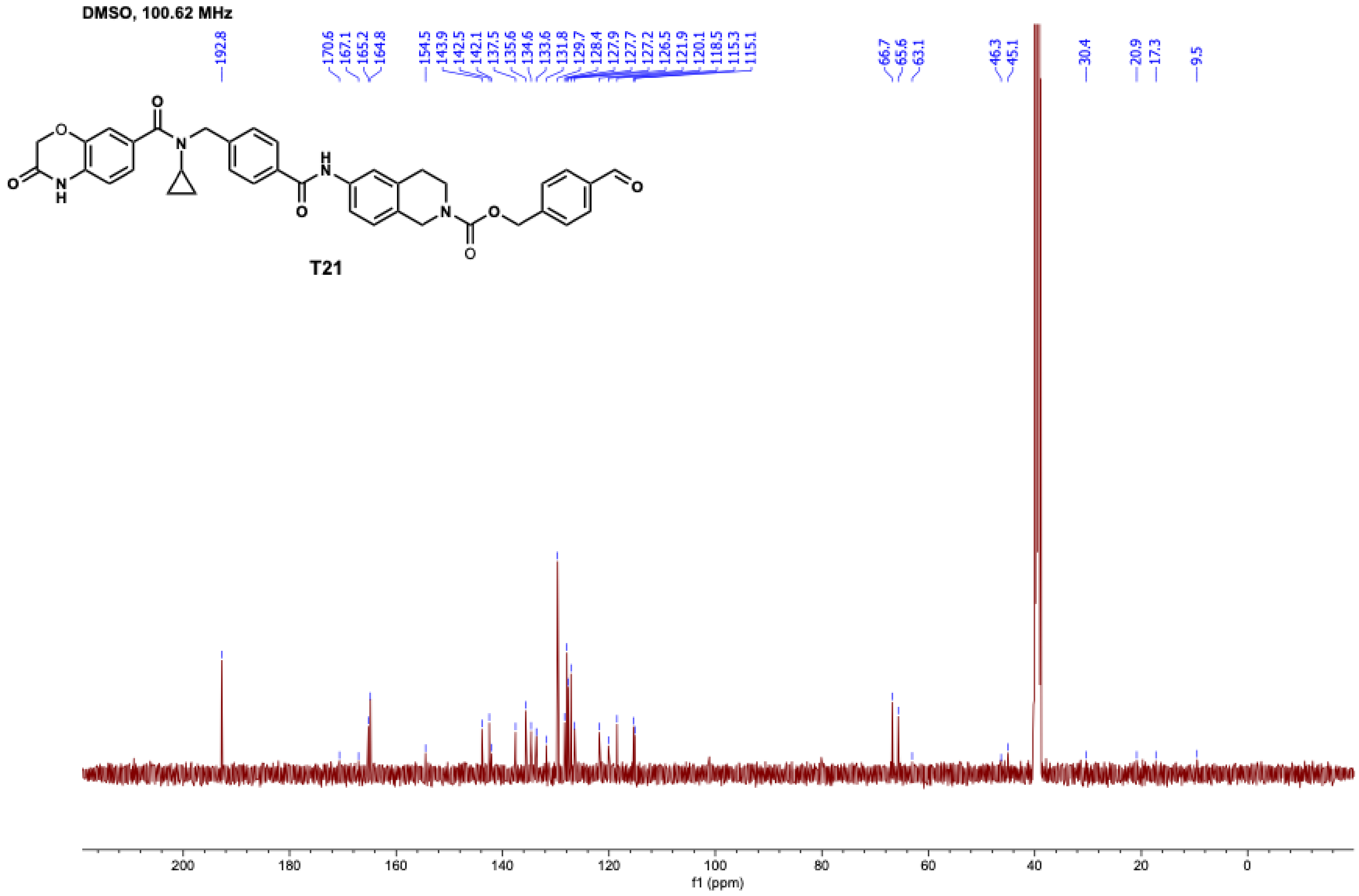

^{13}C NMR spectrum of T21

CDCl3, 400.13 MHz

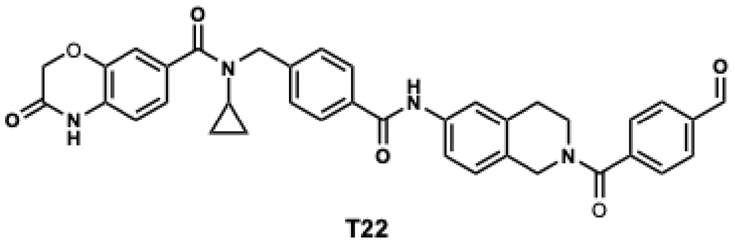

T22

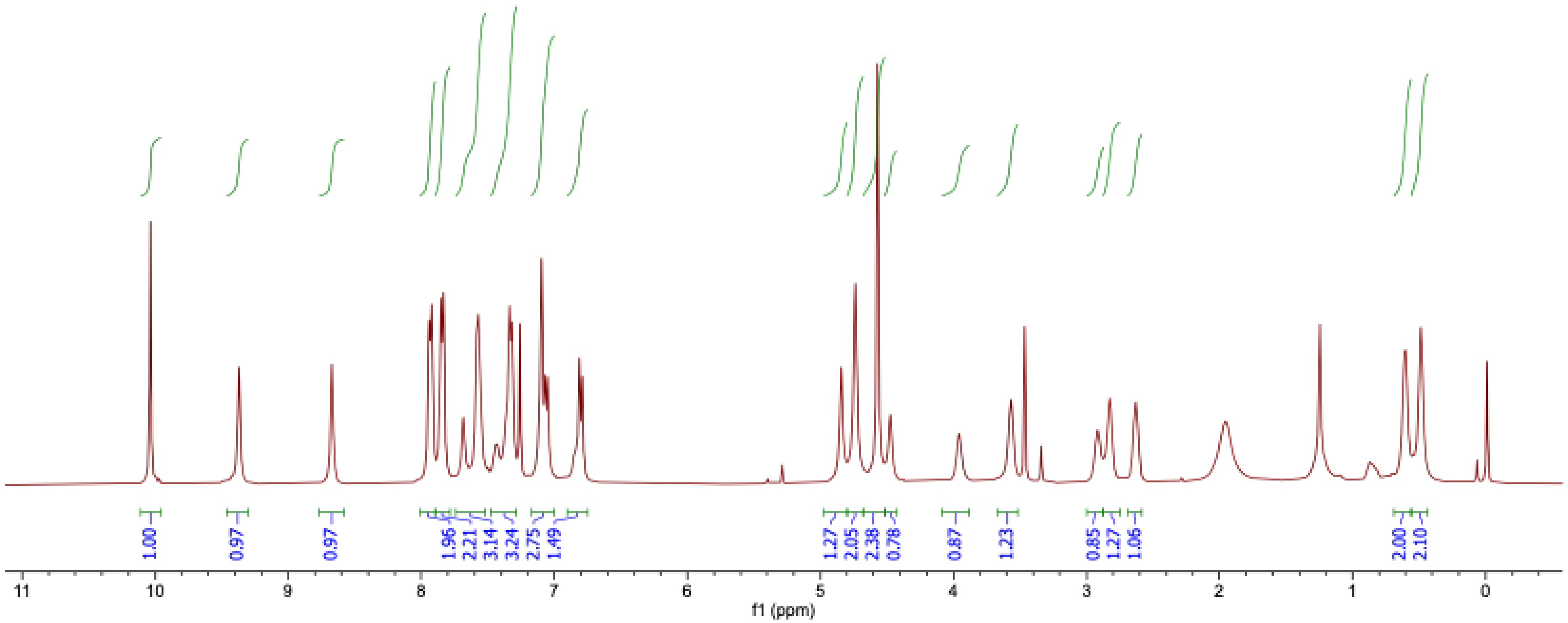

^{1}H NMR spectrum of T22

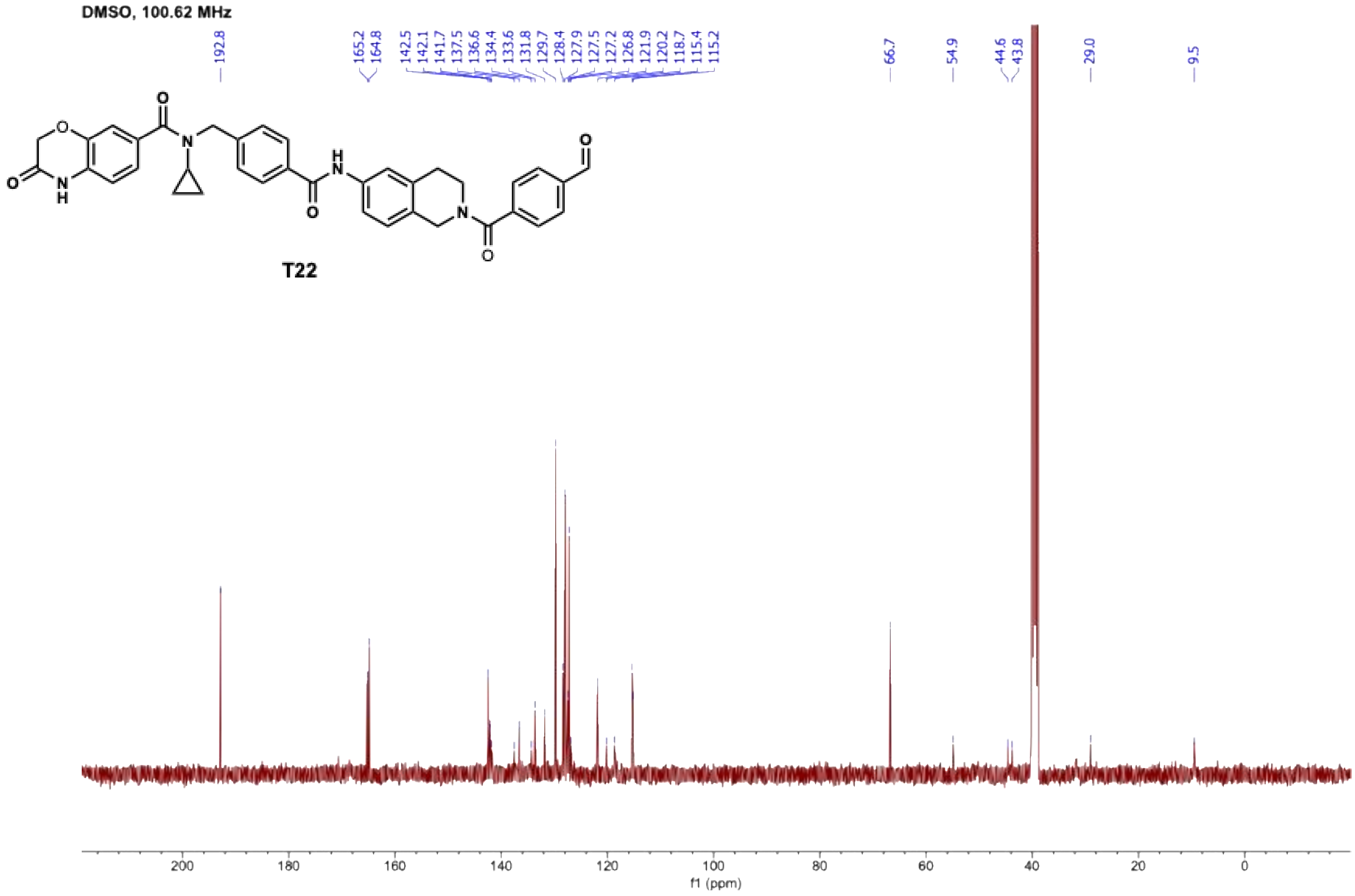

^{13}C NMR spectrum of T22

DMSO, 400.13 MHz

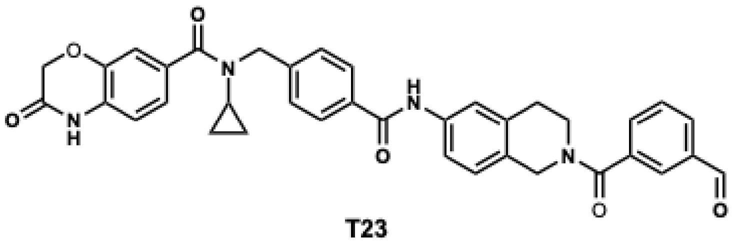

T23

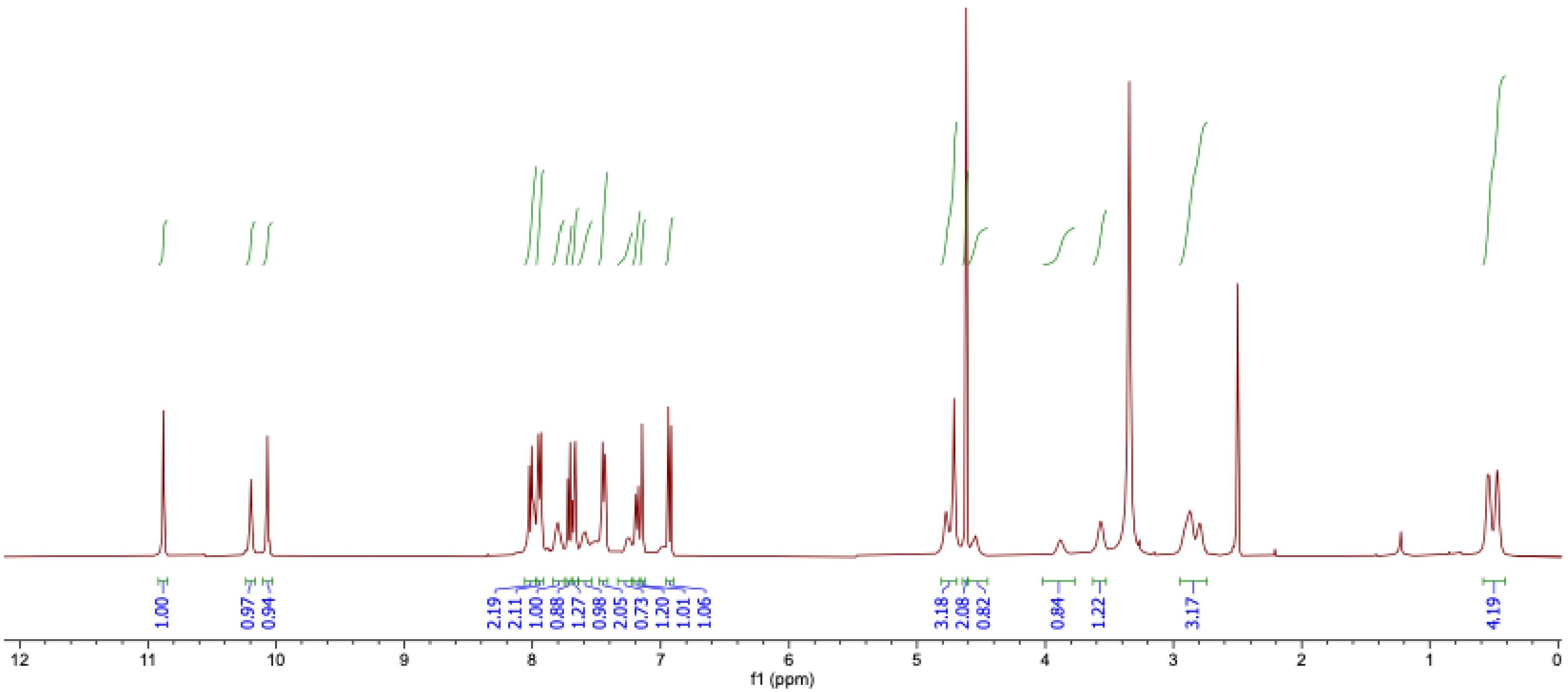

^{1}H NMR spectrum of T23

CDCl3, 100.62 MHz

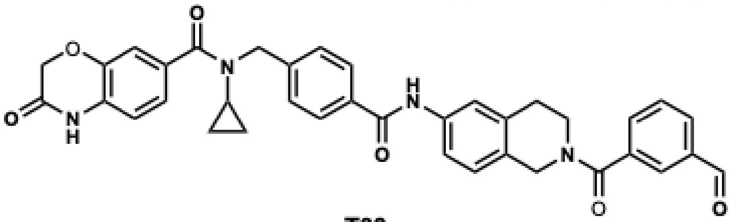

T23

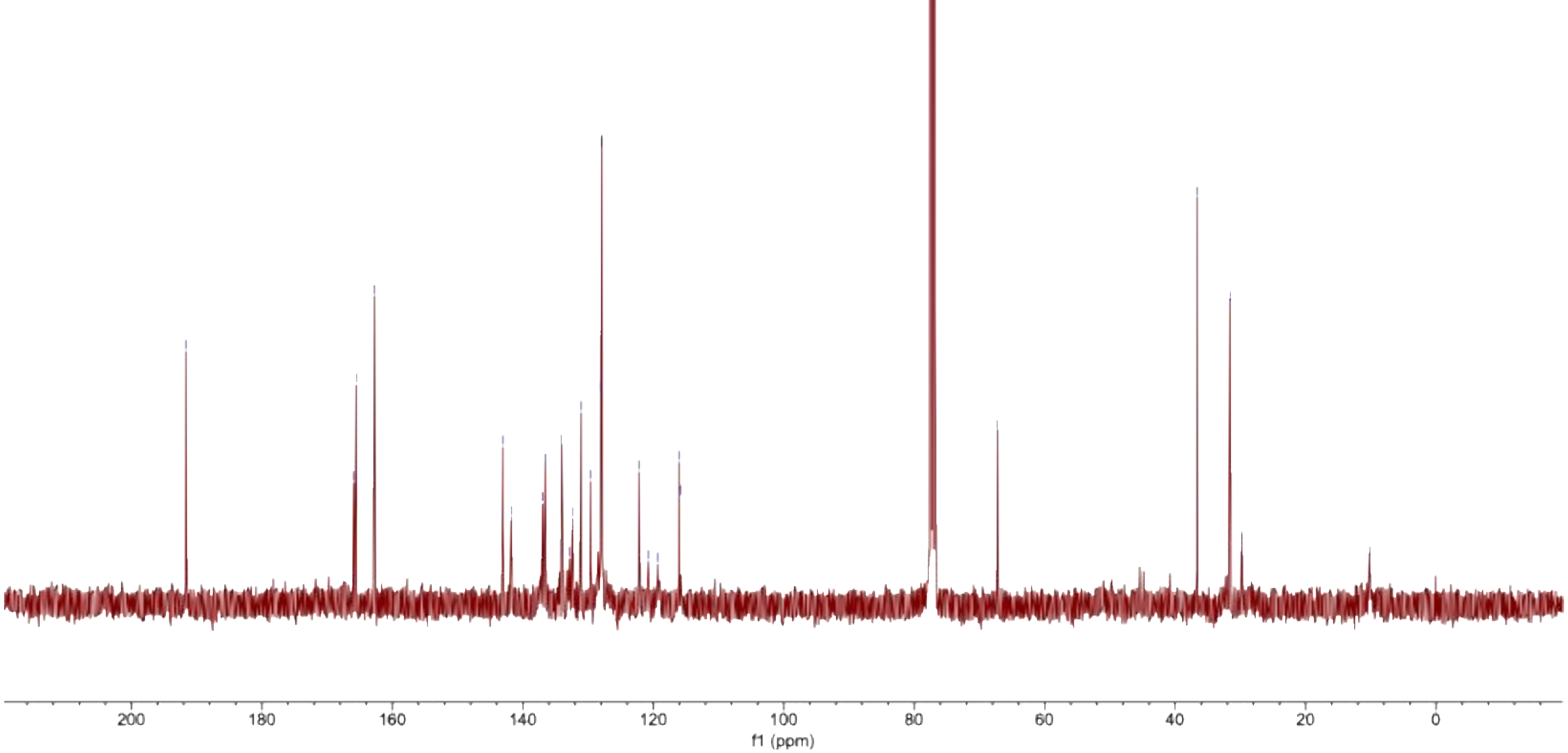

^{13}C NMR spectrum of T23

DMSO, 400.13 MHz

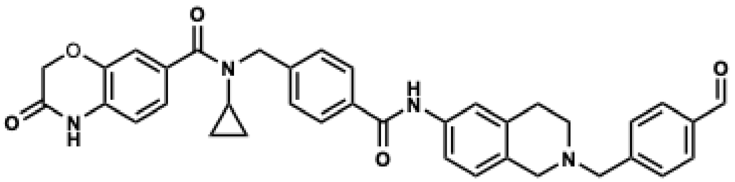

T24

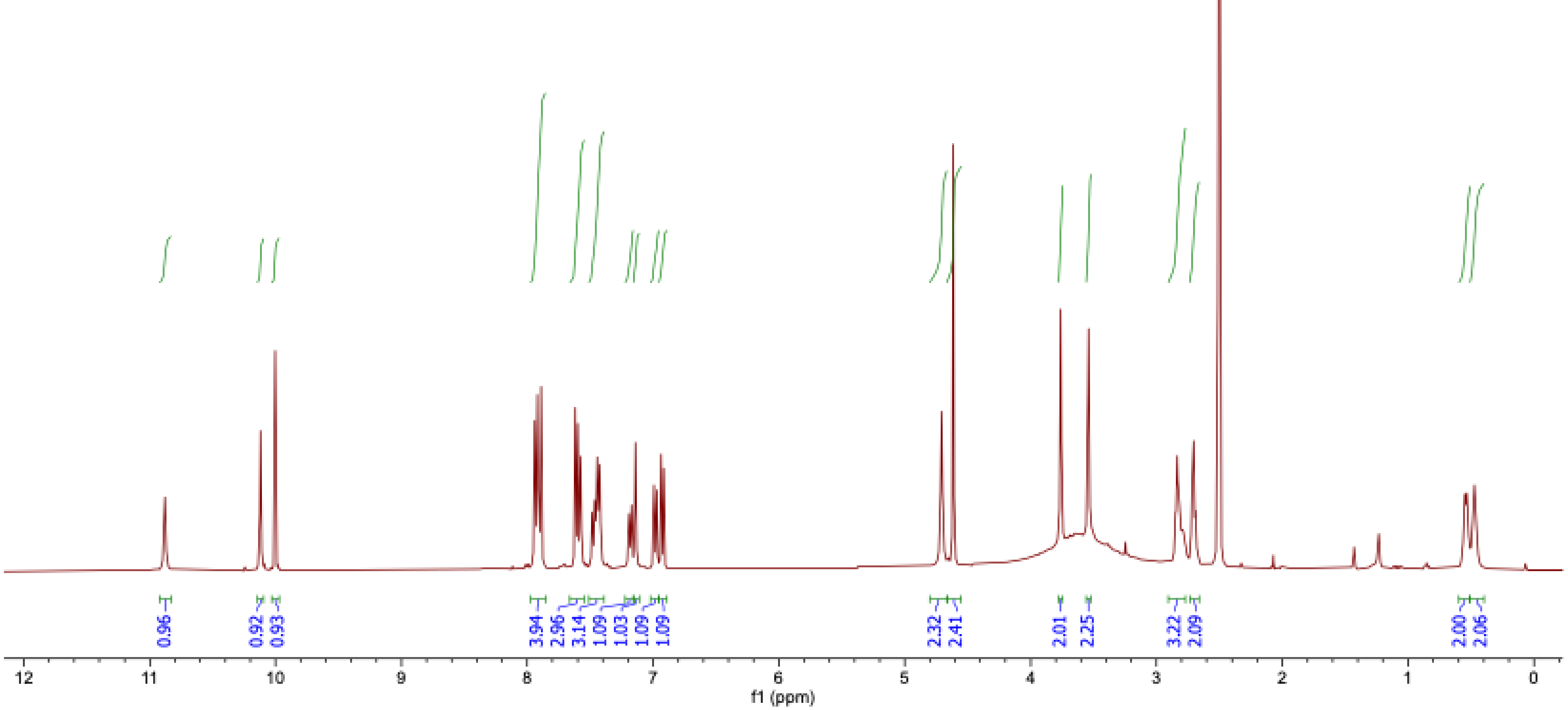

^{1}H NMR spectrum of T24

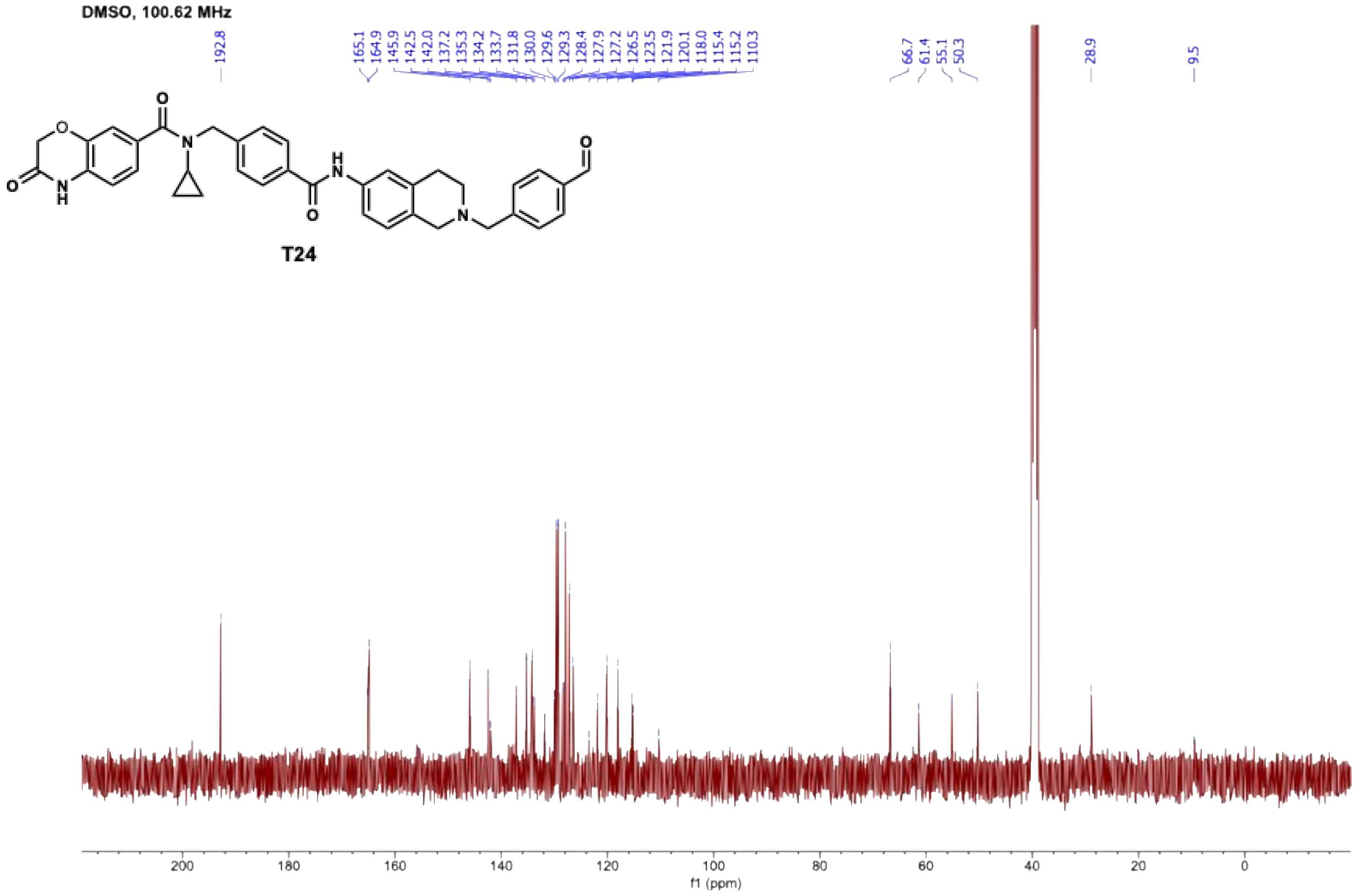

^{13}C NMR spectrum of T24

DMSO, 400.13 MHz

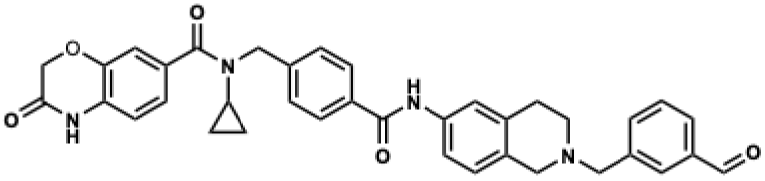

T25

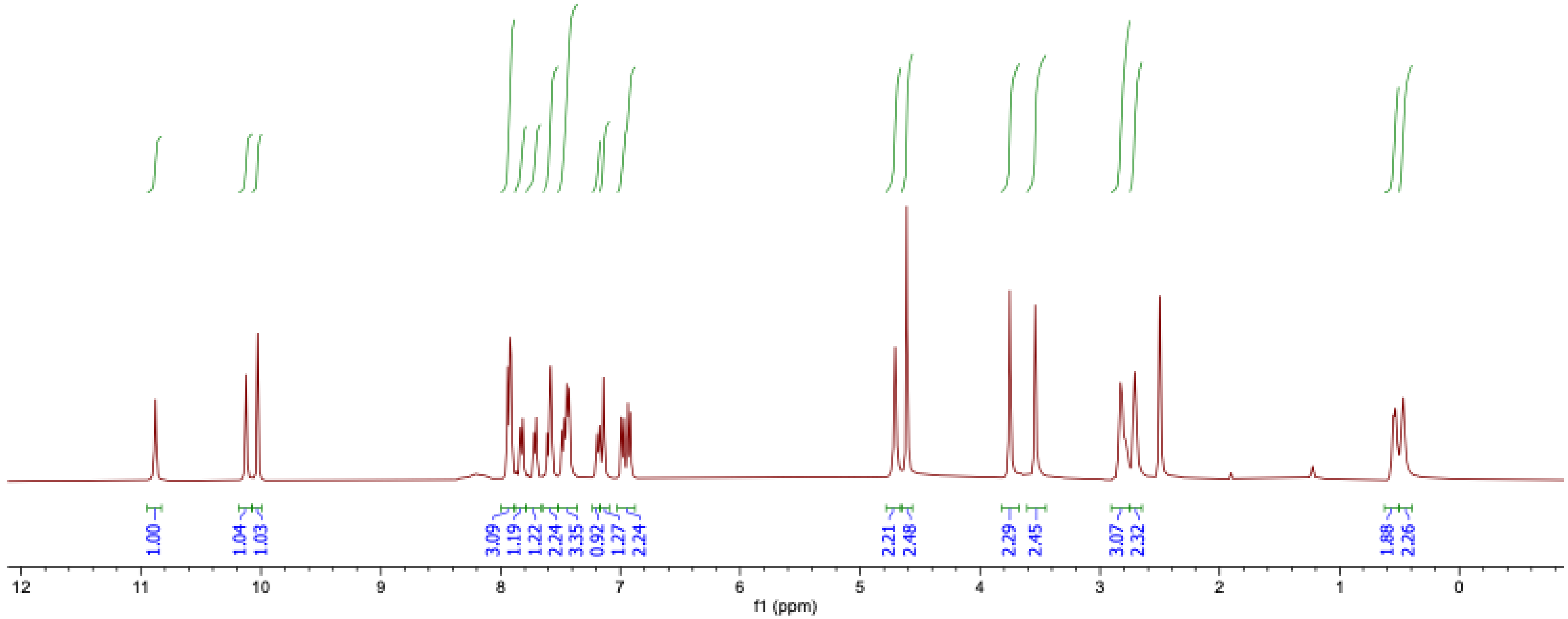

^{1}H NMR spectrum of T25

DMSO, 100.62 MHz

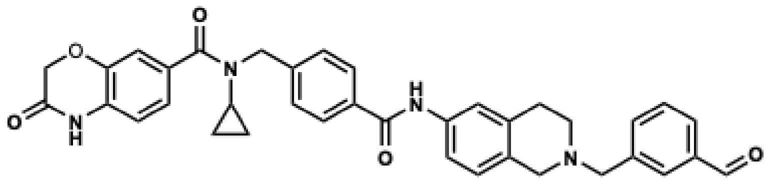

T25

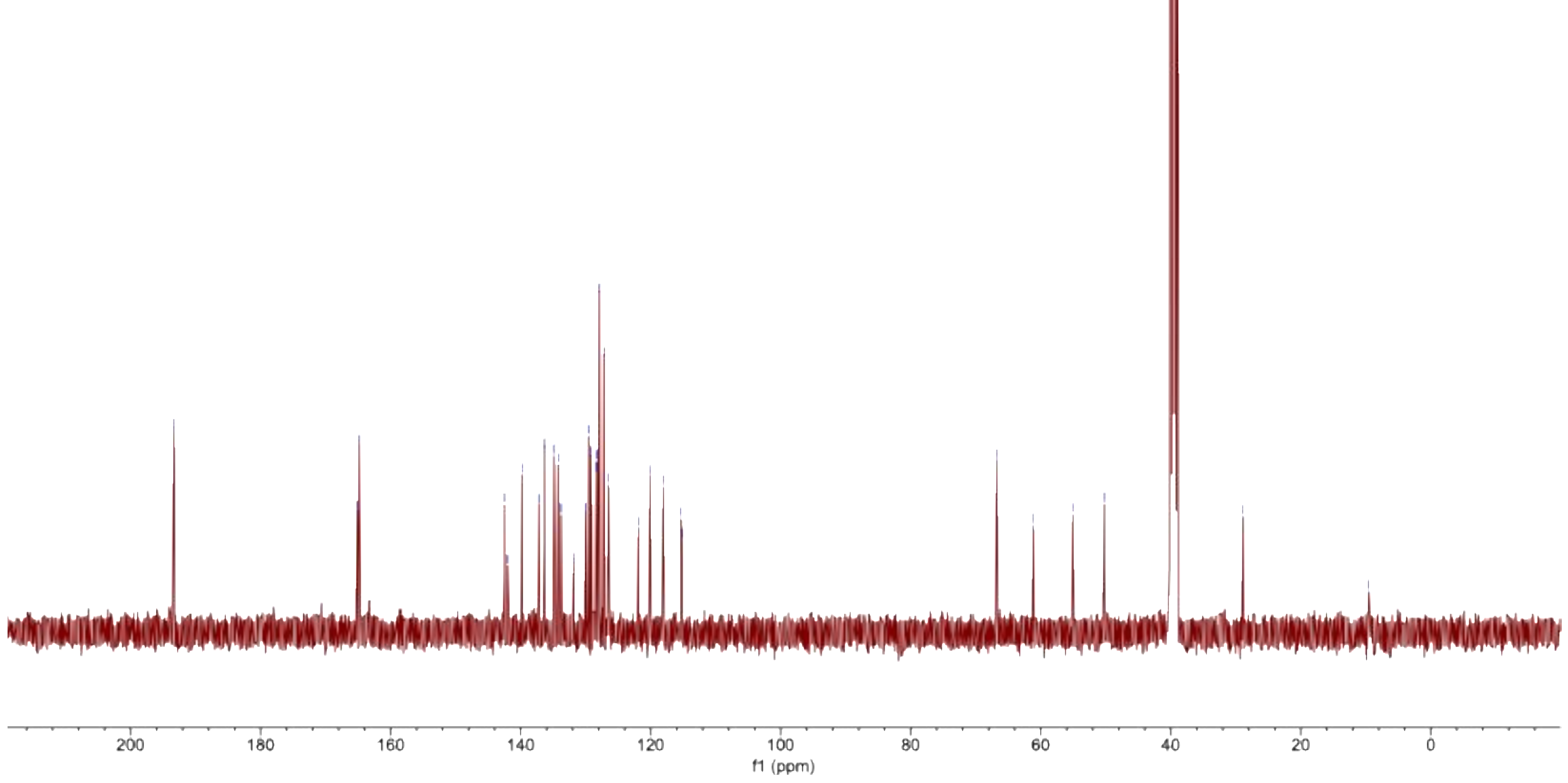

^{13}C NMR spectrum of T25

DMSO, 400.13 MHz

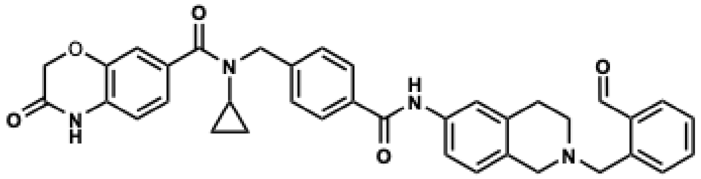

T26

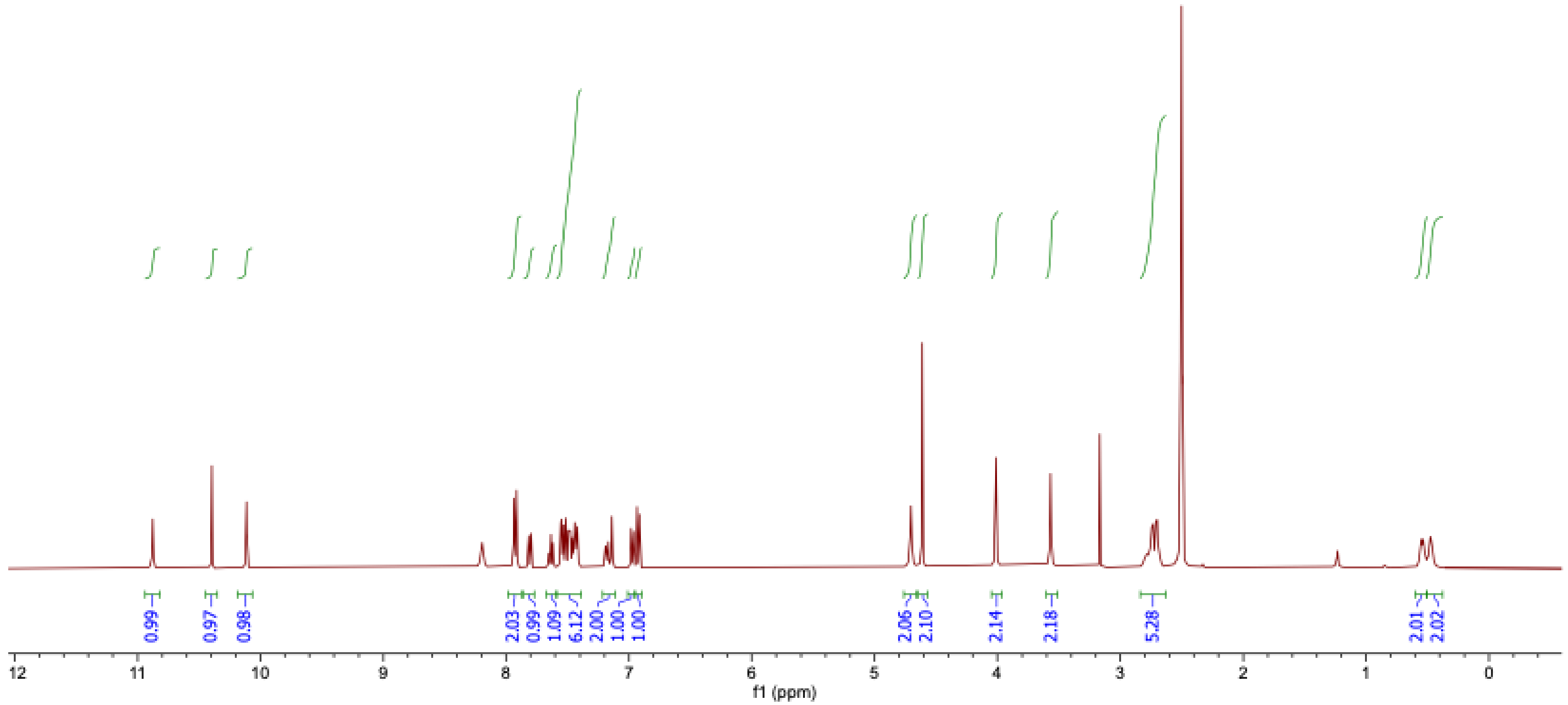

^{1}H NMR spectrum of T26

DMSO, 100.62 MHz

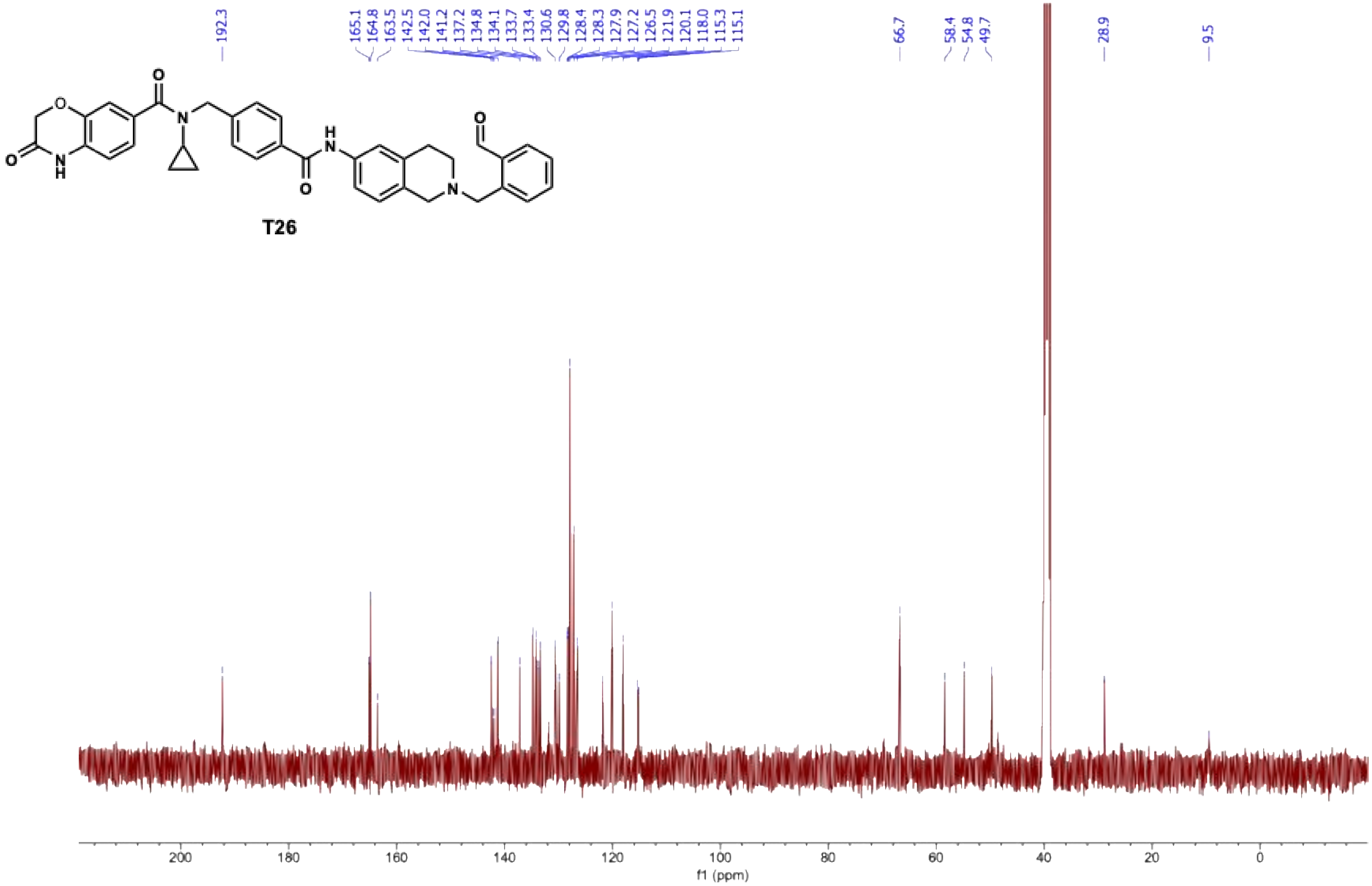

T26

^{13}C NMR spectrum of T26

DMSO, 400.13 MHz

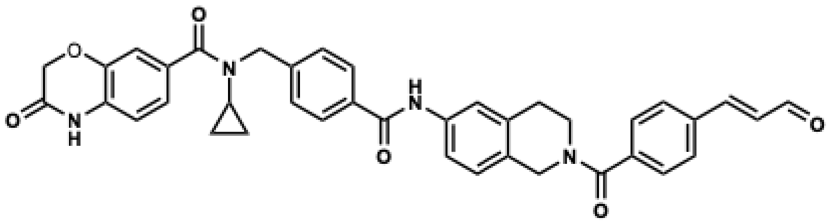

T27

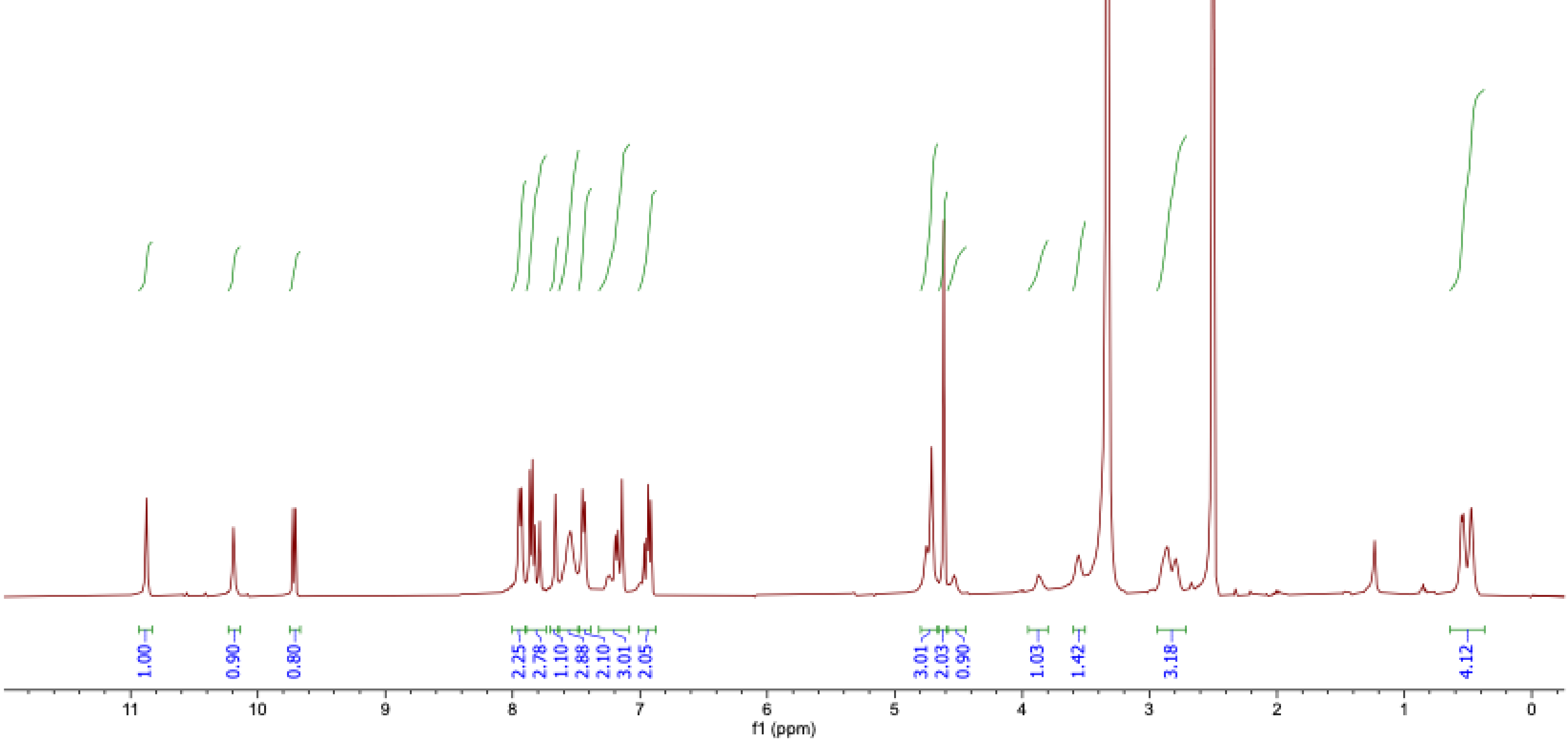

^{1}H NMR spectrum of T27

DMSO, 100.62 MHz

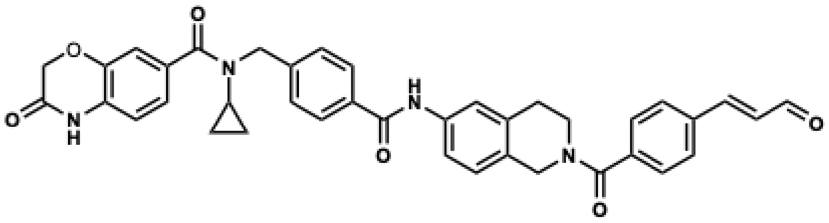

T27

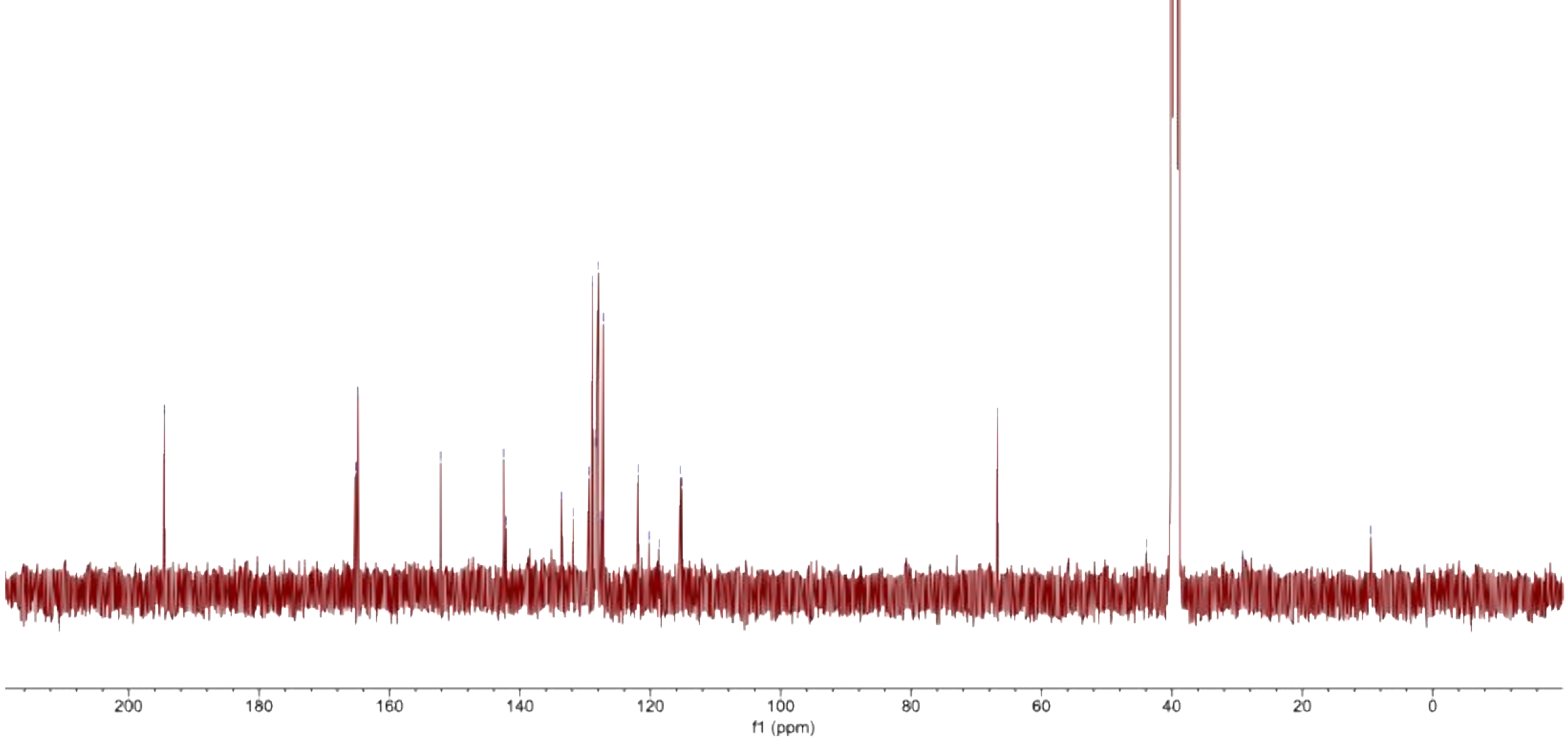

^{13}C NMR spectrum of T27

DMSO, 400.13 MHz

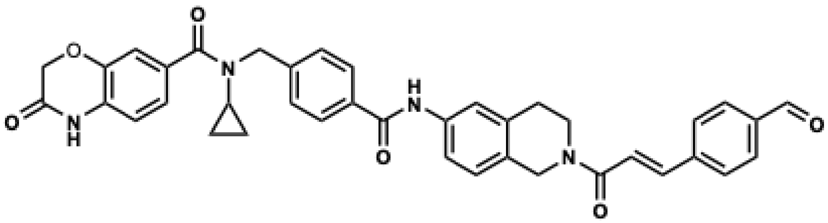

T28

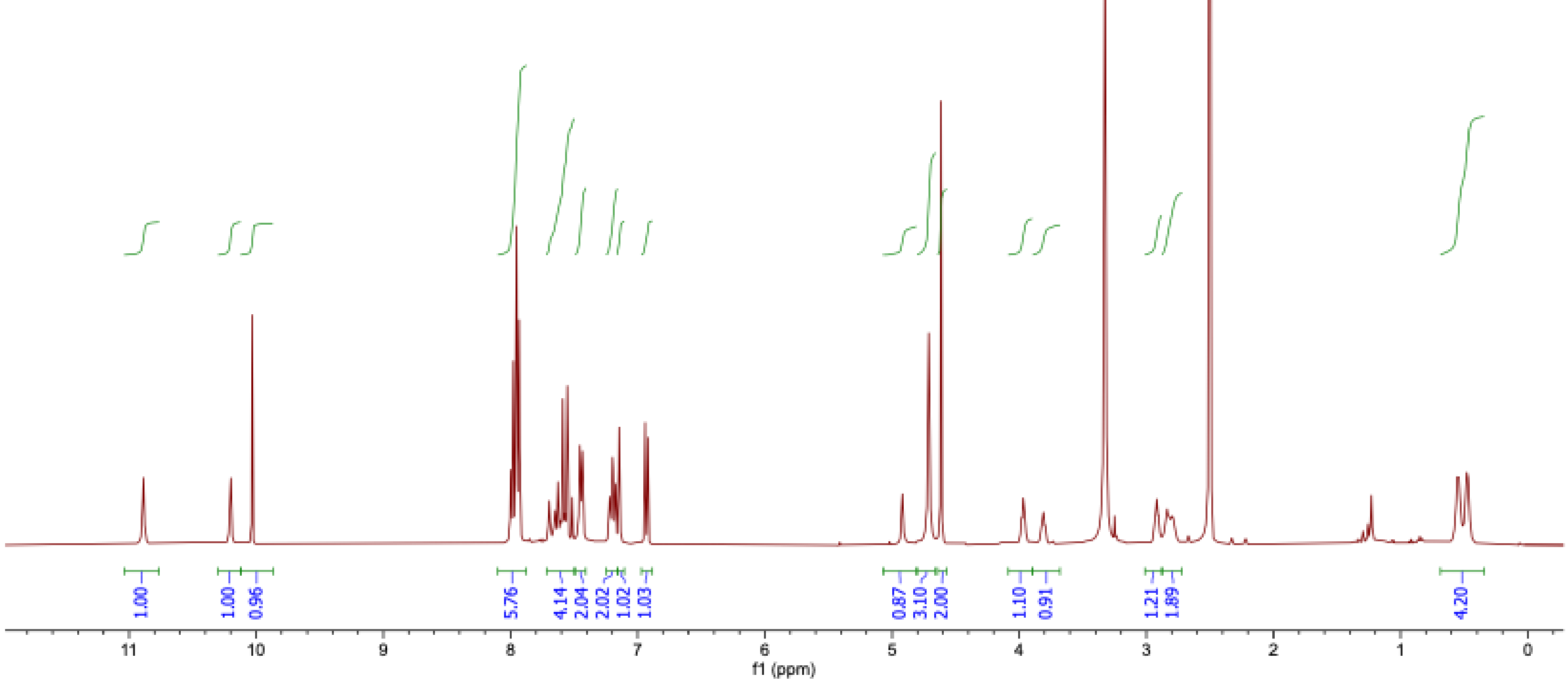

^{1}H NMR spectrum of T28

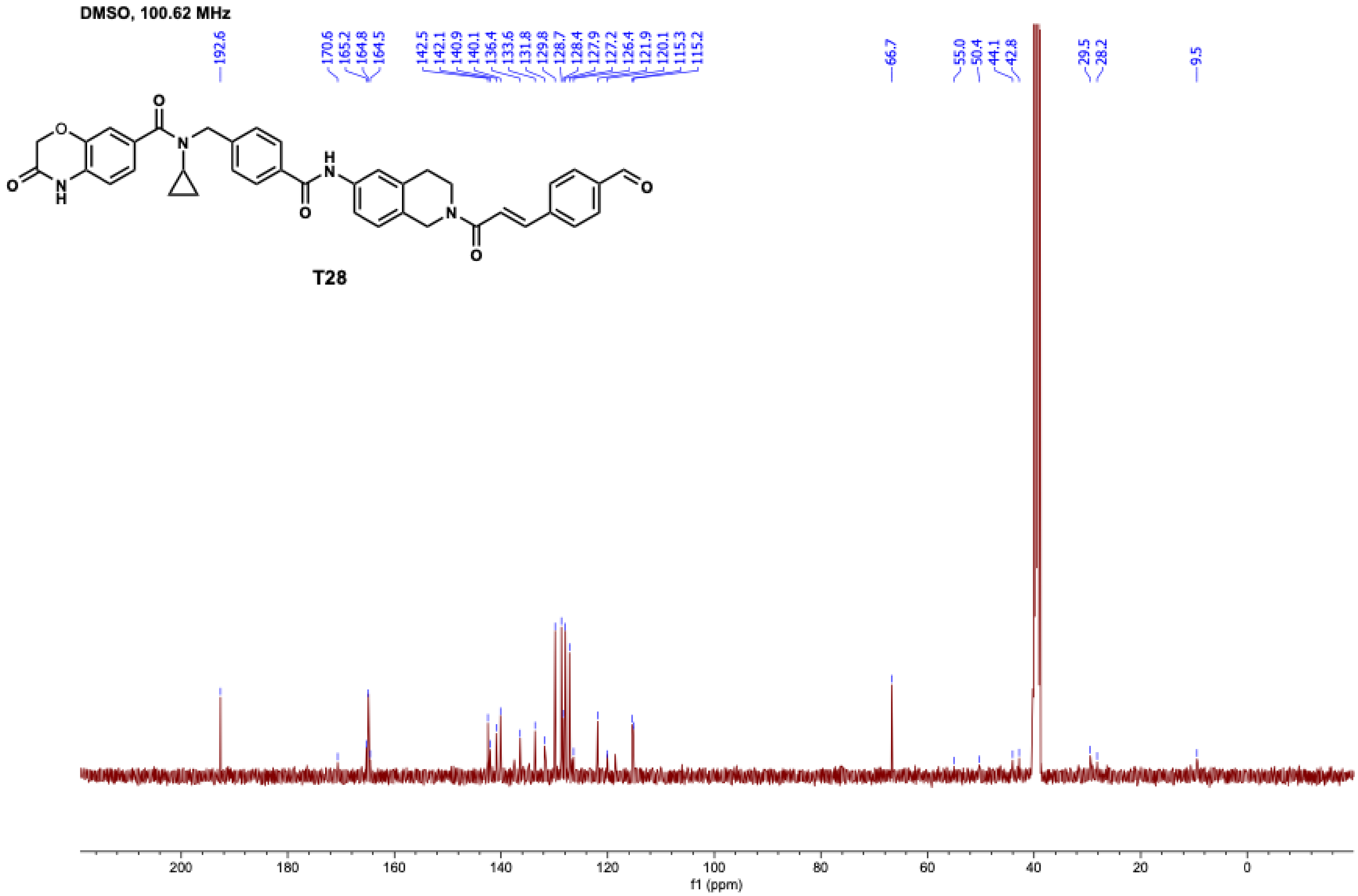

^{13}C NMR spectrum of T28

DMSO, 400.13 MHz

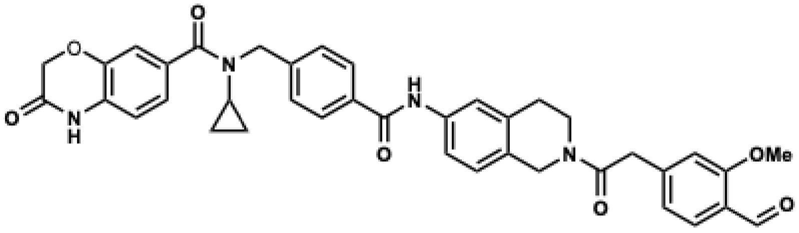

T29

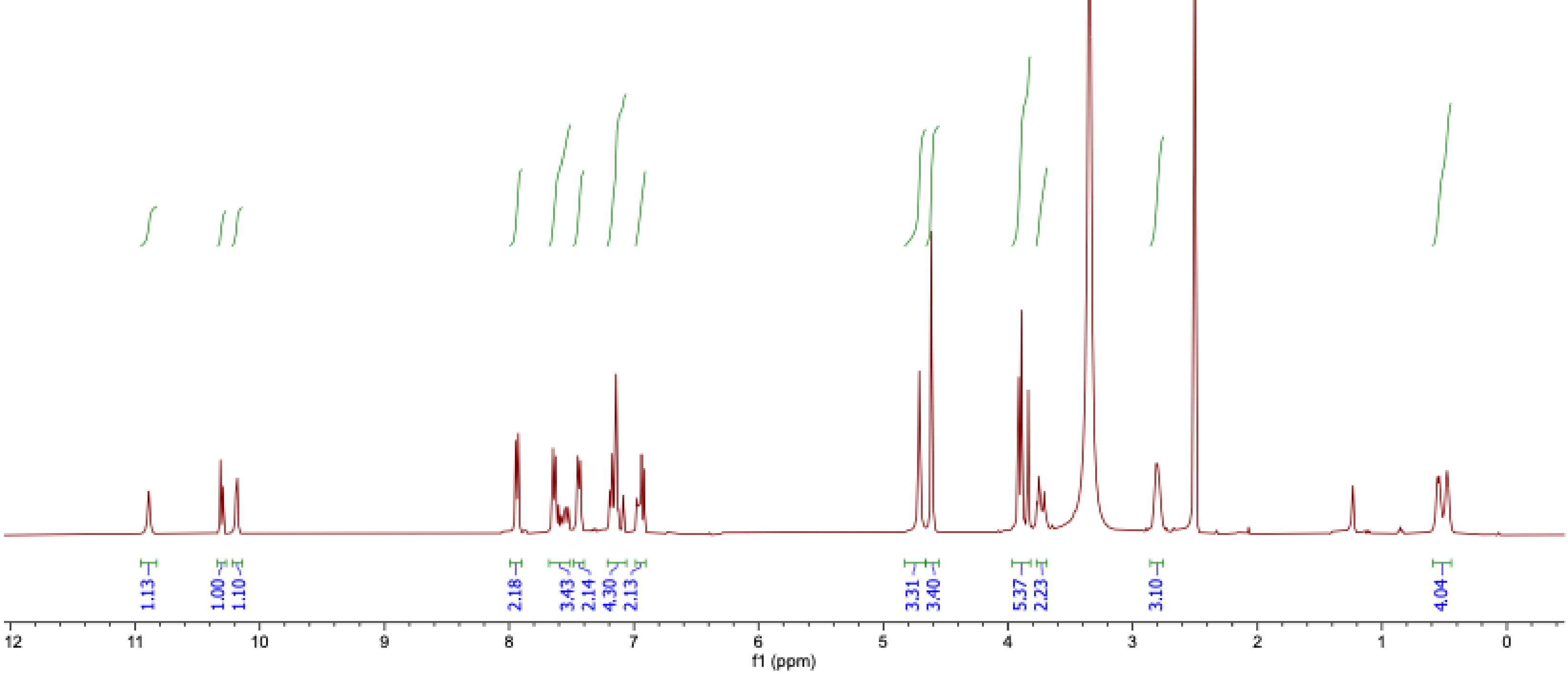

^{1}H NMR spectrum of T29

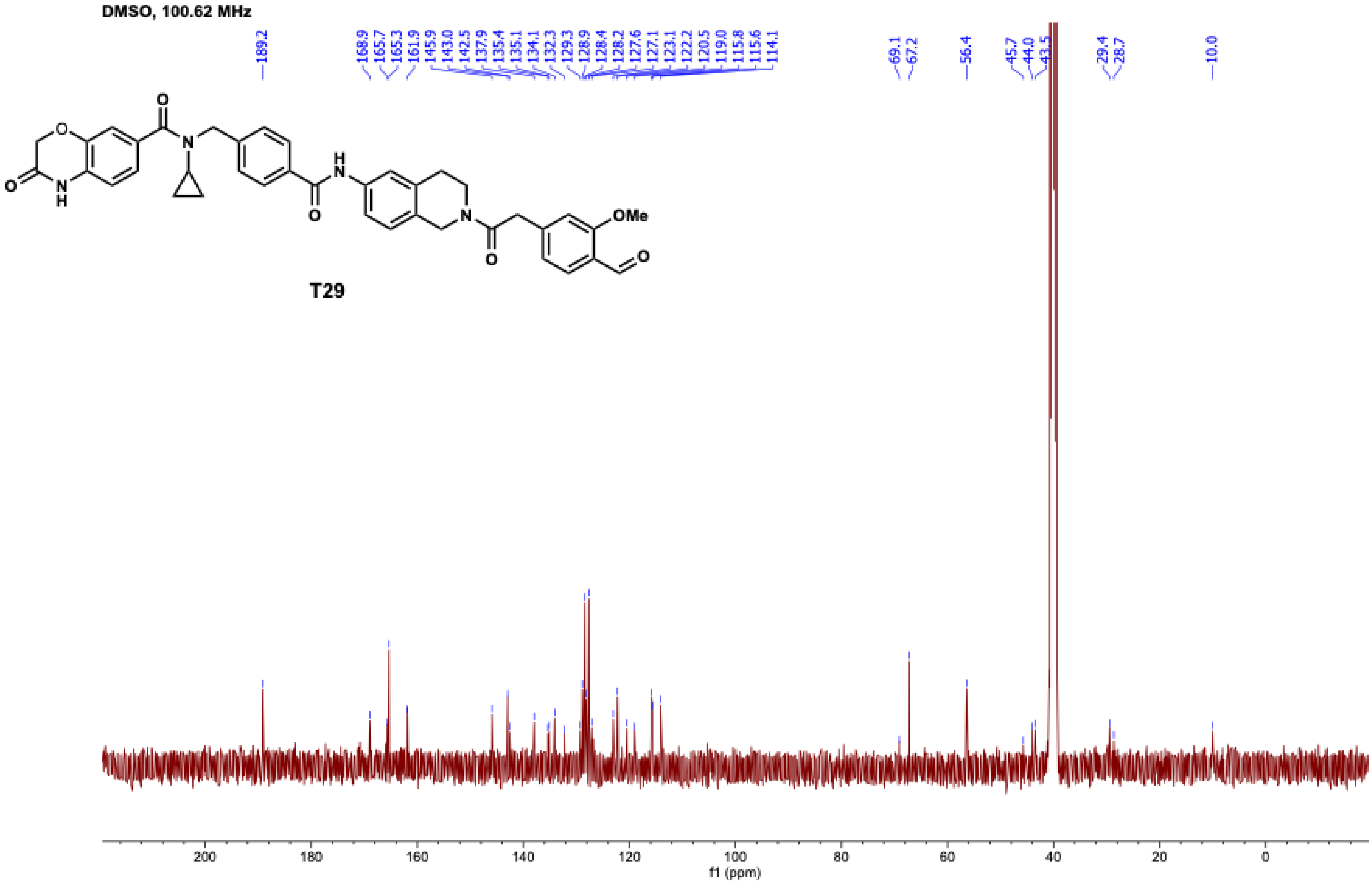

^{13}C NMR spectrum of T29

DMSO, 400.13 MHz

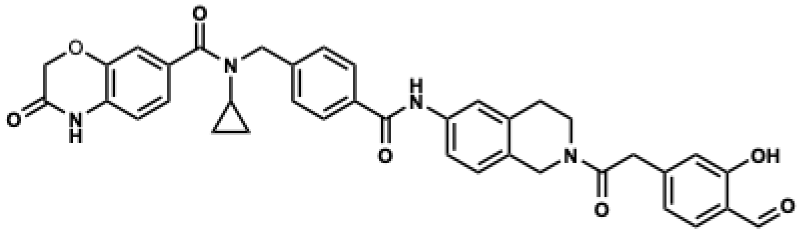

T30

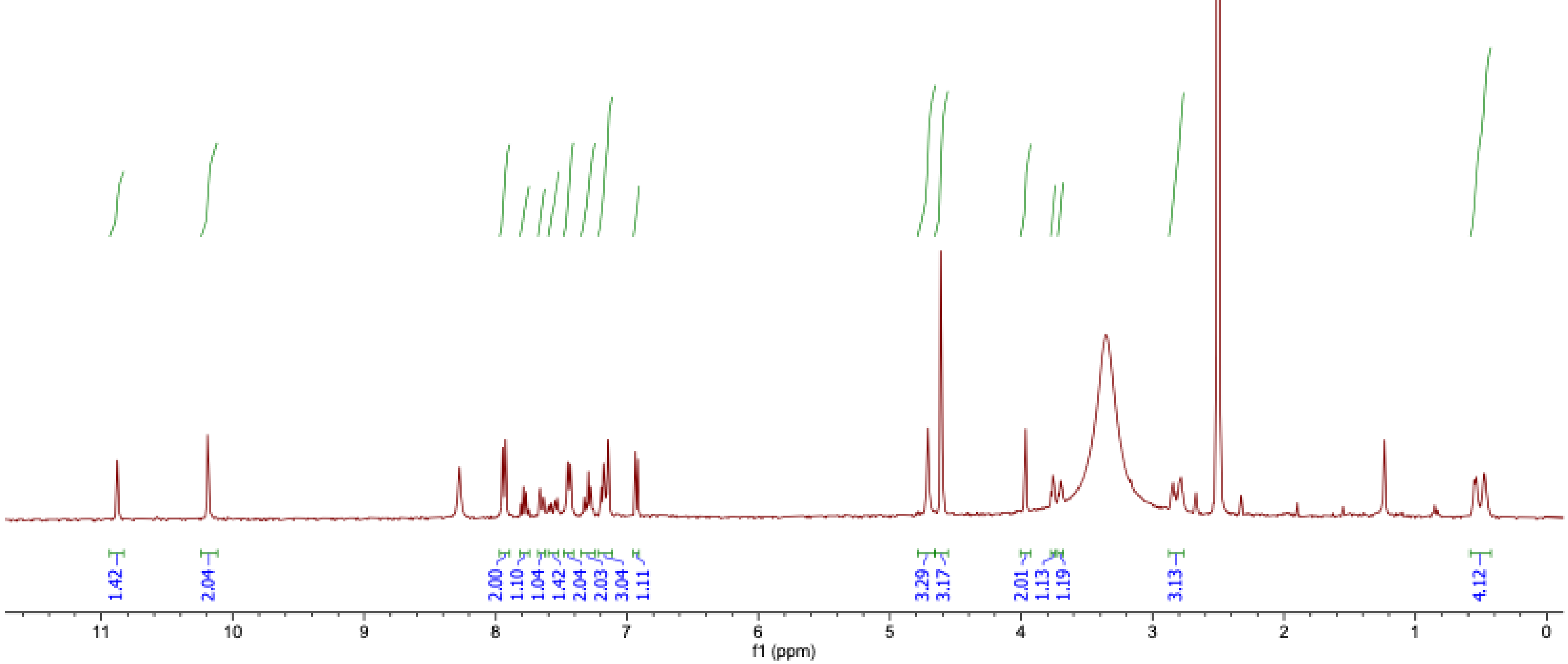

^{1}H NMR spectrum of T30

DMSO, 125.70 MHz

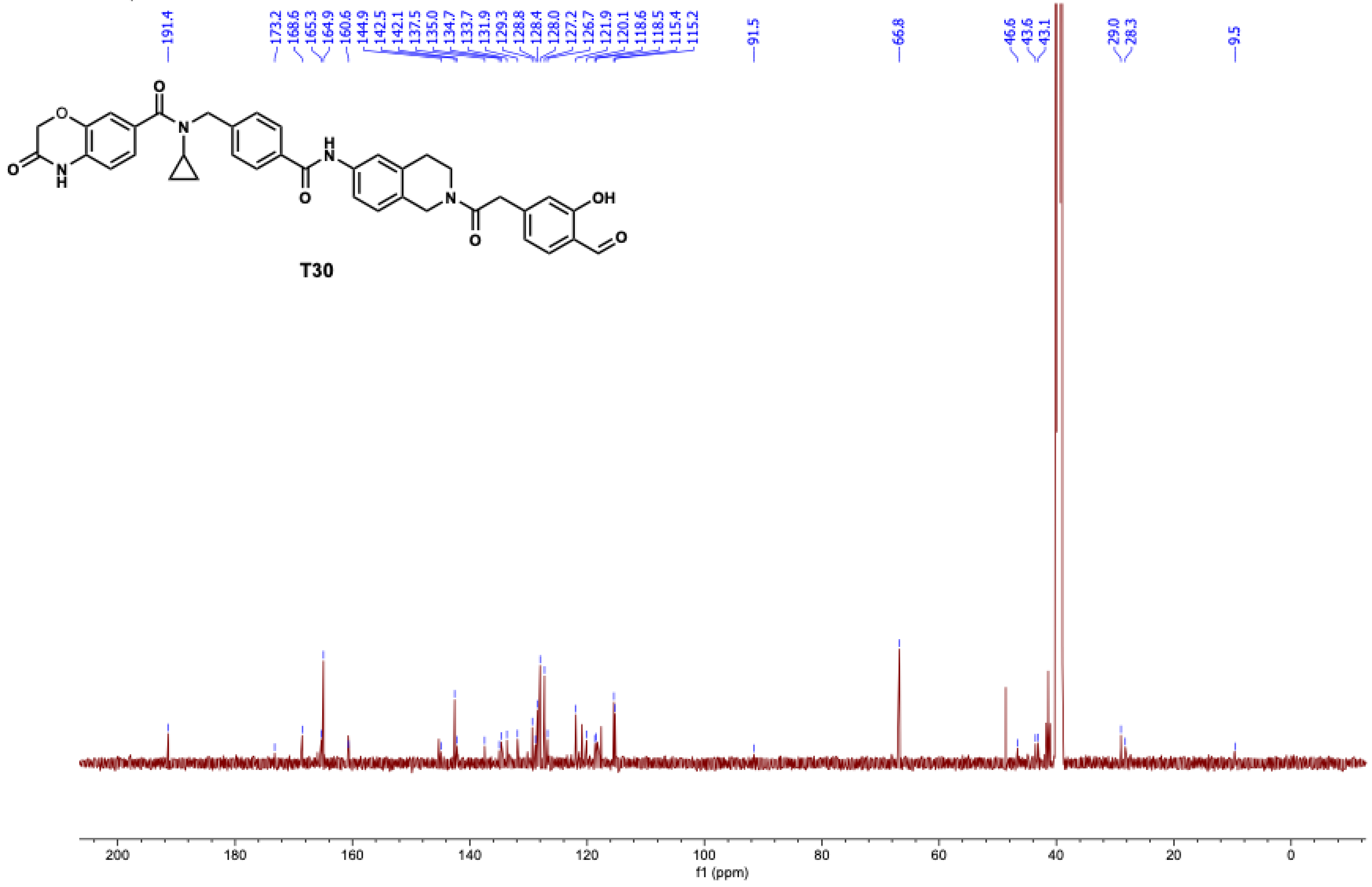

T30

^{13}C NMR spectrum of T30

DMSO, 400.13 MHz

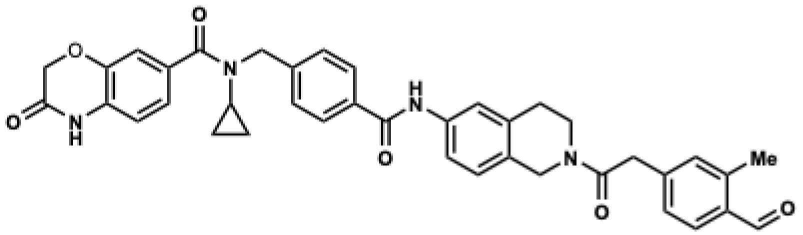

T31

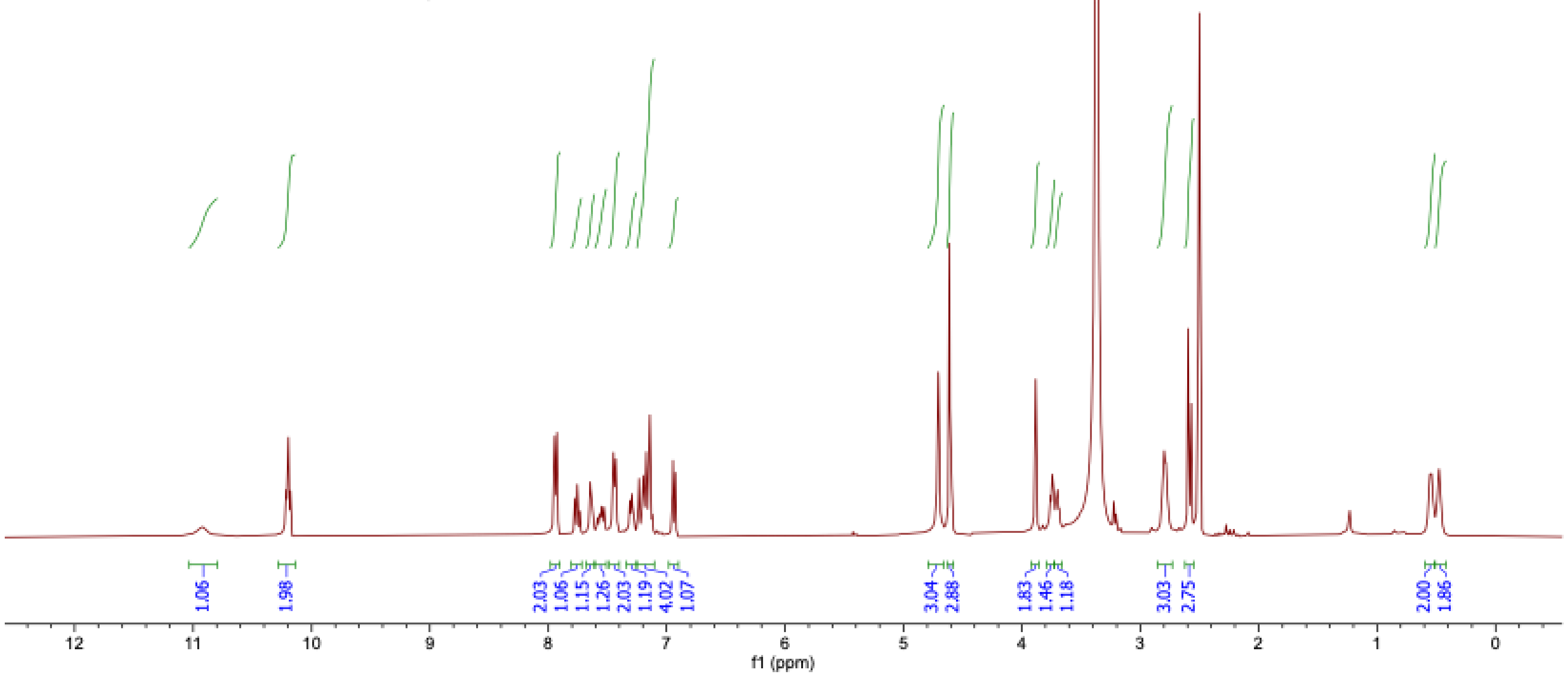

^{1}H NMR spectrum of T31

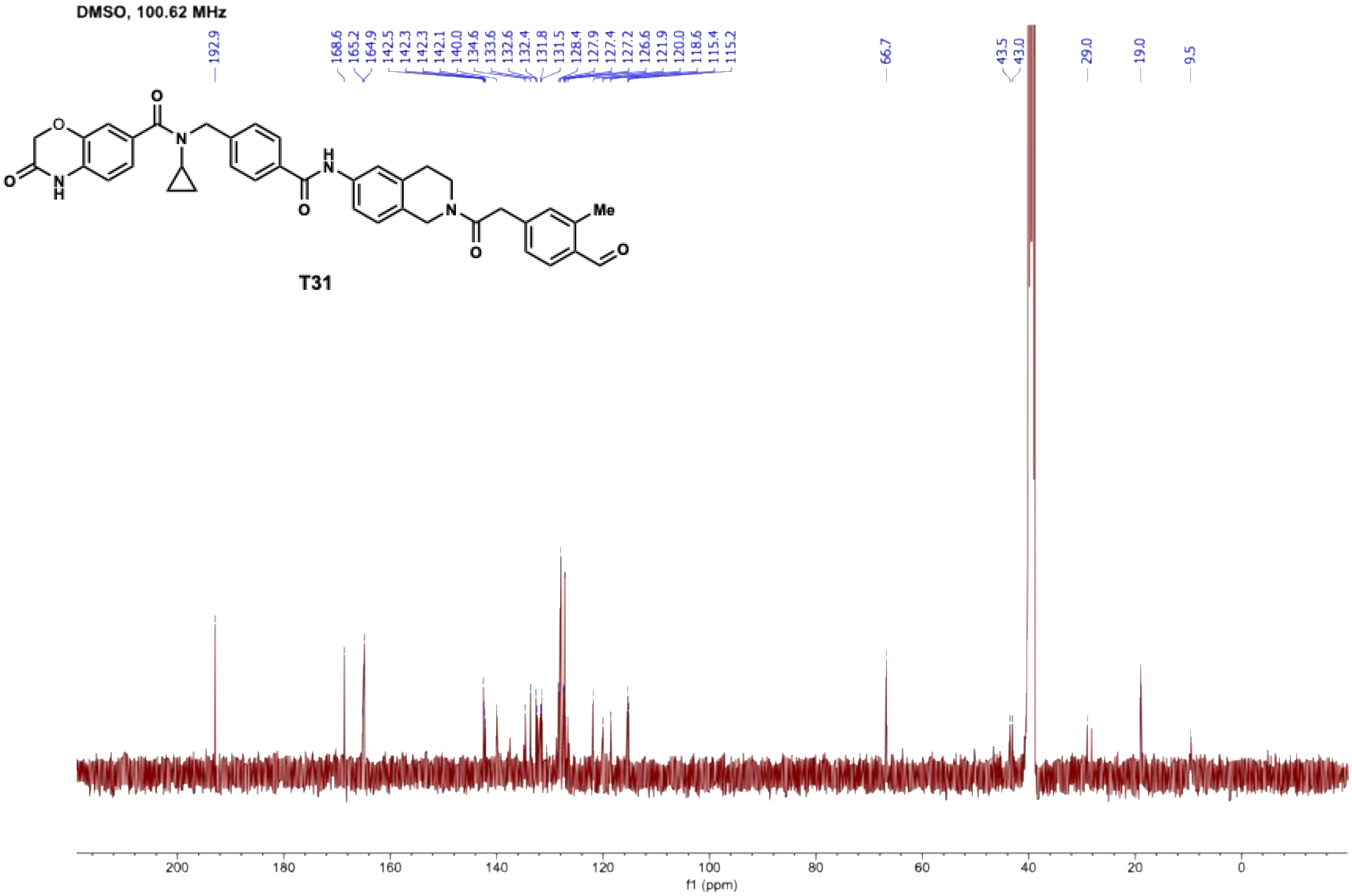

^{13}C NMR spectrum of T31

DMSO, 400.13 MHz

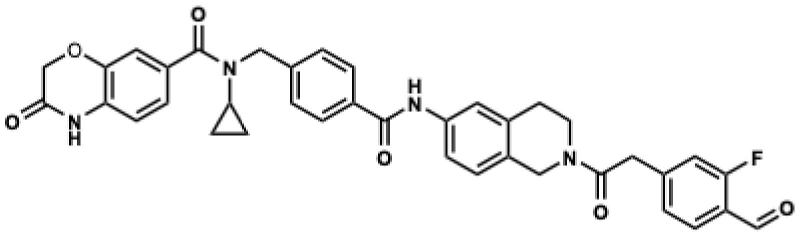

T32

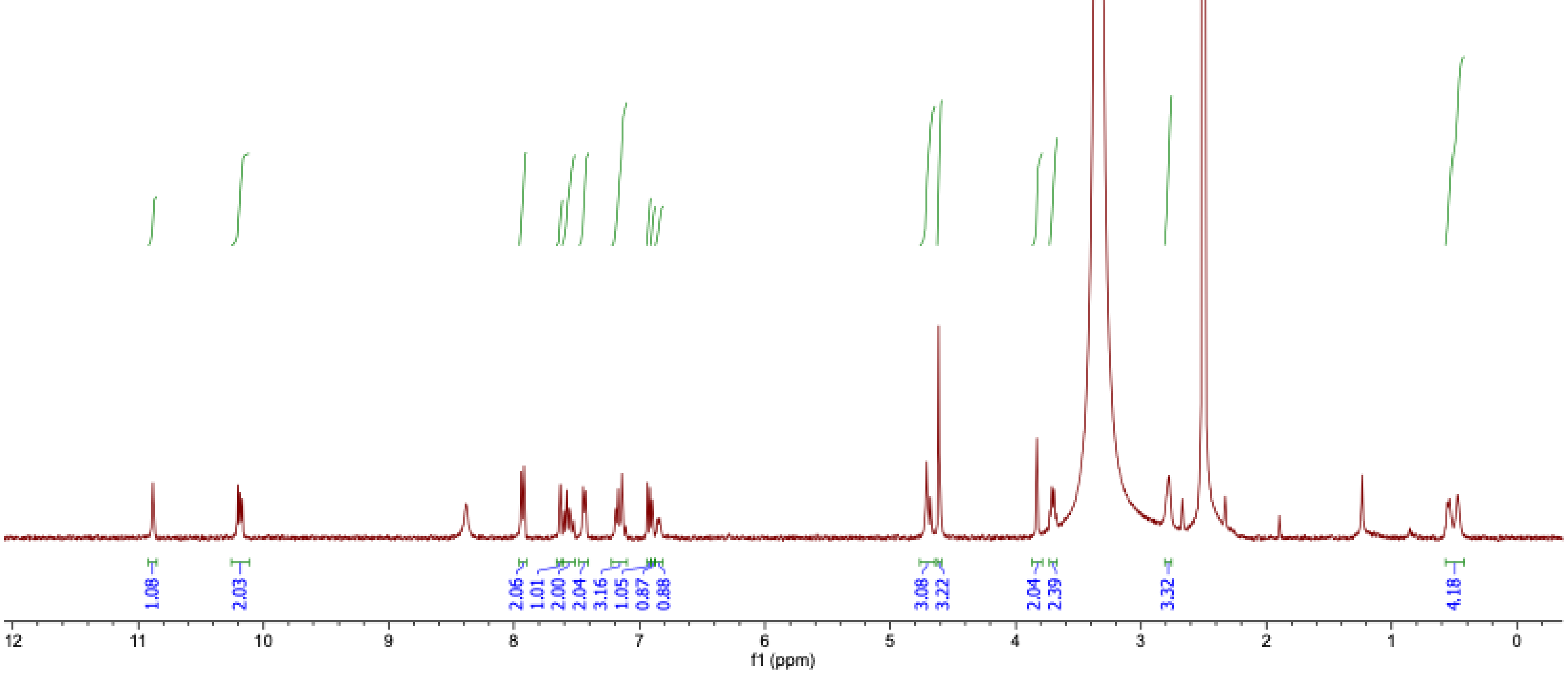

^{1}H NMR spectrum of T32

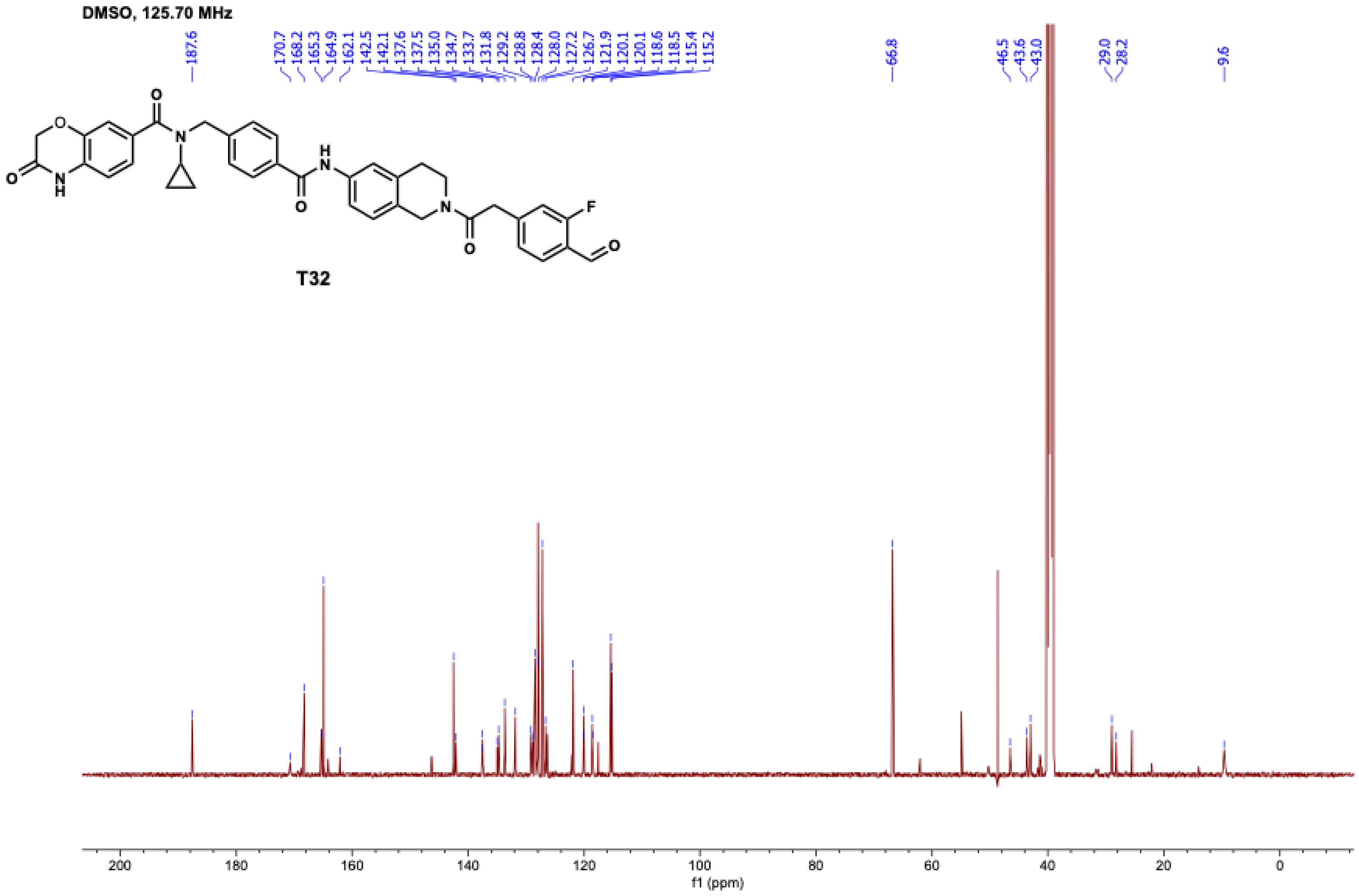

^{13}C NMR spectrum of T32

DMSO, 400.13 MHz

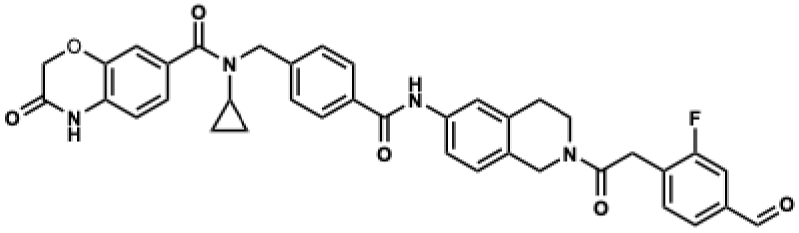
T33

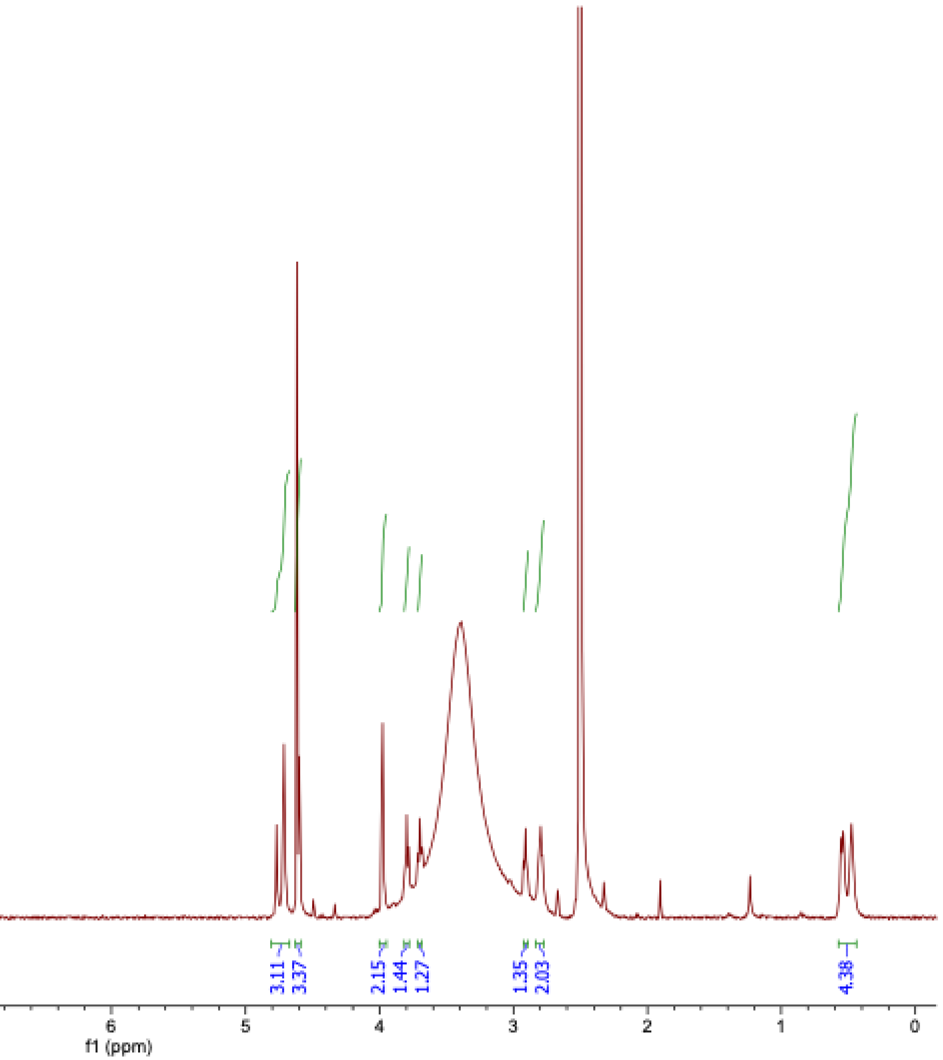

^{1}H NMR spectrum of T33

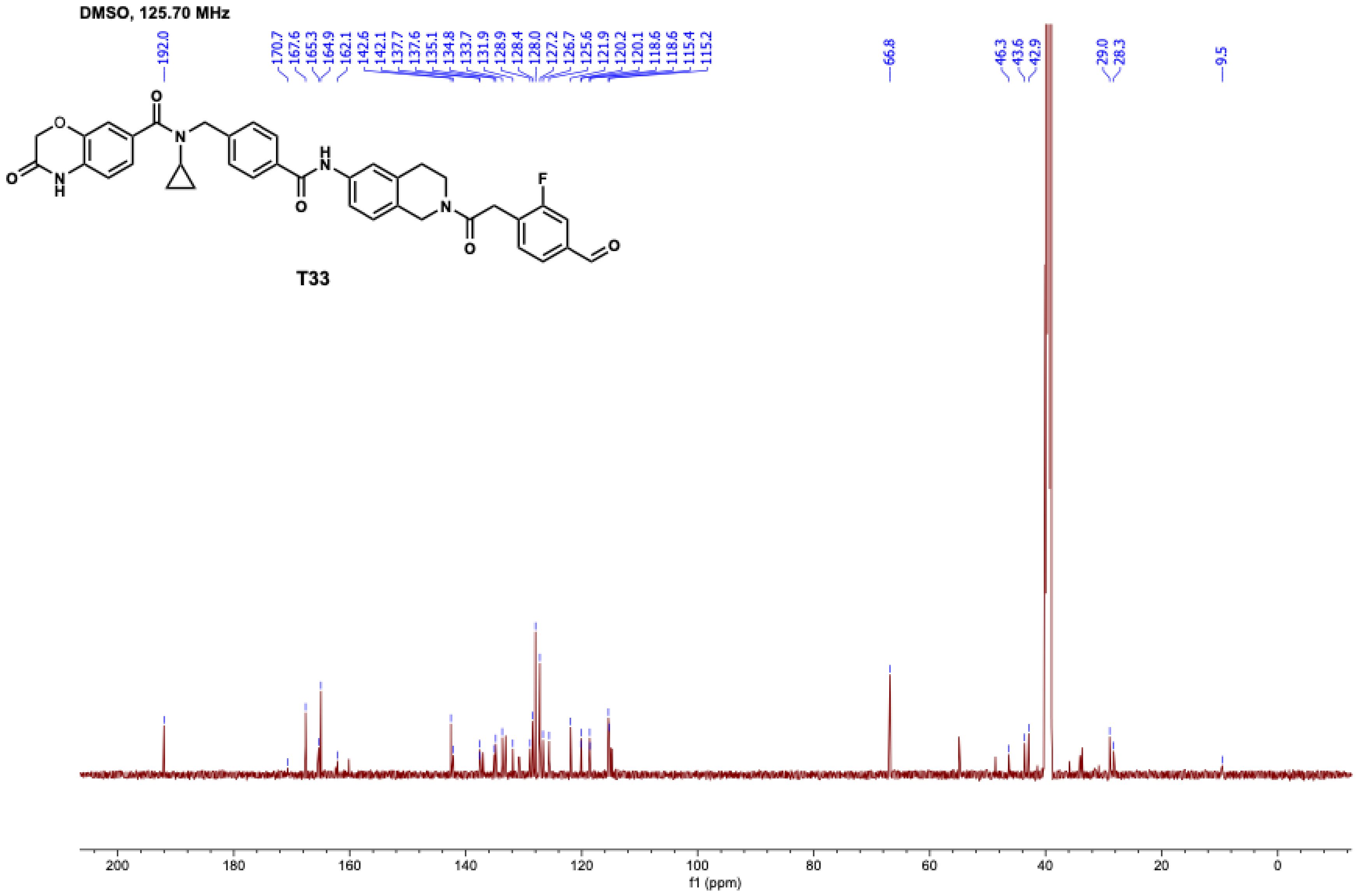

^{13}C NMR spectrum of T33

DMSO, 400.13 MHz

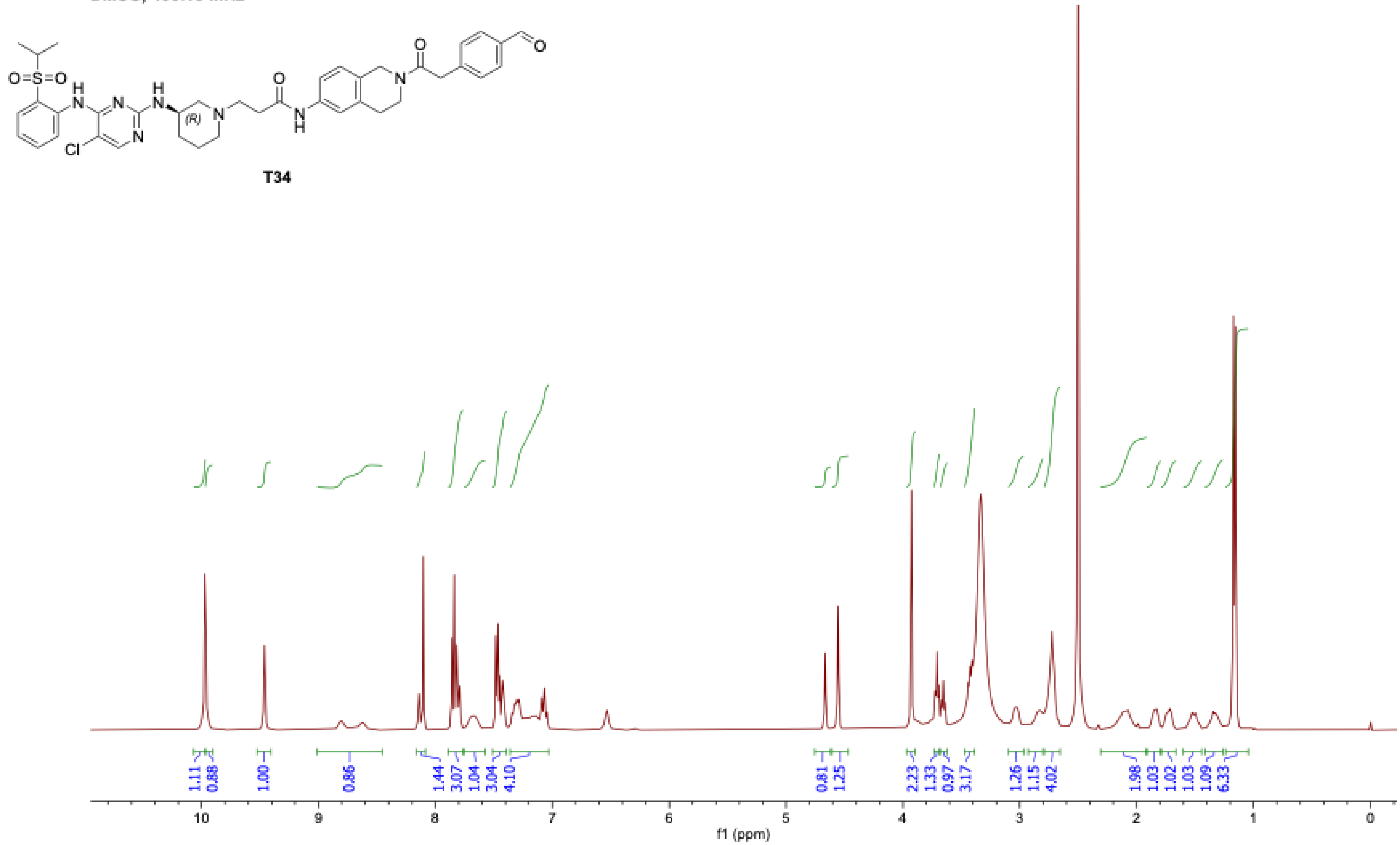

^{1}H NMR spectrum of T34

DMSO, 100.62 MHz

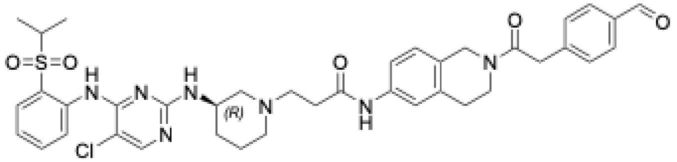

T34

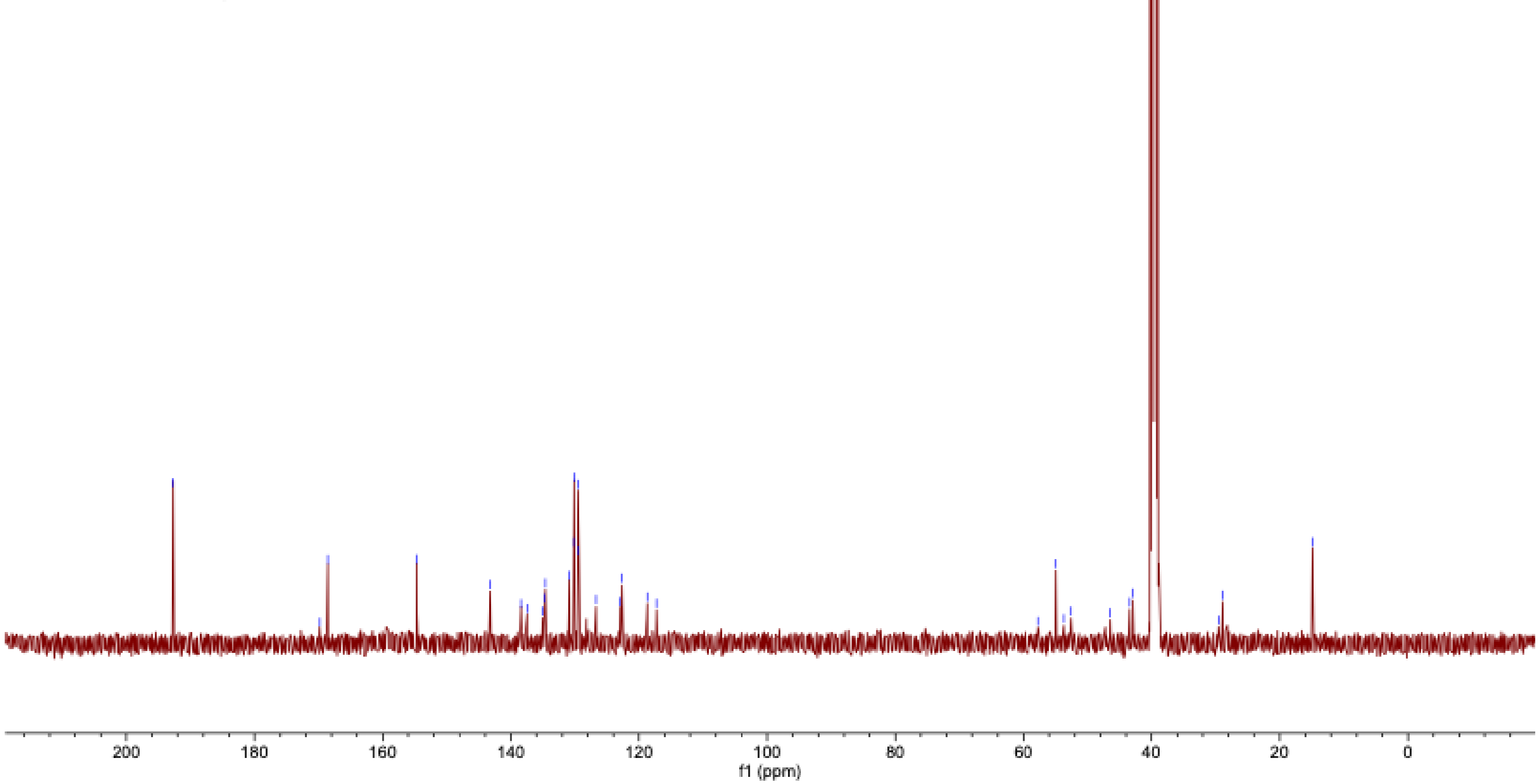

^{13}C NMR spectrum of T34
